# Real-time inverse kinematics for the upper limb: a model-based algorithm using segment orientations

**DOI:** 10.1186/s12938-016-0291-x

**Published:** 2017-01-17

**Authors:** Bence J. Borbély, Péter Szolgay

**Affiliations:** Faculty of Information Technology and Bionics, Pázmány Péter Catholic University, Práter Street 50/a, Budapest, 1083 Hungary

**Keywords:** Inverse kinematics (IK), Inertial measurement unit (IMU), Real-time, Upper limb, OpenSim, Embedded systems, Wearable

## Abstract

**Background:**

Model based analysis of human upper limb movements has key importance in understanding the motor control processes of our nervous system. Various simulation software packages have been developed over the years to perform model based analysis. These packages provide computationally intensive—and therefore off-line—solutions to calculate the anatomical joint angles from motion captured raw measurement data (also referred as inverse kinematics). In addition, recent developments in inertial motion sensing technology show that it may replace large, immobile and expensive optical systems with small, mobile and cheaper solutions in cases when a laboratory-free measurement setup is needed. The objective of the presented work is to extend the workflow of measurement and analysis of human arm movements with an algorithm that allows accurate and real-time estimation of anatomical joint angles for a widely used OpenSim upper limb kinematic model when inertial sensors are used for movement recording.

**Methods:**

The internal structure of the selected upper limb model is analyzed and used as the underlying platform for the development of the proposed algorithm. Based on this structure, a prototype marker set is constructed that facilitates the reconstruction of model-based joint angles using orientation data directly available from inertial measurement systems. The mathematical formulation of the reconstruction algorithm is presented along with the validation of the algorithm on various platforms, including embedded environments.

**Results:**

Execution performance tables of the proposed algorithm show significant improvement on all tested platforms. Compared to OpenSim’s Inverse Kinematics tool 50–15,000x speedup is achieved while maintaining numerical accuracy.

**Conclusions:**

The proposed algorithm is capable of real-time reconstruction of standardized anatomical joint angles even in embedded environments, establishing a new way for complex applications to take advantage of accurate and fast model-based inverse kinematics calculations.

**Electronic supplementary material:**

The online version of this article (doi:10.1186/s12938-016-0291-x) contains supplementary material, which is available to authorized users.

## Background

Quantitative movement analysis is a key concept in understanding processes of the human movement system. Evolved, high precision measurement devices have advanced research activity in movement rehabilitation [1–4], performance analysis of athletes [5] and general understanding of the motor system [6–9] during the last decades by making movement pattern comparison possible. This advancement was further accelerated by model-based analysis approaches that enabled explicit characterization of the studied movement patterns [10–15].

The most widely used measurement systems apply line-of-sight (LoS) methods (optical or ultrasound-based) that require a fixed *marker-sensor* structure. In these cases passive (optical) or active (ultrasound) markers are placed on anatomically relevant locations of the studied subject. Having the markers in place, measurements have to be performed in a specialized laboratory environment where locations and orientations of the sensor elements (i.e. cameras or microphones) are known and invariant (at least across trials). This means that even the spatial positions of the markers can be determined with good accuracy—especially with optical systems—the possible range of motion will always be constrained by the actual measurement volume covered by the sensors of the system. Although this property is not an issue for many movement analysis scenarios, there are cases when a measurement method allowing unconstrained free space movement would be more beneficial (e.g. various outdoor activities or ergonomic assessment of work environments, to name a few).

Advancements in the field of inertial sensor technology have given rise to new development directions in laboratory-free movement analysis methods. The main difference between LoS and inertial systems is the recorded modality: while LoS methods determine the *spatial locations* of markers based on planar position (optical) or timing (ultrasound) information, inertial sensors give their *orientation* in space by measuring physical quantities acting on them directly. These quantities are linear acceleration and angular velocity in most cases while they are supplemented with magnetic field measurements in more complete setups. To obtain orientation from raw inertial measurements, various sensor fusion algorithms have been developed utilizing Kalman-filters [16, 17], gradient descent methods [18], complementary filters [19, 20] and other techniques [21], most of them being capable for real-time operation in embedded systems. In addition, recent evolution of chip-scale inertial sensors based on MEMS technology further widened the possibilities of wearable measurement device development by making the core sensing elements available for better integration. While there is no gold standard among fusion algorithms and sensor chips as compromises have to be made in aspects of accuracy, system complexity and computational demand of the fusion algorithm, it can be stated that inertial sensor technology is taking a more and more growing part in human movement measurements (a good example for this progress is Xsens’ product portfolio).

In addition to accurate measurement, proper evaluation of the recorded motion is an other key building block of human movement analysis. Considering the kinematic assessment of upper limb movements by using inertial sensors, reliable methods have been proposed to estimate joint kinematics in custom local coordinate systems, also dealing with the problem of functional calibration and measurement error compensation [22–25]. While these methods may work well in movement analysis scenarios focusing only on the spacial kinematics of the upper limb, the goal of the present study is to extend the applicability of a more complex freely available upper limb model [26] with inertial measurement and real-time joint angle reconstruction capabilities. The chosen model includes 15 degrees of freedom and 50 muscle compartments and in addition to movement kinematics it enables the evaluation of muscle-tendon lengths, moment arms, muscle forces and joint moments in an anatomically reasonable setup (also conforming to the ISB recommendation [27] as those presented in [23] and [24]). Our motivation of choosing this approach instead of using direct joint kinematics estimates from inertial sensor data lies in the strong belief that the utilization of this upper limb model leads to better understanding of the additional inherent properties of arm movements (e.g. muscle activation patterns) compared to a pure kinematic investigation.

By using the model from [26], two main differences from the direct approach should be considered: (1) because the model was developed mainly for optical motion capture technology, joint angle reconstruction is less coupled with raw measurement data in the sense that it is performed as an inverse task based on locations of arbitrarily placed virtual markers (instead of relying on sensor orientations directly) and (2) as a consequence of the inverse kinematics approach, the model raises more computational demand for joint angle reconstruction. While the first point contributes to a possibly wider adoption of the model (i.e. measurement data of any reasonable source—being it optical, ultrasound based, inertial, etc.—only needs to be expressed in terms of virtual marker locations), the second can be considered as a drawback in certain situations when real-time joint angle reconstruction would be beneficial.

To analyze model-defined parameters of recorded movements, various software packages have been developed for biomechanical analysis over the past decades. Some of them are tightly integrated into their corresponding measurement system’s ecosystem with only kinematic reconstruction capabilities [28–30] while others are independent of a particular hardware setup (SIMM [31] and OpenSim [32]), even including analysis options for muscle activations and movement dynamics if the corresponding models are available. While these tools may address the joint angle reconstruction problem involving inverse kinematics in different ways resulting in varying performance, OpenSim [32] was chosen as the reference model-based analysis software for the purposes of the current study, because (as to the best of the authors’ knowledge) it is the only free and open source (thus widely accessible) option in the biomechanical modeling field.

Because the upper limb model used in this study was originally developed for the SIMM software, an other reason for using OpenSim was it’s ability to import and handle SIMM models natively, so combined with the model it provides a complete package for open source arm movement analysis. For kinematic evaluation, OpenSim uses the “standard” offline *measurement-scaling-inverse kinematics* pipeline where the actual biomechanical model (single limb to full body) is fitted to measurement data. During this process, positions of virtual markers placed on specific model segments are fitted to experimentally recorded marker positions of the subject with the same arrangement. Scaling is important to generate subject-specific model instances while inverse kinematics (IK) is performed to extract model-defined anatomical joint angles that produced the movement. OpenSim uses a text based structured XML model format that contains all information needed for the biomechanical description of the human body [bodies, kinematic constraints and forces (i.e. muscles)] that are accessible through API calls, too.

Complex measurement and analysis of upper limb movements including kinematics and muscle activities is an exciting and growing subfield of human movement analysis [33–37] that promises better understanding of control patterns during specific movements, and as an example benefit may—on the longer term—advance control techniques currently applied to arm and hand prostheses. This process however needs tighter integration of kinematic measurement and reconstruction (from raw data to anatomical joint angles) as the time and computational overhead of the offline *measurement-scaling-inverse kinematics* scheme gives a bottleneck in applications where real-time analysis of the control patterns with respect to the actual kinematics would be beneficial.

As a specific example, let us consider a supervised learning based muscle activity classification framework like the one proposed in [38] that is built around the increasingly popular concept of deep neural networks [39]. The goal of this setup is to assess the classification capability of various network architectures in estimating upper limb movement kinematics based only on muscle activity recordings. The large amount of labeled data that is needed to train these networks (even iteratively after initial deployment) should be presented by a method that performs muscle activity data labeling based on the actual (anatomically relevant) kinematic state of the limb automatically as the measurements occur. By using the approach proposed in this paper, labeled measurement data could be collected in real-time and simultaneously from multiple recording devices and a high level of automation might be reached in the process of measurement, network training and deployment.

### Purpose of the study

The main goal of this study is to extend the measurement and analysis workflow of human arm movements with a method that allows accurate and real-time calculation of anatomical joint angles for a widely used upper limb model in cases when inertial sensors are used for movement recording. For this purpose a custom kinematic algorithm is introduced that utilizes orientation information of arm segments to perform joint angle reconstruction. Accuracy and execution times of the proposed algorithm are validated against the most widely available biomechanic simulation software’s inverse kinematics algorithm on various platforms.

## Methods

### Upper limb model

To analyze arm kinematics with OpenSim the most complete model available was chosen known as the Stanford VA Upper Limb Model [26]. It is freely available as part of the Simtk project [40] in SIMM model format [41] that can be imported directly into OpenSim. The model is based on experimental data, includes 15 degrees of freedom and 50 muscle compartments and enables the evaluation of kinematics, muscle-tendon lengths, moment arms, muscle forces and joint moments in an anatomically reasonable setup. After importing, the structure of the model follows OpenSim’s convention including bodies connected with joints, rotational and translational kinematic constraints and forces defining muscle paths and attributes (for details, see Additional file 1). The 15 degrees of freedom define the kinematics of the shoulder (3), elbow (2), wrist (2), index finger (4) and thumb (4). As the current work focuses on kinematics of the shoulder, elbow and wrist joints only, any muscles and kinematics of the index finger and the thumb will not be taken into account further in this study. The seven degrees of freedom that define the kinematic state of the whole arm excluding the fingers are *elevation plane, thoracohumeral (elevation) angle* and *axial rotation* for the shoulder, *elbow flexion* and *forearm rotation* for the elbow and *deviation* and *flexion* for the wrist.

The model represents the upper limb as a linked kinematic chain of bodies, each having a parent body, a location in the parent’s frame and a joint describing the possible relative motion of the child with respect to the parent. The three-dimensional posture of the arm is generated by consecutive rotations of bodies determined by the actual angle values (joint coordinates) in proximal to distal order. As the movement of the shoulder girdle (clavicle, scapula and humerus) is complex and cannot be measured directly in most cases, the model implements regression equations that vary only with the *thoracohumeral angle* to determine the position of the shoulder joint with respect to the thorax.

#### Orientation from joint coordinates

**Fig. 1:**
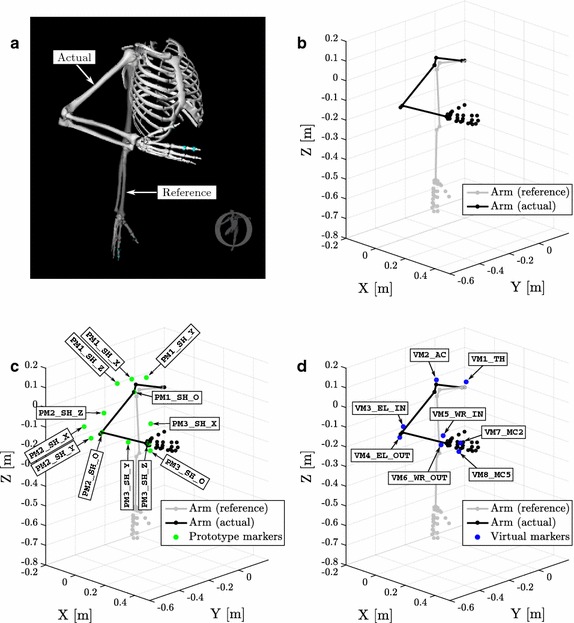
Representations of the used upper limb model with reference poses and markers. **a** Screenshot taken from OpenSim while displaying the used full arm model. The reference configuration is shown as a *shaded overlay* on an actual example configuration determined by the joint angle vector [$\theta _\mathtt{{elv}}$ = $0^\circ$, $\theta _\mathtt{{sh\_elv}}$ = $63^\circ$, $\theta _\mathtt{{sh\_rot}}$ = $15^\circ$, $\theta _\mathtt{{el\_flex}}$ = $95^\circ$, $\theta _\mathtt{{pro\_sup}}$ = $-60^\circ$, $\theta _\mathtt{{dev\_c}}$ = $0^\circ$, $\theta _\mathtt{{flex\_c}}$ = $20^\circ$]. **b** Representation of the model’s exported structure in MATLAB producing the same actual configuration as in sub-figure (**a**) using the developed forward kinematics function (functionally equivalent to OpenSim’s version). **c** Locations of prototype markers that are solely used to the reconstruction of model-defined anatomical joint angles with the proposed algorithm. **d** Locations of virtual markers that are used for the algorithm validation process by serving as inputs to OpenSim’s inverse kinematics tool directly

The reference orientation of the model (all joint coordinate values equal 0°) occurs when all of the following conditions are true [26] (for a visual reference, see Fig.  1a):

1. The shaft of the humerus is parallel to the vertical axis of the thorax.
2. In case of shoulder elevation, the humerus moves in the plane of shoulder abduction.
3. In case of elbow flexion, the forearm moves in the sagittal plane.
4. The hand is in the sagittal plane.
5. The third metacarpal bone in aligned with the long axis of the forearm.

During the calculation of arm orientation determined by the actual joint coordinates the position of the shoulder joint is calculated first from the *thoracohumeral angle*. This is followed by four consecutive rotations in the shoulder joint in the order of *elevation plane*, *elevation angle*, *elevation plane* and *axial rotation*, where the rotation axes of *elevation plane* and *axial rotation* overlap in the reference arm orientation. *Elbow flexion* occurs in the humeroulnar joint while *forearm rotation* takes place in the radioulnar joint. Motion of the wrist is distributed among the proximal and distal rows of carpal bones by having two rotations for each row (four in total) both depending on *flexion* and *deviation* values.

#### Markers, scaling and inverse kinematics

To evaluate subject motion with OpenSim, model parameters have to be adjusted to experimental data. As of OpenSim’s latest version at the time of writing (version 3.3), this can be achieved by using marker based motion capture data and virtual markers located on the model at approximately the same places as the experimental markers are located on the subject. This setup allows automatic subject specific scaling of the model [42] and calculation of anatomical joint coordinates (inverse kinematics) during the measured movement using weighted least squares optimization [43]. In the inverse kinematics tool, individual marker weights can be user specified and the least squares problem is solved with a quadratic programming solver (convergence criterion: 0.0001, iteration limit: 1000). As the efficiency of both scaling and inverse kinematics is highly dependent on the accuracy of virtual marker locations, marker placement is usually an iterative process until the best fit to experimental data is found.

### Prototype markers

To enable the utilization of the upper limb model with inertial measurements, a prototype marker set was defined (see Additional file 2). For this purpose, orthonormal bases were formed for each anatomical joint (shoulder, elbow and wrist) and markers were placed at specific locations in these bases to reflect the actual compound rotations among the respective degrees of freedom (for the corresponding mathematical definitions, see Appendix 1.

#### Orthonormal bases

*Shoulder* The three independent model axes for the shoulder (defined in model bodies *humphant*, *humphant1* and *humerus* that have the same position), collectively denoted as $\mathbf {B}_{sh\_orig}$, were good candidates to form a basis because they are unit length vectors (like all axes in the model) and almost orthogonal to each other (pairwise deviations from right angle are 0.00064°, 0.0029° and 0.0002°). For proper operation of the proposed algorithm however, these axes were orthogonalized using QR decomposition (see Appendix 2) to prevent error accumulation during the calculations. This resulted in the orthonormal basis $\mathbf {B}_{sh\_orth}$.

As a result, rotations in the shoulder can be expressed as elemental rotations of $\mathbf {B}_{sh\_orth}$ with acceptable angle errors due to the pairwise deviations between the original and new basis vectors after orthogonalization (0.000019°, 0.000655° and 0.002925°, respectively).

*Elbow* As relative orientation of the two rotation axes in the elbow is not close enough to orthogonal and the axes are defined in different parent bodies ($\mathbf {r}_\mathtt{{el\_flex}}$ $\rightarrow$ *ulna* and $\mathbf {r}_\mathtt{{pro\_sup}}$ $\rightarrow$ *radius*), the orthonormal basis $\mathbf {B}_\mathtt{{pro\_sup}}$ and the rotation matrix $\mathbf {R}_{{\mathbf {r}_\mathtt{{el\_flex}}}}^{{\mathbf {B}_\mathtt{{pro\_sup}}}}({\theta _\mathtt{{el\_flex}}})$ were formed to properly express the compound rotation as the product of an axis-angle and an elementary rotation about the main axis in $\mathbf {B}_\mathtt{{pro\_sup}}$. Again, some angle errors are expected while calculating the elbow flexion angle in this basis because $\mathbf {r}_\mathtt{{el\_flex}}$ is threated as it would belong to the *radius* body of the model.

*Wrist* Rotations in the wrist are the most complex among the three anatomical joints. Effects of the two active joint coordinates (*deviation* and *flexion*) are distributed among two model bodies (*lunate* and *capitate)*, each having two nonorthogonal rotations ($\mathbf {r}_\mathtt{{dev}}$, $\mathbf {r}_\mathtt{{flex}}$ $\rightarrow$ *lunate* and $\mathbf {r}_\mathtt{{pdr1}}$, $\mathbf {r}_\mathtt{{pdr3}}$ $\rightarrow$ *capitate*) depending on both joint coordinates. To deal with the complexity of this structure, the orthonormal basis $\mathbf {B}_\mathtt{{pdr3}}$ and rotation axes $\mathbf {r}_\mathtt{{dev}}^{{\mathbf {B}_\mathtt{{pdr3}}}}$, $\mathbf {r}_\mathtt{{flex}}^{{\mathbf {B}_\mathtt{{pdr3}}}}$ and $\mathbf {r}_\mathtt{{pdr1}}^{{\mathbf {B}_\mathtt{{pdr3}}}}$ were constructed to prepare the calculation of $\theta _\mathtt{{dev}}$ and $\theta _\mathtt{{flex}}$. Using this approach, $\mathbf {r}_\mathtt{{dev}}$ and $\mathbf {r}_\mathtt{{flex}}$ are threated as if they would belong to the *capitate* body of the model.

#### Marker placement

In order to add virtual markers to any OpenSim model, the parent body and the location within the parent’s frame have to be defined for each marker. Having the orthonormal bases from the previous section ($\mathbf {B}_{sh\_orth}$, $\mathbf {B}_\mathtt{{pro\_sup}}$ and $\mathbf {B}_\mathtt{{pdr3}}$), 12 prototype markers were placed on the model as follows (for reference, see Fig. 1c):

- Four markers were placed into each orthonormal basis having one at the origin of the actual basis ([0 0 0] in its parent body) and one in each axis of the basis.
- The markers were named using the convention PMx_[SH|EL|WR]_[O|X|Y|Z] where PM refers to *prototype marker*, x is the serial number of the basis in which the marker is located (1–3), [SH|EL|WR] refers to the anatomical joint in which the marker is located and [O|X|Y|Z] refers to the marker’s location within the actual basis (origin or any of the axes). For example the name of the wrist’s origin marker is PM3_WR_O.

Because all markers follow their parent bodies’ orientation during analyzed movements, coordinates of the difference vectors between the origin markers and the same basis’ axis markers reflect the compound rotation matrix in each anatomical joint (in the corresponding basis) at any time instant. To utilize this feature it is crucial that the structure of each joint’s marker subset remains consistent during measurements (by keeping the formation of an orthonormal basis), because any deviation in relative marker positions renders the derived compound rotation matrix inaccurate. As a consequence, it is recommended to use arm segment *orientations* to calculate the actual positions of prototype markers instead of measuring them directly. This can be achieved when using optical motion capture devices as segment orientations can be reconstructed with most systems by having at least three markers on each segment, however this is still an offline process. More importantly, utilizing orientation information makes the application of inertial sensors possible and beneficial in this setup as they are used to determine orientation directly. As an additional benefit, the offset-independent nature of orientation information enables subject-independent joint angle reconstruction, rendering the *scaling* step of the standard inverse kinematics approach unnecessary in the process. Using this feature a real-time inverse kinematics algorithm is introduced in the next section that provides joint coordinate outputs coherent with OpenSim’s inverse kinematics tool.

### Algorithm description

The key point in accelerating the selected upper limb model’s inverse kinematics calculation is the model specific determination of prototype marker locations. By constructing representative orthonormal bases in each anatomical joint of interest ($\mathbf {B}_{sh\_orth}$ in the shoulder, $\mathbf {B}_\mathtt{{pro\_sup}}$ in the elbow and $\mathbf {B}_\mathtt{{pdr3}}$ in the wrist) joint specific rotations can be addressed as elementary or axis-angle rotations in the corresponding bases. Having prototype markers in locations that reflect the actual orientations of these bases gives the possibility to express joint coordinates (rotation angles) in an efficient way, even in closed algebraic form in the shoulder and the elbow. As there was no closed form solution found to calculate angles in the wrist, a numerical algorithm is given for this part of the problem. MATLAB R2015b (Mathworks, Natick, MA, USA) was used for algorithm prototyping and development.

**Fig. 2:**
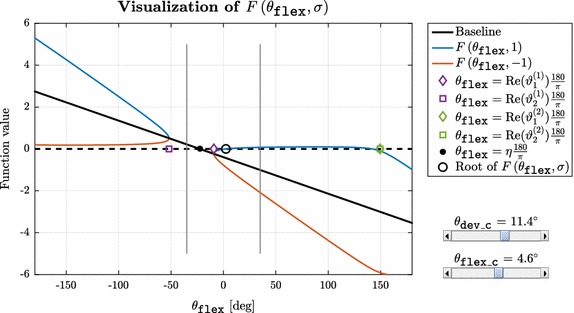
Representative screenshot of the tool developed for visual inspection of $F \left( {\theta _\mathtt{{flex}}}, \sigma \right)$ [defined in (6)]. The interactive MATLAB script allows simulation of different user-defined wrist configurations through separate sliders for $\theta _\mathtt{{dev\_c}}$ and $\theta _\mathtt{{flex\_c}}$ while plotting all relevant information about the optimization problem. The two *thinner vertical black lines* located at $\pm 35 ^\circ$ indicate model-defined limits for $\theta _\mathtt{{flex}}$

#### Shoulder

Because shoulder prototype markers are placed on the model in a way that they show the actual orientations of the main axes of $\mathbf {B}_{sh\_orth}$, an experimental (numerical) compound rotation matrix can be constructed from their spatial coordinates as shown in (1), where each marker position should be considered as a row vector.

1 $$\begin{aligned} {\widetilde{\mathbf {R}}^{shoulder}_{}} = \left( \begin{bmatrix} \texttt {PM1\_SH\_X} - \texttt {PM1\_SH\_O} \\ \texttt {PM1\_SH\_Y} - \texttt {PM1\_SH\_O} \\ \texttt {PM1\_SH\_Z} - \texttt {PM1\_SH\_O} \end{bmatrix}~ \mathbf {B}_{sh\_orth} \right) ^T \end{aligned}$$

By utilizing the kinematic structure of the shoulder joint (and keeping the assumption that $\widetilde{\mathbf {R}}^{shoulder}_{} = \mathbf {R}^{shoulder}$ as detailed in Appendix 3), estimations of rotation angle values can be calculated as follows:

2a $$\begin{aligned} {\widetilde{\theta }_\mathtt{{sh\_elv}}}&= \arccos \left( {\widetilde{\mathbf {R}}^{shoulder}_{(2,2)}} \right) \end{aligned}$$

2b $$\begin{aligned} {\widetilde{\theta }_\mathtt{{elv}}}&= \text {atan2} \left( {\widetilde{\mathbf {R}}^{shoulder}_{(3,2)}}, -{\widetilde{\mathbf {R}}^{shoulder}_{(1,2)}} \right) \end{aligned}$$

2c $$\begin{aligned} {\widetilde{\theta }_\mathtt{{sh\_rot}}}&= \arcsin \left( \frac{A {\widetilde{\mathbf {R}}^{shoulder}_{(2,1)}} + B {\widetilde{\mathbf {R}}^{shoulder}_{(2,3)}}}{A^2 + B^2} \right) \end{aligned}$$

$$\text {where } A = \sin ({\widetilde{\theta }_\mathtt{{elv}}}) \sin ({\widetilde{\theta }_\mathtt{{sh\_elv}}}) \quad \text{and} \quad B = \cos ({\widetilde{\theta }_\mathtt{{elv}}}) \sin ({\widetilde{\theta }_\mathtt{{sh\_elv}}}) $$

Although the formulations in (2a) and (2c) could be susceptible to modulo $\pi$ and sign errors in general, the allowed angle ranges defined in the model ($\theta _\mathtt{{sh\_elv}}$: $0^\circ \rightarrow 180^\circ$, $\theta _\mathtt{{sh\_rot}}$: $-90^\circ \rightarrow 20^\circ$) keep these equations safe to use until the experimental data does not force the model outside of these ranges.

#### Elbow

Similarly to the shoulder, the experimental compound rotation matrix can be constructed from the actual spatial positions of the elbow’s prototype markers. Because the model implements rotations in an incremental way, a reverse rotation of the extracted frame have to be performed in the shoulder’s basis to get the correct experimental rotation matrix for the elbow as shown in (3).

3 $$\begin{aligned} {\widetilde{\mathbf {R}}^{elbow}_{}} = \left( \begin{bmatrix} \mathtt {PM2\_EL\_X} - \mathtt {PM2\_EL\_O} \\ \mathtt {PM2\_EL\_Y} - \mathtt {PM2\_EL\_O} \\ \mathtt {PM2\_EL\_Z} - \mathtt {PM2\_EL\_O} \end{bmatrix}~ \left( \mathbf {B}_{sh\_orth}~{\widetilde{\mathbf {R}}^{shoulder}_{}}~\mathbf {B}_{sh\_orth}^T \right) ~ {\mathbf {B}_\mathtt{{pro\_sup}}} \right) ^T \end{aligned}$$

Having $\widetilde{\mathbf {R}}^{elbow}_{} = \mathbf {R}^{elbow}$ estimations of joint angle values in the elbow can be calculated as (for further details, see Appendix 5):

4a $$\begin{aligned} {\widetilde{\theta }_\mathtt{{el\_flex}}}&= \arccos \left( \frac{{\widetilde{\mathbf {R}}^{elbow}_{(1,1)}} - x^2}{1 - x^2} \right) \end{aligned}$$

4b $$\begin{aligned} {\widetilde{\theta }_\mathtt{{pro\_sup}}}&= \arcsin \left( \frac{A {\widetilde{\mathbf {R}}^{elbow}_{(1,2)}} + B {\widetilde{\mathbf {R}}^{elbow}_{(1,3)}}}{A^2 + B^2} \right) \end{aligned}$$

where $$ {\mathbf {r}_\mathtt{{el\_flex}}^{{\mathbf {B}_\mathtt{{pro\_sup}}}}} =  [x~y~z]$$

$$\begin{aligned} A & = y \sin \left( {\widetilde{\theta }_\mathtt{{el\_flex}}}\right) - x z \left( \cos ({\widetilde{\theta }_\mathtt{{el\_flex}}}) - 1 \right) \\ B &=  z \sin \left( {\widetilde{\theta }_\mathtt{{el\_flex}}}\right) + x y \left( \cos ({\widetilde{\theta }_\mathtt{{el\_flex}}}) - 1 \right)\end{aligned}$$

As in the case of the shoulder, (4a) and (4b) should be used with care because of modulo $\pi$ and sign errors, but again having sufficient joint angle limits in the model ($\theta _\mathtt{{el\_flex}}$: $0^\circ \rightarrow 130^\circ$, $\theta _\mathtt{{pro\_sup}}$: $-90^\circ \rightarrow 90^\circ$) application of these formulas is safe until experimental data does not force the model outside of these ranges.

#### Wrist

The experimental compound rotation matrix for the wrist can be constructed from the actual spatial positions of its prototype markers. Because of incremental rotations in the model, reverse rotations of the extracted frame have to be performed in the shoulder’s and elbow’s bases to get the correct experimental rotation matrix as shown in (5).

5 $$\begin{aligned}
{\widetilde{\mathbf {R}}^{wrist}_{}} &= \left( \begin{bmatrix} \texttt {PM3\_WR\_X} - \texttt {PM3\_WR\_O} \\
\texttt {PM3\_WR\_Y} - \texttt {PM3\_WR\_O}
\\ \texttt {PM3\_WR\_Z} - \texttt {PM3\_WR\_O} \end{bmatrix} \left( \mathbf {B}_{sh\_orth}~{\widetilde{\mathbf {R}}^{shoulder}_{}}~\mathbf {B}_{sh\_orth}^T \right) \right.
\\ & \qquad \left.\cdot \left( {\mathbf {B}_\mathtt{{pro\_sup}}}~{\widetilde{\mathbf {R}}^{elbow}_{}}~{\mathbf {B}_\mathtt{{pro\_sup}}}^T \right) {\mathbf {B}_\mathtt{{pdr3}}} {\vphantom {\begin{bmatrix} \texttt {PM3\_WR\_X} - \texttt {PM3\_WR\_O} \\ \texttt {PM3\_WR\_Y} - \texttt {PM3\_WR\_O} \\ \texttt {PM3\_WR\_Z} - \texttt {PM3\_WR\_O} \end{bmatrix}}}\right)^T \end{aligned}$$

Although there is no closed form solution to calculate joint angle rotations in the wrist, the flexion angle can be determined as the solution of the following root finding problem (further details and definitions of *a*, *b*, *c*, *x*, *y* and *z* can be found in Appendix 7):

6 $$\begin{aligned} \begin{array}{l} \text {Given } F \left( {\theta _\mathtt{{flex}}}, \sigma \right) = - {\theta _\mathtt{{flex}}} + \eta + \sigma ~ \text {atan2} \left( \text {Re} \left( \sqrt{\xi - c^2} \right) ,~c \right) , \\ \text {where} \\ \begin{array}{lrll} &{} \quad \eta &{} = &{} \text {atan2}(b,a) \\ &{} \sigma &{} \in &{} \{-1,1\} \\ &{} \xi &{} = &{} a^2 + b^2 \\ \end{array} \\ {\text {find}\;\theta _\mathtt{{flex}}} = \mu \text { such that } F \left( \mu , \sigma \right) = 0. \end{array} \end{aligned}$$

Based on this definition, the following properties hold for $F \left( {\theta _\mathtt{{flex}}}, \sigma \right)$:

1. $\left( - {\theta _\mathtt{{flex}}} + \eta \right)$ defines a baseline with constant negative slope for the two possible solutions $F \left( {\theta _\mathtt{{flex}}}, -1 \right)$ and $F \left( {\theta _\mathtt{{flex}}}, 1 \right)$.
2. Because of the definition of the *atan2(y,x)* function, the value of $\text {atan2} \left( \sqrt{\xi - c^2},~c \right)$ will always be positive if $\sqrt{\xi - c^2}$ is real (i.e. $c^2 \le \xi$). This implies that the two solutions to $F \left( {\theta _\mathtt{{flex}}}, \sigma \right)$ do not cross the baseline but remain “below” ($\sigma = -1$) and “above” ($\sigma = 1$) of it for all values of $\theta _\mathtt{{flex}}$.

As *c* depends on the actual compound rotation matrix $\widetilde{\mathbf {R}}^{wrist}_{}$, its value is influenced by both $\theta _\mathtt{{dev\_c}}$ and $\theta _\mathtt{{flex\_c}}$. As a consequence, there may be wrist configurations where $c^2 > \xi$ for some regions of $\theta _\mathtt{{flex}}$, driving $F \left( {\theta _\mathtt{{flex}}}, \sigma \right)$ into a singular state within these regions. To prevent problems arising from this situation during the root finding process, singularity border points for $\theta _\mathtt{{flex}}$ can be determined as follows. Let (7) as defined in (28), only $\theta _\mathtt{{flex}}$ changed to $\vartheta$ to denote specific singularity border points.

7 $$\begin{aligned} c = x \cos (\vartheta ) + y \sin (\vartheta ) + z \end{aligned}$$

**Fig. 3:**
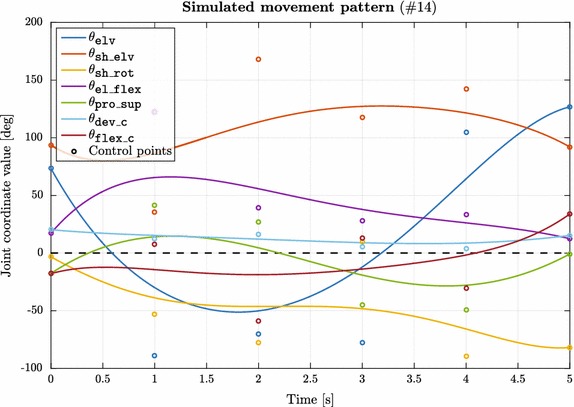
Representative simulated movement pattern used for algorithm validation. Simulated movement patterns were generated to validate the proposed kinematic algorithm. 100 separate pseudo-random joint coordinate trajectories were constructed as 5th order Bézier-curves having 5 s duration and 100 Hz sampling frequency. PMx and VMx marker trajectories were calculated with our forward kinematics MATLAB function to generate simulated “measurement” data for the proposed algorithm and OpenSim

Considering (6), singularity borders occur at locations where $c^2 = \xi$, resulting in $c_{1,2} = \pm \sqrt{\xi }$. Using these equalities and Euler’s formula, *c* can be rewritten into an exponential form that can be solved for $\vartheta$ resulting in the formulas shown below.

8 $$\begin{aligned} c_1 = &\sqrt{\xi }{:}\\  & \vartheta ^{(1)}_{1,2} =  -\ln \left( \dfrac{\sqrt{\xi } - z \pm \sqrt{\xi - 2 z \sqrt{\xi } - x^2 - y^2 + z^2}}{x - \mathrm {i}y} \right) \mathrm {i} \end{aligned}$$

9 $$\begin{aligned} c_2 = -&\sqrt{\xi }{:} \\ &\vartheta ^{(2)}_{1,2}  =  -\ln \left( - \dfrac{\sqrt{\xi } + z \pm \sqrt{\xi + 2 z \sqrt{\xi } - x^2 - y^2 + z^2}}{x - \mathrm {i}y} \right) \mathrm {i} \end{aligned}$$

As a result, four separate complex-valued singularity border points can be determined for all wrist configurations. To get a better understanding of the structure of $F \left( {\theta _\mathtt{{flex}}}, \sigma \right)$, the function was visually inspected with an interactive MATLAB script developed for this purpose. The tool allows the simulation of different user-defined wrist configurations through separate sliders for $\theta _\mathtt{{dev\_c}}$ and $\theta _\mathtt{{flex\_c}}$ while plotting all relevant information about the problem (a representative screenshot is shown in Fig. 2). Based on manual testing throughout the model-defined ranges for $\theta _\mathtt{{dev\_c}}$ and $\theta _\mathtt{{flex\_c}}$, the following observations were made:

1. The condition in (23) is always met.
2. $\vartheta ^{(k)}_{1,2} ~ ((k = 1) \vee (k = 2))$ are separate real numbers if there is a singularity region in the actual wrist configuration for $c_k$. In this case ${\theta _\mathtt{{flex}}} = \vartheta ^{(k)}_{1}$ and ${\theta _\mathtt{{flex}}} = \vartheta ^{(k)}_{2}$ indicate singularity border locations directly.
3. $\vartheta ^{(k)}_{1,2} ~ ((k = 1) \vee (k = 2))$ are complex conjugates if there is no singularity region in the actual wrist configuration for $c_k$. In this case ${\theta _\mathtt{{flex}}} = \text {Re} \left( \vartheta ^{(k)}_{1} \right) = \text {Re} \left( \vartheta ^{(k)}_{2} \right)$ indicates the location where the values of $F \left( {\theta _\mathtt{{flex}}}, -1 \right)$ and $F \left( {\theta _\mathtt{{flex}}}, 1 \right)$ are closest to ($k = 1$) and furthest from ($k = 2$) each other.
4. $\text {Re} \left( \vartheta ^{(2)}_{1,2} \right)$ always remain outside the model defined range of $\theta _\mathtt{{flex}}$.
5. ${\theta _\mathtt{{flex}}} = \eta$ is the “gluing point” of $F \left( {\theta _\mathtt{{flex}}}, -1 \right)$ and $F \left( {\theta _\mathtt{{flex}}}, 1 \right)$, meaning that the singularity region for $c_1$ starts to develop from this location, driving $F \left( {\theta _\mathtt{{flex}}}, \sigma \right)$ to “stick” to the baseline.
6. If there is a singularity region for $c_1$, $\text {Re} \left( \vartheta ^{(1)}_{1} \right)$ remains always smaller than $\mu$ where $F \left( \mu , \sigma \right) = 0$.
7. In cases when the singularity region starts to develop (i.e. $\left| \text {Im} \left( \vartheta ^{(k)}_{1} \right) \right|$ is sufficiently small but not zero), two separate roots may appear, but only one being valid.
8. $F \left( {\theta _\mathtt{{flex}}}, \sigma \right)$ will have a valid root at ${\theta _\mathtt{{flex}}} = \mu$ if and only if $\sigma = \text {sign} \left( \mu - \eta \right)$.

Based on these observations, (6) can be solved with the following algorithm: 
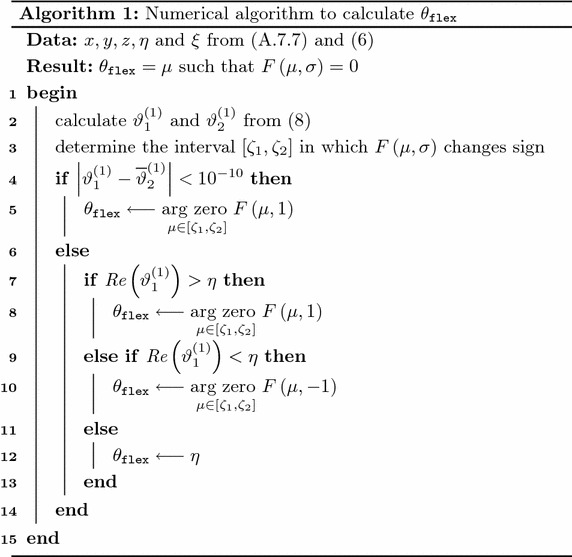


Having the value of $\theta _\mathtt{{flex}}$, $\theta _\mathtt{{flex\_c}}$ and $\theta _\mathtt{{dev\_c}}$ can be calculated as follows:

10a $$\begin{aligned} {\widetilde{\theta }_\mathtt{{flex\_c}}}&= 2*{\theta _\mathtt{{flex}}} \end{aligned}$$

10b $$\begin{aligned} {\widetilde{\theta }_\mathtt{{dev\_c}}}&= \text {atan2} \left( \mathbf {w}_1^T \mathbf {r}_1 \times \mathbf {v}_1, \mathbf {v}_1^T \mathbf {w}_1 - \left( \mathbf {v}_1^T \mathbf {r}_1 \right) \left( \mathbf {w}_1^T \mathbf {r}_1 \right) \right) \end{aligned}$$

where $$\mathbf {v}_1 = \text {exp} \left( {\theta _\mathtt{{flex}}} \widehat{\mathbf {r}}_{\texttt {flex}}^{{\mathbf {B}_\mathtt{{pdr3}}}} \right) {\mathbf {r}_\mathtt{{pdr1}}^{{\mathbf {B}_\mathtt{{pdr3}}}}}$$ and $$\mathbf {w}_1 = \left( {\widetilde{\mathbf {R}}^{wrist}_{}} ~ \text {exp} \left( -{\theta _\mathtt{{flex}}} \widehat{[1~0~0]} \right) \right) \mathbf {r}_{\texttt {flex}}^{{\mathbf {B}_\mathtt{{pdr3}}}}$$.

Although the computational demand of wrist angle calculations is higher than of the shoulder and the elbow, the algorithm has still higher overall time efficiency than the optimization approach used by OpenSim’s inverse kinematics tool, as it is shown in the "Results" section.

### Algorithm validation

Testing and validation of the described algorithm was automated using OpenSim with its Python API and MATLAB. To make direct comparison possible between OpenSim’s optimization method and the proposed algorithm, eight additional virtual markers were placed on the model at locations that are suitable for optical motion capture (e.g. using Vicon) simulating an environment where OpenSim is generally applied. The virtual marker locations are the following (for visual reference, see Fig. 1d):

- VM1_TH : Thorax marker at the upper end of the sternum.
- VM2_AC : Acromio-clavicular joint of the shoulder girdle.
- VM3_EL_IN : Medial epicondyle of the humerus.
- VM4_EL_OUT : Lateral epicondyle of the humerus.
- VM5_WR_IN : Distal head of the radius.
- VM6_WR_OUT : Distal head of the ulna.
- VM7_MC2 : Distal head of the second metacarpal bone.
- VM8_MC5 : Distal head of the fifth metacarpal bone.

The structure of the upper limb model (including marker positions) was extracted using OpenSim’s Python API and saved into a *.mat* file for further processing with MATLAB. A forward kinematics function (functionally equivalent to OpenSim’s implementation) was developed in MATLAB to calculate body and marker positions for specific joint coordinate vectors of [$\theta _\mathtt{{elv}}$, $\theta _\mathtt{{sh\_elv}}$, $\theta _\mathtt{{sh\_rot}}$, $\theta _\mathtt{{el\_flex}}$, $\theta _\mathtt{{pro\_sup}}$, $\theta _\mathtt{{dev\_c}}$, $\theta _\mathtt{{flex\_c}}$] in the model, enabling the analysis of trajectories for both PMx and VMx markers from artificially generated movement patterns (Fig. 1b–d).

#### Simulated movement patterns

To avoid possible problems accompanying experimental measurements, simulated movement patterns were generated to test the performance and validity of the proposed algorithm. 100 separate pseudo-random (random seed = 10) joint coordinate trajectories were constructed in MATLAB having a duration of 5 s and a sampling frequency of 100 Hz. The trajectories were generated as 5th order Bézier-curves as shown in (11) using six uniformly distributed control points (0, 20,...,100%) with randomly chosen values for each joint coordinate from their valid intervals defined in the model. A representative movement pattern is shown in Fig. 3.

11 $$ \begin{aligned}  \mathbf {B}_{5}(t) & =   \sum _{i=0}^{5} \left( {\begin{array}{c}5\\ i\end{array}}\right) ~ t^{i} ~ (1-t)^{5-i} ~ \mathbf {P}_i  \\ &=  (1-t)^5\mathbf {P}_0 + 5t(1-t)^4\mathbf {P}_1 + 10t^2(1-t)^3\mathbf {P}_2  \\ &\quad+10t^3(1-t)^2\mathbf {P}_3 + 5t^4(1-t)\mathbf {P}_4 + t^5\mathbf {P}_5 \\ \text{where} \;\;& t \in [0,1] \;\;\text { and}\;\; \mathbf {P}_i, i \in \{0, \ldots , 5\} \text { are the control points.}\end{aligned} $$

Following this step, forward kinematics was performed for each of the simulated patterns to calculate PMx and VMx marker trajectories yielding simulated “measurement” data as it would have been recorded during a real movement. The resulted trajectories were then used as inputs to inverse kinematics calculations with OpenSim (VMx) and our algorithm (PMx) while the corresponding movement patterns served as reference for the outputs of each of the tested methods.

#### Inverse kinematics with OpenSim

To speed up the validation process, OpenSim (v3.3) was compiled from source on a Supermicro server having two Intel^®^ Xeon^®^ E5-2695 v3 CPUs (with a total of 56 execution threads) and 64 GB RAM, running Ubuntu Server 14.04.2 LTS operating system. Although the inverse kinematics (IK) algorithm in the used OpenSim version do not utilize multi-core architectures natively, each IK task can be divided into separate subtasks that can run in parallel thanks to the applied optimization method (there is no data dependency between time frames). To utilize this property, a pipeline was developed using MATLAB and Bash to prepare VMx marker data and the required files for OpenSim and manage file transfers, multi-threaded IK execution, results collection and evaluation. One important step before performing the IK calculation in OpenSim is subject-specific scaling of the used model and relative weighting of the markers. As only simulated data were used in the current study on an unmodified upper limb model, the scaled model file was identical to the original file during IK execution, while all marker weights were equal.

#### Algorithm implementation

The prototype of the proposed algorithm was implemented in MATLAB and tested with the simulated PMx marker trajectories. Calculation of (6) was performed using MATLAB’s built-in fzero() function. Based on the MATLAB version, the algorithm was implemented in ANSI C to target practical applications. In this case (6) was solved with Brent’s root finding algorithm from [44]. Furthermore, compilation options were included to assess the effects of different data precisions (float or double) on the accuracy and execution time of the algorithm. This was not an option with MATLAB because fzero() cannot be used with float input.

To address possible accuracy problems arising from the lower precision of float data, an additional test case with a simple output continuity check for wrist angles was included, namely when the absolute difference between two successive $\theta _\mathtt{{flex\_c}}$ values is larger than 5°, the actual $\theta _\mathtt{{flex\_c}}$ will be the previous $\theta _\mathtt{{flex\_c}}$ + 0.5°. This modified version of the algorithm is denoted with *mod.* suffix among the results.

#### Evaluation platforms

MATLAB and C implementations of the proposed algorithm were tested on a system with an Intel^®^ Core^®^ i5-540M processor running Ubuntu Desktop 14.04.4 LTS. In addition, the C implementation was evaluated on the following microcontroller units (MCUs) that are capable of targeting resource constrained environments (e.g. wearable measurement devices) with high performance:

1. STM32F407VG - ARM Cortex-M4 core with single precision floating point unit (FPU), up to 168 MHz core clock, 1 MB Flash memory, 192 KB SRAM.
2. STM32F746NG - ARM Cortex-M7 core with single precision FPU and L1-cache, up to 216 MHz core clock, 1 MB Flash memory, 320 KB SRAM.

For proper comparison, both MCUs were clocked at 168 MHz and the source codes differed only in device specific details (e.g. hardware initialization). Algorithm evaluation on the MCUs was controlled with MATLAB via a UART link including data preparation, transmission and storage.

#### Performance metrics

To evaluate the overall performance of the algorithm compared to OpenSim’s IK method, accuracy and execution times were analyzed in all cases (OpenSim, MATLAB and C implementations). To assess accuracy, RMS values were computed for the differences between the calculated and simulated joint coordinate trajectories for each trial. Means and standard deviations of these RMS values were then calculated across trials for each platform and precision (where this was applicable).

Running times of OpenSim’s IK evaluation were calculated as a sum of subtask execution times from the IK log output directly. Algorithm execution times were measured by the tic and toc methods in MATLAB, the clock() function from the $\texttt {<time.h>}$ library for the C implementation on PC and on-chip hardware timers clocked at 1 MHz for both MCUs.

#### Data exclusion from OpenSim trials

Although inverse kinematics in OpenSim was calculated using an unmodified and unscaled model in each trial, there were cases when large step errors occurred at seemingly random locations in the IK output (independently of subtask borders mentioned in "Inverse kinematics with OpenSim" section). This phenomenon may be handled by marker placement adjustment or error checking in measurement data in general. As IK input was strictly controlled by using simulated trajectories and the markers remained intact in the model between trials, further troubleshooting would have been needed to find a solution to this issue. Because the main emphasis of the study is the proposed algorithm and not OpenSim’s internal workings and IK troubleshooting, all OpenSim trials were excluded from final accuracy assessment where any of the resulted joint coordinate RMS errors exceeded 5° to not bias the results with incorrect data. As a result, only 59 trials out of 100 were used to calculate the accuracy of OpenSim’s IK algorithm. This however did not have any effect on the other measurements, so MATLAB and all C results were calculated across 100 trials.

## Results

### Accuracy

RMS errors from algorithm evaluation are shown in Table 1. The results show that considering the mean of all valid trials (59 for OpenSim, 100 for all others), all platforms performed reasonably well producing errors below 3° for all joint coordinates (for a trial-wise visual comparison between OpenSim’s IK method and the proposed algorithm, see Additional file 3).

**Table 1:** RMS errors

Test environment	$\theta _\mathtt{{elv}}$ (°)	$\theta _\mathtt{{sh\_elv}}$ (°)	$\theta _\mathtt{{sh\_rot}}$ (°)	$\theta _\mathtt{{el\_flex}}$ (°)	$\theta _\mathtt{{pro\_sup}}$ (°)	$\theta _\mathtt{{dev\_c}}$ (°)	$\theta _\mathtt{{flex\_c}}$ (°)
OpenSim	0.0429 ± 0.0339	0.0192 ± 0.0053	0.1472 ± 0.0760	0.0764 ± 0.0288	0.6365 ± 0.1701	0.9198 ± 0.2477	2.2916 ± 1.1142
MATLAB	0.0028 ± 0.0003	0.0006 ± 0.0002	0.0014 ± 0.0007	0.0005 ± 0.0002	0.0008 ± 0.0006	0.0023 ± 0.0041	0.0049 ± 0.0087
PC double	0.0028 ± 0.0003	0.0006 ± 0.0002	0.0014 ± 0.0007	0.0005 ± 0.0002	0.0008 ± 0.0006	0.0025 ± 0.0051	0.0053 ± 0.0107
PC float	0.0028 ± 0.0003	0.0006 ± 0.0002	0.0016 ± 0.0013	0.0005 ± 0.0002	0.0008 ± 0.0006	0.4193 ± 0.8995	1.1148 ± 2.4730
PC float mod.	0.0028 ± 0.0003	0.0006 ± 0.0002	0.0016 ± 0.0013	0.0005 ± 0.0002	0.0008 ± 0.0006	0.0045 ± 0.0092	0.0097 ± 0.0195
ARM M4 double	0.0028 ± 0.0003	0.0006 ± 0.0002	0.0014 ± 0.0007	0.0005 ± 0.0002	0.0008 ± 0.0006	0.0025 ± 0.0051	0.0053 ± 0.0107
ARM M4 soft float	0.0028 ± 0.0003	0.0006 ± 0.0002	0.0016 ± 0.0013	0.0005 ± 0.0002	0.0008 ± 0.0006	0.4193 ± 0.8995	1.1147 ± 2.4730
ARM M4 hard float	0.0028 ± 0.0003	0.0006 ± 0.0002	0.0016 ± 0.0013	0.0005 ± 0.0002	0.0008 ± 0.0006	0.4095 ± 0.9051	1.0944 ± 2.4840
ARM M4 soft float mod.	0.0028 ± 0.0003	0.0006 ± 0.0002	0.0016 ± 0.0013	0.0005 ± 0.0002	0.0008 ± 0.0006	0.0078 ± 0.0128	0.0170 ± 0.0276
ARM M4 hard float mod.	0.0028 ± 0.0003	0.0006 ± 0.0002	0.0016 ± 0.0013	0.0005 ± 0.0002	0.0008 ± 0.0006	0.0077 ± 0.0128	0.0167 ± 0.0275
ARM M7 double	0.0028 ± 0.0003	0.0006 ± 0.0002	0.0014 ± 0.0007	0.0005 ± 0.0002	0.0008 ± 0.0006	0.0025 ± 0.0051	0.0053 ± 0.0107
ARM M7 soft float	0.0028 ± 0.0003	0.0006 ± 0.0002	0.0016 ± 0.0013	0.0005 ± 0.0002	0.0008 ± 0.0006	0.4193 ± 0.8995	1.1147 ± 2.4730
ARM M7 hard float	0.0028 ± 0.0003	0.0006 ± 0.0002	0.0016 ± 0.0013	0.0005 ± 0.0002	0.0008 ± 0.0006	0.4095 ± 0.9051	1.0944 ± 2.4840
ARM M7 soft float mod.	0.0028 ± 0.0003	0.0006 ± 0.0002	0.0016 ± 0.0013	0.0005 ± 0.0002	0.0008 ± 0.0006	0.0078 ± 0.0128	0.0170 ± 0.0276
ARM M7 hard float mod.	0.0028 ± 0.0003	0.0006 ± 0.0002	0.0016 ± 0.0013	0.0005 ± 0.0002	0.0008 ± 0.0006	0.0077 ± 0.0128	0.0167 ± 0.0275

Each row represents a separate test environment for the reference (OpenSim) and proposed inverse kinematics algorithm. The columns show *mean ± standard deviation* joint angle RMS errors across all valid trials (59 for OpenSim, 100 otherwise) for each test environment

Regarding OpenSim it can be seen that errors for each joint coordinate are larger than those provided by our algorithm. The reason for this can lie in the optimization approach of OpenSim that in fact contains hard-coded convergence (0.0001) and iteration (1000) limits. However these limits prevent OpenSim’s IK algorithm to match the simulated movement patterns perfectly, they provide a practical solution to the $accuracy \leftrightarrow running~time$ trade-off for the software’s general usage.

MATLAB and C implementations of the proposed algorithm performed equally well for shoulder and elbow angles independent of the used data precision (double / float). This could occur because of the relatively low number of operations needed by these joint coordinates shown in equation groups () and () that prevented considerable error accumulation due to the lower precision of float. Regarding wrist angles a clear distinction can be made between double and float (MATLAB uses double as default). The two main reasons for this phenomenon are (1) the significantly larger computational demand of $\theta _\mathtt{{dev\_c}}$ and $\theta _\mathtt{{flex\_c}}$ involving iterative processes that can lead to precision error accumulations and (2) rounding error based mismatch in the root finding process involved in the calculation of $\theta _\mathtt{{flex}}$ in rare cases when two roots are present in (6). A deeper analysis among the trial-wise results revealed that the second reason was more significant as roughly 70% of the trials ended up in no more than 0.1° maximum error with float precision. The rest of the trials contained 1–5 “wrong” samples showing 15°–20° impulse-like errors while the remaining samples within the trial did not have this problem. Investigation of the erroneous samples revealed that indeed a wrong root for (6) was found in these cases. To deal with this issue, an output continuity checking step was implemented for float precision in cases denoted with the *mod.* suffix. This turned out to be a simple yet effective solution to the problem as the corresponding results show the disappearance of the impulse-like errors.

### Execution time

To assess overall performance, execution times were compared between OpenSim’s IK method and our proposed algorithm on different platforms and are shown in Table 2.

**Table 2:** Execution times

Test environment	Execution time per iteration (ms)	Speedup wrt. OpenSim
OpenSim	145.0532 ± 10.0669	1x
MATLAB	2.3656 ± 0.6689	61x
PC double	0.0111 ± 0.0013	13011x
PC float	0.0088 ± 0.0008	16416x
PC float mod.	0.0097 ± 0.0013	14982x
ARM M4 double	4.8777 ± 0.3554	30x
ARM M4 soft float	2.7327 ± 0.0928	53x
ARM M4 hard float	0.9713 ± 0.0214	149x
ARM M4 soft float mod.	2.7394 ± 0.0930	53x
ARM M4 hard float mod.	0.9740 ± 0.0216	149x
ARM M7 double	2.3124 ± 0.1704	63x
ARM M7 soft float	1.4293 ± 0.0504	101x
ARM M7 hard float	0.4462 ± 0.0117	325x
ARM M7 soft float mod.	1.4296 ± 0.0505	101x
ARM M7 hard float mod.	0.4478 ± 0.0115	324x

Each row represents a separate test environment for the reference (OpenSim) and proposed inverse kinematics algorithm. Table values show mean ± *standard deviation* for a single iteration across all valid trials (59 for OpenSim, 100 otherwise) and the speed increase of each tested setup with respect to OpenSim

Measurement results show that the optimization approach of OpenSim performed the calculation of a single iteration in 145 ms on average. Because of the application specific nature of the proposed algorithm, its running times considering different implementations (MATLAB/C), data precisions (double/float) and platforms (PC/ARM Cortex-M) all showed a significant increase in execution performance compared to OpenSim, the worst result being about 5 ms on average for a single iteration.

As expected, the C implementation is more than two orders of magnitude faster than the MATLAB version on the PC, yielding execution times per iteration about 10 μs with all precision variants (double, float and float mod.). Opposed to this, running times on embedded platforms showed more scattered results. The difference between double and float is more expressed in these cases while application of the FPU accelerates float computations even further (*hard float* entries in Table 2). Regarding the modified algorithm variant it can be seen that even the extra continuity check adds some amount to the execution time per iteration, the possibility to use float precision brings more speed advantage, especially with the FPU enabled. These findings are true for both tested MCUs with the observation that ARM’s M7 architecture is about twice as fast as M4 when running the presented algorithm with the same core clock.

## Discussion

Evaluation results of the tested algorithms show that each approach provides proper accuracy for most common arm movement analysis scenarios. One important aspect however is that while OpenSim provides a useful general tool for biomechanical analysis including fields beyond inverse kinematics (e.g. inverse and forward dynamics), the calculation of joint angles from the actual experimental data is rather demanding computationally. As the output of this step gives the basis for all other analysis methods in the software, the amount of time needed for the overall processing pipeline highly depends on the efficiency of this algorithm. As Table 2 shows, the average amount of time needed for OpenSim’s IK algorithm to perform a single iteration would allow about 7 Hz operation that falls behind the generally accepted practice in human movement recording of at least 50 Hz. This property excludes OpenSim from tight integration with systems requiring real-time movement kinematics, however that is not the software’s original target application anyway (up to version 3.3 at least).

Considering the algorithm proposed in the study Tables 1 and 2 show a significant improvement in performance in both accuracy and execution time when compared to OpenSim’s IK method. The main reason for this difference is the algorithm’s application specific nature with the utilization of both the internal structure of the used upper limb model and inertial sensing of movement to determine limb segment orientations directly. As the MATLAB version showed proper accuracy and sufficiently short execution time on the PC, implementation of the algorithm in ANSI C was reasonable to assess its “real” performance without the overhead of a general prototyping tool that MATLAB essentially has. Because accuracy results are the same or very similar across specific variants of the C implementation (i.e. using double/float precision), only execution time differences are discussed later in the text.

Running times of the algorithm’s C implementation showed more than four orders of magnitude speedup on the tested Intel^®^ Core^®^ i5-540M processor compared to OpenSim’s IK algorithm on a more recent and higher performance server CPU with Xeon^®^ architecture, yielding about 10 μs execution time per iteration for all variants. However this is an impressive improvement, running the algorithm on PC would still pose problems from practical aspects of possible applications (e.g. total size and mobility of the measurement system or communication overhead between the measuring and processing device), so the real benefit of this speed increase lies in the “spare” performance that opened the way to testing the algorithm in resource constrained embedded environments. Evaluation of the proposed method on high performance MCUs showed that all implementation variants that provided good accuracy (double and [soft/hard] float mod.) had acceptable execution times on both architectures (M4 and M7) for real-time operation, considering 100 Hz as sufficient sampling frequency for human movement analysis. Based on these results, the specific implementation variant should be chosen taking the overall design requirements of the actual practical application into account (i.e. wearable measurement devices like the one presented in [45]) as in most cases the algorithm should fit into a system containing other computationally demanding processes (like sensor fusion algorithms) with power consumption being a critical part of the design for example.

An other practical advantage of the described algorithm is that it enables subject-independent joint angle reconstruction during the measurements. This means that by taking advantage of the offset-independent nature of orientation sensing, no scaling is required for the proper calculation of inverse kinematics (opposed to OpenSim) as long as the IMUs are capable to produce good approximations of limb segment orientations.

It needs to be emphasized however that the application specific nature of the algorithm and its dependency on the used upper limb model induce some practical considerations, because having a method that works in a strictly controlled simulation environment does not guarantee its applicability in a real situation. A fundamental thing to consider is whether data provided by real sensors reflect arm segment orientations needed by the algorithm accurately. As this was a key requirement from the beginning of algorithm design, the prototype markers were defined in local bases of the joints that can be directly expressed in terms of sensor orientations (for details, see Additional files 1, 2). As an additional benefit, the definition of an anatomical calibration procedure—often needed when inertial sensors are used for human movement recording—can be avoided as the proposed algorithm does not use segment length information for joint angle reconstruction. What cannot be avoided however is the accurate estimate of sensor orientations, as the whole process depends highly on the precision of this step. Although there is no ultimate solution to the problem of inertial sensor fusion yet, there are continuous algorithmic efforts to reach higher accuracy and reliability (for different highlighted approaches, see "Background" section). But even in cases when the sensors provide accurate orientation information of the measured limb, care must be taken when determining the limb’s reference orientation based on the measurements. The reason for this is mainly inter-subject variability in the sense that even the model defines the reference posture clearly, it cannot be assumed that any actual subject will reproduce the same posture very accurately that can lead to offset errors during the measurement. Furthermore, the assumption was made during algorithm development that the measured movement always remains within the valid joint angle ranges defined in the model. As long as this assumption holds (as in the case of simulated movement patterns presented in this study), the algorithm should not have problems with proper joint angle reconstruction. However, if outliers are present in the experimental data (e.g. reference posture errors, inaccuracies in the measurement or the sensor fusion algorithm or extreme anatomical ranges of a subject) undefined output states can occur. This may be handled with a simple saturation technique on the algorithm level but rather should be prevented by applying proper experimental design and calibration methods. In a practical setup this involves proper sensor placement and various steps before the measurements including zero motion offset compensation, hard and soft iron error compensation in the magnetometer and determining relative sensor orientations with respect to the measured segments [46, 47] for example.

## Conclusions

With keeping the upper mentioned considerations in mind the proposed algorithm is capable for real-time reconstruction of standardized anatomical joint angles even in embedded environments, opening the way to complex applications requiring accurate and fast calculation of model-based movement kinematics. Although the presented algorithm is special to the selected upper limb model, the introduced approach by strategically placing the prototype markers can further be applied to other biomechanical models in the future. As a result, the proposed method brings the possibility to widen the application areas of OpenSim with complex models and making its overall analysis pipeline more efficient by accelerating the calculation of inverse kinematics and providing the possibility to perform this step even on the measurement device in cases when accurate inertial movement sensing is applicable.

## Appendices

### Appendix 1: Definitions

To facilitate algorithm description in the text, notations in Table 3 will be used to identify each degree of freedom and the rotation axes and angle values for them, where each rotation axis ($\mathbf {r}_\mathtt{{axis\_id}}$) should be considered as a row vector.

**Table 3:** Axis and angle notations

Anatomical joint	Degree of freedom	Identifier	Rotation axis	Rotation angle
Shoulder	Elevation plane	elv	$\mathbf {r}_\mathtt{{elv}}$	$\theta _\mathtt{{elv}}$
	Thoracohumeral (elevation) angle	sh_elv	$\mathbf {r}_\mathtt{{sh\_elv}}$	$\theta _\mathtt{{sh\_elv}}$
	Axial rotation	sh_rot	$\mathbf {r}_\mathtt{{sh\_rot}}$	$\theta _\mathtt{{sh\_rot}}$
Elbow	Elbow flexion	el_flex	$\mathbf {r}_\mathtt{{el\_flex}}$	$\theta _\mathtt{{el\_flex}}$
	Forearm rotation	pro_sup	$\mathbf {r}_\mathtt{{pro\_sup}}$	$\theta _\mathtt{{pro\_sup}}$
Wrist	Deviation	dev	$\mathbf {r}_\mathtt{{dev}}$	$\theta _\mathtt{{dev}}$
	Flexion	flex	$\mathbf {r}_\mathtt{{flex}}$	$\theta _\mathtt{{flex}}$
	Proximal-distal r1	pdr1	$\mathbf {r}_\mathtt{{pdr1}}$	$\theta _\mathtt{{pdr1}}$
	Proximal-distal r3	pdr3	$\mathbf {r}_\mathtt{{pdr3}}$	$\theta _\mathtt{{pdr3}}$

The table lists identifiers derived from the model file and notations of rotation axes and angles for all relevant degrees of freedom used for algorithm formulation

Furthermore, the following definitions are introduced:

1. An orthonormal basis with a selected rotation axis in its main axis will be noted as $\mathbf {B}_\mathtt{{axis\_id\_0}}$, where axis_id_0 is the identifier of the axis (e.g. for a basis with $\mathbf {r}_\mathtt{{elv}}$ in its main axis the basis will be $\mathbf {B}_\mathtt{{elv}}$). If the basis is not formed by orthogonalization of rotation vectors in the model (as in the case of the shoulder), $\mathbf {B}_\mathtt{{axis\_id\_0}}$ is defined as
  12a $$\begin{aligned} {\mathbf {B}_\mathtt{{axis\_id\_0}}} = \begin{bmatrix} {\mathbf {r}_\mathtt{{axis\_id\_0}}} \\ \mathbf {r}_2 \\ \mathbf {r}_3 \end{bmatrix}^T&\quad \text {where}\end{aligned}$$
  12b $$\begin{aligned} \mathbf {r}_2 = \frac{{\mathbf {r}_\mathtt{{axis\_id\_0}}} \times \left( {\mathbf {r}_\mathtt{{axis\_id\_0}}}-[1~0~0]\right) }{\left\| {\mathbf {r}_\mathtt{{axis\_id\_0}}} \times \left( {\mathbf {r}_\mathtt{{axis\_id\_0}}}-[1~0~0]\right) \right\| }&\quad \text {and} \end{aligned}$$
  12c $$\begin{aligned} \mathbf {r}_3 = \frac{{\mathbf {r}_\mathtt{{axis\_id\_0}}} \times \mathbf {r}_2}{\left\| {\mathbf {r}_\mathtt{{axis\_id\_0}}} \times \mathbf {r}_2\right\| } \end{aligned}$$
2. The rotation axis $\mathbf {r}_\mathtt{{axis\_id}}$ expressed in the basis $\mathbf {B}_\mathtt{{axis\_id\_0}}$ is defined as
  13 $$\begin{aligned} {\mathbf {r}_\mathtt{{axis\_id}}^{{\mathbf {B}_\mathtt{{axis\_id\_0}}}}} = {\mathbf {r}_\mathtt{{axis\_id}}} {\mathbf {B}_\mathtt{{axis\_id\_0}}} \end{aligned}$$
3. Given a unit length vector $\mathbf {r} \in \mathbf {R}^3$, Rodrigues’ formula gives the rotation matrix about $\mathbf {r}$ of an arbitrary angle $\theta \in [-\pi ,\pi [$ as
  14 $$\begin{aligned} \begin{array}{l} \text {exp} \left( \theta \widehat{\mathbf {r}} \right) = I_3 + \sin \left( \theta \right) \widehat{\mathbf {r}} + \left( 1 - \cos \left( \theta \right) \right) \widehat{\mathbf {r}}^2 \\ \\ \begin{array}{llll} \text {where } &{} \widehat{\mathbf {r}}: \mathbf {R}^3 \rightarrow \mathbf {R}^3 &{} \overset{def}{=} &{} \widehat{\mathbf {r}} ~ \mathbf {v} = \mathbf {r} \times \mathbf {v} \end{array} \end{array} \end{aligned}$$
4. The matrix representation of an axis-angle rotation formed from a unit axis $\mathbf {r}_\mathtt{{axis\_id}}$ and angle $\theta _\mathtt{{axis\_id}}$ expressed in the basis $\mathbf {B}_\mathtt{{axis\_id\_0}}$ is given as follows:
  15 $$\begin{aligned} {\mathbf {R}_{{\mathbf {r}_\mathtt{{axis\_id}}}}^{{\mathbf {B}_\mathtt{{axis\_id\_0}}}}({\theta _\mathtt{{axis\_id}}})} &= \text {exp} \left( {\theta _\mathtt{{axis\_id}}} ~ \widehat{\mathbf {r}}_{\texttt {axis\_id}}^{{\mathbf {B}_\mathtt{{axis\_id\_0}}}} \right)  \\ &\equiv \begin{bmatrix} xxC+c & xyC-zs & xzC+ys \\ yxC+zs & yyC+c &  yzC-xs \\ zxC-ys &  zyC+xs &  zzC+c \end{bmatrix}  \end{aligned}$$
  where $${\mathbf {r}_\mathtt{{axis\_id}}^{{\mathbf {B}_\mathtt{{axis\_id\_0}}}}}= [x~y~z] $$, $$s = \sin \left( {\theta _\mathtt{{axis\_id}}} \right)$$, $$c = \cos \left( {\theta _\mathtt{{axis\_id}}} \right)$$ and $$C = 1 - C.$$
5. The *i*th element of vector $\mathbf {v}$ is denoted as $\mathbf {v}_{(i)}$. Similarly, the (*i*, *j*)th element of matrix $\mathbf {R}$ is denoted as $\mathbf {R}_{(i,j)}$, where *i* is the row index and *j* is the column index. Indexing complete rows and columns is denoted as $\mathbf {R}_{(i,:)}$ and $\mathbf {R}_{(:,j)}$, respectively.

### Appendix 2: QR orthogonalization

The general formula of QR orthogonalization is shown below, where $\mathbf {A}$ is a regular matrix, $\mathbf {Q}$ is an orthogonal matrix, $\mathbf {R}$ is an upper triangular matrix, $\mathbf {S}$ is the sign diagonal matrix of $\mathbf {R}$ and $\mathbf {B}$ is the resulting orthogonal matrix.

16 $$\begin{aligned} \mathbf {A} = \mathbf {QR} \end{aligned}$$

17 $$\begin{aligned} \mathbf {S} = {\left\{ \begin{array}{ll} s_{ii} = 1 &{} \quad \text {if } r_{ii} > 0 \\ s_{ii} = -1 &{} \quad \text {if } r_{ii} < 0 \\ s_{ij} = 0 &{} \quad \text {if } i \ne j \end{array}\right. } \end{aligned}$$

18 $$\begin{aligned} \mathbf {B} = \mathbf {QS} \end{aligned}$$

### Appendix 3: Auxiliary calculations for the shoulder

The orientation of the humerus is determined by four consecutive rotations in the shoulder in the order of *elevation plane*, *elevation angle*, *-elevation plane* and *axial rotation* degrees of freedom (for axis and angle notations, see Table 3). Based on axis definitions in the model, rotations about $\mathbf {r}_\mathtt{{elv}}$ and $\mathbf {r}_\mathtt{{sh\_rot}}$ can be estimated with an elementary rotation about the second axis of $\mathbf {B}_{sh\_orth}$ while the estimation of the rotation about $\mathbf {r}_\mathtt{{sh\_elv}}$ can be done with an elementary rotation about $\mathbf {B}_{sh\_orth}$’s third axis as shown in ().

19a $$\begin{aligned} \begin{array}{lll} {\mathbf {R}_{{\mathbf {r}_\mathtt{{elv}}}}^{\mathbf {B}_{sh\_orth}}({\theta _\mathtt{{elv}}})} &{} = &{} \begin{bmatrix} \cos ({\theta _\mathtt{{elv}}}) &{} 0 &{} \sin ({\theta _\mathtt{{elv}}}) \\ 0 &{} 1 &{} 0 \\ -\sin ({\theta _\mathtt{{elv}}}) &{} 0 &{} \cos ({\theta _\mathtt{{elv}}}) \end{bmatrix} \end{array}\end{aligned}$$

19b $$\begin{aligned} \begin{array}{lll} {\mathbf {R}_{{\mathbf {r}_\mathtt{{sh\_rot}}}}^{\mathbf {B}_{sh\_orth}}({\theta _\mathtt{{sh\_rot}}})} &{} = &{} \begin{bmatrix} \cos ({\theta _\mathtt{{sh\_rot}}}) &{} 0 &{} \sin ({\theta _\mathtt{{sh\_rot}}}) \\ 0 &{} 1 &{} 0 \\ -\sin ({\theta _\mathtt{{sh\_rot}}}) &{} 0 &{} \cos ({\theta _\mathtt{{sh\_rot}}}) \end{bmatrix} \end{array} \end{aligned}$$

19c $$\begin{aligned} \begin{array}{lll} {\mathbf {R}_{{\mathbf {r}_\mathtt{{sh\_elv}}}}^{\mathbf {B}_{sh\_orth}}({\theta _\mathtt{{sh\_elv}}})} &{} = &{} \begin{bmatrix} \cos ({\theta _\mathtt{{sh\_rot}}}) &{} -\sin ({\theta _\mathtt{{sh\_elv}}}) &{} 0 \\ \sin ({\theta _\mathtt{{sh\_elv}}}) &{} \cos ({\theta _\mathtt{{sh\_rot}}}) &{} 0 \\ 0 &{} 0 &{} 1 \end{bmatrix} \end{array} \end{aligned}$$

Using these definitions, the symbolic expression of the compound rotation matrix that represents the actual orientation in the shoulder can be calculated as shown in (20). For the convenience of calculation, this step was performed with MATLAB’s Symbolic Math Toolbox using the script shown in Appendix 4.

20 $$\begin{aligned}
{\mathbf {R}^{shoulder}} &= {\mathbf {R}_{{\mathbf
{r}_\mathtt{{elv}}}}^{\mathbf {B}_{sh\_orth}}({\theta
_\mathtt{{elv}}})}~{\mathbf {R}_{{\mathbf
{r}_\mathtt{{sh\_elv}}}}^{\mathbf {B}_{sh\_orth}}({\theta
_\mathtt{{sh\_elv}}})}~{\mathbf {R}_{{\mathbf
{r}_\mathtt{{elv}}}}^{\mathbf {B}_{sh\_orth}}(-{\theta
_\mathtt{{elv}}})}~{\mathbf {R}_{{\mathbf
{r}_\mathtt{{sh\_rot}}}}^{\mathbf {B}_{sh\_orth}}({\theta
_\mathtt{{sh\_rot}}})} \\ &=\small { \begin{bmatrix} A \cos ({\theta
_\mathtt{{sh\_rot}}}) - B \sin ({\theta _\mathtt{{sh\_rot}}}) &{}
-\cos ({\theta _\mathtt{{elv}}}) \sin ({\theta _\mathtt{{sh\_elv}}})
&{} A \sin ({\theta _\mathtt{{sh\_rot}}}) + B \cos ({\theta
_\mathtt{{sh\_rot}}}) \\ \sin ({\theta _\mathtt{{elv}}}) \sin
({\theta _\mathtt{{sh\_elv}}}) \sin ({\theta _\mathtt{{sh\_rot}}})
&{} \cos ({\theta _\mathtt{{sh\_elv}}}) &{} \cos ({\theta
_\mathtt{{elv}}}) \sin ({\theta _\mathtt{{sh\_elv}}}) \sin ({\theta
_\mathtt{{sh\_rot}}})  \\ +\cos ({\theta _\mathtt{{elv}}}) \sin
({\theta _\mathtt{{sh\_elv}}}) \cos ({\theta _\mathtt{{sh\_rot}}})
&& -\sin ({\theta _\mathtt{{elv}}}) \sin ({\theta
_\mathtt{{sh\_elv}}}) \cos ({\theta _\mathtt{{sh\_rot}}})  \\ B \cos
({\theta _\mathtt{{sh\_rot}}}) - C \sin ({\theta
_\mathtt{{sh\_rot}}}) &{} \sin ({\theta _\mathtt{{elv}}}) \sin
({\theta _\mathtt{{sh\_elv}}}) &{} B \sin ({\theta
_\mathtt{{sh\_rot}}}) + C \cos ({\theta _\mathtt{{sh\_rot}}})
\end{bmatrix} }
 \end{aligned}$$

where $$A = \cos ({\theta _\mathtt{{sh\_elv}}}) \cos ^2
({\theta _\mathtt{{elv}}}) + \sin ^2 ({\theta _\mathtt{{elv}}}), $$ $$B= (1 - \cos ({\theta _\mathtt{{sh\_elv}}})) \cos ({\theta
_\mathtt{{elv}}}) \sin ({\theta _\mathtt{{elv}}}),  $$ and $$C = \cos ({\theta _\mathtt{{sh\_elv}}}) \sin ^2 ({\theta
_\mathtt{{elv}}}) + \cos ^2 ({\theta _\mathtt{{elv}}})  .$$

### Appendix 5: Auxiliary calculations for the elbow

The orientation of the forearm is defined by two consecutive rotations in the order of *elbow flexion* and *forearm rotation*. If expressed in $\mathbf {B}_\mathtt{{pro\_sup}}$, the rotation matrix about $\mathbf {r}_\mathtt{{el\_flex}}$ can be estimated as $\mathbf {R}_{{\mathbf {r}_\mathtt{{el\_flex}}}}^{{\mathbf {B}_\mathtt{{pro\_sup}}}}({\theta _\mathtt{{el\_flex}}})$ while rotation about $\mathbf {r}_\mathtt{{pro\_sup}}$ corresponds to the elementary rotation about the first axis of $\mathbf {B}_\mathtt{{pro\_sup}}$ [denoted as $\mathbf {R}_{{\mathbf {r}_\mathtt{{pro\_sup}}}}^{{\mathbf {B}_\mathtt{{pro\_sup}}}}({\theta _\mathtt{{pro\_sup}}})$]. Multiplication of these matrices yields the compound rotation matrix in the elbow as shown in (21) (the corresponding MATLAB script can be found in Appendix 6).

21 $$\begin{aligned} \begin{array}{l} {\mathbf {R}^{elbow}} = {\mathbf {R}_{{\mathbf {r}_\mathtt{{el\_flex}}}}^{{\mathbf {B}_\mathtt{{pro\_sup}}}}({\theta _\mathtt{{el\_flex}}})}~{\mathbf {R}_{{\mathbf {r}_\mathtt{{pro\_sup}}}}^{{\mathbf {B}_\mathtt{{pro\_sup}}}}({\theta _\mathtt{{pro\_sup}}})}  \\ \phantom {{\mathbf {R}^{elbow}}} = \begin{bmatrix} \left( 1 - \cos ({\theta _\mathtt{{el\_flex}}})\right) x^2 + &{} &{} A \sin ({\theta _\mathtt{{pro\_sup}}}) - &{} &{} A \cos ({\theta _\mathtt{{pro\_sup}}}) + \\ \cos ({\theta _\mathtt{{el\_flex}}}) &{} &{} B \cos ({\theta _\mathtt{{pro\_sup}}}) \phantom {-} &{} &{} B \sin ({\theta _\mathtt{{pro\_sup}}}) \phantom {+} \\ z \sin ({\theta _\mathtt{{el\_flex}}}) - &{} &{} D \cos ({\theta _\mathtt{{pro\_sup}}}) - &{} &{} - D \sin ({\theta _\mathtt{{pro\_sup}}}) - \\ C &{} &{} E \sin ({\theta _\mathtt{{pro\_sup}}}) \phantom {-} &{} &{} E \cos ({\theta _\mathtt{{pro\_sup}}}) \\ -y \sin ({\theta _\mathtt{{el\_flex}}}) - &{} &{} G \sin ({\theta _\mathtt{{pro\_sup}}}) + &{} &{} G \cos ({\theta _\mathtt{{pro\_sup}}}) - \\ F &{} &{} H \cos ({\theta _\mathtt{{pro\_sup}}}) \phantom {+} &{} &{} H \sin ({\theta _\mathtt{{pro\_sup}}}) \phantom {-} \end{bmatrix} \\ \\ \begin{array}{ll} \begin{array}{lll} \text {where} &{} &{} \\ {\mathbf {r}_\mathtt{{el\_flex}}^{{\mathbf {B}_\mathtt{{pro\_sup}}}}} &{} = &{} [x~y~z] \\ A &{} = &{} y \sin ({\theta _\mathtt{{el\_flex}}}) - F \\ B &{} = &{} z \sin ({\theta _\mathtt{{el\_flex}}}) + C \\ C &{} = &{} x y \left( \cos ({\theta _\mathtt{{el\_flex}}}) - 1 \right) \end{array} &{} \begin{array}{lll} D &{} = &{} \left( 1 - \cos ({\theta _\mathtt{{el\_flex}}}) \right) y^2 + \cos ({\theta _\mathtt{{el\_flex}}}) \\ E &{} = &{} x \sin ({\theta _\mathtt{{el\_flex}}}) + y z \left( \cos ({\theta _\mathtt{{el\_flex}}}) - 1 \right) \\ F &{} = &{} x z \left( \cos ({\theta _\mathtt{{el\_flex}}}) - 1 \right) \\ G &{} = &{} \left( 1 - \cos ({\theta _\mathtt{{el\_flex}}}) \right) z^2 + \cos ({\theta _\mathtt{{el\_flex}}}) \\ H &{} = &{} x \sin ({\theta _\mathtt{{el\_flex}}}) - y z \left( \cos ({\theta _\mathtt{{el\_flex}}}) - 1 \right) \end{array} \end{array} \end{array} \end{aligned}$$

### Appendix 7: Auxiliary calculations for the wrist

The orientation of the hand is defined in the model by four consecutive rotations in the order of *deviation*, *flexion*, *proximal-distal r1* and *proximal-distal r3*. Although *deviation* and *flexion* are used here as intermediate rotations, naming convention of actively controlled joint coordinates and intermediate rotations is inconsistent in the model file at this point because active joint coordinates of the wrist are called *deviation* and *flexion*, too. To prevent ambiguity, angle values of controlled joint coordinates of the wrist will be denoted as $\theta _\mathtt{{dev\_c}}$ and $\theta _\mathtt{{flex\_c}}$ further in the text. Intermediate wrist rotations are distributed among two rows of carpal bones with different rotation angles depending on the actual values of $\theta _\mathtt{{dev\_c}}$ and $\theta _\mathtt{{flex\_c}}$ as follows:

1. *Deviation* and *flexion* are defined in the *lunate* body with rotation angle values of $\theta _\mathtt{{dev}}$ = $\theta _\mathtt{{dev\_c}}$ and $\theta _\mathtt{{flex}}$ = $0.5 * {\theta _\mathtt{{flex\_c}}}$.
2. *Proximal-distal r1* and *proximal-distal r3* are defined in the *capitate* body with rotation angle values of $\theta _\mathtt{{pdr1}}$ = $1.5 * {\theta _\mathtt{{dev\_c}}}$ if $\theta _\mathtt{{dev\_c}}$ is negative and $\theta _\mathtt{{pdr1}}$ = $\theta _\mathtt{{dev\_c}}$ otherwise, and $\theta _\mathtt{{pdr3}}$ = $0.5 * {\theta _\mathtt{{flex\_c}}}$.
3. The angle limits for the controlled coordinates are: ${\theta _\mathtt{{dev\_c}}} \in [-10^\circ ,25^\circ ]$ and ${\theta _\mathtt{{flex\_c}}} \in [-70^\circ ,70^\circ ]$.

If expressed in $\mathbf {B}_\mathtt{{pdr3}}$, the rotation matrices about $\mathbf {r}_\mathtt{{dev}}$ and $\mathbf {r}_\mathtt{{flex}}$ can be estimated as $\mathbf {R}_{{\mathbf {r}_\mathtt{{dev}}}}^{{\mathbf {B}_\mathtt{{pdr3}}}}({\theta _\mathtt{{dev}}})$ and $\mathbf {R}_{{\mathbf {r}_\mathtt{{flex}}}}^{{\mathbf {B}_\mathtt{{pdr3}}}}({\theta _\mathtt{{flex}}})$, while rotation matrices for $\mathbf {r}_\mathtt{{pdr1}}$ and $\mathbf {r}_\mathtt{{pdr3}}$ can be expressed as $\mathbf {R}_{{\mathbf {r}_\mathtt{{pdr1}}}}^{{\mathbf {B}_\mathtt{{pdr3}}}}({\theta _\mathtt{{pdr1}}})$ and an elementary rotation about the first axis of $\mathbf {B}_\mathtt{{pdr3}}$, denoted by $\mathbf {R}_{{\mathbf {r}_\mathtt{{pdr3}}}}^{{\mathbf {B}_\mathtt{{pdr3}}}}({\theta _\mathtt{{pdr3}}})$. Similarly to the shoulder and elbow joints, the compound rotation matrix in the wrist can be written as follows:

22 $$\begin{aligned} {\mathbf {R}^{wrist}} = {\mathbf {R}_{{\mathbf {r}_\mathtt{{dev}}}}^{{\mathbf {B}_\mathtt{{pdr3}}}}({\theta _\mathtt{{dev}}})}~{\mathbf {R}_{{\mathbf {r}_\mathtt{{flex}}}}^{{\mathbf {B}_\mathtt{{pdr3}}}}({\theta _\mathtt{{flex}}})}~{\mathbf {R}_{{\mathbf {r}_\mathtt{{pdr1}}}}^{{\mathbf {B}_\mathtt{{pdr3}}}}({\theta _\mathtt{{pdr1}}})}~{\mathbf {R}_{{\mathbf {r}_\mathtt{{pdr3}}}}^{{\mathbf {B}_\mathtt{{pdr3}}}}({\theta _\mathtt{{pdr3}}})} \end{aligned}$$

One difficulty however is that this formulation contains three axis-angle rotations out of four that makes $\mathbf {R}^{wrist}$ a very complex symbolic expression with no closed form algebraic solution for the individual rotation angle values.

This problem was handled using a decomposition approach from the literature. As it is shown by Piovan and Bullo in [48], three Euler angles about arbitrary axes can be determined from a rotation matrix if the rotation axes are known and the following condition is fulfilled:

23 $$\begin{aligned} \left| \mathbf {r}_1^T \left( \mathbf {R} - \mathbf {r}_2^{\phantom {T}} \mathbf {r}_2^T \right) \mathbf {r}_3 \right| \le \sqrt{1 - \left( \mathbf {r}_1^T \mathbf {r}_2^{\phantom {T}}\right) ^2} ~ \sqrt{1 - \left( \mathbf {r}_3^T \mathbf {r}_2^{\phantom {T}}\right) ^2} \end{aligned}$$

where $\mathbf {R}$ is the rotation matrix and $\left\{ \mathbf {r}_1, \mathbf {r}_2, \mathbf {r}_3\right\}$ are the column vector rotation axes. If we assume that $\mathbf {r}_1$, $\mathbf {r}_2$, $\mathbf {r}_3$ and $\mathbf {R}$ satisfy (23), Euler angle values $\left\{ \theta _1, \theta _2, \theta _3 \right\}$ can be calculated as follows:

- $\theta _2$ is one of the two solutions to
  24 $$\begin{aligned} \left( \theta _2 \right) _{1,2} = \mathrm{atan2}(b,a) \pm \mathrm{atan2}\left( \sqrt{a^2 + b^2 - c^2},~c~ \right) \end{aligned}$$
  where $$a = -\mathbf {r}_1^T \widehat{\mathbf {r}}_2^2 \mathbf {r}_3$$, $$b = \mathbf {r}_1^T \widehat{\mathbf {r}}_2 \mathbf {r}_3$$ and $$c = \mathbf {r}_1^T \left( \mathbf {R} - I_3 - \widehat{\mathbf {r}}_2^2 \right) \mathbf {r}_3$$.
- if $\mathbf {R}^T \mathbf {r}_1 \ne \pm \mathbf {r}_3$, then the angles $\theta _1$ and $\theta _3$ are uniquely determined by
  25 $$\begin{aligned} \theta _1 = \phantom {-} \mathrm{atan2} \left( \mathbf {w}_1^T \mathbf {r}_1 \times \mathbf {v}_1, \mathbf {v}_1^T \mathbf {w}_1 - \left( \mathbf {v}_1^T \mathbf {r}_1 \right) \left( \mathbf {w}_1^T \mathbf {r}_1 \right) \right) \end{aligned}$$
  26 $$\begin{aligned} \theta _3 = -\mathrm{atan2} \left( \mathbf {w}_3^T \mathbf {r}_3 \times \mathbf {v}_3, \mathbf {v}_3^T \mathbf {w}_3 - \left( \mathbf {v}_3^T \mathbf {r}_3 \right) \left( \mathbf {w}_3^T \mathbf {r}_3 \right) \right) \end{aligned}$$
  where $$\mathbf {v}_1 = \mathrm{exp} \left( \theta _2 \widehat{\mathbf {r}}_2 \right) \mathbf {r}_3$$, $$\mathbf {w}_1 = \mathbf {R} \mathbf {r}_3$$, $$\mathbf {v}_3 = \mathrm{exp} \left( -\theta _2 \widehat{\mathbf {r}}_2 \right) \mathbf {r}_1$$, $$\mathbf {w}_3 = \mathbf {R}^T \mathbf {r}_1$$.

As the inverse of any rotation matrix equals its transpose and the model defines the equality of $\theta _\mathtt{{flex}}$ $=$ $\theta _\mathtt{{pdr3}}$, (22) can be rewritten into

27 $$\begin{aligned} {\mathbf {R}^{wrist}} \begin{bmatrix} 1&0&0 \\ 0&\cos \left( {\theta _\mathtt{{flex}}} \right)&\sin \left( {\theta _\mathtt{{flex}}} \right) \\ 0&-\sin \left( {\theta _\mathtt{{flex}}} \right)&\cos \left( {\theta _\mathtt{{flex}}} \right) \\ \end{bmatrix} = {\mathbf {R}_{{\mathbf {r}_\mathtt{{dev}}}}^{{\mathbf {B}_\mathtt{{pdr3}}}}({\theta _\mathtt{{dev}}})}~{\mathbf {R}_{{\mathbf {r}_\mathtt{{flex}}}}^{{\mathbf {B}_\mathtt{{pdr3}}}}({\theta _\mathtt{{flex}}})}~{\mathbf {R}_{{\mathbf {r}_\mathtt{{pdr1}}}}^{{\mathbf {B}_\mathtt{{pdr3}}}}({\theta _\mathtt{{pdr1}}})}. \end{aligned}$$

Having $\widetilde{\mathbf {R}}^{wrist}_{}$ $=$ $\mathbf {R}^{wrist}$, (24) can be used to express $\theta _\mathtt{{flex}}$ from (27) as follows (all $\mathbf {r}_\mathtt{{axis\_id}}^{{\mathbf {B}_\mathtt{{axis\_id\_0}}}}$ here are considered as column vectors):

28 $$\begin{aligned} \begin{array}{l} \left( {\theta _\mathtt{{flex}}} \right) _{1,2} = \mathrm{atan2}(b,a) \pm \mathrm{atan2}\left( \sqrt{a^2 + b^2 - c^2},~c~ \right) ,\\ \text {where} \\ \begin{array}{rll} a &{} = &{} - \left( {\mathbf {r}_\mathtt{{dev}}^{{\mathbf {B}_\mathtt{{pdr3}}}}} \right) ^T \left( \widehat{\mathbf {r}}_{\texttt {flex}}^{{\mathbf {B}_\mathtt{{pdr3}}}} \right) ^2 {\mathbf {r}_\mathtt{{pdr1}}^{{\mathbf {B}_\mathtt{{pdr3}}}}} \\ b &{} = &{} \left( {\mathbf {r}_\mathtt{{dev}}^{{\mathbf {B}_\mathtt{{pdr3}}}}} \right) ^T \widehat{\mathbf {r}}_{\texttt {flex}}^{{\mathbf {B}_\mathtt{{pdr3}}}} ~ {\mathbf {r}_\mathtt{{pdr1}}^{{\mathbf {B}_\mathtt{{pdr3}}}}} \\ c &{} = &{} \left( {\mathbf {r}_\mathtt{{dev}}^{{\mathbf {B}_\mathtt{{pdr3}}}}} \right) ^T \left( {\widetilde{\mathbf {R}}^{wrist}_{}} \begin{bmatrix} 1 &{} 0 &{} 0 \\ 0 &{} \cos \left( {\theta _\mathtt{{flex}}} \right) &{} \sin \left( {\theta _\mathtt{{flex}}} \right) \\ 0 &{} -\sin \left( {\theta _\mathtt{{flex}}} \right) &{} \cos \left( {\theta _\mathtt{{flex}}} \right) \\ \end{bmatrix} - I_3 - \left( \widehat{\mathbf {r}}_{\texttt {flex}}^{{\mathbf {B}_\mathtt{{pdr3}}}} \right) ^2 \right) {\mathbf {r}_\mathtt{{pdr1}}^{{\mathbf {B}_\mathtt{{pdr3}}}}}  \\ &{} = &{} x \cos \left( {\theta _\mathtt{{flex}}} \right) + y \sin \left( {\theta _\mathtt{{flex}}} \right) + z \end{array} \end{array} \end{aligned}$$

where *x*, *y* and *z* are defined as

$$\begin{aligned} \begin{array}{llll} &{} x &{} = &{} \left( {\mathbf {r}_\mathtt{{pdr1}}^{{\mathbf {B}_\mathtt{{pdr3}}}}} \right) ^T ~ \left[ 0 , \left( {\mathbf {r}_\mathtt{{dev}}^{{\mathbf {B}_\mathtt{{pdr3}}}}} \right) ^T {\widetilde{\mathbf {R}}^{wrist}_{(:,(2,3))}} \right] ^T \\ &{} y &{} = &{} \left[ {\mathbf {r}_\mathtt{{pdr1}}^{{\mathbf {B}_\mathtt{{pdr3}}}}}_{(3)}, -{\mathbf {r}_\mathtt{{pdr1}}^{{\mathbf {B}_\mathtt{{pdr3}}}}}_{(2)} \right] \left[ \left( {\mathbf {r}_\mathtt{{dev}}^{{\mathbf {B}_\mathtt{{pdr3}}}}} \right) ^T {\widetilde{\mathbf {R}}^{wrist}_{(:,(2,3))}} \right] ^T \\ &{} z &{} = &{} \left[ {\mathbf {r}_\mathtt{{pdr1}}^{{\mathbf {B}_\mathtt{{pdr3}}}}}_{(1)}, -{\mathbf {r}_\mathtt{{pdr1}}^{{\mathbf {B}_\mathtt{{pdr3}}}}}_{(2)}, -{\mathbf {r}_\mathtt{{pdr1}}^{{\mathbf {B}_\mathtt{{pdr3}}}}}_{(3)} \right] \left( \begin{bmatrix} {\widetilde{\mathbf {R}}^{wrist}_{(1,1)}} &{} {\widetilde{\mathbf {R}}^{wrist}_{(2,1)}} &{} {\widetilde{\mathbf {R}}^{wrist}_{(3,1)}} \\ 0 &{} 0 &{} 0 \\ 0 &{} 0 &{} 0 \end{bmatrix}^{\phantom {M}} \right. \\ &{} &{} &{} \left. + \begin{bmatrix} -1 &{} 0 &{} 0 \\ 0 &{} 1 &{} 0 \\ 0 &{} 0 &{} 1 \\ \end{bmatrix} {\mathbf {r}_\mathtt{{flex}}^{{\mathbf {B}_\mathtt{{pdr3}}}}} \left( {\mathbf {r}_\mathtt{{flex}}^{{\mathbf {B}_\mathtt{{pdr3}}}}} \right) ^T \right) ^{\phantom {M}} {\mathbf {r}_\mathtt{{dev}}^{{\mathbf {B}_\mathtt{{pdr3}}}}} \end{array} \end{aligned}$$

## Additional files

**Additional file 1.** OpenSim version of the used upper limb model.

**Additional file 2.** Marker sets defined for the study.

**Additional file 3.** Joint angle reconstruction figures for all simulated trajectories.

## Abbreviations

LoS: line of sight
MEMS: micro-electro-mechanical system
IMU: inertial measurement unit
API: application programming interface
IK: inverse kinematics
XML: extensible markup language
ANSI: American National Standards Institute
MCU: microcontroller unit
FPU: floating point unit
PC: personal computer
RMS: root mean square

## References

[1] Zheng H, Black ND, Harris ND (2005). Position-sensing technologies for movement analysis in stroke rehabilitation. Med Biol Eng Comput.

[2] Wu CY, Lin KC, Chen HC, Chen IH, Hong WH (2007). Effects of modified constraint-induced movement therapy on movement kinematics and daily function in patients with stroke: a kinematic study of motor control mechanisms. Neurorehabil Neural Repair.

[3] Zhou H, Hu H (2008). Human motion tracking for rehabilitation—a survey. Biomed Signal Process Control.

[4] Stephenson JL, Lamontagne A, De Serres SJ (2009). The coordination of upper and lower limb movements during gait in healthy and stroke individuals. Gait Posture.

[5] O’Donoghue P (2010). Research methods for sports performance analysis.

[6] Yang N, Zhang M, Huang C, Jin D (2002). Motion quality evaluation of upper limb target-reaching movements. Med Eng Phys.

[7] Vandenberghe A, Levin O, De Schutter J, Swinnen S, Jonkers I (2010). Three-dimensional reaching tasks: effect of reaching height and width on upper limb kinematics and muscle activity. Gait Posture.

[8] Borbély BJ, Straube A, Eggert T (2013). Motor synergies during manual tracking differ between familiar and unfamiliar trajectories. Exp Brain Res.

[9] Vignais N, Marin F (2014). Analysis of the musculoskeletal system of the hand and forearm during a cylinder grasping task. Int J Ind Ergon.

[10] Song D, Lan N, Loeb GE, Gordon J (2008). Model-based sensorimotor integration for multi-joint control: development of a virtual arm model. Ann Biomed Eng.

[11] Park MS, Chung CY, Lee SH, Choi IH, Cho TJ, Yoo WJ, Park BSMY, Lee KM (2009). Effects of distal hamstring lengthening on sagittal motion in patients with diplegia. Hamstring length and its clinical use. Gait Posture.

[12] Arnold EM, Ward SR, Lieber RL, Delp SL (2010). A model of the lower limb for analysis of human movement. Ann Biomed Eng.

[13] Veeger DHEJ (2011). “ What if”: the use of biomechanical models for understanding and treating upper extremity musculoskeletal disorders. Man Ther.

[14] Gustus A, Stillfried G, Visser J (2012). Human hand modelling: kinematics, dynamics, applications. Biol Cybern.

[15] Bolsterlee B, Veeger DHEJ, Chadwick EK (2013). Clinical applications of musculoskeletal modelling for the shoulder and upper limb. Med Biol Eng Comput.

[16] Luinge HJ, Veltink PH (2005). Measuring orientation of human body segments using miniature gyroscopes and accelerometers. Med Biol Eng Comput.

[17] Sabatelli S, Galgani M, Fanucci L, Rocchi A. A double stage Kalman filter for sensor fusion and orientation tracking in 9D IMU. In: Sensors applications symposium (SAS). New York: IEEE; 2012. p. 1–5.

[18] Madgwick SOH, Harrison AJL, Vaidyanathan A (2011). Estimation of IMU and MARG orientation using a gradient descent algorithm. IEEE Int Conf Rehabil Robot.

[19] Mahony R, Hamel T, Pflimlin JM (2008). Nonlinear complementary filters on the special orthogonal group. IEEE Trans Autom Control.

[20] Tian Y, Wei H, Tan J (2013). An adaptive-gain complementary filter for real-time human motion tracking with MARG sensors in free-living environments. IEEE Trans Neural Syst Rehabil Eng.

[21] Olivares A, Górriz JM, Ramírez J, Olivares G (2011). Accurate human limb angle measurement: sensor fusion through Kalman, least mean squares and recursive least-squares adaptive filtering. Meas Sci Technol.

[22] Cutti AG, Giovanardi A, Rocchi L, Davalli A, Sacchetti R (2008). Ambulatory measurement of shoulder and elbow kinematics through inertial and magnetic sensors. Med Biol Eng Comput.

[23] Kontaxis A, Cutti AG, Johnson GR, Veeger HEJ (2009). A framework for the definition of standardized protocols for measuring upper-extremity kinematics. Clin Biomech.

[24] de Vries WHK, Veeger HEJ, Cutti AG, Baten C, van der Helm FCT (2010). Functionally interpretable local coordinate systems for the upper extremity using inertial & magnetic measurement systems. J Biomech.

[25] Parel I, Cutti AG, Fiumana G, Porcellini G, Verni G, Accardo AP (2012). Ambulatory measurement of the scapulohumeral rhythm: intra- and inter-rater reliability of a protocol based on inertial and magnetic sensors. Gait Posture.

[26] Holzbaur KRS, Murray WM, Delp SL (2005). A model of the upper extremity for simulating musculoskeletal surgery and analyzing neuromuscular control. Ann Biomed Eng.

[27] Wu G, Van Der Helm FCT, Veeger HEJ, Makhsous M, Van Roy P, Anglin C, Nagels J, Karduna AR, McQuade K, Wang X, Werner FW, Buchholz B (2005). ISB recommendation on definitions of joint coordinate systems of various joints for the reporting of human joint motion—Part II: Shoulder, elbow, wrist and hand. J Biomech.

[28] Vicon Oxford Foot Model. https://www.vicon.com/products/software/oxford-foot-model. Accessed 7 Nov 2016.

[29] Motive: body software for OptiTrack. http://www.optitrack.com/products/motive/body/. Accessed 7 Nov 2016.

[30] van den Bogert AJ, Geijtenbeek T, Even-Zohar O, Steenbrink F, Hardin EC (2013). A real-time system for biomechanical analysis of human movement and muscle function. Med Biol Eng Comput.

[31] Delp SL, Loan JP, Hoy MG, Zajac FE, Topp EL, Rosen JM (1990). An interactive graphics-based model of the lower extremity to study orthopaedic surgical procedures. IEEE Trans Biomed Eng.

[32] Delp SL, Anderson FC, Arnold AS, Loan P, Habib A, John CT, Guendelman E, Thelen DG (2007). OpenSim: open-source software to create and analyze dynamic simulations of movement. IEEE Trans Biomed Eng.

[33] Muceli S, Farina D (2012). Simultaneous and proportional estimation of hand kinematics from EMG during mirrored movements at multiple degrees-of-freedom. IEEE Trans Neural Syst Rehabil Eng.

[34] Jiang N, Vest-Nielsen JL, Muceli S, Farina D (2012). EMG-based simultaneous and proportional estimation of wrist/hand dynamics in uni-lateral trans-radial amputees. J Neuroeng Rehabil.

[35] Jiang N, Muceli S, Graimann B, Farina D (2013). Effect of arm position on the prediction of kinematics from EMG in amputees. Med Biol Eng Comput.

[36] Borbély BJ, Szolgay P. Estimating the instantaneous wrist flexion angle from multi-channel surface EMG of forearm muscles. In: 2013 IEEE biomedical circuits and systems conference, BioCAS. New York: IEEE; 2013. p. 77–80.

[37] Blana D, Kyriacou T, Lambrecht JM, Chadwick EK (2015). Feasibility of using combined EMG and kinematic signals for prosthesis control: a simulation study using a virtual reality environment. J Electromyogr Kinesiol.

[38] Borbély BJ, Szolgay P. A system concept for emg classification from measurement to deployment. In: 2016 15th international workshop on cellular nanoscale networks and their applications (CNNA). 2016. p. 121–122.

[39] Schmidhuber J (2015). Deep learning in neural networks: an overview. Neural Netw.

[40] Holzbaur KRS, Murray WM, Delp SL. Upper extremity kinematic model, Simtk resource. https://simtk.org/home/up-ext-model. Accessed 6 Jul 2016.

[41] Delp SL, Loan P, Krystyne B. SIMM 7.0 for windows user’s manual. 2013. http://www.musculographics.com/download/SIMM7.0UserGuide.pdf. Accessed 6 Jul 2016.

[42] Hicks J. OpenSim documentations: how scaling works. http://simtk-confluence.stanford.edu:8080/display/OpenSim/How+Scaling+Works. Accessed 6 Jul 2016.

[43] Hicks J. OpenSim documentations: how inverse kinematics works. http://simtk-confluence.stanford.edu:8080/display/OpenSim/How+Inverse+Kinematics+Works. Accessed 6 Jul 2016.

[44] Press WH, Teukolsky SA, Vetterling WT, Flannery BP (1992). Numerical recipes in the art of scientific computing.

[45] Borbély BJ, Tihanyi A, Szolgay P. A measurement system for wrist movements in biomedical applications. In: 2015 European conference on circuit theory and design (ECCTD). New York: IEEE. p. 1–4.

[46] Bonnet S, Bassompierre C, Godin C, Lesecq S, Barraud A (2009). Calibration methods for inertial and magnetic sensors. Sens Actuators A Phys.

[47] Vanegas M, Stirling L. Characterization of inertial measurement unit placement on the human body upon repeated donnings. In: 2015 IEEE 12th international conference on wearable and implantable body sensor networks (BSN). New York: IEEE; 2015. p. 1–6.

[48] Piovan G, Bullo F (2012). On coordinate-free rotation decomposition: Euler angles about arbitrary axes. IEEE Trans Robot.

