# Supplementary material for: Real-time inverse kinematics for the upper limb: a model-based algorithm using segment orientations
Source: Biomed Eng Online. 2017 Jan 17;16:21. doi: 10.1186/s12938-016-0291-x (PMC5240469; doi:10.1186/s12938-016-0291-x)
Supplement: Supplementary file 3 — Additional file 3. Joint angle reconstruction figures for all simulated trajectories. [file 12938_2016_291_MOESM3_ESM.pdf]

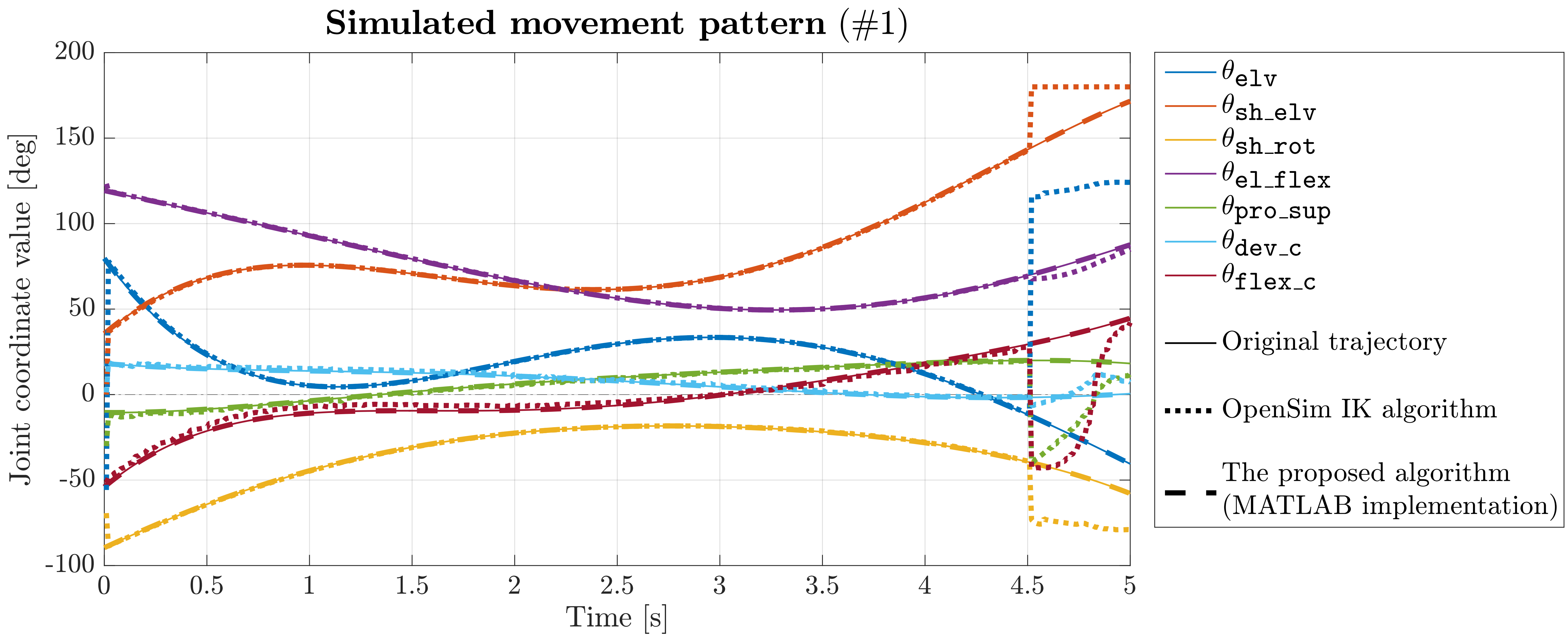

Simulated movement pattern (#1)
Joint coordinate value [deg]
Time [s]
200
150
100
50
0
-50
-100
0
0.5
1
1.5
2
2.5
3
3.5
4
4.5
5
θelv
θsh_elv
θsh_rot
θel_flex
θpro_sup
θdev_c
θflex_c
Original trajectory
OpenSim IK algorithm
The proposed algorithm
(MATLAB implementation)

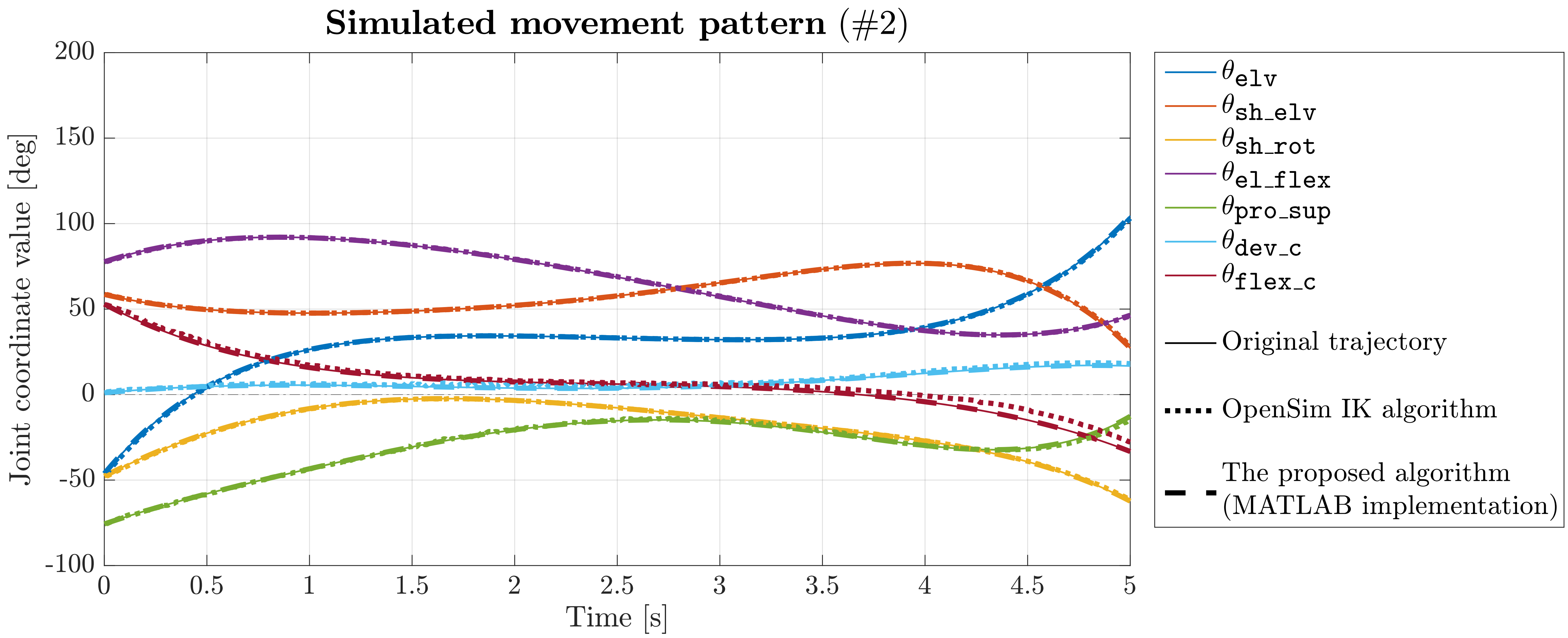

Simulated movement pattern (#2)
Joint coordinate value [deg]
200
150
100
50
0
-50
-100
0
0.5
1
1.5
2
2.5
3
3.5
4
4.5
5
Time [s]
$\theta_{\texttt{elv}}$
$\theta_{\texttt{sh\_elv}}$
$\theta_{\texttt{sh\_rot}}$
$\theta_{\texttt{el\_flex}}$
$\theta_{\texttt{pro\_sup}}$
$\theta_{\texttt{dev\_c}}$
$\theta_{\texttt{flex\_c}}$
Original trajectory
OpenSim IK algorithm
The proposed algorithm
(MATLAB implementation)

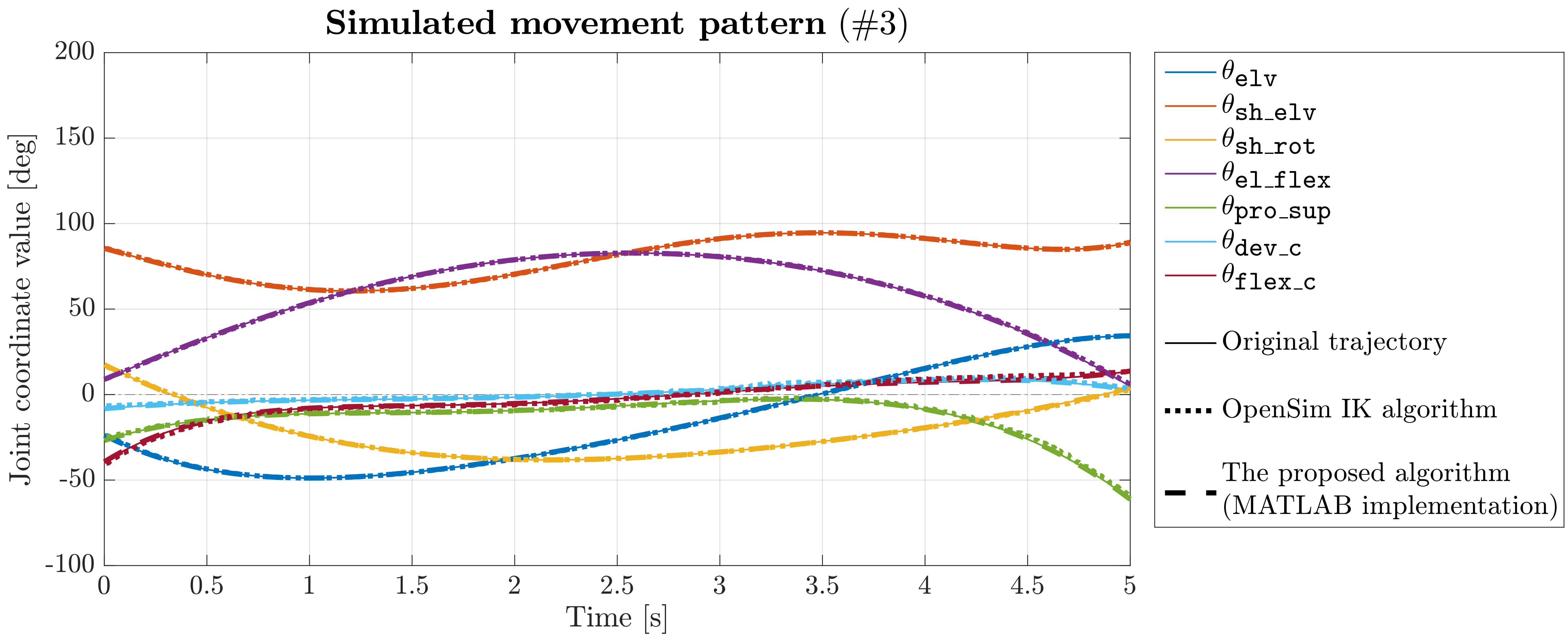

Simulated movement pattern (#3)
Joint coordinate value [deg]
Time [s]
200
150
100
50
0
-50
-100
0
0.5
1
1.5
2
2.5
3
3.5
4
4.5
5
$\theta_{\texttt{elv}}$
$\theta_{\texttt{sh\_elv}}$
$\theta_{\texttt{sh\_rot}}$
$\theta_{\texttt{el\_flex}}$
$\theta_{\texttt{pro\_sup}}$
$\theta_{\texttt{dev\_c}}$
$\theta_{\texttt{flex\_c}}$
Original trajectory
OpenSim IK algorithm
The proposed algorithm (MATLAB implementation)

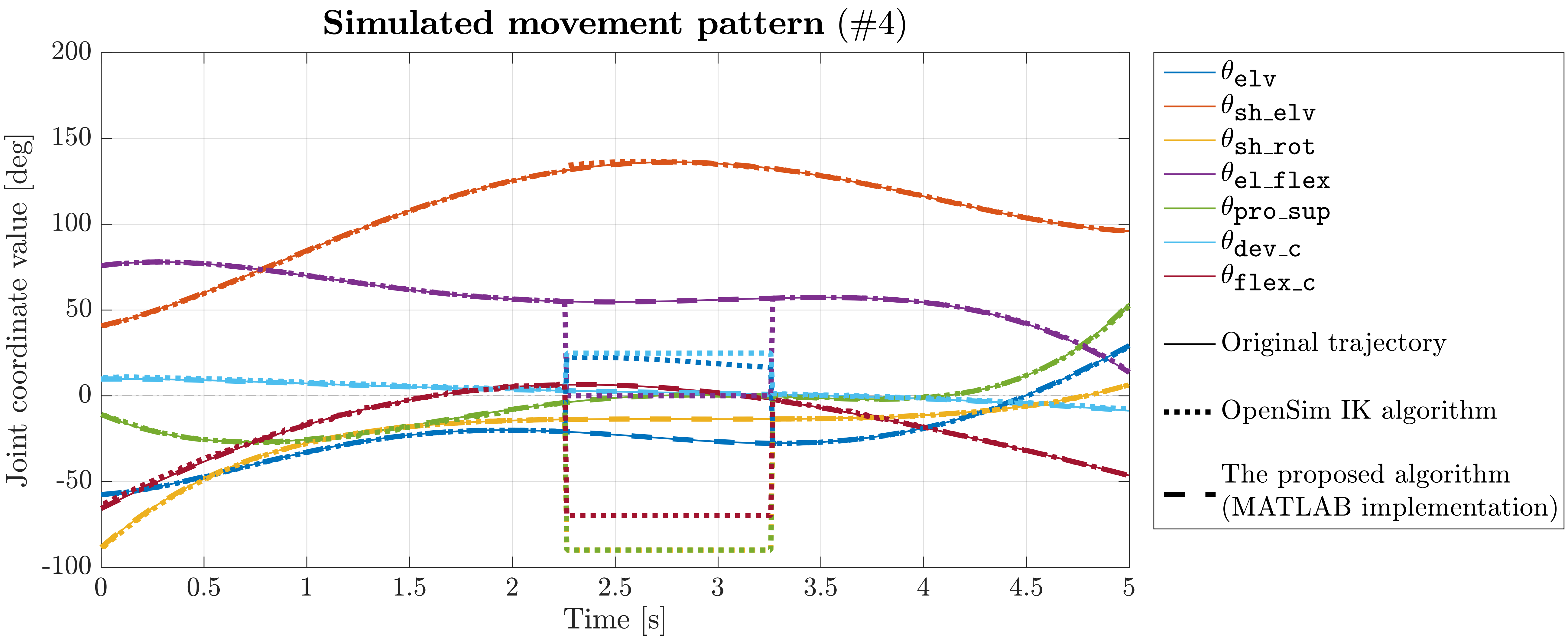

Simulated movement pattern (#4)
Joint coordinate value [deg]
Time [s]
200
150
100
50
0
-50
-100
0
0.5
1
1.5
2
2.5
3
3.5
4
4.5
5
θelv
θsh_elv
θsh_rot
θel_flex
θpro_sup
θdev_c
θflex_c
Original trajectory
OpenSim IK algorithm
The proposed algorithm (MATLAB implementation)

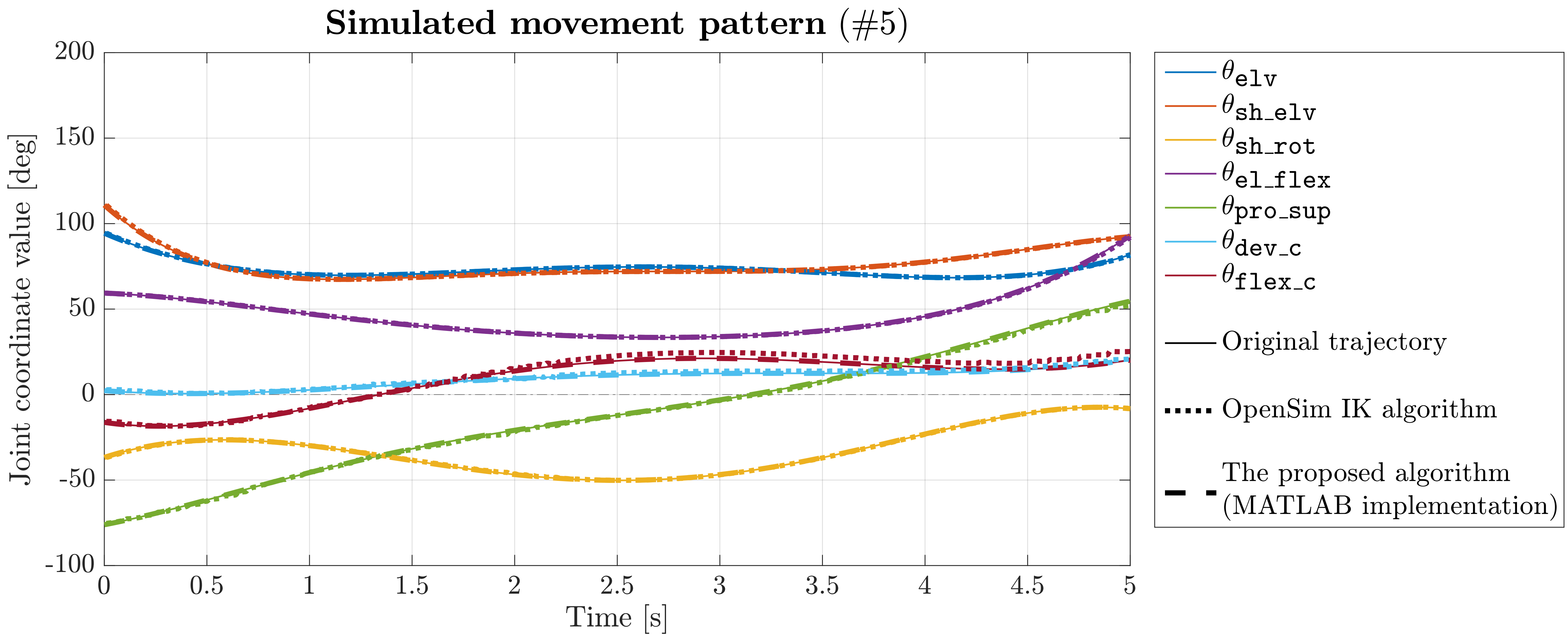

Simulated movement pattern (#5)
Joint coordinate value [deg]
200
150
100
50
0
-50
-100
0
0.5
1
1.5
2
2.5
3
3.5
4
4.5
5
Time [s]
θelv
θsh_elv
θsh_rot
θel_flex
θpro_sup
θdev_c
θflex_c
Original trajectory
OpenSim IK algorithm
The proposed algorithm (MATLAB implementation)

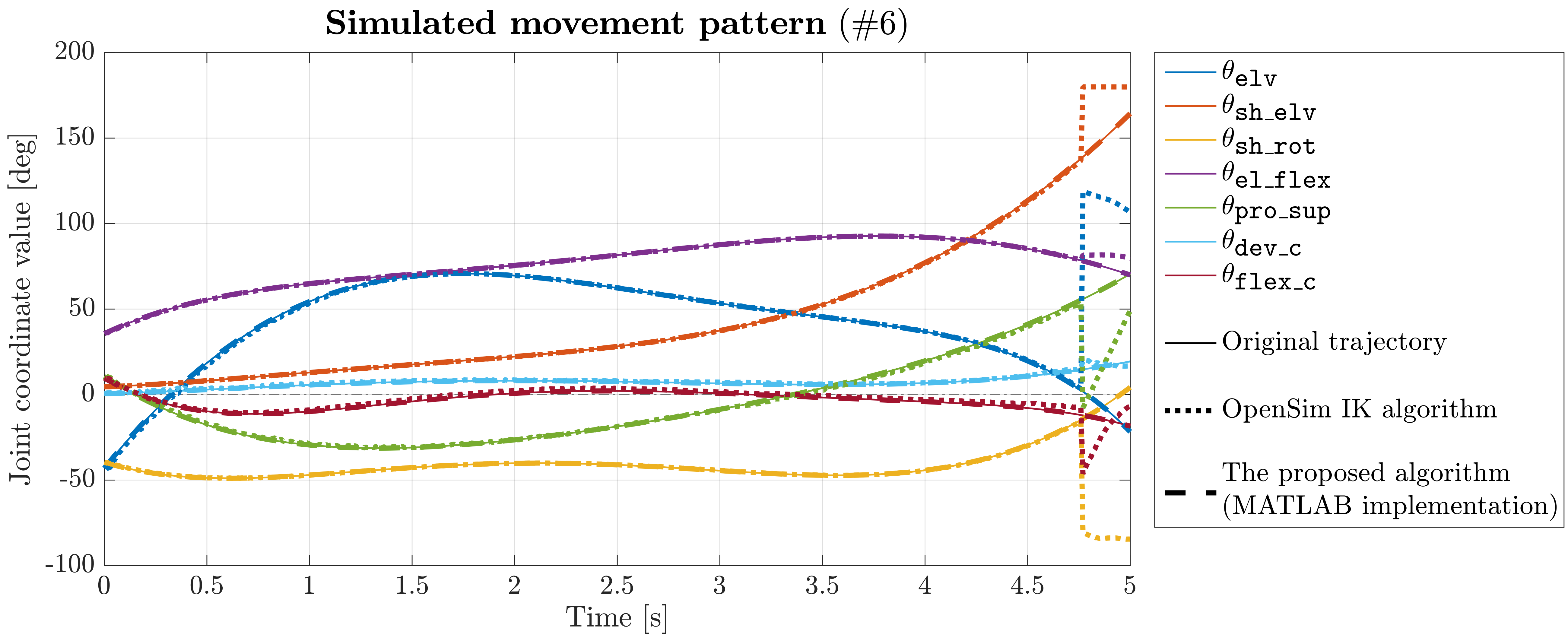
Simulated movement pattern (#6)
Joint coordinate value [deg]
Time [s]
200
150
100
50
0
-50
-100
0
0.5
1
1.5
2
2.5
3
3.5
4
4.5
5
θelv
θsh_elv
θsh_rot
θel_flex
θpro_sup
θdev_c
θflex_c
Original trajectory
OpenSim IK algorithm
The proposed algorithm
(MATLAB implementation)

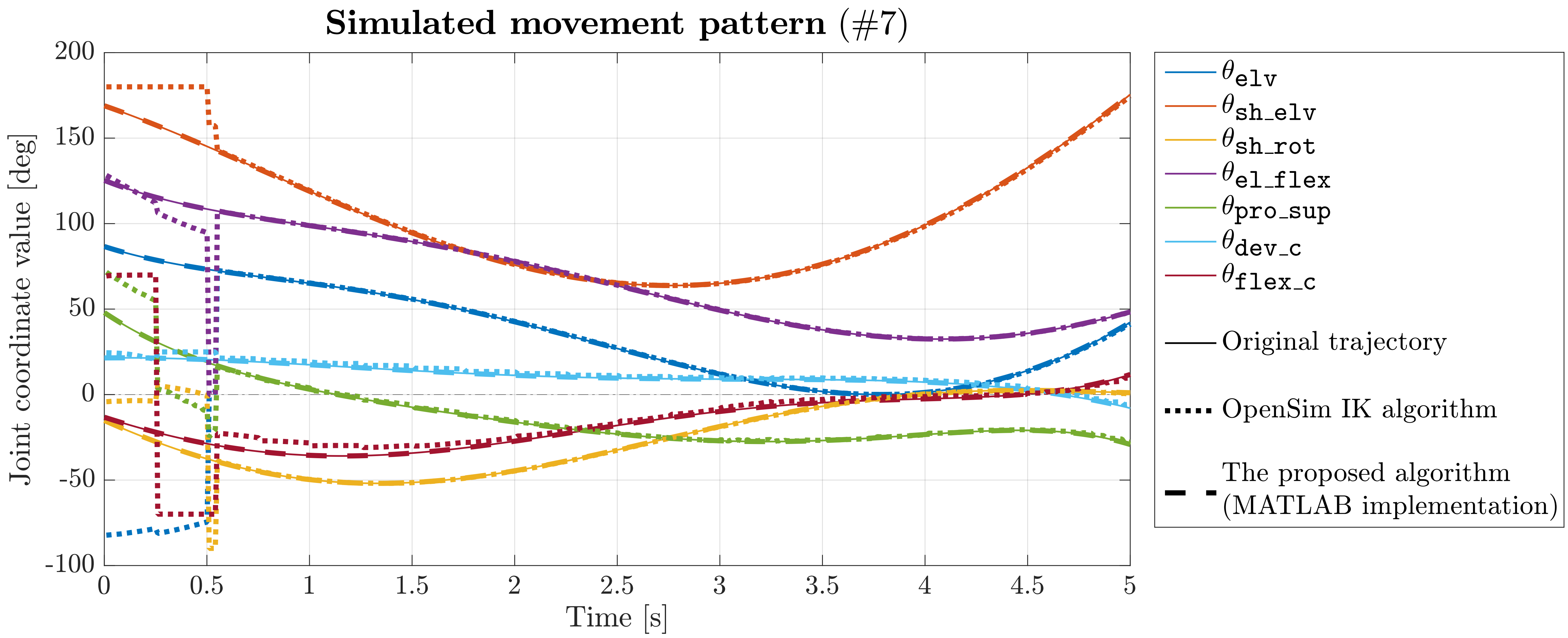

Simulated movement pattern (#7)
Joint coordinate value [deg]
Time [s]
200
150
100
50
0
-50
-100
0
0.5
1
1.5
2
2.5
3
3.5
4
4.5
5
θelv
θsh_elv
θsh_rot
θel_flex
θpro_sup
θdev_c
θflex_c
Original trajectory
OpenSim IK algorithm
The proposed algorithm
(MATLAB implementation)

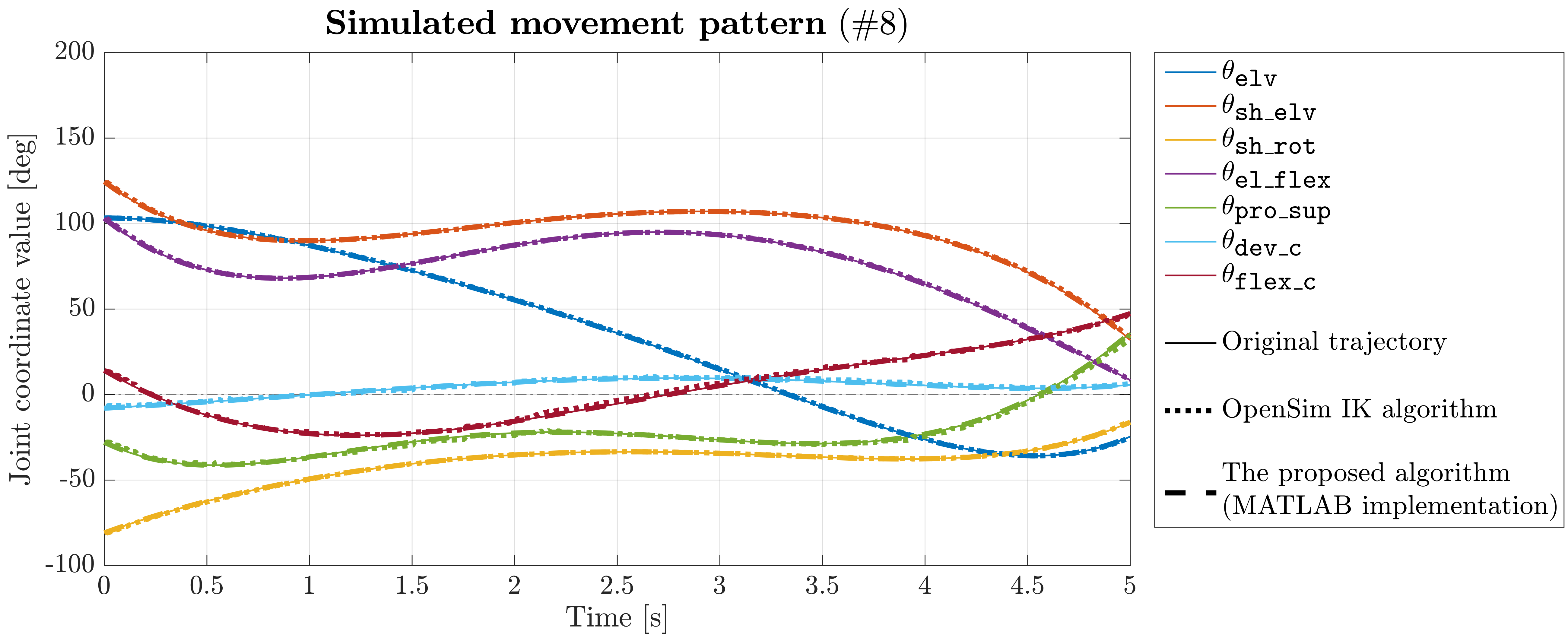

Simulated movement pattern (#8)
Joint coordinate value [deg]
Time [s]
200
150
100
50
0
-50
-100
0
0.5
1
1.5
2
2.5
3
3.5
4
4.5
5
θelv
θsh_elv
θsh_rot
θel_flex
θpro_sup
θdev_c
θflex_c
Original trajectory
OpenSim IK algorithm
The proposed algorithm
(MATLAB implementation)

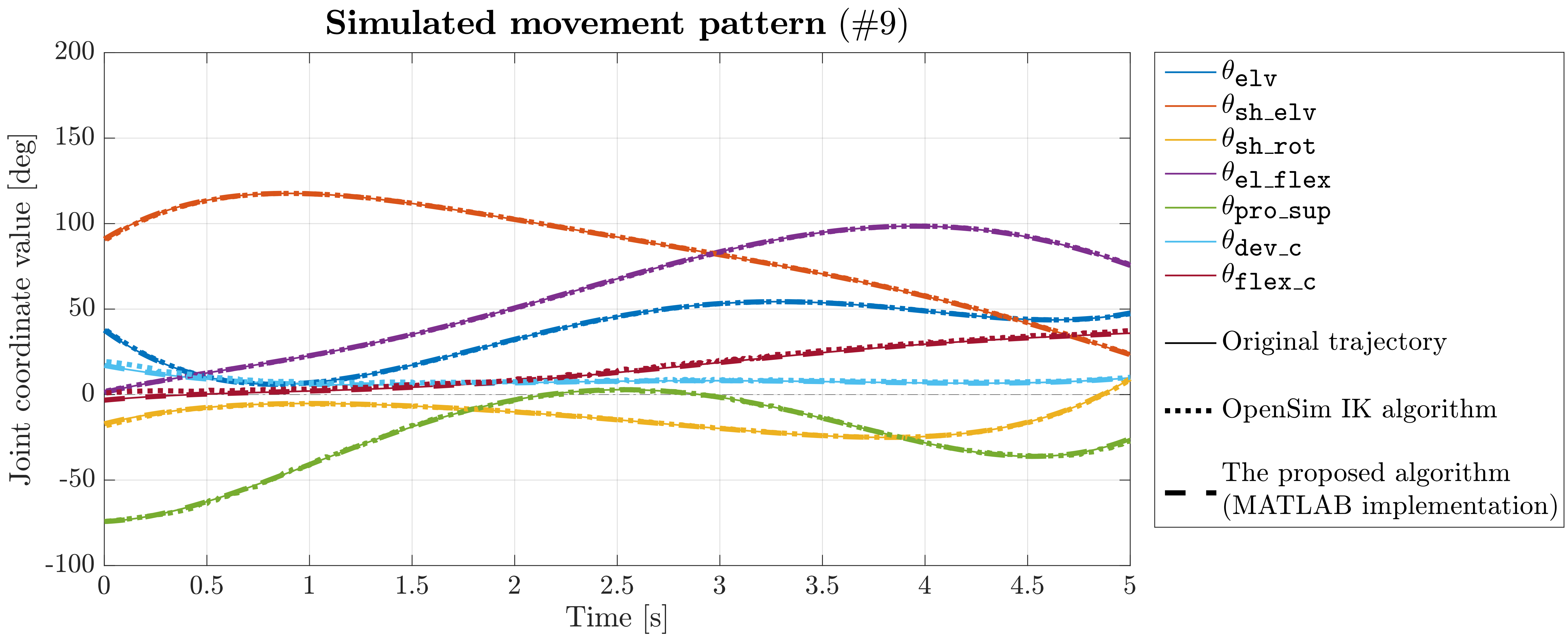

Simulated movement pattern (#9)
Joint coordinate value [deg]
200
150
100
50
0
-50
-100
0
0.5
1
1.5
2
2.5
3
3.5
4
4.5
5
Time [s]
θ_{elv}
θ_{sh_elv}
θ_{sh_rot}
θ_{el_flex}
θ_{pro_sup}
θ_{dev_c}
θ_{flex_c}
Original trajectory
OpenSim IK algorithm
The proposed algorithm
(MATLAB implementation)

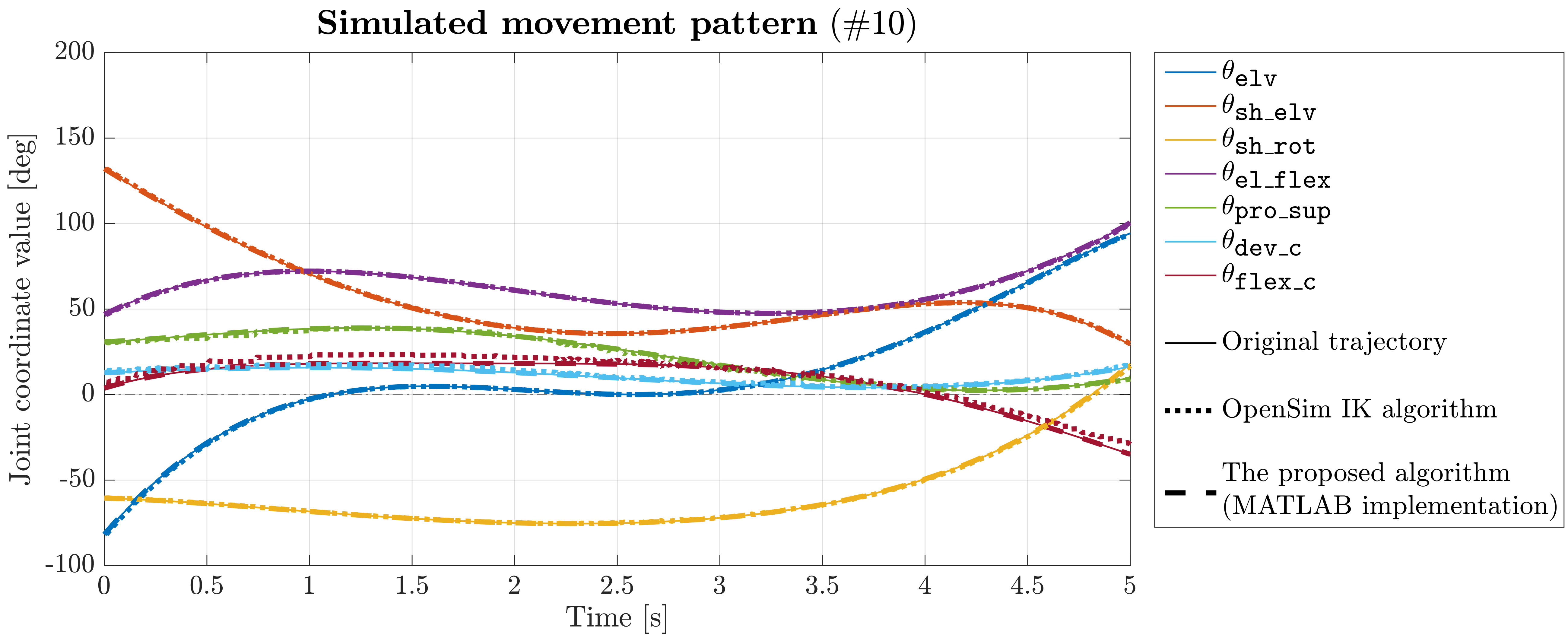

Simulated movement pattern (#10)
200
150
100
50
0
-50
-100
Joint coordinate value [deg]
0
0.5
1
1.5
2
2.5
3
3.5
4
4.5
5
Time [s]
$\theta_{\mathtt{elv}}$
$\theta_{\mathtt{sh\_elv}}$
$\theta_{\mathtt{sh\_rot}}$
$\theta_{\mathtt{el\_flex}}$
$\theta_{\mathtt{pro\_sup}}$
$\theta_{\mathtt{dev\_c}}$
$\theta_{\mathtt{flex\_c}}$
Original trajectory
OpenSim IK algorithm
The proposed algorithm
(MATLAB implementation)

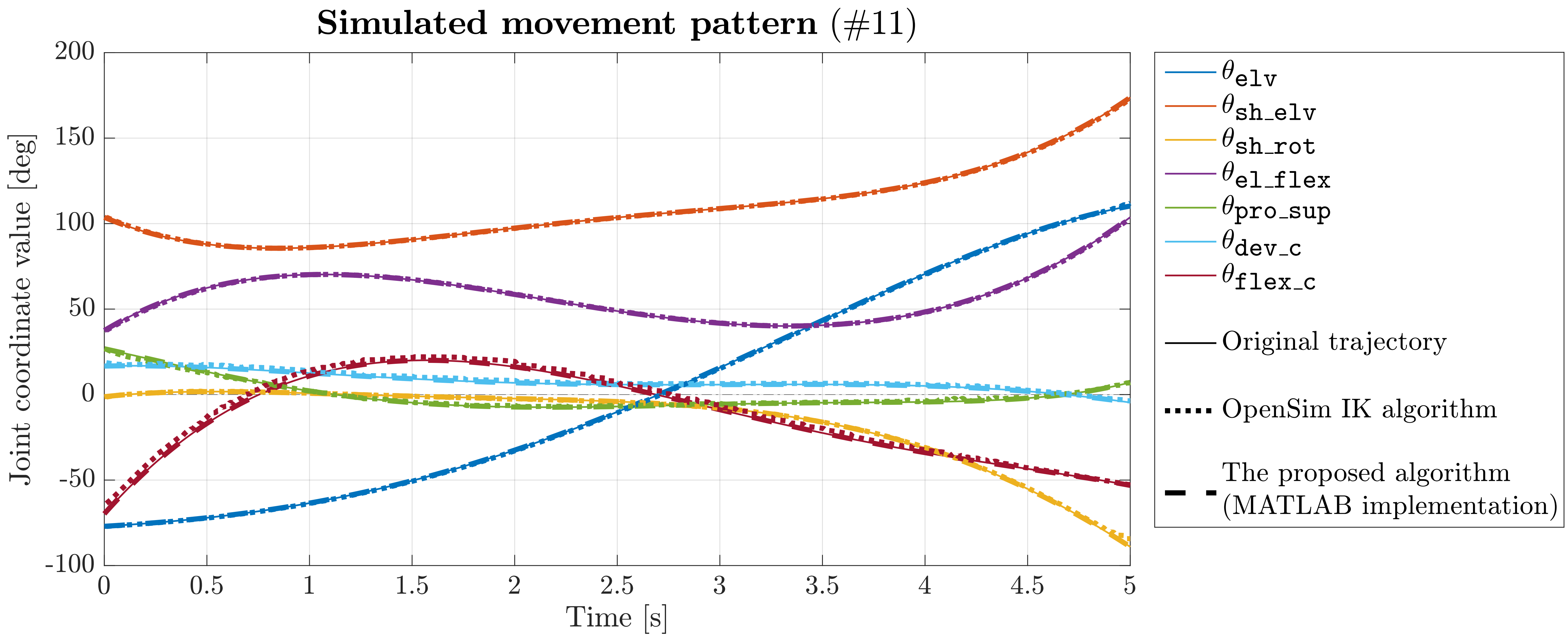

Simulated movement pattern (#11)
Joint coordinate value [deg]
Time [s]
200
150
100
50
0
-50
-100
0
0.5
1
1.5
2
2.5
3
3.5
4
4.5
5
θelv
θsh_elv
θsh_rot
θel_flex
θpro_sup
θdev_c
θflex_c
Original trajectory
OpenSim IK algorithm
The proposed algorithm
(MATLAB implementation)

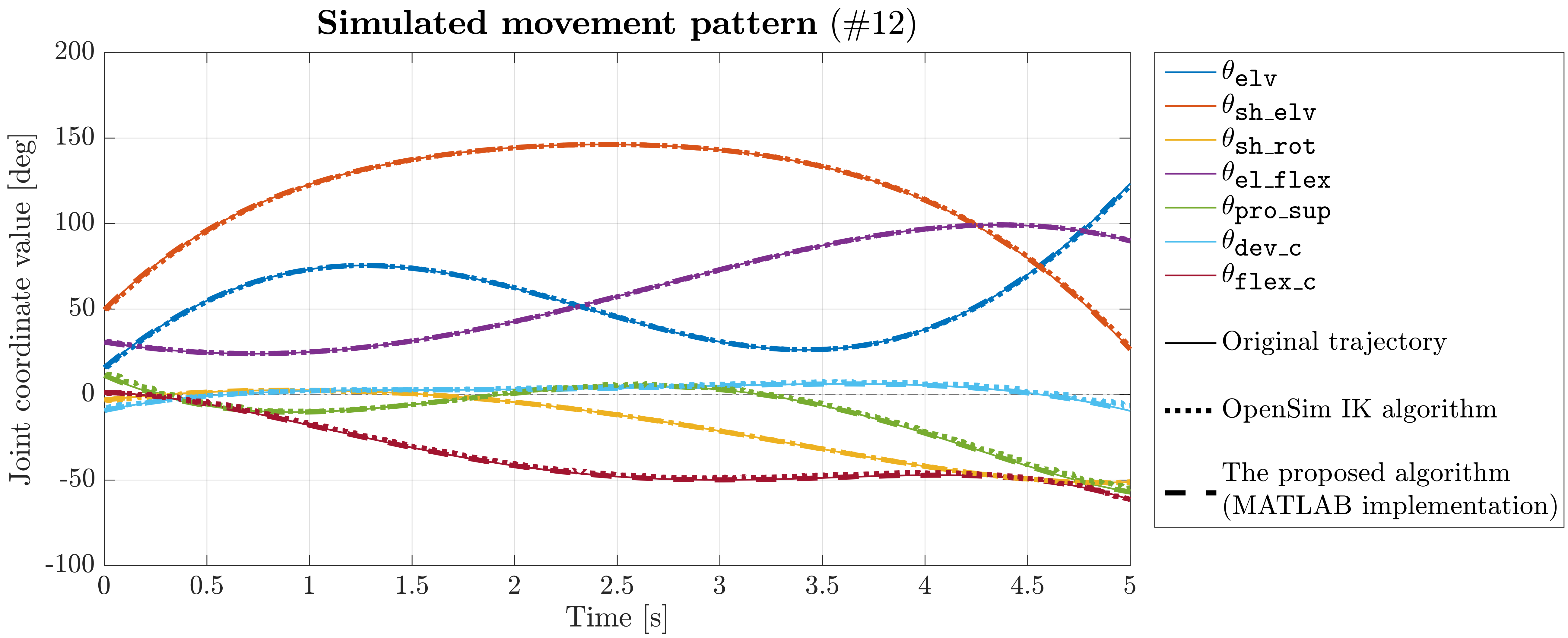

Simulated movement pattern (#12)
Joint coordinate value [deg]
200
150
100
50
0
-50
-100
0
0.5
1
1.5
2
2.5
3
3.5
4
4.5
5
Time [s]
$\theta_{\texttt{elv}}$
$\theta_{\texttt{sh\_elv}}$
$\theta_{\texttt{sh\_rot}}$
$\theta_{\texttt{el\_flex}}$
$\theta_{\texttt{pro\_sup}}$
$\theta_{\texttt{dev\_c}}$
$\theta_{\texttt{flex\_c}}$
Original trajectory
OpenSim IK algorithm
The proposed algorithm
(MATLAB implementation)

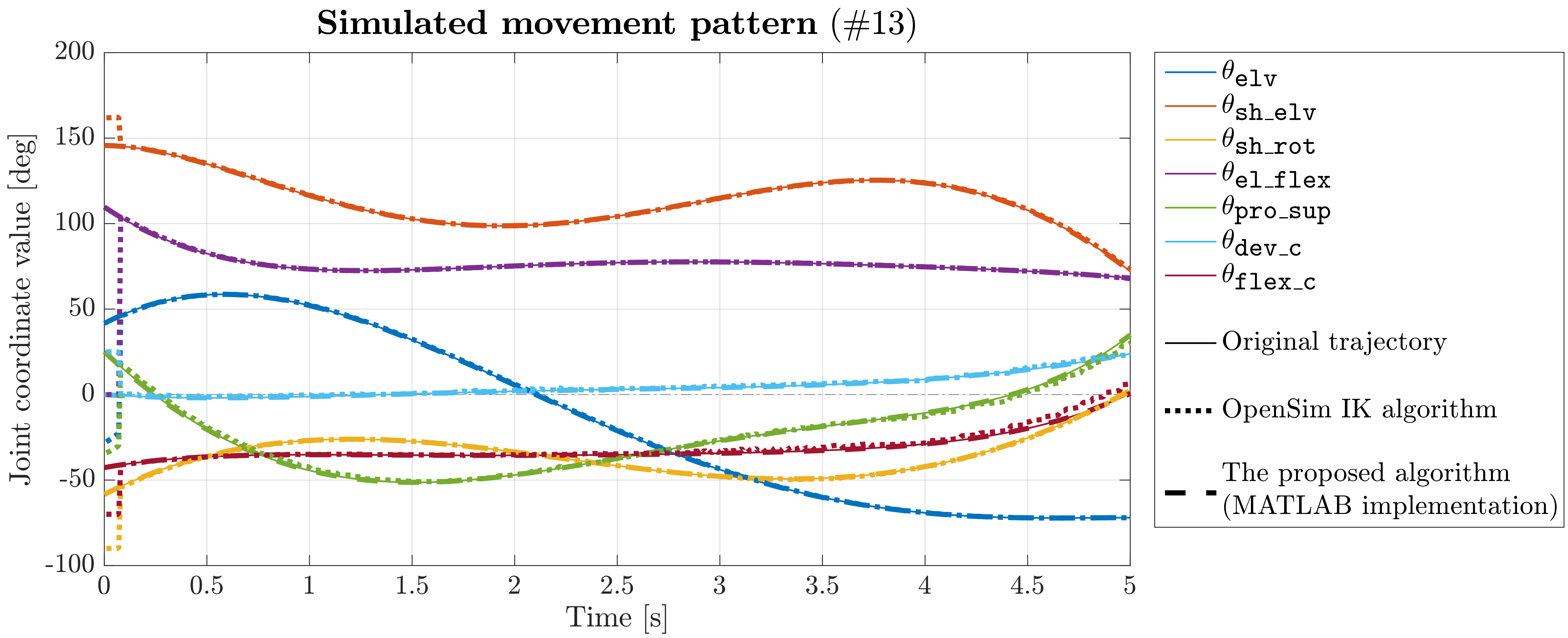

Simulated movement pattern (#13)
Joint coordinate value [deg]
200
150
100
50
0
-50
-100
0
0.5
1
1.5
2
2.5
3
3.5
4
4.5
5
Time [s]
$\theta_{\texttt{elv}}$
$\theta_{\texttt{sh\_elv}}$
$\theta_{\texttt{sh\_rot}}$
$\theta_{\texttt{el\_flex}}$
$\theta_{\texttt{pro\_sup}}$
$\theta_{\texttt{dev\_c}}$
$\theta_{\texttt{flex\_c}}$
Original trajectory
OpenSim IK algorithm
The proposed algorithm (MATLAB implementation)

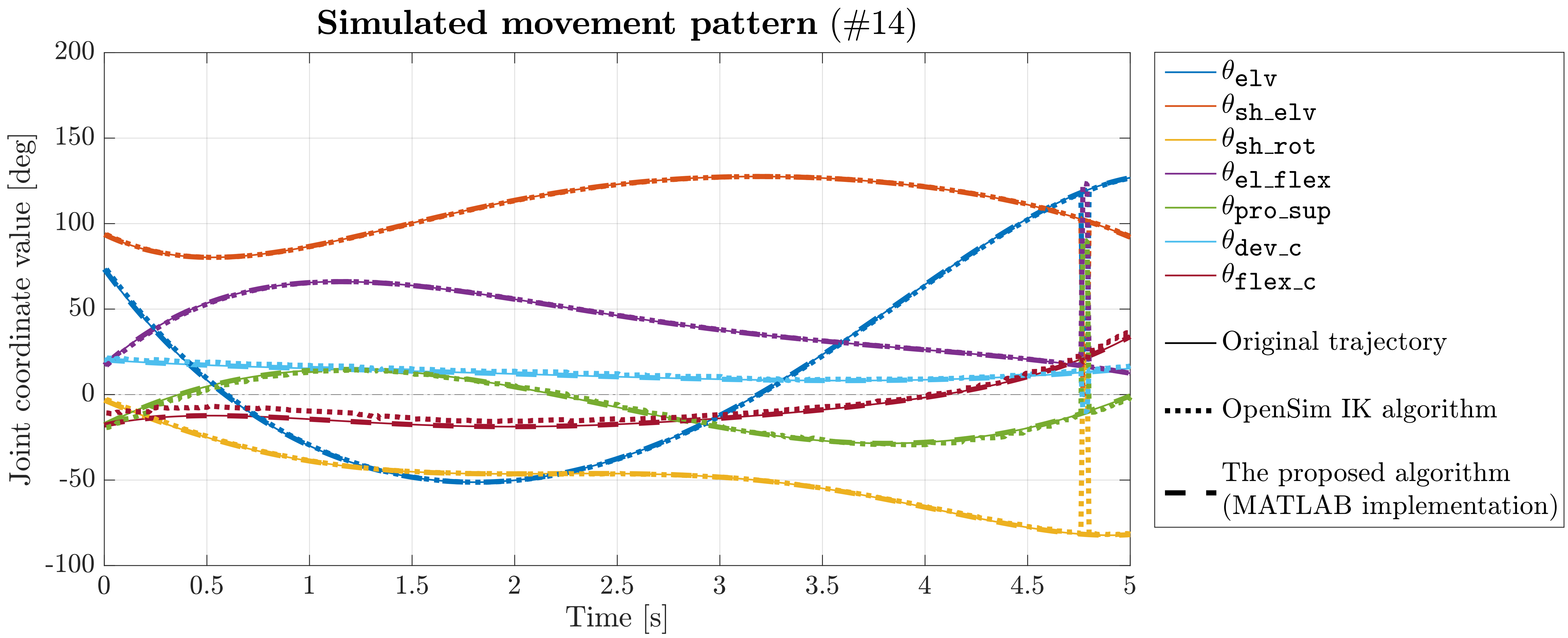

Simulated movement pattern (#14)
Joint coordinate value [deg]
200
150
100
50
0
-50
-100
0
0.5
1
1.5
2
2.5
3
3.5
4
4.5
5
Time [s]
θelv
θsh_elv
θsh_rot
θel_flex
θpro_sup
θdev_c
θflex_c
Original trajectory
OpenSim IK algorithm
The proposed algorithm (MATLAB implementation)

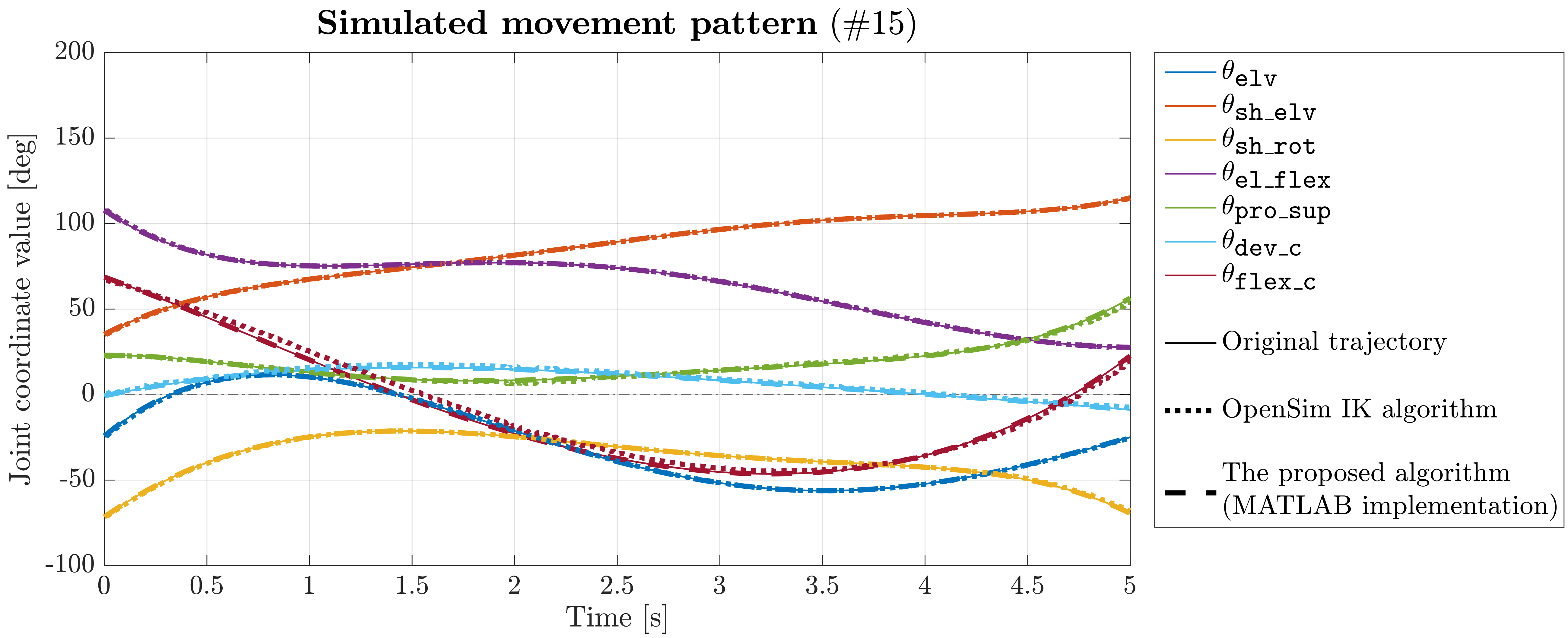

Simulated movement pattern (#15)
Joint coordinate value [deg]
Time [s]
200
150
100
50
0
-50
-100
0
0.5
1
1.5
2
2.5
3
3.5
4
4.5
5
$\theta_{\mathtt{elv}}$
$\theta_{\mathtt{sh\_elv}}$
$\theta_{\mathtt{sh\_rot}}$
$\theta_{\mathtt{el\_flex}}$
$\theta_{\mathtt{pro\_sup}}$
$\theta_{\mathtt{dev\_c}}$
$\theta_{\mathtt{flex\_c}}$
Original trajectory
OpenSim IK algorithm
The proposed algorithm (MATLAB implementation)

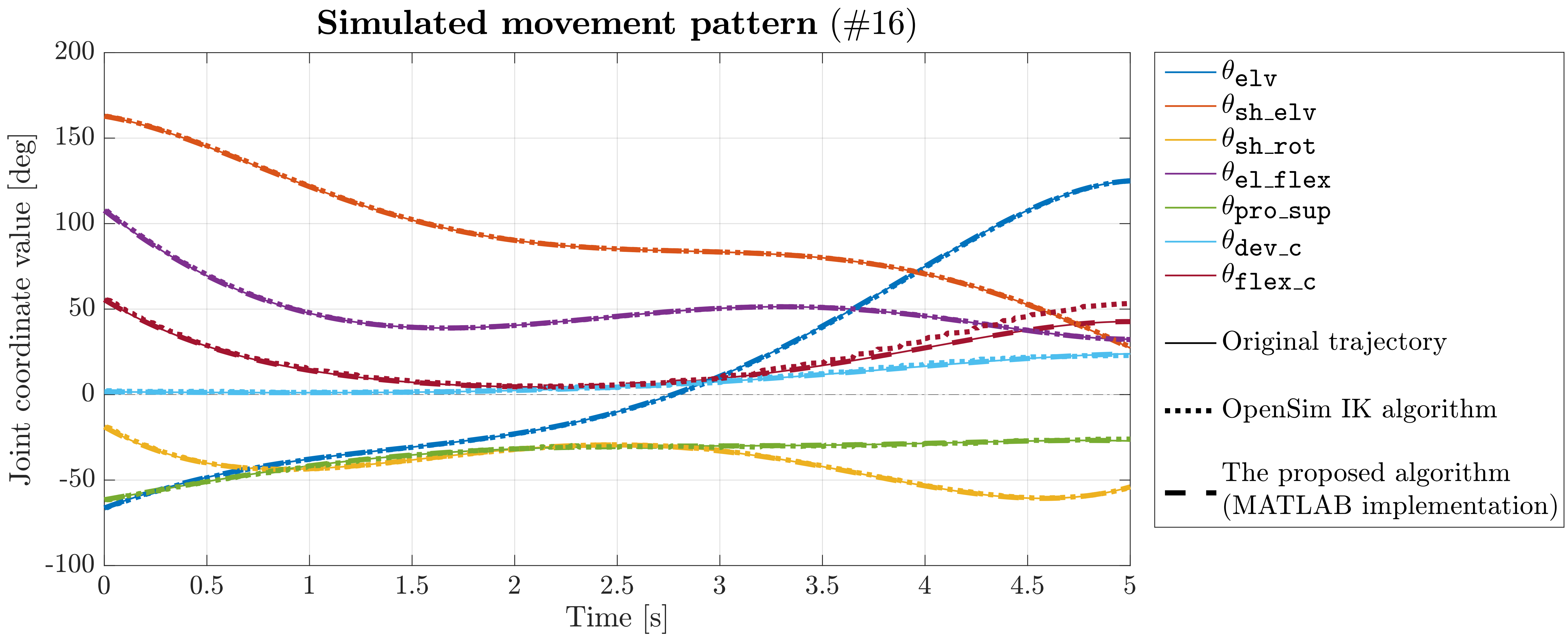

Simulated movement pattern (#16)
Joint coordinate value [deg]
Time [s]
200
150
100
50
0
-50
-100
0
0.5
1
1.5
2
2.5
3
3.5
4
4.5
5
$\theta_{\mathtt{elv}}$
$\theta_{\mathtt{sh\_elv}}$
$\theta_{\mathtt{sh\_rot}}$
$\theta_{\mathtt{el\_flex}}$
$\theta_{\mathtt{pro\_sup}}$
$\theta_{\mathtt{dev\_c}}$
$\theta_{\mathtt{flex\_c}}$
Original trajectory
OpenSim IK algorithm
The proposed algorithm
(MATLAB implementation)

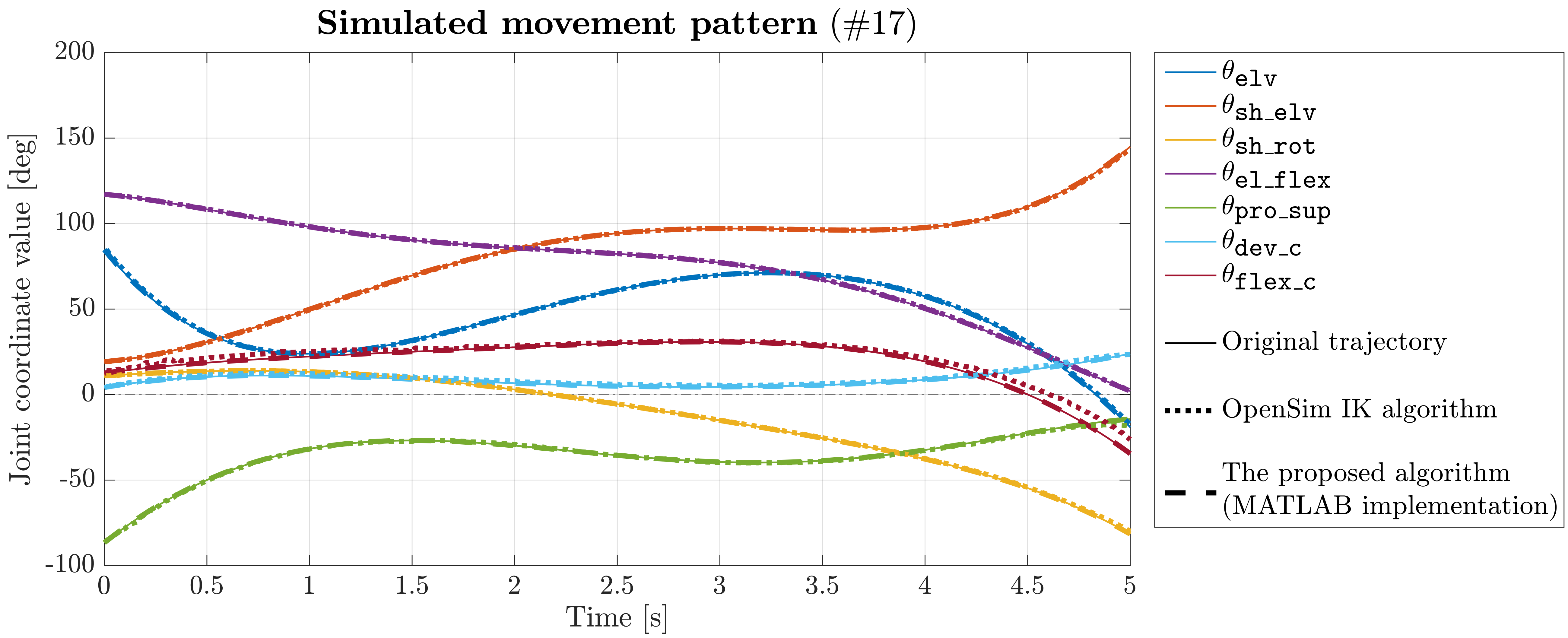

Simulated movement pattern (#17)
Joint coordinate value [deg]
200
150
100
50
0
-50
-100
0
0.5
1
1.5
2
2.5
3
3.5
4
4.5
5
Time [s]
θ_{elv}
θ_{sh_elv}
θ_{sh_rot}
θ_{el_flex}
θ_{pro_sup}
θ_{dev_c}
θ_{flex_c}
Original trajectory
OpenSim IK algorithm
The proposed algorithm (MATLAB implementation)

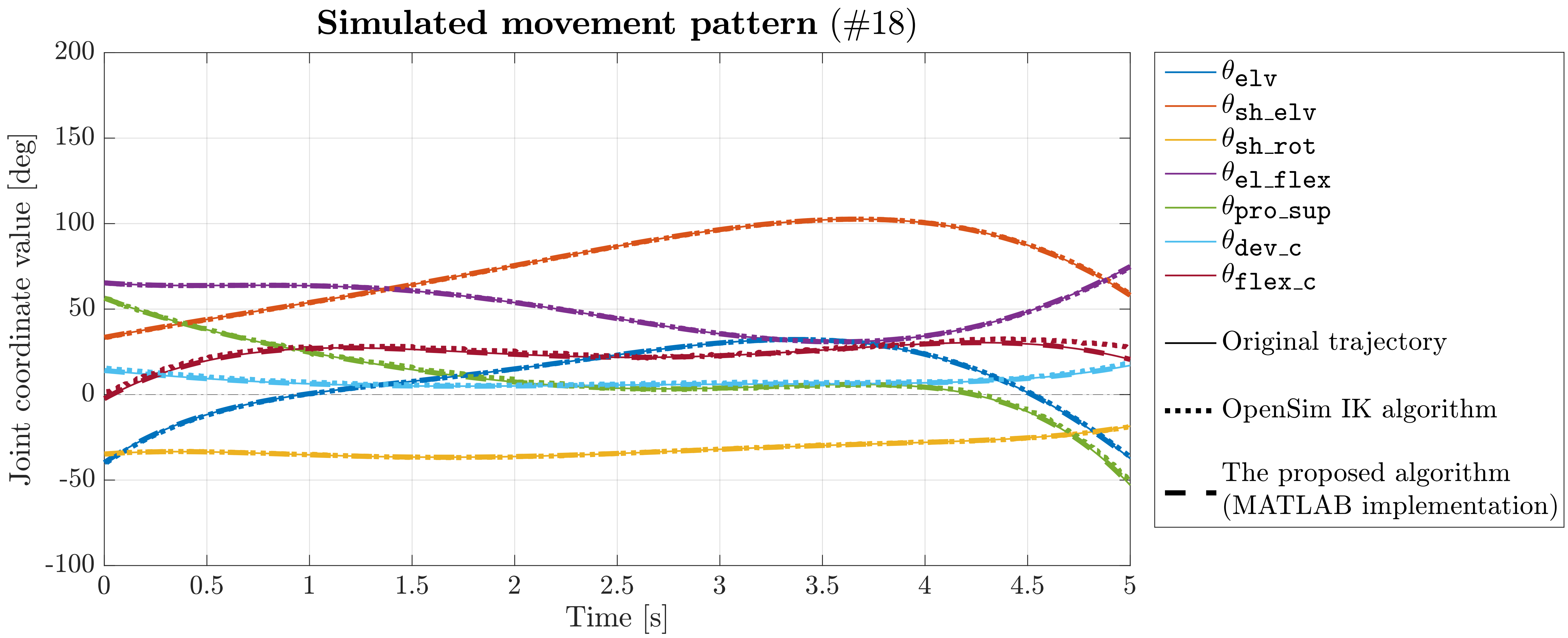

Simulated movement pattern (#18)
Joint coordinate value [deg]
200
150
100
50
0
-50
-100
0
0.5
1
1.5
2
2.5
3
3.5
4
4.5
5
Time [s]
$\theta_{\mathtt{elv}}$
$\theta_{\mathtt{sh\_elv}}$
$\theta_{\mathtt{sh\_rot}}$
$\theta_{\mathtt{el\_flex}}$
$\theta_{\mathtt{pro\_sup}}$
$\theta_{\mathtt{dev\_c}}$
$\theta_{\mathtt{flex\_c}}$
Original trajectory
OpenSim IK algorithm
The proposed algorithm
(MATLAB implementation)

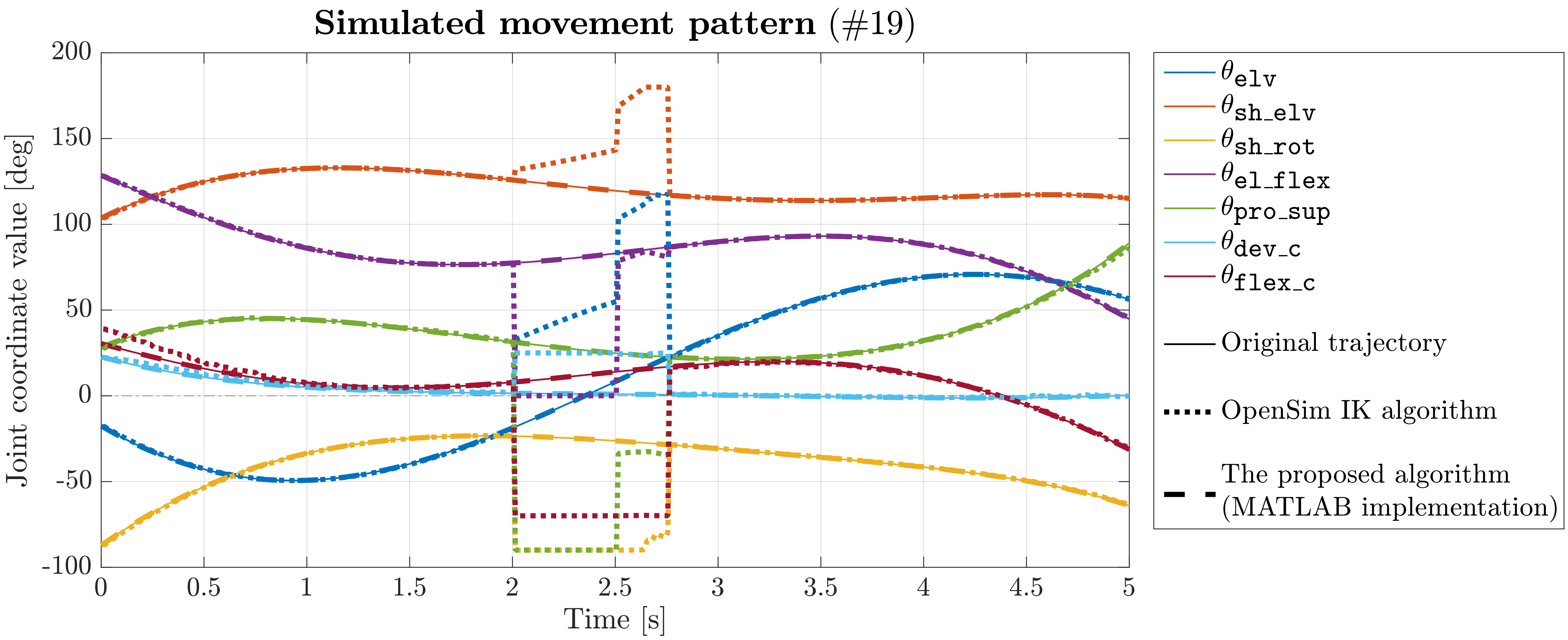

Simulated movement pattern (#19)
Joint coordinate value [deg]
200
150
100
50
0
-50
-100
0
0.5
1
1.5
2
2.5
3
3.5
4
4.5
5
Time [s]
θ_{elv}
θ_{sh_elv}
θ_{sh_rot}
θ_{el_flex}
θ_{pro_sup}
θ_{dev_c}
θ_{flex_c}
Original trajectory
OpenSim IK algorithm
The proposed algorithm (MATLAB implementation)

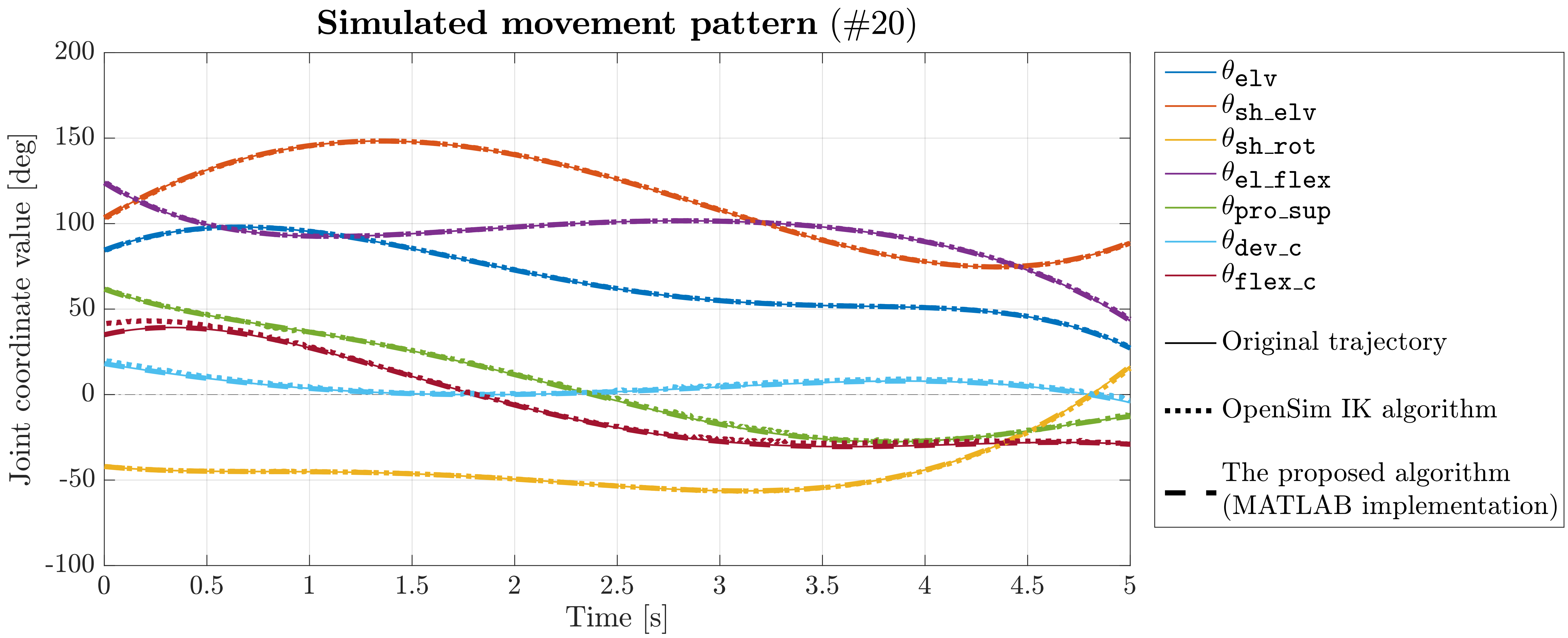

Simulated movement pattern (#20)
Joint coordinate value [deg]
200
150
100
50
0
-50
-100
0
0.5
1
1.5
2
2.5
3
3.5
4
4.5
5
Time [s]
θ_{elv}
θ_{sh_elv}
θ_{sh_rot}
θ_{el_flex}
θ_{pro_sup}
θ_{dev_c}
θ_{flex_c}
Original trajectory
OpenSim IK algorithm
The proposed algorithm
(MATLAB implementation)

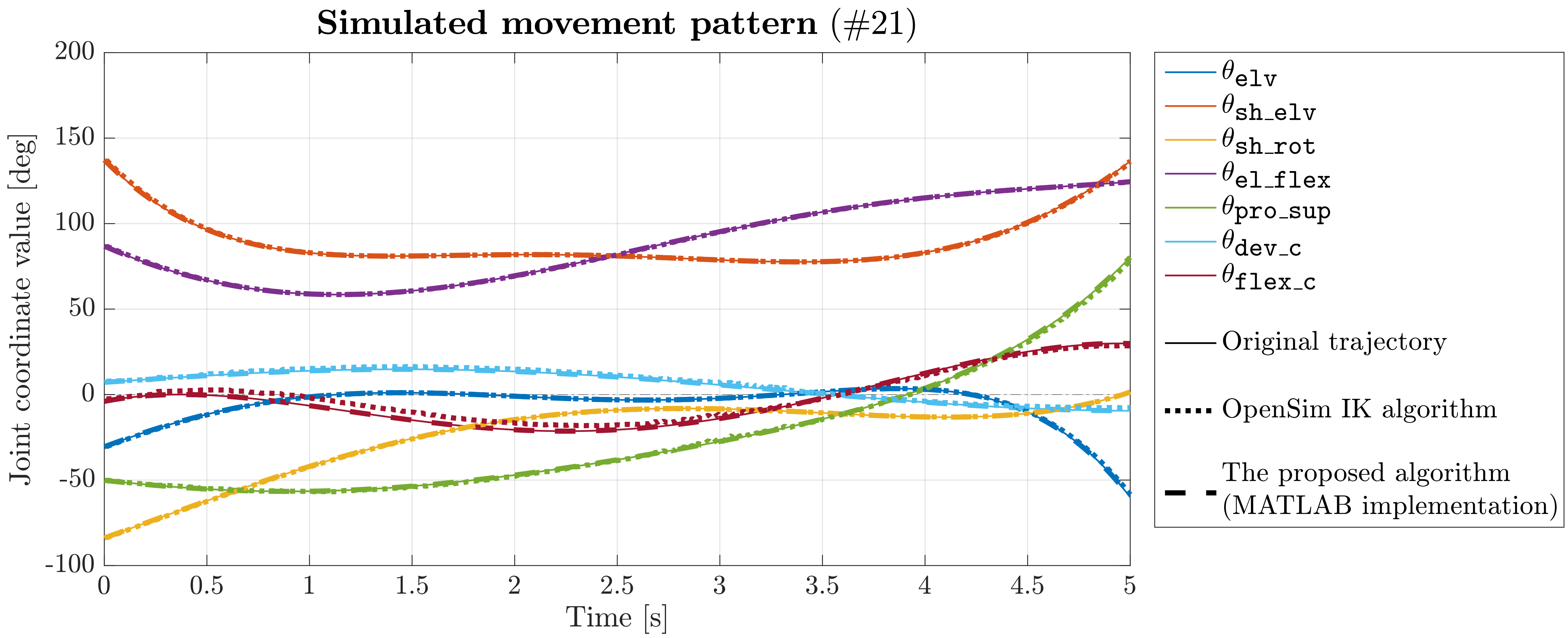

Simulated movement pattern (#21)
Joint coordinate value [deg]
200
150
100
50
0
-50
-100
0
0.5
1
1.5
2
2.5
3
3.5
4
4.5
5
Time [s]
$\theta_{\texttt{elv}}$
$\theta_{\texttt{sh\_elv}}$
$\theta_{\texttt{sh\_rot}}$
$\theta_{\texttt{el\_flex}}$
$\theta_{\texttt{pro\_sup}}$
$\theta_{\texttt{dev\_c}}$
$\theta_{\texttt{flex\_c}}$
Original trajectory
OpenSim IK algorithm
The proposed algorithm (MATLAB implementation)

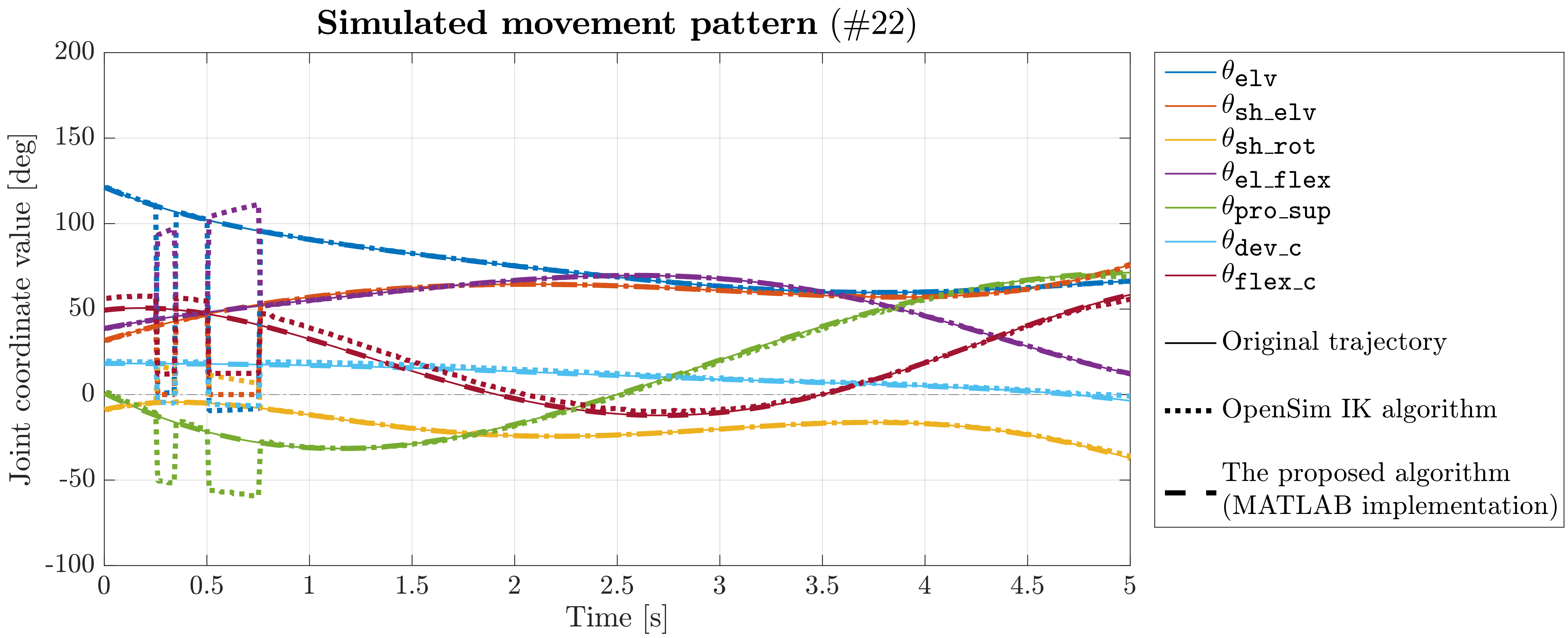

Simulated movement pattern (#22)
Joint coordinate value [deg]
200
150
100
50
0
-50
-100
0
0.5
1
1.5
2
2.5
3
3.5
4
4.5
5
Time [s]
θelv
θsh_elv
θsh_rot
θel_flex
θpro_sup
θdev_c
θflex_c
Original trajectory
OpenSim IK algorithm
The proposed algorithm (MATLAB implementation)

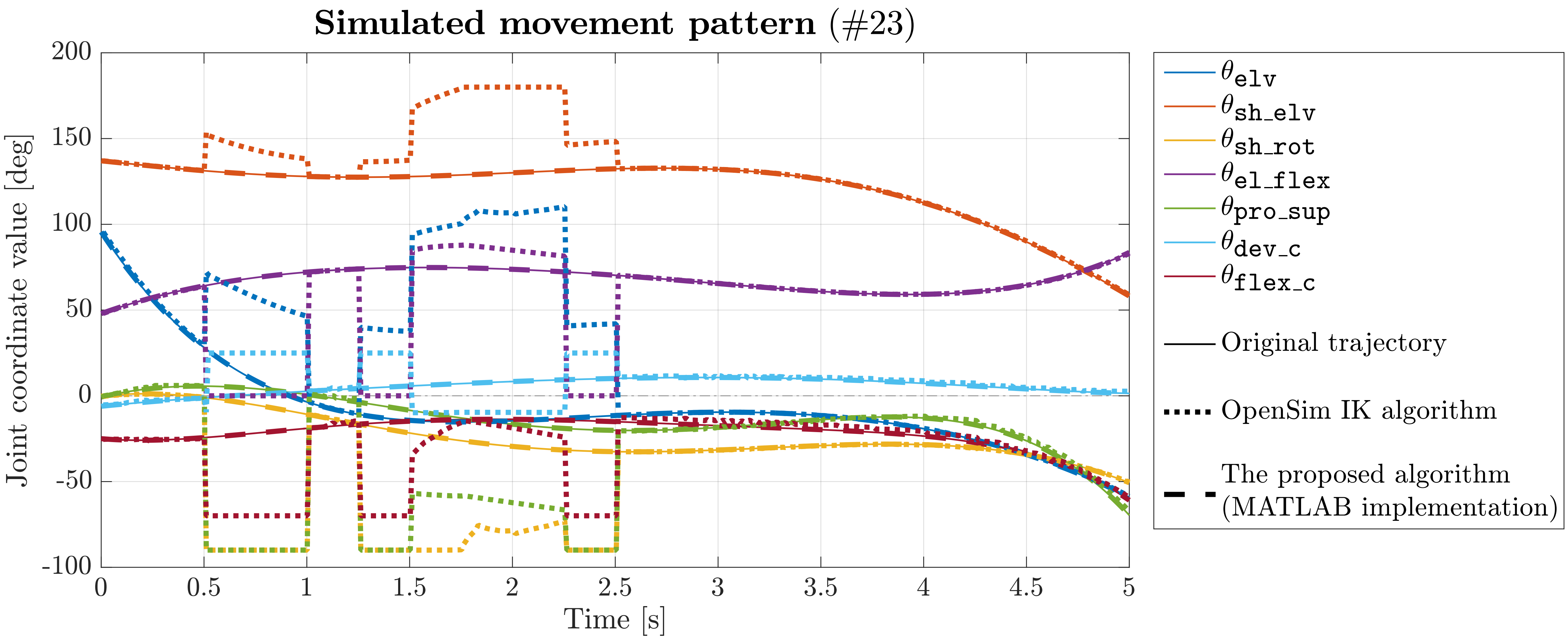

Simulated movement pattern (#23)
Joint coordinate value [deg]
Time [s]
200
150
100
50
0
-50
-100
0
0.5
1
1.5
2
2.5
3
3.5
4
4.5
5
$\theta_{\mathtt{elv}}$
$\theta_{\mathtt{sh\_elv}}$
$\theta_{\mathtt{sh\_rot}}$
$\theta_{\mathtt{el\_flex}}$
$\theta_{\mathtt{pro\_sup}}$
$\theta_{\mathtt{dev\_c}}$
$\theta_{\mathtt{flex\_c}}$
Original trajectory
OpenSim IK algorithm
The proposed algorithm
(MATLAB implementation)

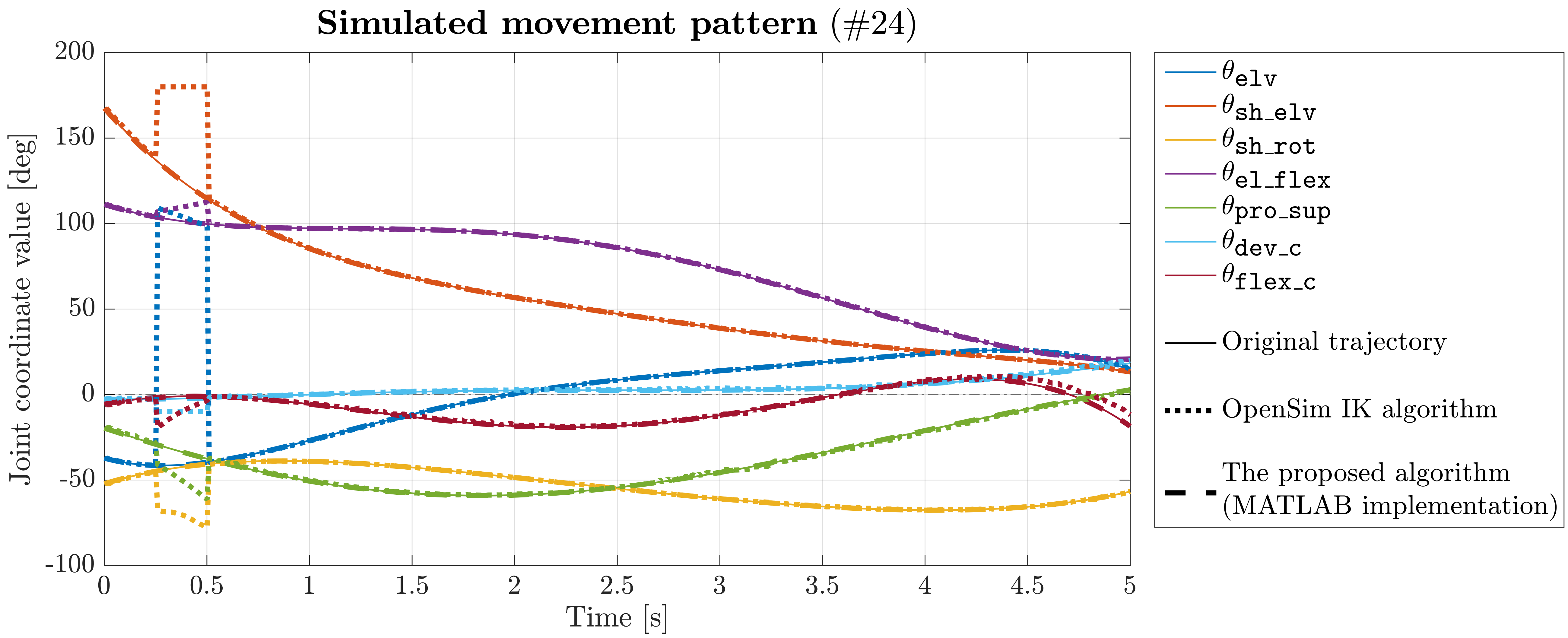

Simulated movement pattern (#24)
Joint coordinate value [deg]
Time [s]
200
150
100
50
0
-50
-100
0
0.5
1
1.5
2
2.5
3
3.5
4
4.5
5
$\theta_{\texttt{elv}}$
$\theta_{\texttt{sh\_elv}}$
$\theta_{\texttt{sh\_rot}}$
$\theta_{\texttt{el\_flex}}$
$\theta_{\texttt{pro\_sup}}$
$\theta_{\texttt{dev\_c}}$
$\theta_{\texttt{flex\_c}}$
Original trajectory
OpenSim IK algorithm
The proposed algorithm
(MATLAB implementation)

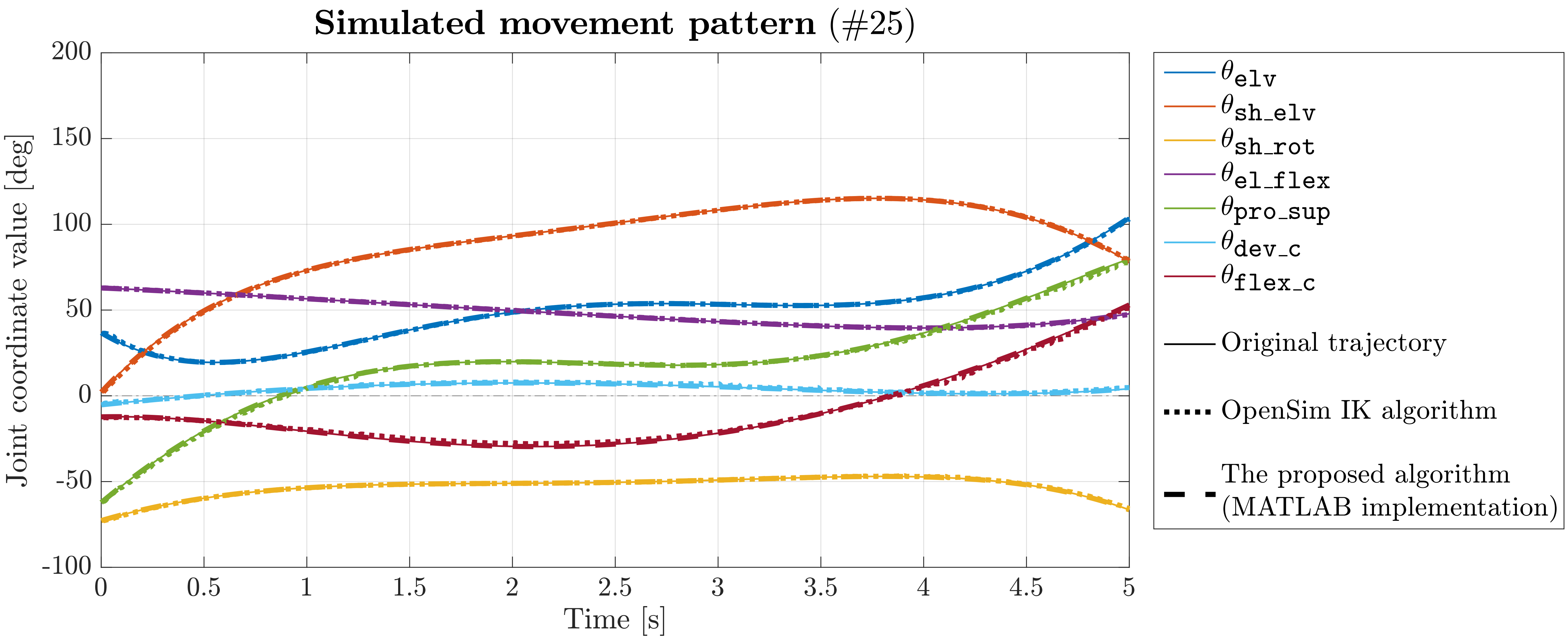

Simulated movement pattern (#25)
Joint coordinate value [deg]
Time [s]
200
150
100
50
0
-50
-100
0
0.5
1
1.5
2
2.5
3
3.5
4
4.5
5
θelv
θsh_elv
θsh_rot
θel_flex
θpro_sup
θdev_c
θflex_c
Original trajectory
OpenSim IK algorithm
The proposed algorithm (MATLAB implementation)

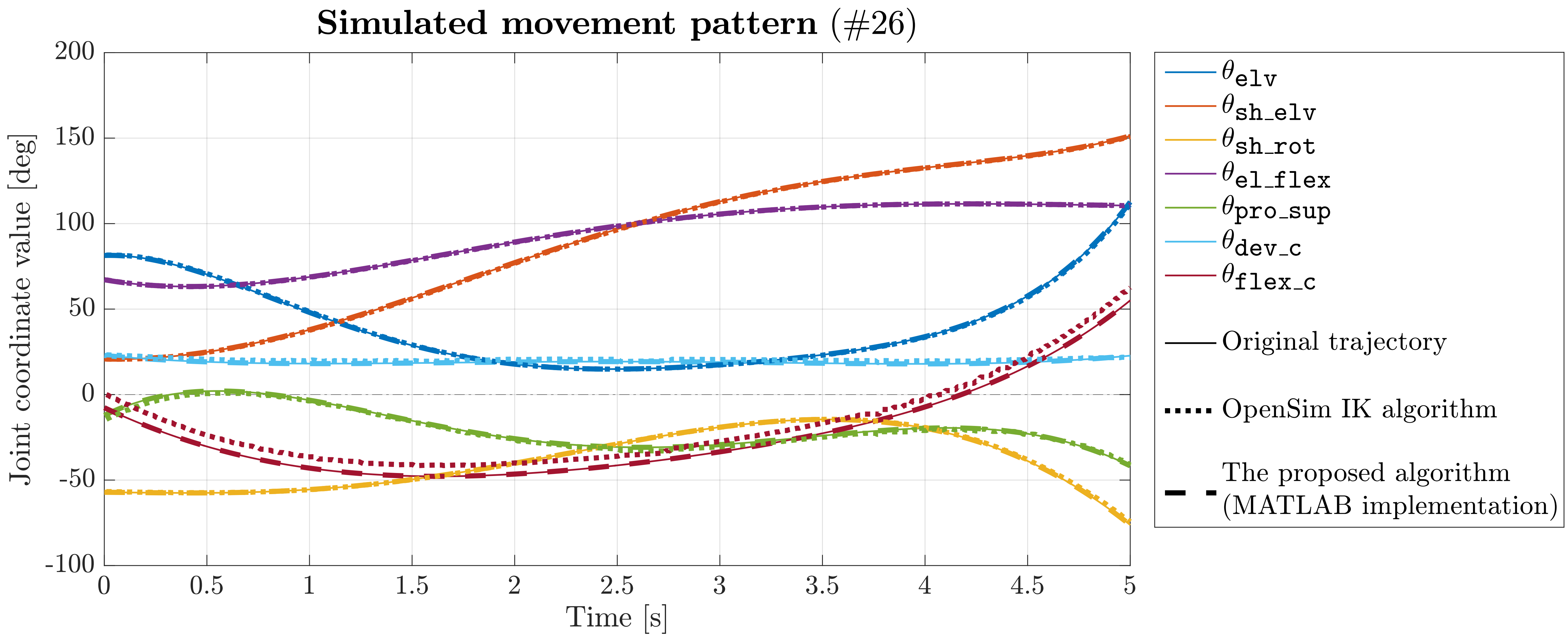
Simulated movement pattern (#26)
200
150
100
50
0
-50
-100
Joint coordinate value [deg]
0
0.5
1
1.5
2
2.5
3
3.5
4
4.5
5
Time [s]
θ_{elv}
θ_{sh_elv}
θ_{sh_rot}
θ_{el_flex}
θ_{pro_sup}
θ_{dev_c}
θ_{flex_c}
Original trajectory
OpenSim IK algorithm
The proposed algorithm (MATLAB implementation)

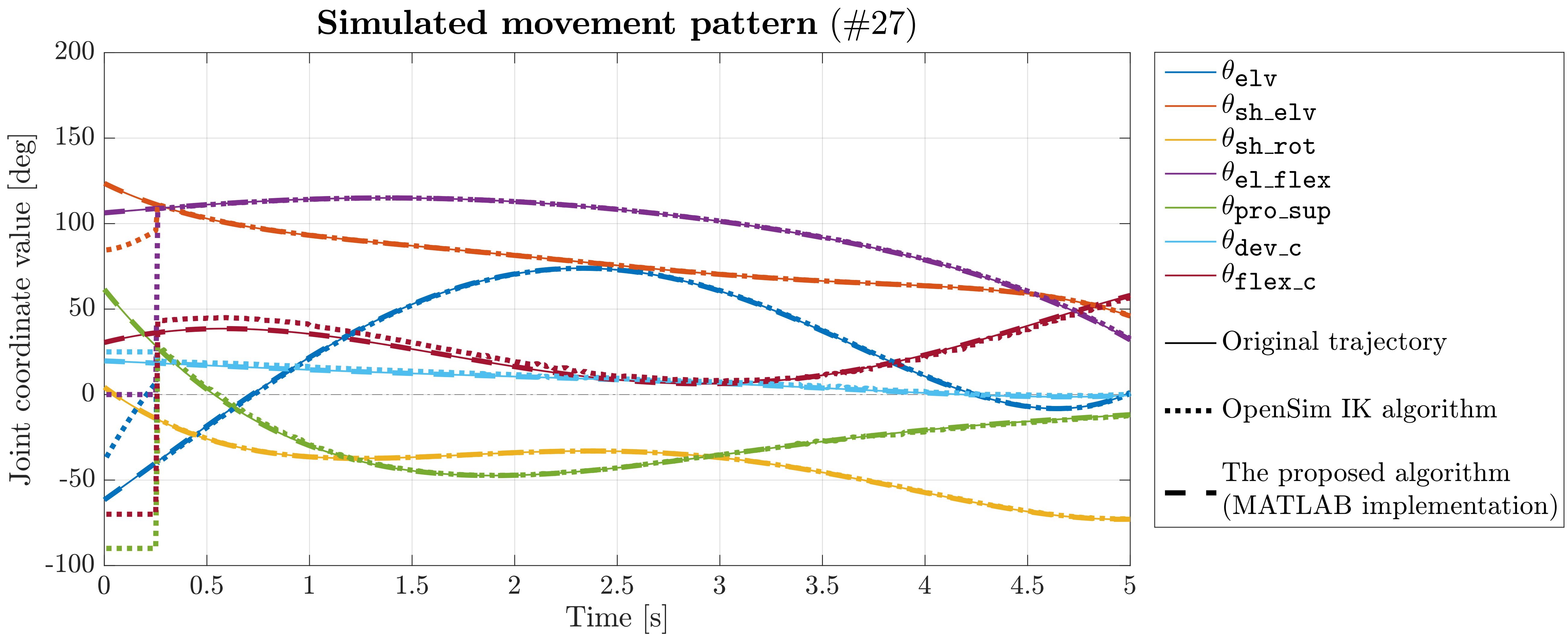

Simulated movement pattern (#27)
200
150
100
50
0
-50
-100
Joint coordinate value [deg]
0
0.5
1
1.5
2
2.5
3
3.5
4
4.5
5
Time [s]
θelv
θsh_elv
θsh_rot
θel_flex
θpro_sup
θdev_c
θflex_c
Original trajectory
OpenSim IK algorithm
The proposed algorithm
(MATLAB implementation)

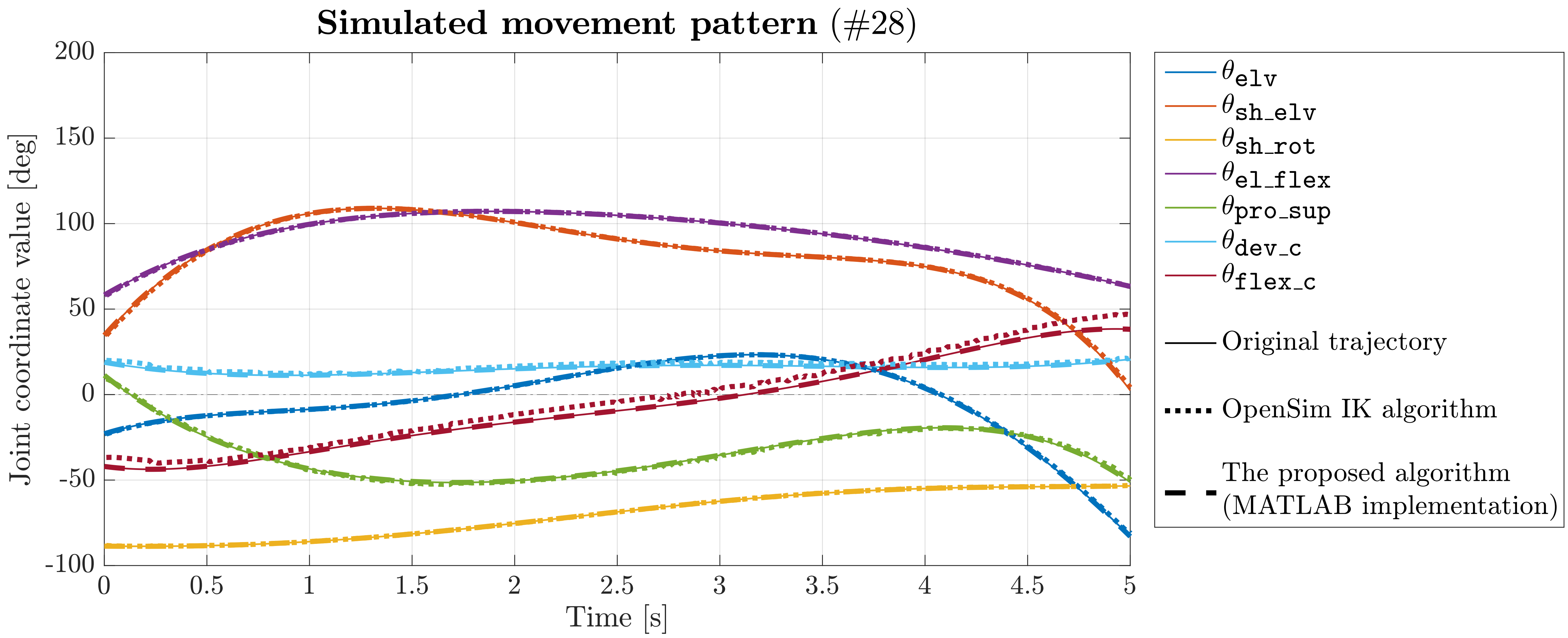

Simulated movement pattern (#28)
Joint coordinate value [deg]
200
150
100
50
0
-50
-100
0
0.5
1
1.5
2
2.5
3
3.5
4
4.5
5
Time [s]
θelv
θsh_elv
θsh_rot
θel_flex
θpro_sup
θdev_c
θflex_c
Original trajectory
OpenSim IK algorithm
The proposed algorithm
(MATLAB implementation)

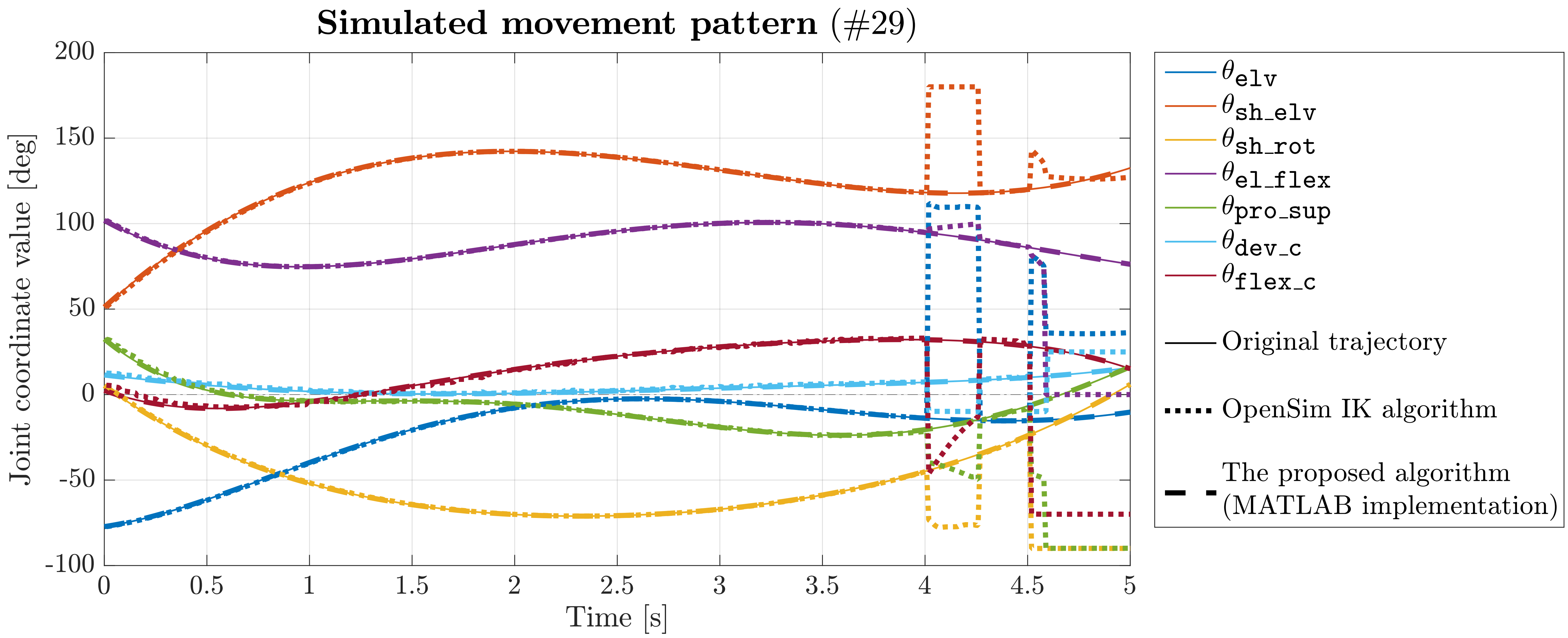
Simulated movement pattern (#29)
Joint coordinate value [deg]
Time [s]
200
150
100
50
0
-50
-100
0
0.5
1
1.5
2
2.5
3
3.5
4
4.5
5
θelv
θsh_elv
θsh_rot
θel_flex
θpro_sup
θdev_c
θflex_c
Original trajectory
OpenSim IK algorithm
The proposed algorithm
(MATLAB implementation)

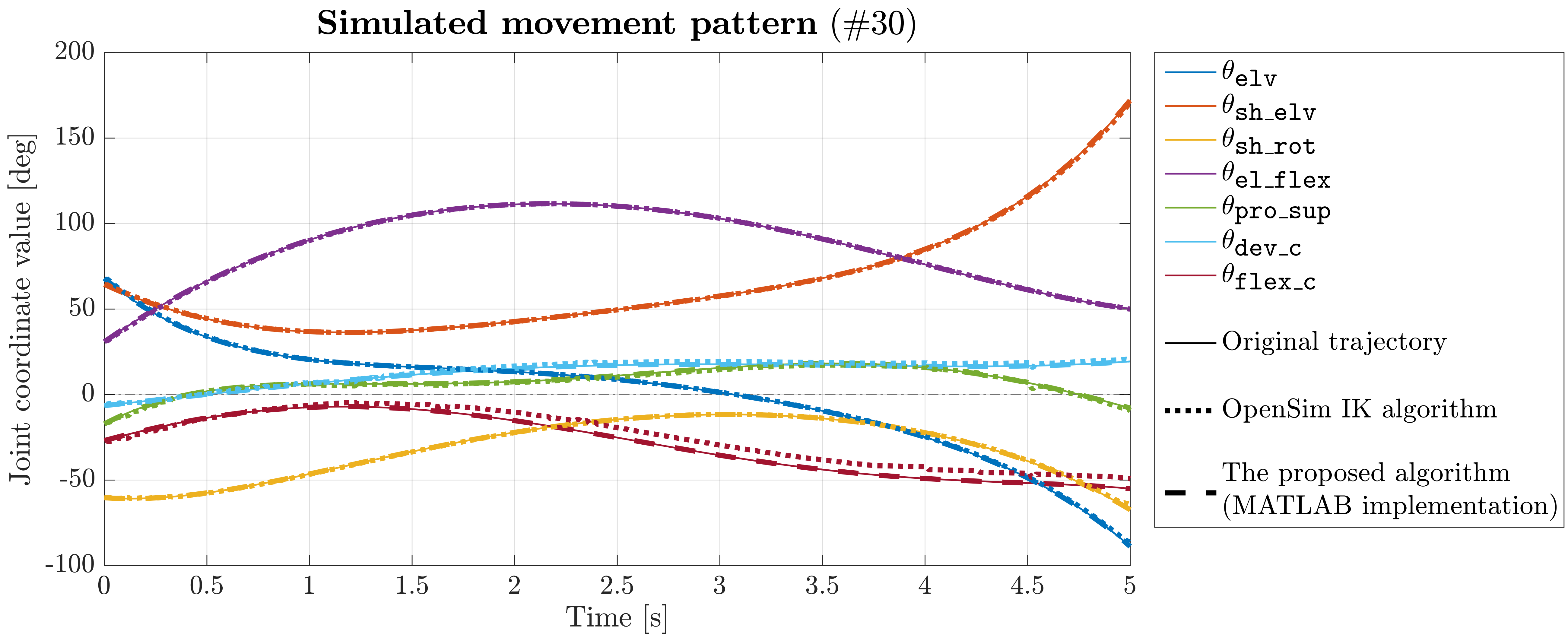

Simulated movement pattern (#30)
Joint coordinate value [deg]
Time [s]
200
150
100
50
0
-50
-100
0
0.5
1
1.5
2
2.5
3
3.5
4
4.5
5
θelv
θsh_elv
θsh_rot
θel_flex
θpro_sup
θdev_c
θflex_c
Original trajectory
OpenSim IK algorithm
The proposed algorithm (MATLAB implementation)

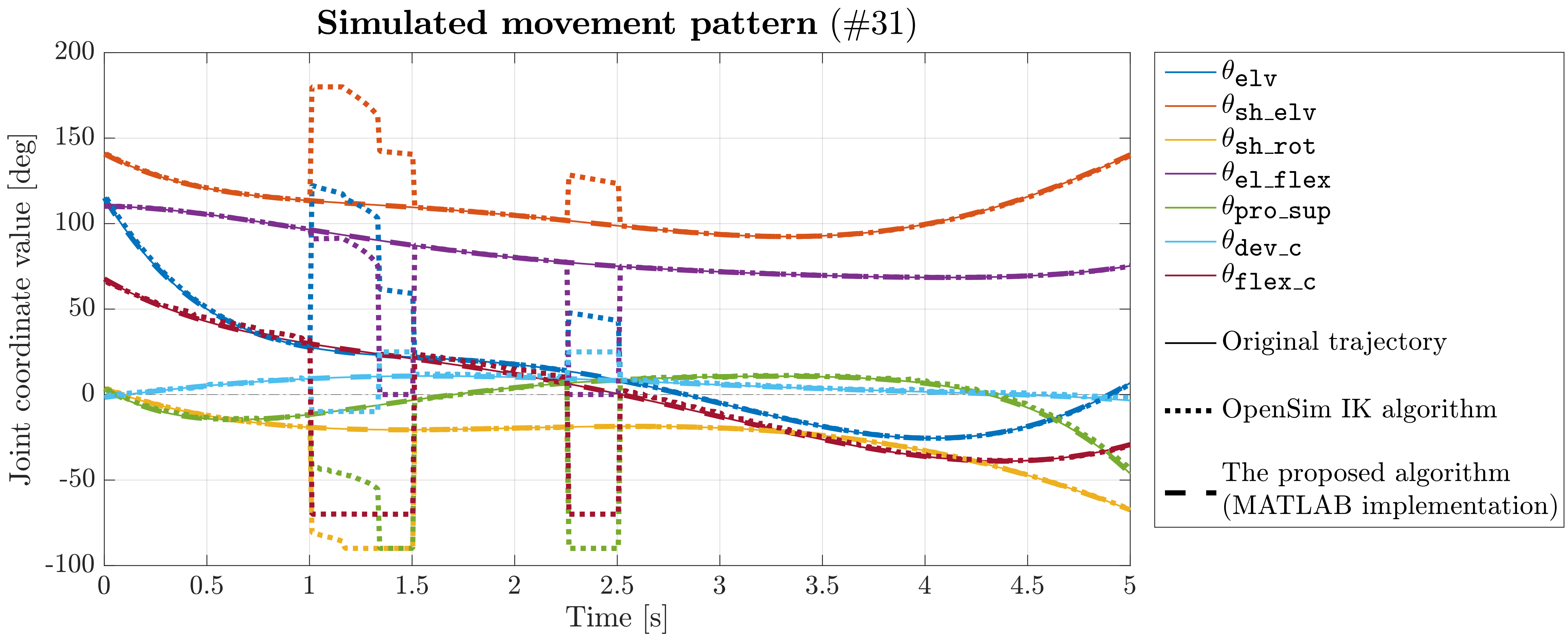

Simulated movement pattern (#31)
200
150
100
50
0
-50
-100
Joint coordinate value [deg]
0
0.5
1
1.5
2
2.5
3
3.5
4
4.5
5
Time [s]
θelv
θsh_elv
θsh_rot
θel_flex
θpro_sup
θdev_c
θflex_c
Original trajectory
OpenSim IK algorithm
The proposed algorithm (MATLAB implementation)

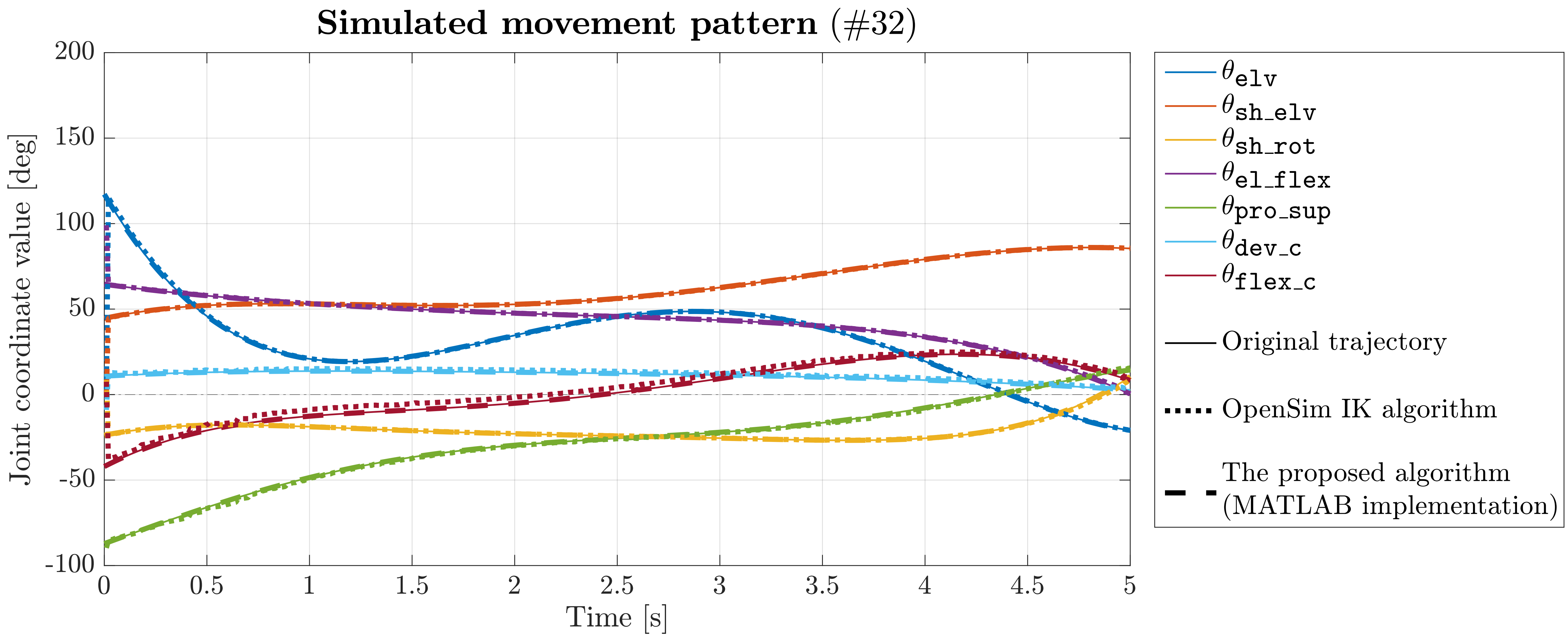
Simulated movement pattern (#32)
200
150
100
50
0
-50
-100
Joint coordinate value [deg]
0
0.5
1
1.5
2
2.5
3
3.5
4
4.5
5
Time [s]
$\theta_{\texttt{elv}}$
$\theta_{\texttt{sh\_elv}}$
$\theta_{\texttt{sh\_rot}}$
$\theta_{\texttt{el\_flex}}$
$\theta_{\texttt{pro\_sup}}$
$\theta_{\texttt{dev\_c}}$
$\theta_{\texttt{flex\_c}}$
Original trajectory
OpenSim IK algorithm
The proposed algorithm
(MATLAB implementation)

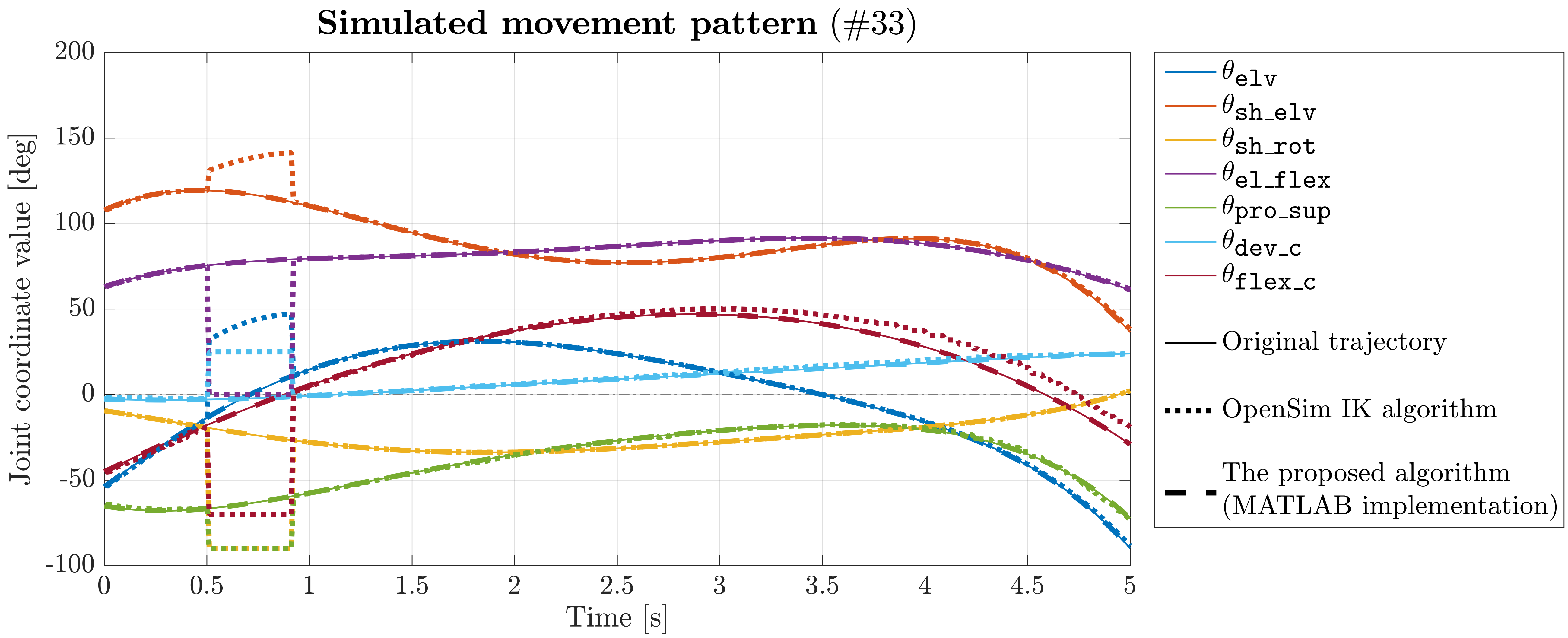

Simulated movement pattern (#33)
Joint coordinate value [deg]
Time [s]
200
150
100
50
0
-50
-100
0
0.5
1
1.5
2
2.5
3
3.5
4
4.5
5
θelv
θsh_elv
θsh_rot
θel_flex
θpro_sup
θdev_c
θflex_c
Original trajectory
OpenSim IK algorithm
The proposed algorithm
(MATLAB implementation)

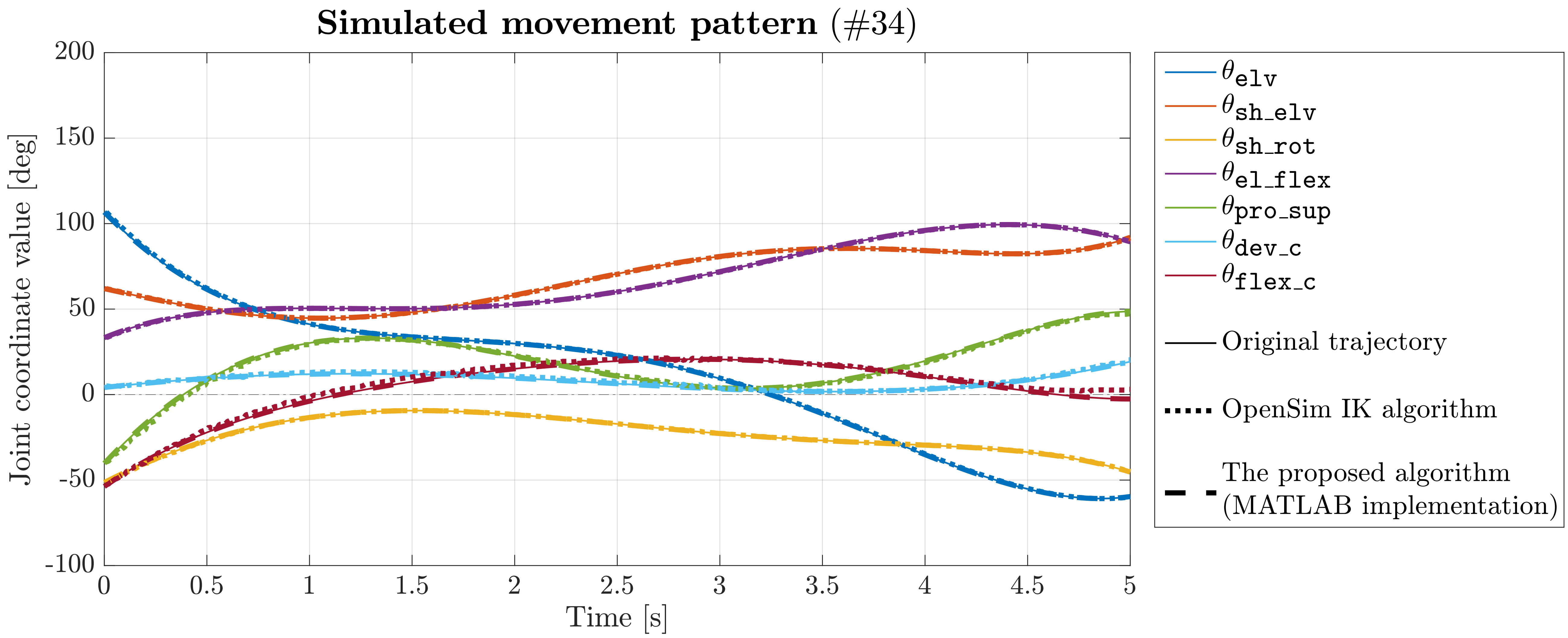

Simulated movement pattern (#34)
Joint coordinate value [deg]
200
150
100
50
0
-50
-100
0
0.5
1
1.5
2
2.5
3
3.5
4
4.5
5
Time [s]
$\theta_{\mathtt{elv}}$
$\theta_{\mathtt{sh\_elv}}$
$\theta_{\mathtt{sh\_rot}}$
$\theta_{\mathtt{el\_flex}}$
$\theta_{\mathtt{pro\_sup}}$
$\theta_{\mathtt{dev\_c}}$
$\theta_{\mathtt{flex\_c}}$
Original trajectory
OpenSim IK algorithm
The proposed algorithm
(MATLAB implementation)

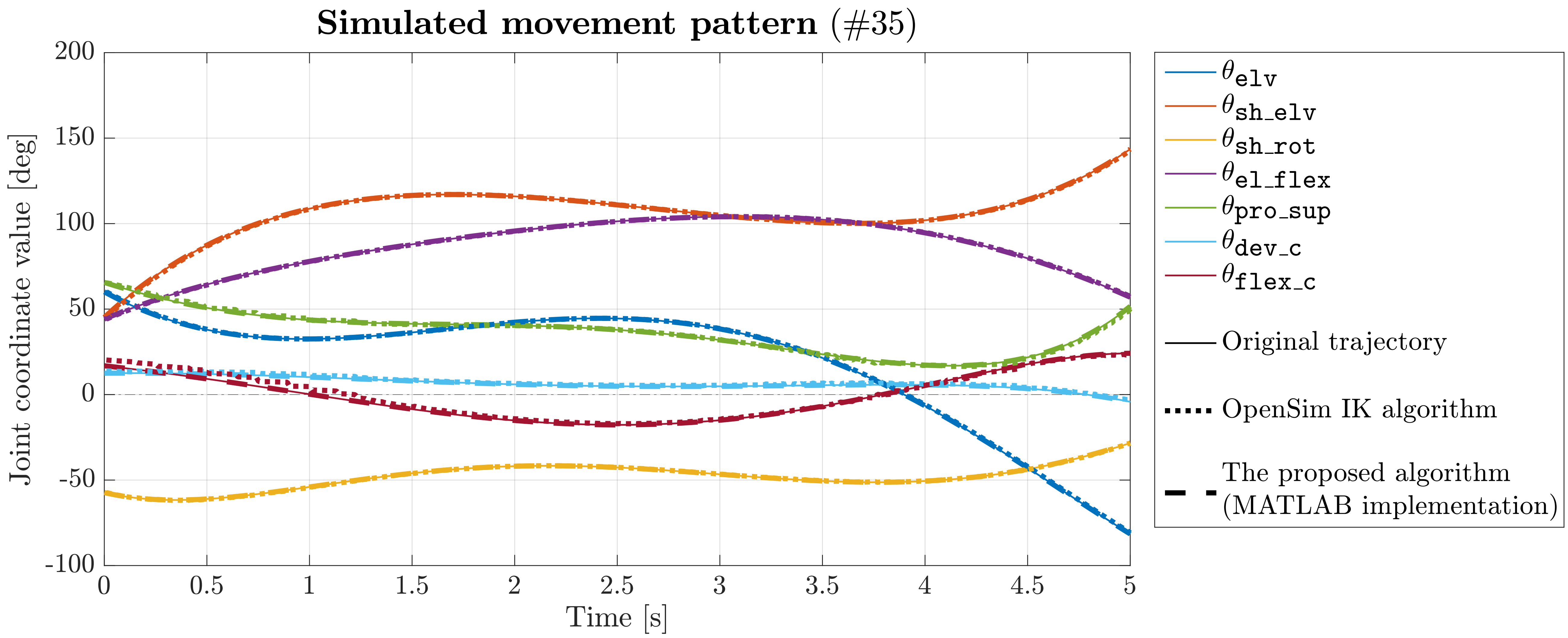

Simulated movement pattern (#35)
Joint coordinate value [deg]
Time [s]
200
150
100
50
0
-50
-100
0
0.5
1
1.5
2
2.5
3
3.5
4
4.5
5
θ_{elv}
θ_{sh_elv}
θ_{sh_rot}
θ_{el_flex}
θ_{pro_sup}
θ_{dev_c}
θ_{flex_c}
Original trajectory
OpenSim IK algorithm
The proposed algorithm
(MATLAB implementation)

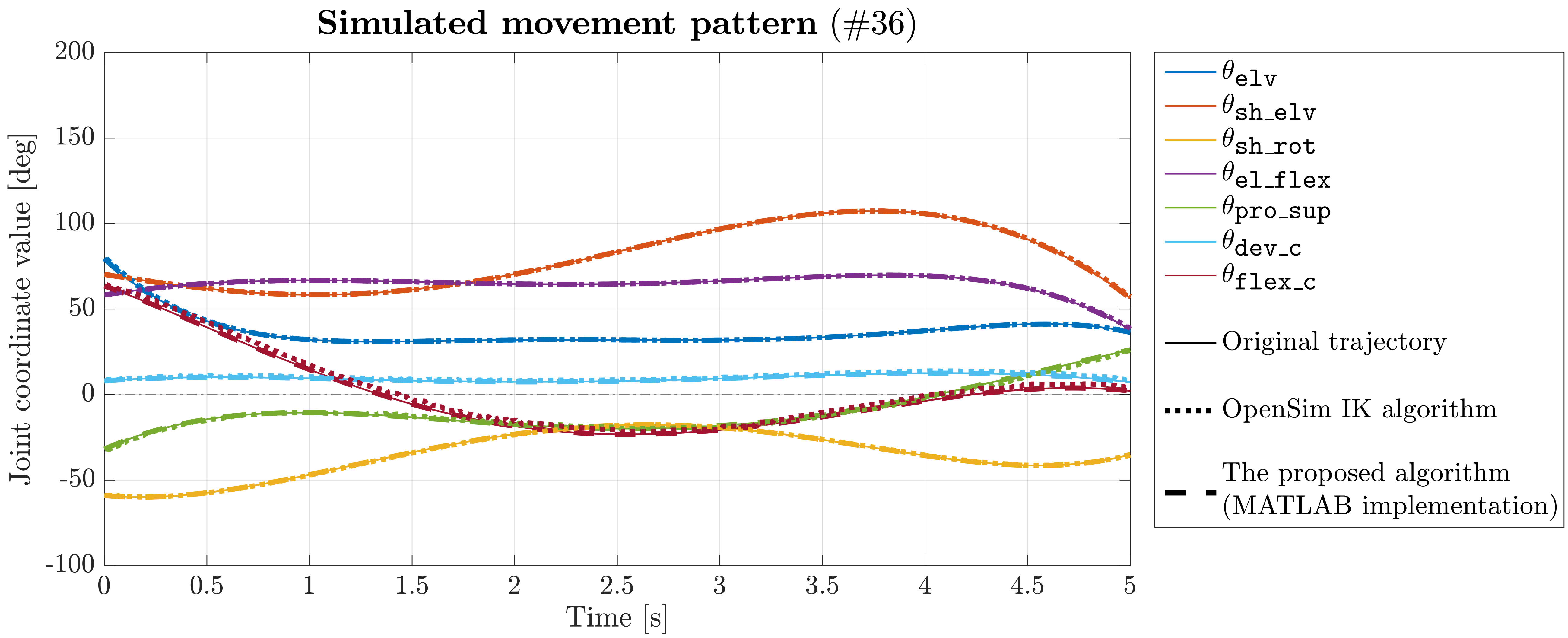

Simulated movement pattern (#36)
Joint coordinate value [deg]
200
150
100
50
0
-50
-100
0
0.5
1
1.5
2
2.5
3
3.5
4
4.5
5
Time [s]
θelv
θsh_elv
θsh_rot
θel_flex
θpro_sup
θdev_c
θflex_c
Original trajectory
OpenSim IK algorithm
The proposed algorithm
(MATLAB implementation)

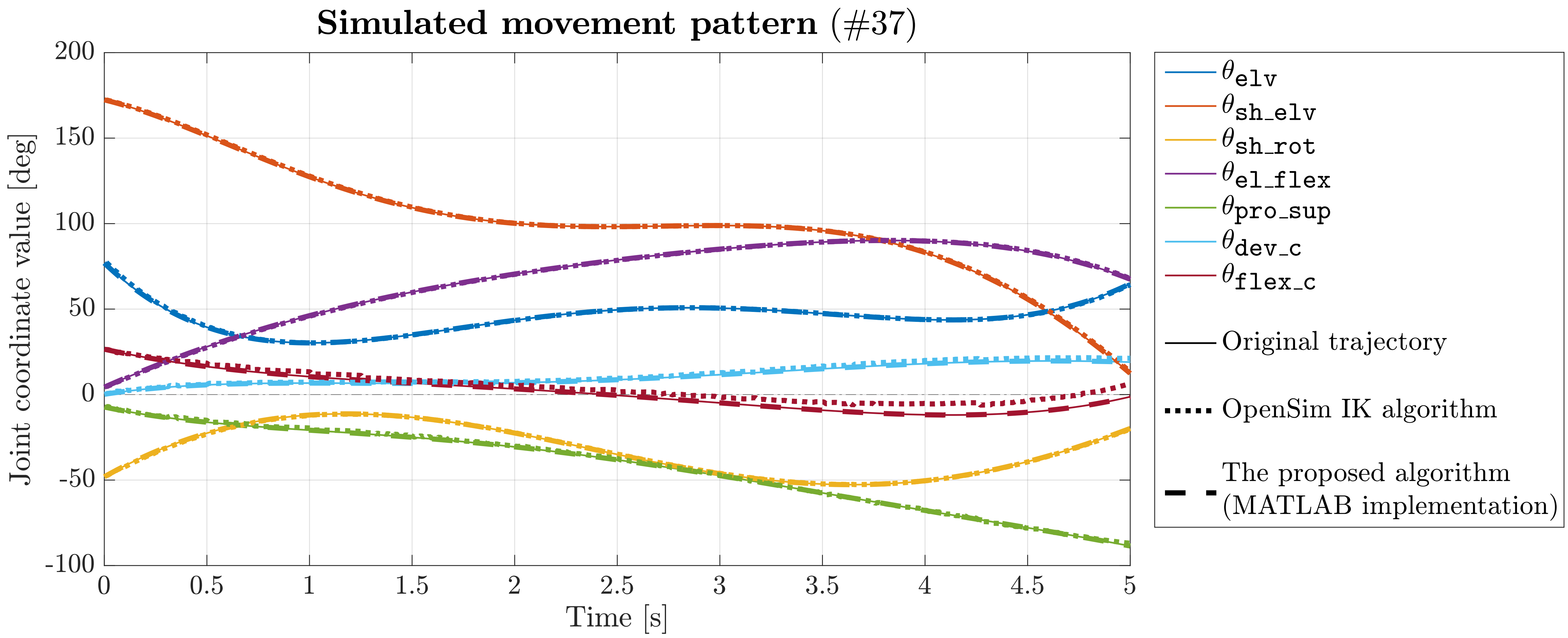

Simulated movement pattern (#37)
Joint coordinate value [deg]
Time [s]
200
150
100
50
0
-50
-100
0
0.5
1
1.5
2
2.5
3
3.5
4
4.5
5
θ_{elv}
θ_{sh_elv}
θ_{sh_rot}
θ_{el_flex}
θ_{pro_sup}
θ_{dev_c}
θ_{flex_c}
Original trajectory
OpenSim IK algorithm
The proposed algorithm (MATLAB implementation)

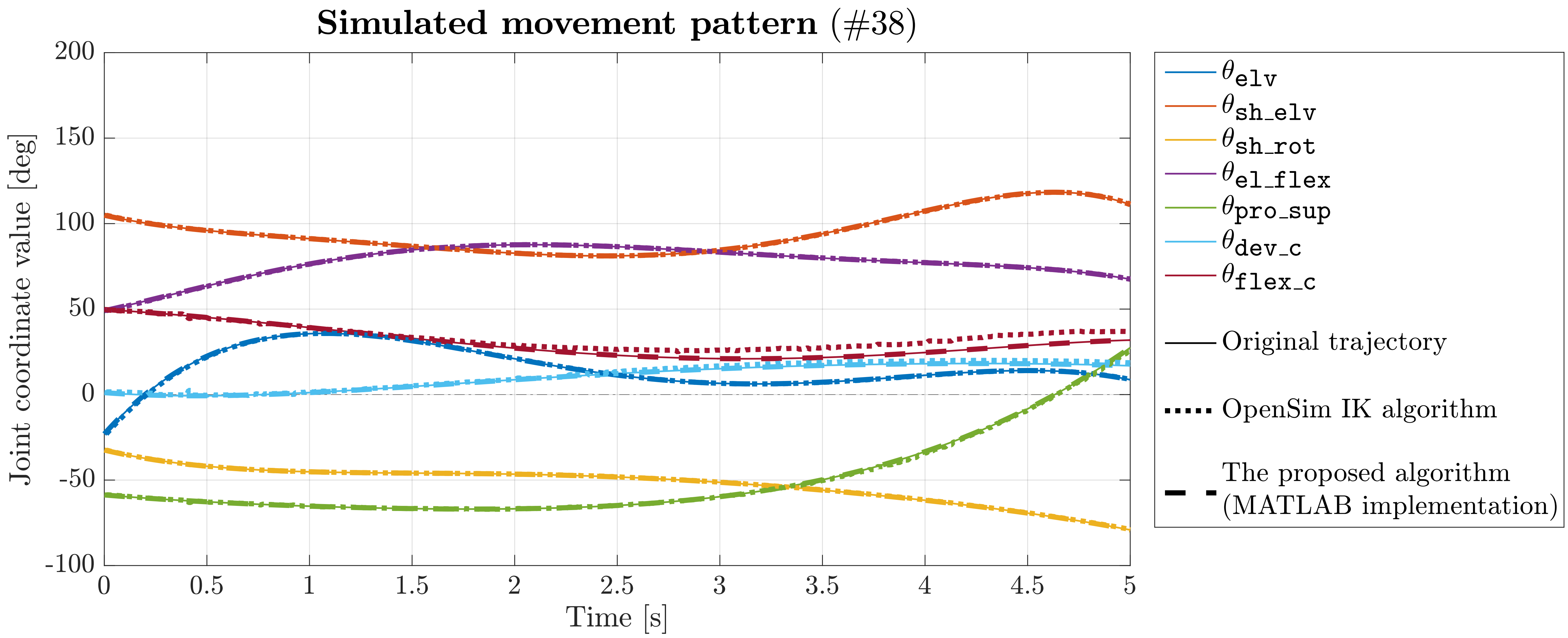

Simulated movement pattern (#38)
Joint coordinate value [deg]
200
150
100
50
0
-50
-100
0
0.5
1
1.5
2
2.5
3
3.5
4
4.5
5
Time [s]
θelv
θsh_elv
θsh_rot
θel_flex
θpro_sup
θdev_c
θflex_c
Original trajectory
OpenSim IK algorithm
The proposed algorithm (MATLAB implementation)

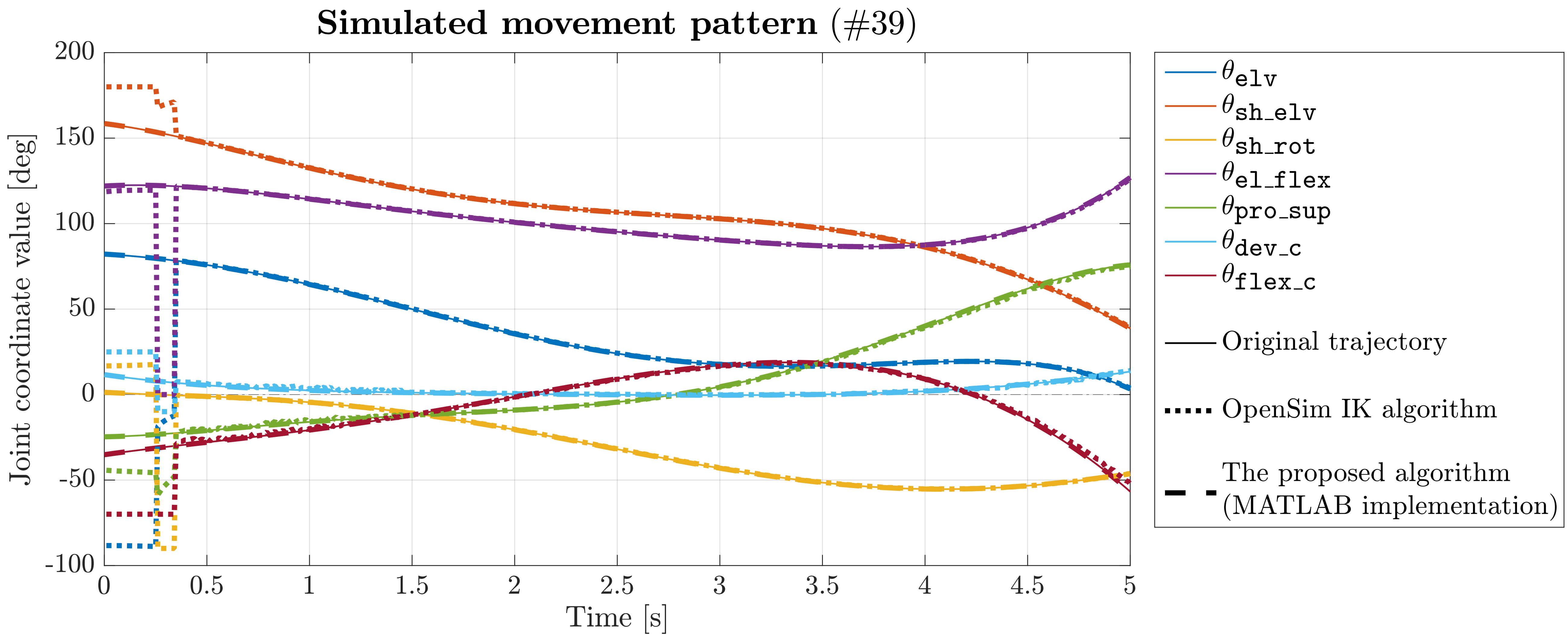

Simulated movement pattern (#39)
Joint coordinate value [deg]
200
150
100
50
0
-50
-100
0
0.5
1
1.5
2
2.5
3
3.5
4
4.5
5
Time [s]
θelv
θsh_elv
θsh_rot
θel_flex
θpro_sup
θdev_c
θflex_c
Original trajectory
OpenSim IK algorithm
The proposed algorithm (MATLAB implementation)

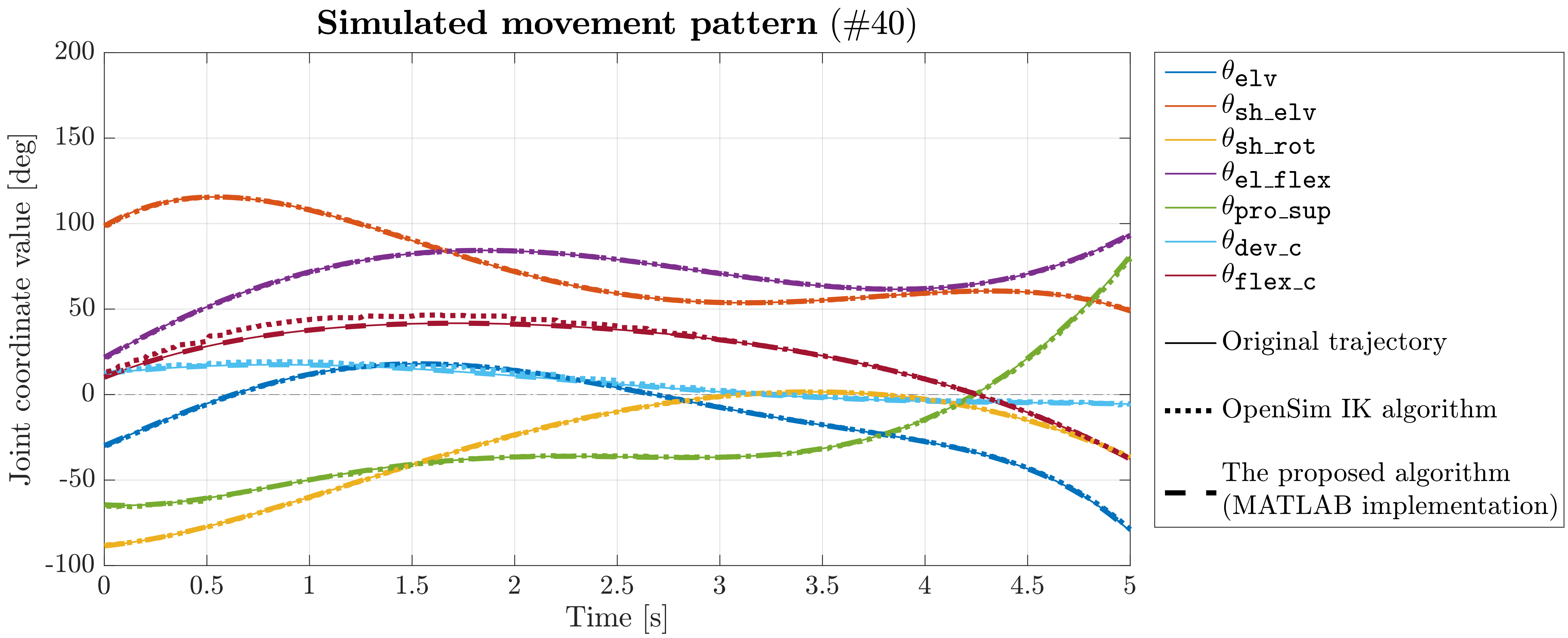
Simulated movement pattern (#40)
Joint coordinate value [deg]
Time [s]
200
150
100
50
0
-50
-100
0
0.5
1
1.5
2
2.5
3
3.5
4
4.5
5
θelv
θsh_elv
θsh_rot
θel_flex
θpro_sup
θdev_c
θflex_c
Original trajectory
OpenSim IK algorithm
The proposed algorithm (MATLAB implementation)

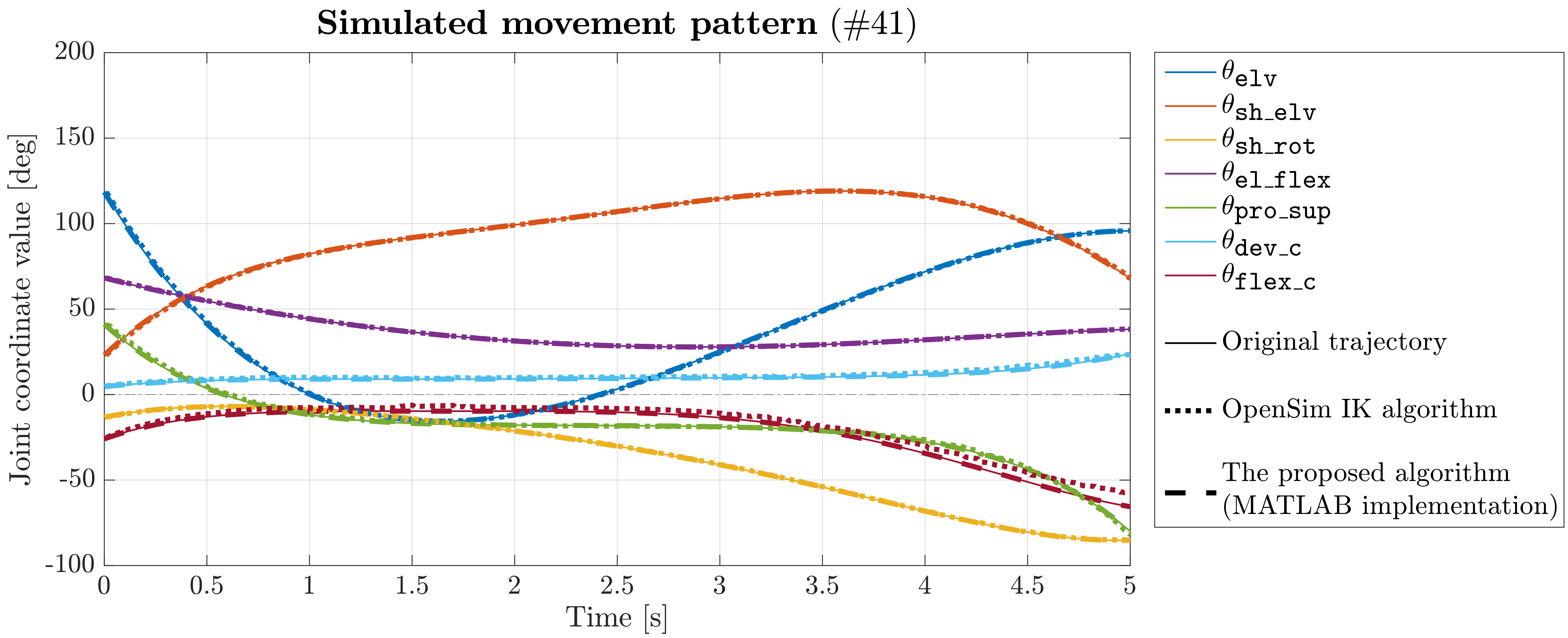

Simulated movement pattern (#41)
Joint coordinate value [deg]
200
150
100
50
0
-50
-100
0
0.5
1
1.5
2
2.5
3
3.5
4
4.5
5
Time [s]
θelv
θsh_elv
θsh_rot
θel_flex
θpro_sup
θdev_c
θflex_c
Original trajectory
OpenSim IK algorithm
The proposed algorithm
(MATLAB implementation)

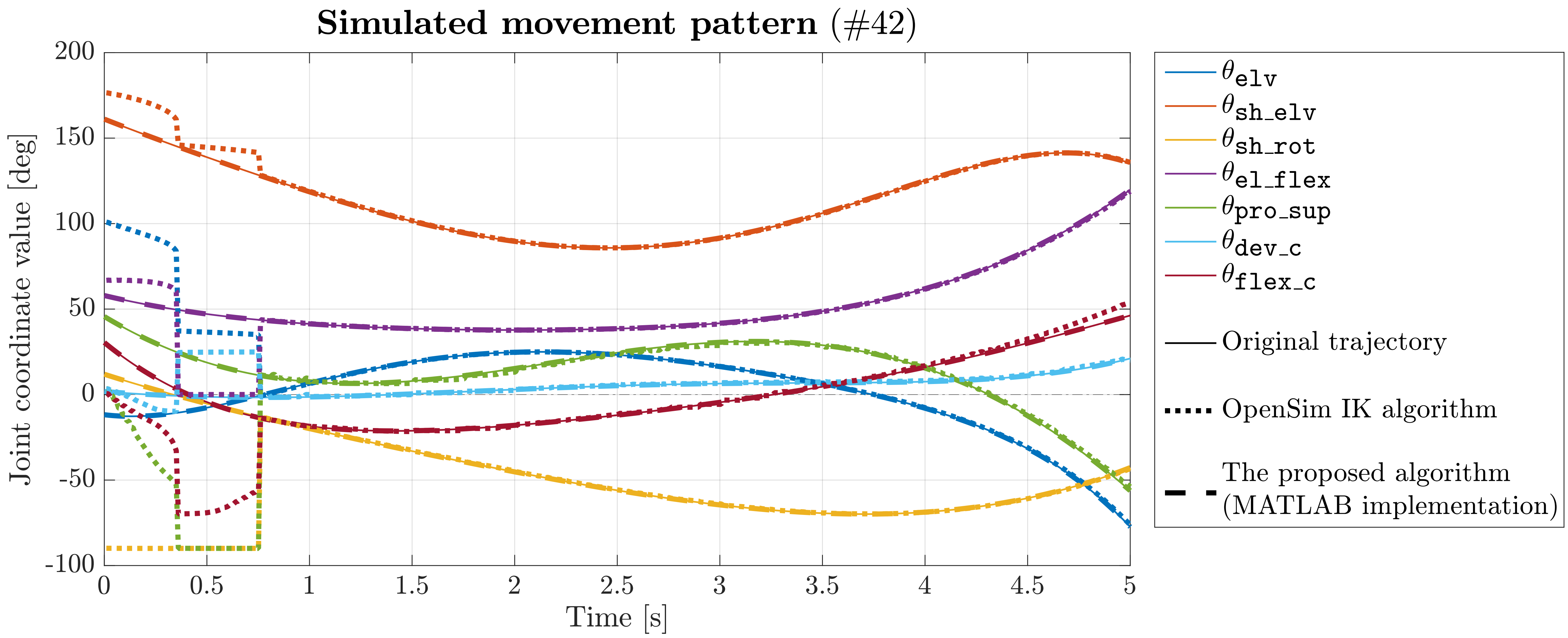

Simulated movement pattern (#42)
Joint coordinate value [deg]
Time [s]
200
150
100
50
0
-50
-100
0
0.5
1
1.5
2
2.5
3
3.5
4
4.5
5
θ_{elv}
θ_{sh_elv}
θ_{sh_rot}
θ_{el_flex}
θ_{pro_sup}
θ_{dev_c}
θ_{flex_c}
Original trajectory
OpenSim IK algorithm
The proposed algorithm (MATLAB implementation)

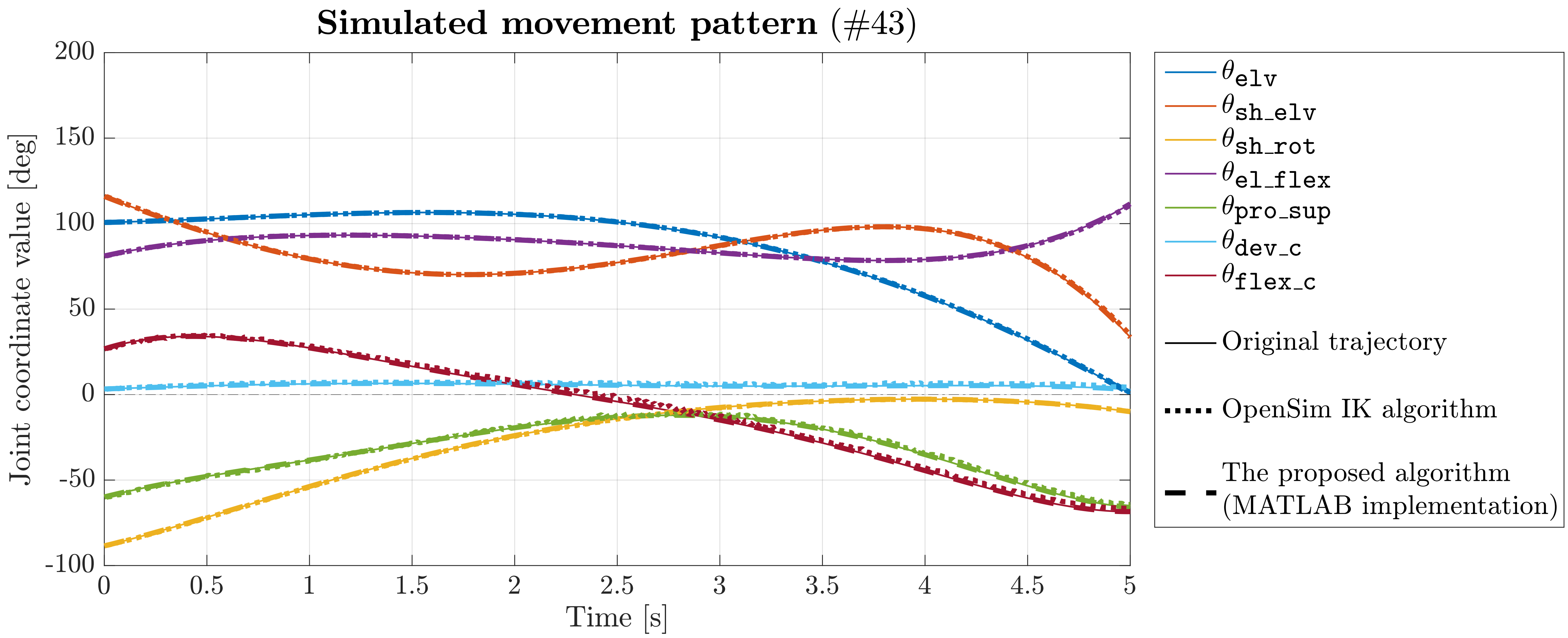

Simulated movement pattern (#43)
Joint coordinate value [deg]
200
150
100
50
0
-50
-100
0
0.5
1
1.5
2
2.5
3
3.5
4
4.5
5
Time [s]
θelv
θsh_elv
θsh_rot
θel_flex
θpro_sup
θdev_c
θflex_c
Original trajectory
OpenSim IK algorithm
The proposed algorithm
(MATLAB implementation)

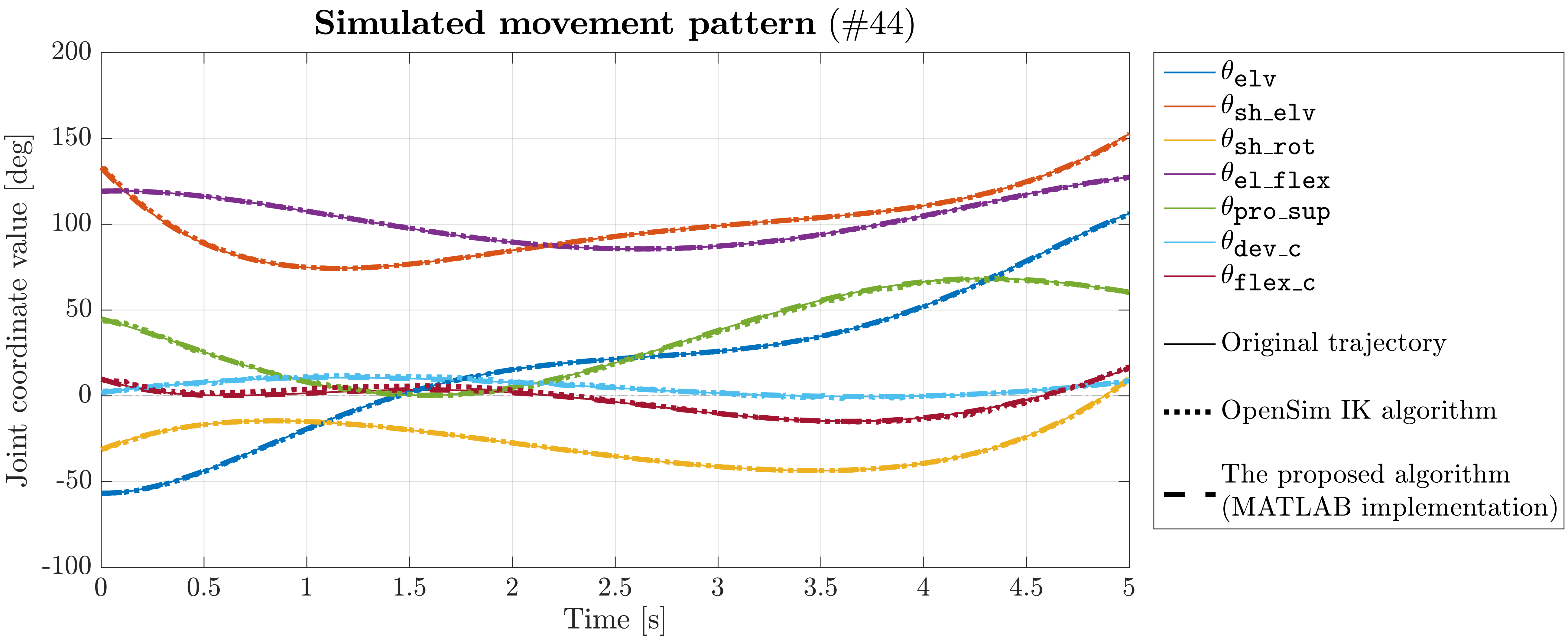

Simulated movement pattern (#44)
200
150
100
50
0
-50
-100
Joint coordinate value [deg]
0
0.5
1
1.5
2
2.5
3
3.5
4
4.5
5
Time [s]
θ_{elv}
θ_{sh_elv}
θ_{sh_rot}
θ_{el_flex}
θ_{pro_sup}
θ_{dev_c}
θ_{flex_c}
Original trajectory
OpenSim IK algorithm
The proposed algorithm (MATLAB implementation)

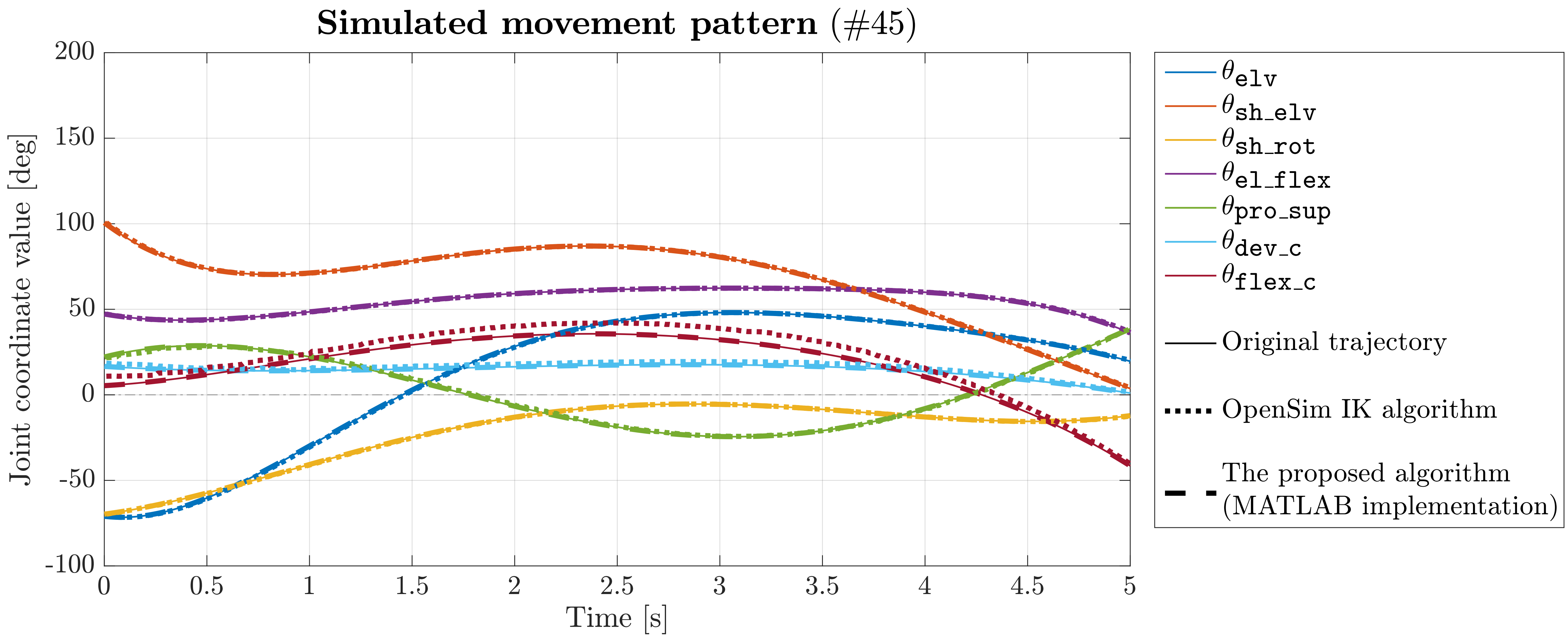

Simulated movement pattern (#45)
200
150
100
50
0
-50
-100
Joint coordinate value [deg]
0
0.5
1
1.5
2
2.5
3
3.5
4
4.5
5
Time [s]
$\theta_{\mathtt{elv}}$
$\theta_{\mathtt{sh\_elv}}$
$\theta_{\mathtt{sh\_rot}}$
$\theta_{\mathtt{el\_flex}}$
$\theta_{\mathtt{pro\_sup}}$
$\theta_{\mathtt{dev\_c}}$
$\theta_{\mathtt{flex\_c}}$
Original trajectory
OpenSim IK algorithm
The proposed algorithm (MATLAB implementation)

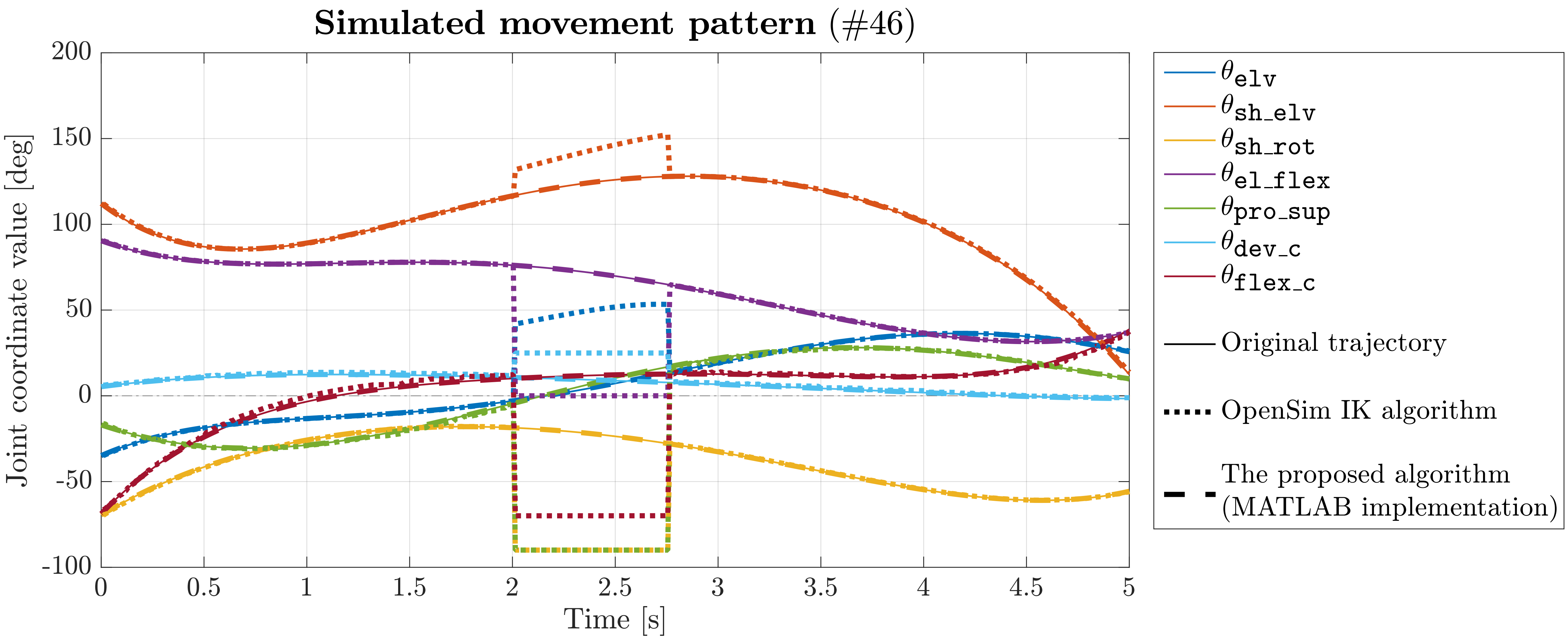

Simulated movement pattern (#46)
200
150
100
50
0
-50
-100
Joint coordinate value [deg]
0
0.5
1
1.5
2
2.5
3
3.5
4
4.5
5
Time [s]
θ_{elv}
θ_{sh_elv}
θ_{sh_rot}
θ_{el_flex}
θ_{pro_sup}
θ_{dev_c}
θ_{flex_c}
Original trajectory
OpenSim IK algorithm
The proposed algorithm (MATLAB implementation)

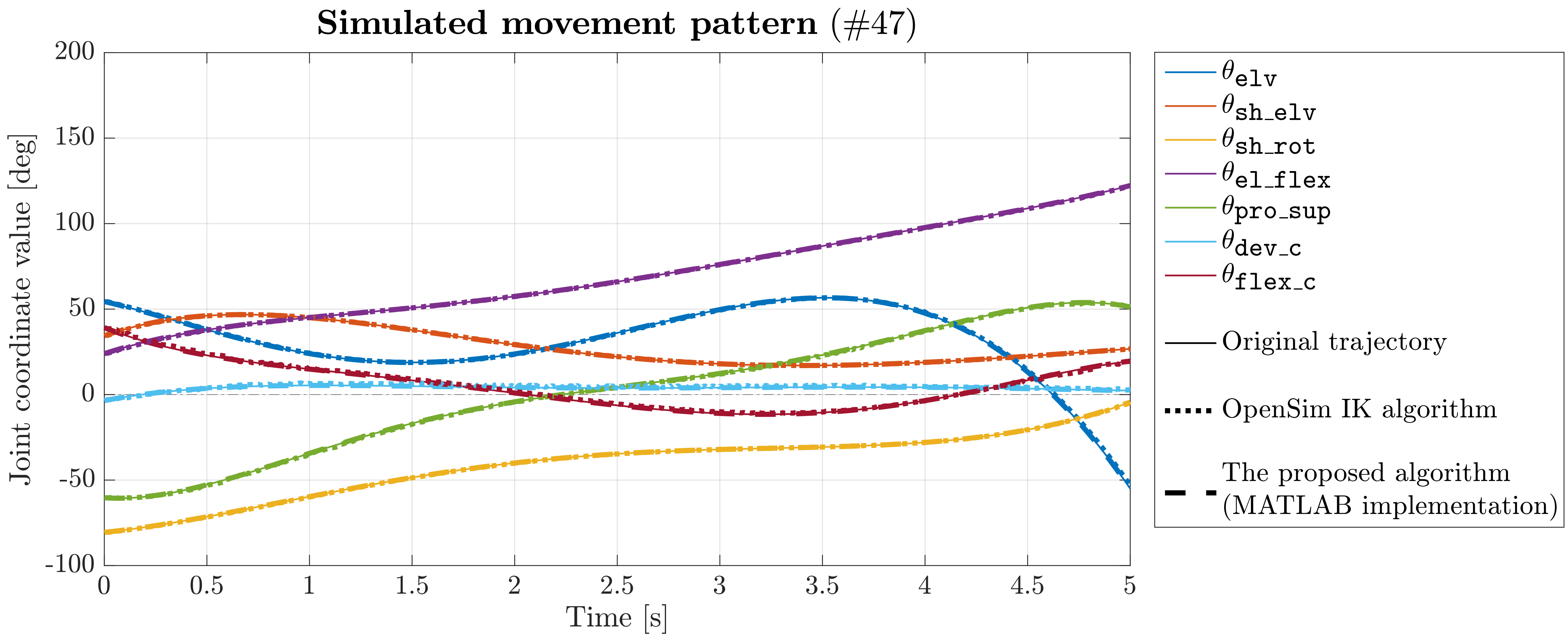

Simulated movement pattern (#47)
Joint coordinate value [deg]
Time [s]
200
150
100
50
0
-50
-100
0
0.5
1
1.5
2
2.5
3
3.5
4
4.5
5
$\theta_{\mathtt{elv}}$
$\theta_{\mathtt{sh\_elv}}$
$\theta_{\mathtt{sh\_rot}}$
$\theta_{\mathtt{el\_flex}}$
$\theta_{\mathtt{pro\_sup}}$
$\theta_{\mathtt{dev\_c}}$
$\theta_{\mathtt{flex\_c}}$
Original trajectory
OpenSim IK algorithm
The proposed algorithm (MATLAB implementation)

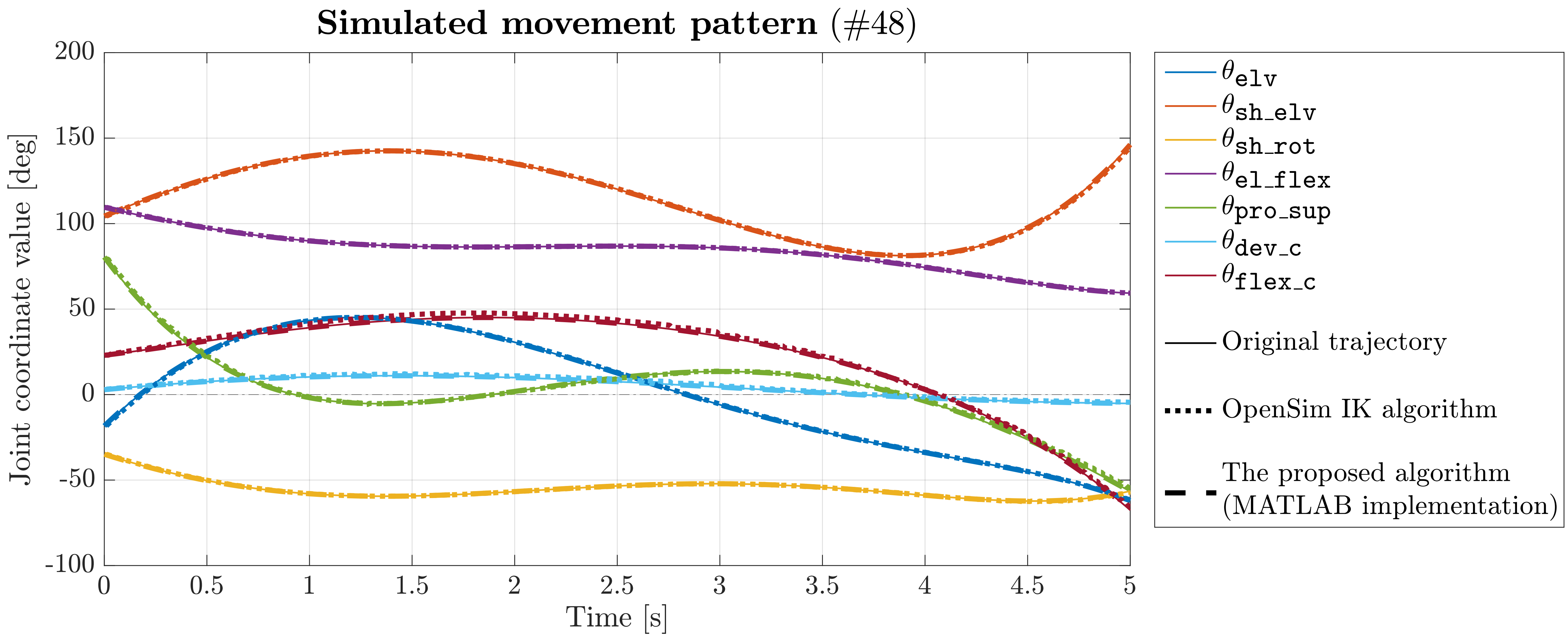

Simulated movement pattern (#48)
200
150
100
50
0
-50
-100
Joint coordinate value [deg]
0
0.5
1
1.5
2
2.5
3
3.5
4
4.5
5
Time [s]
θ_{elv}
θ_{sh_elv}
θ_{sh_rot}
θ_{el_flex}
θ_{pro_sup}
θ_{dev_c}
θ_{flex_c}
Original trajectory
OpenSim IK algorithm
The proposed algorithm (MATLAB implementation)

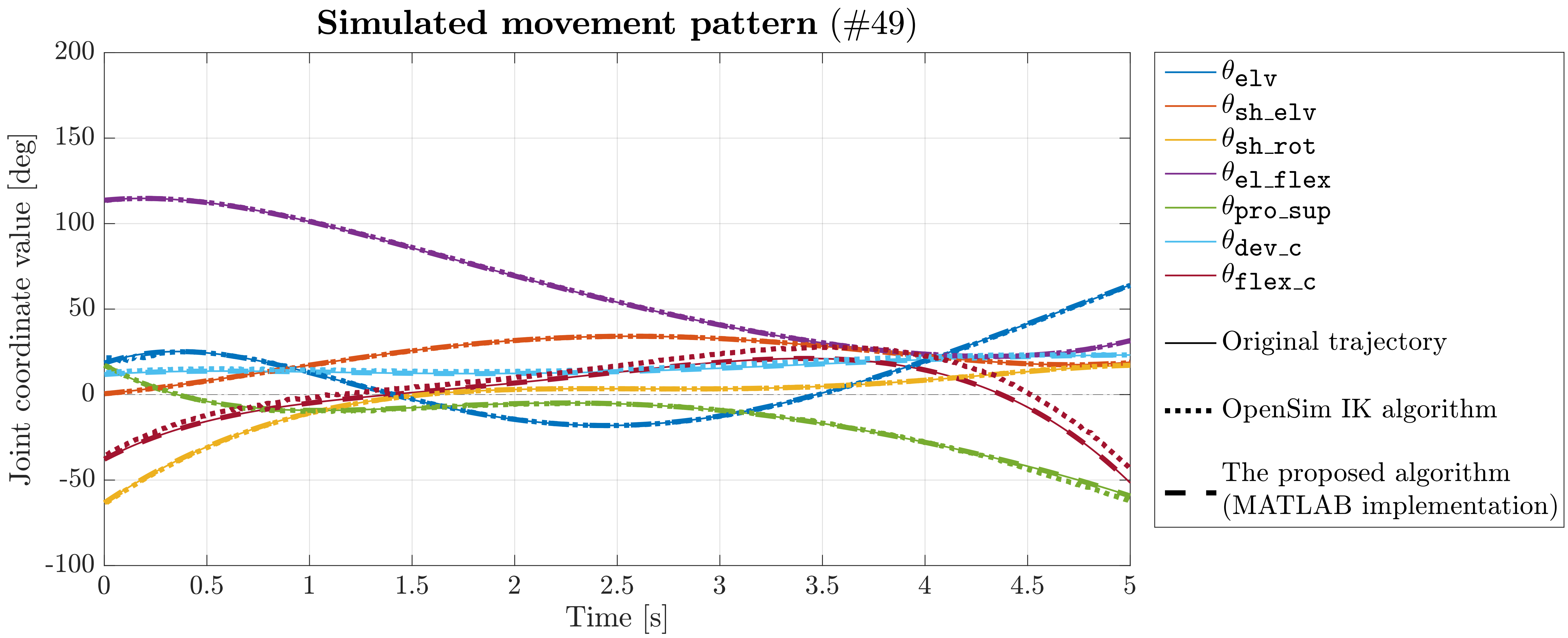

Simulated movement pattern (#49)
Joint coordinate value [deg]
200
150
100
50
0
-50
-100
0
0.5
1
1.5
2
2.5
3
3.5
4
4.5
5
Time [s]
θ_{elv}
θ_{sh_elv}
θ_{sh_rot}
θ_{el_flex}
θ_{pro_sup}
θ_{dev_c}
θ_{flex_c}
Original trajectory
OpenSim IK algorithm
The proposed algorithm (MATLAB implementation)

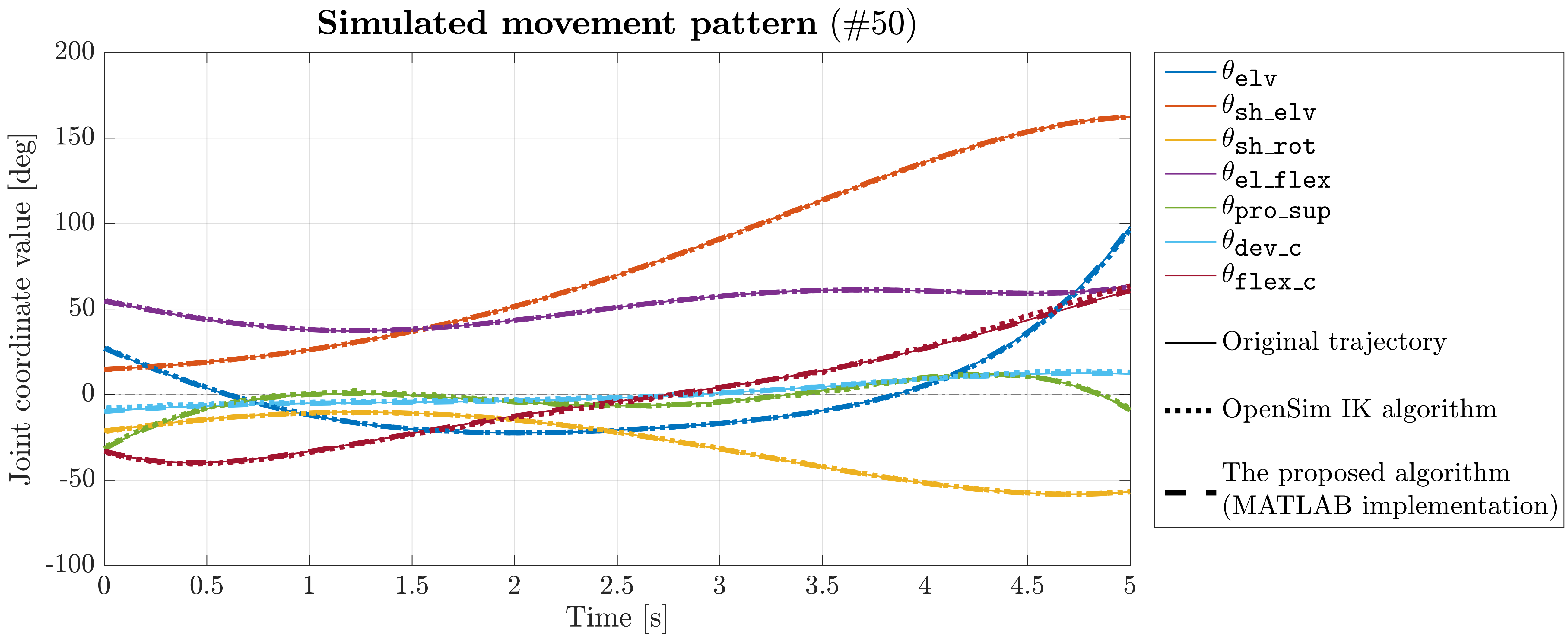

Simulated movement pattern (#50)
200
150
100
50
0
-50
-100
Joint coordinate value [deg]
0
0.5
1
1.5
2
2.5
3
3.5
4
4.5
5
Time [s]
θ_{elv}
θ_{sh_elv}
θ_{sh_rot}
θ_{el_flex}
θ_{pro_sup}
θ_{dev_c}
θ_{flex_c}
Original trajectory
OpenSim IK algorithm
The proposed algorithm (MATLAB implementation)

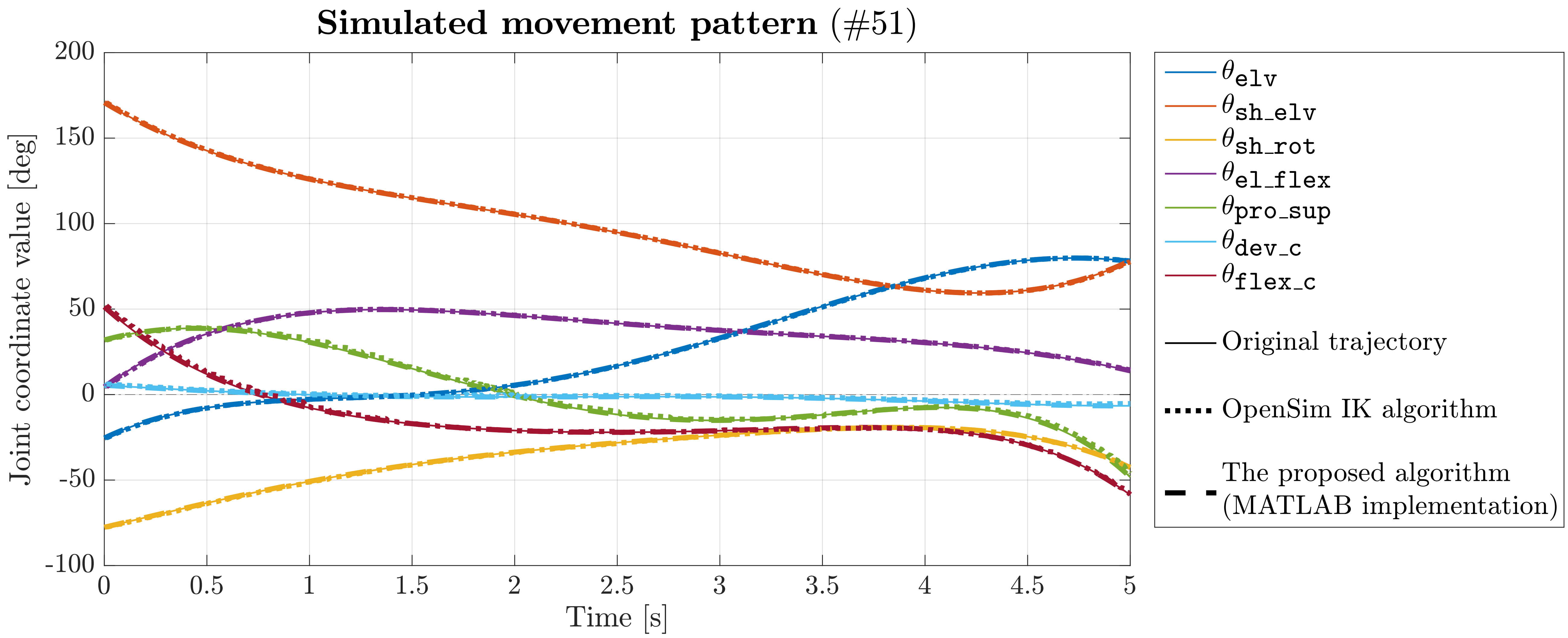
Simulated movement pattern (#51)
Joint coordinate value [deg]
Time [s]
200
150
100
50
0
-50
-100
0
0.5
1
1.5
2
2.5
3
3.5
4
4.5
5
$\theta_{\mathtt{elv}}$
$\theta_{\mathtt{sh\_elv}}$
$\theta_{\mathtt{sh\_rot}}$
$\theta_{\mathtt{el\_flex}}$
$\theta_{\mathtt{pro\_sup}}$
$\theta_{\mathtt{dev\_c}}$
$\theta_{\mathtt{flex\_c}}$
Original trajectory
OpenSim IK algorithm
The proposed algorithm (MATLAB implementation)

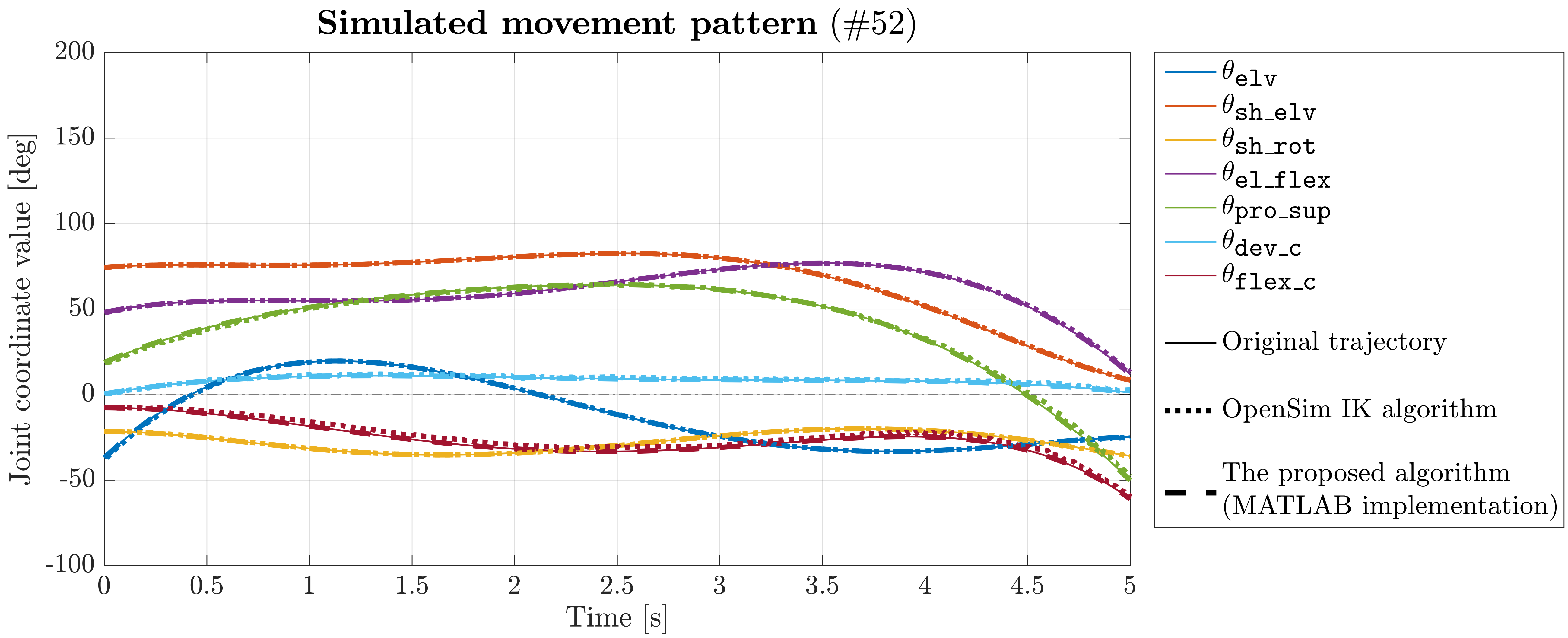

Simulated movement pattern (#52)
Joint coordinate value [deg]
200
150
100
50
0
-50
-100
0
0.5
1
1.5
2
2.5
3
3.5
4
4.5
5
Time [s]
θ_{elv}
θ_{sh_elv}
θ_{sh_rot}
θ_{el_flex}
θ_{pro_sup}
θ_{dev_c}
θ_{flex_c}
Original trajectory
OpenSim IK algorithm
The proposed algorithm
(MATLAB implementation)

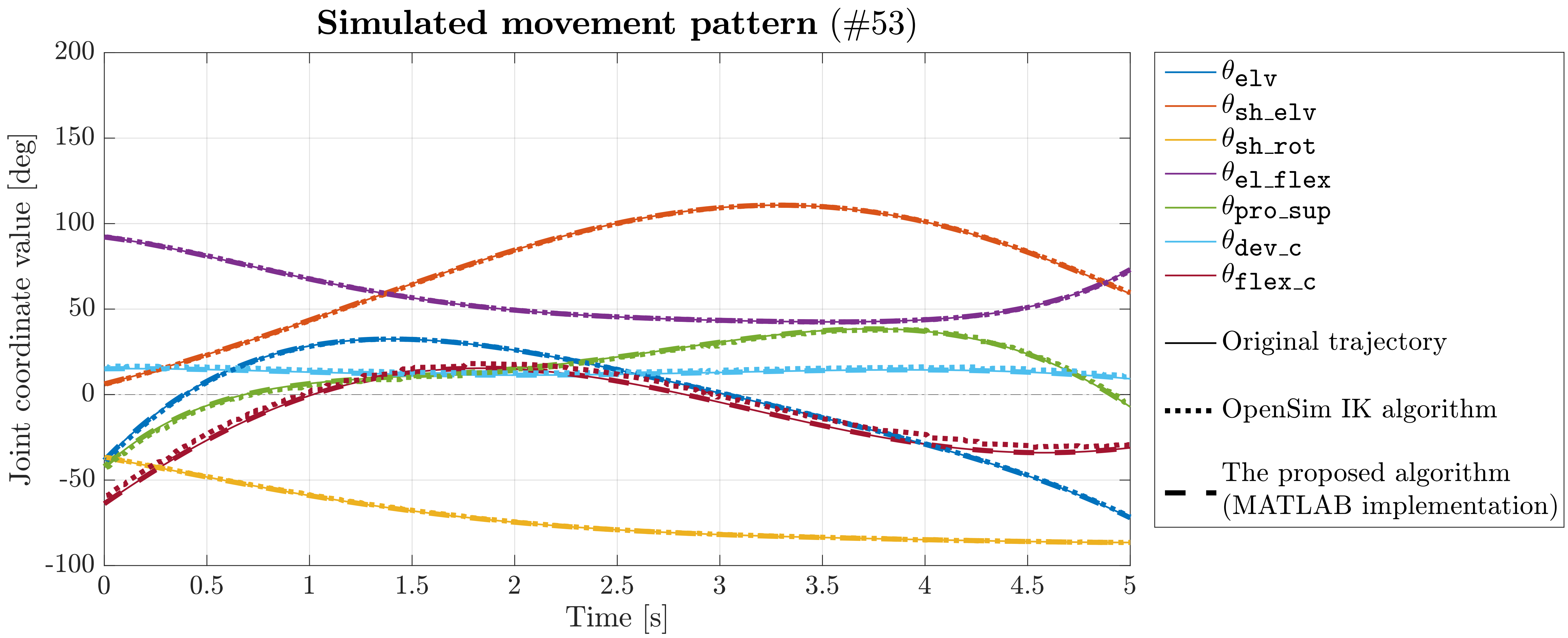

Simulated movement pattern (#53)
Joint coordinate value [deg]
200
150
100
50
0
-50
-100
0
0.5
1
1.5
2
2.5
3
3.5
4
4.5
5
Time [s]
θelv
θsh_elv
θsh_rot
θel_flex
θpro_sup
θdev_c
θflex_c
Original trajectory
OpenSim IK algorithm
The proposed algorithm
(MATLAB implementation)

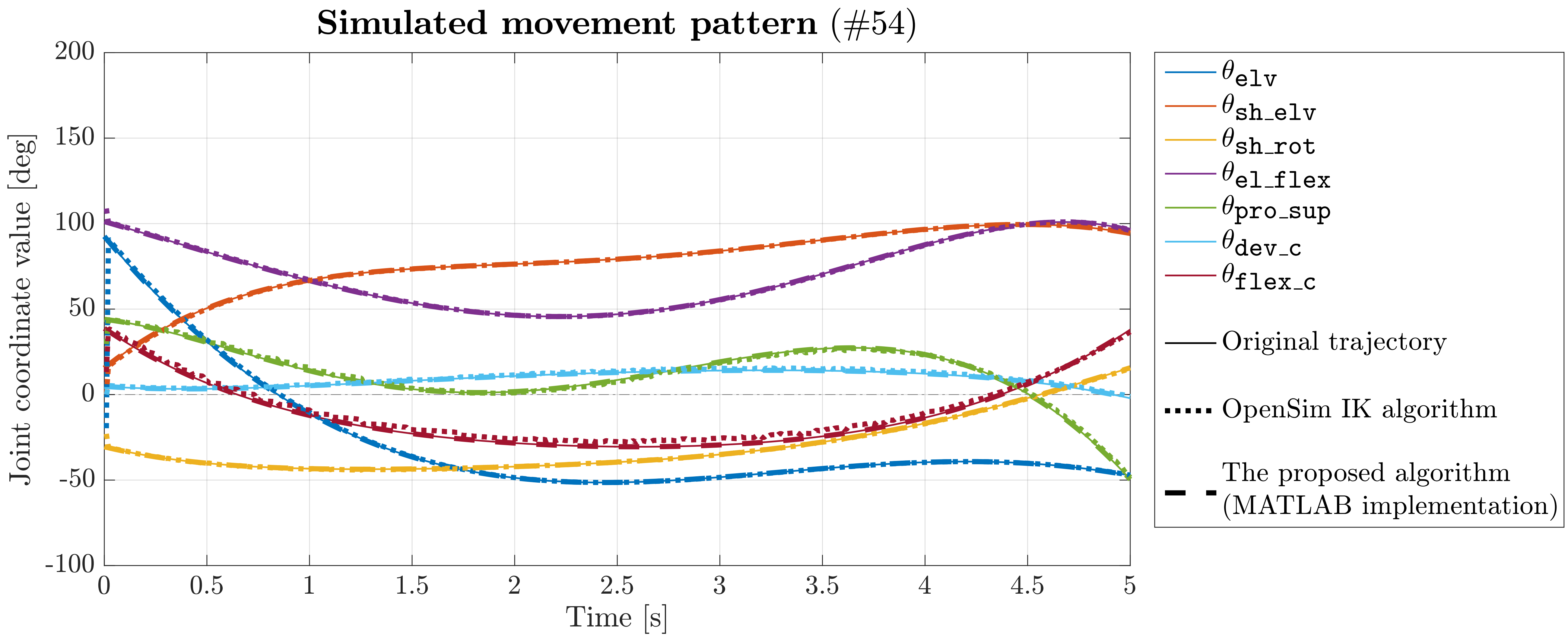

Simulated movement pattern (#54)
Joint coordinate value [deg]
200
150
100
50
0
-50
-100
Time [s]
0
0.5
1
1.5
2
2.5
3
3.5
4
4.5
5
θelv
θsh_elv
θsh_rot
θel_flex
θpro_sup
θdev_c
θflex_c
Original trajectory
OpenSim IK algorithm
The proposed algorithm (MATLAB implementation)

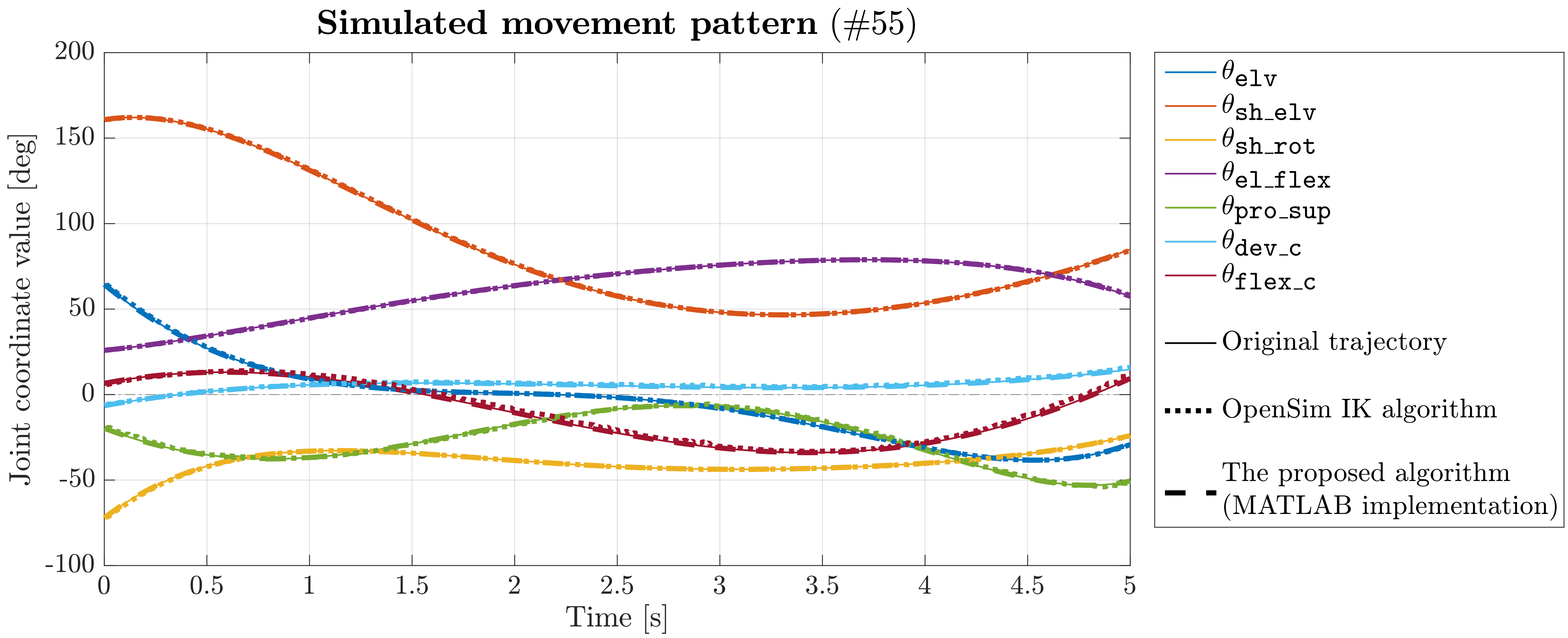

Simulated movement pattern (#55)
200
150
100
50
0
-50
-100
Joint coordinate value [deg]
0
0.5
1
1.5
2
2.5
3
3.5
4
4.5
5
Time [s]
θelv
θsh_elv
θsh_rot
θel_flex
θpro_sup
θdev_c
θflex_c
Original trajectory
OpenSim IK algorithm
The proposed algorithm (MATLAB implementation)

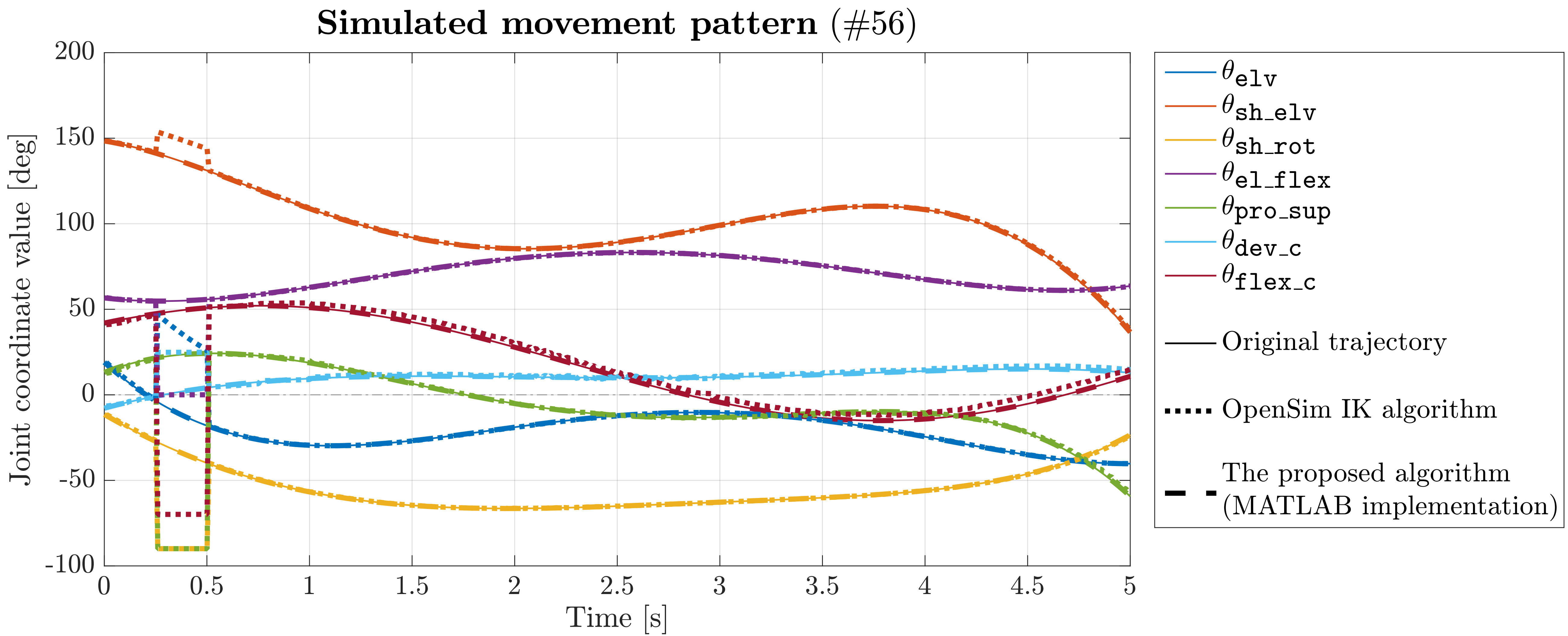

Simulated movement pattern (#56)
200
150
100
50
0
-50
-100
Joint coordinate value [deg]
0
0.5
1
1.5
2
2.5
3
3.5
4
4.5
5
Time [s]
$\theta_{\texttt{elv}}$
$\theta_{\texttt{sh\_elv}}$
$\theta_{\texttt{sh\_rot}}$
$\theta_{\texttt{el\_flex}}$
$\theta_{\texttt{pro\_sup}}$
$\theta_{\texttt{dev\_c}}$
$\theta_{\texttt{flex\_c}}$
Original trajectory
OpenSim IK algorithm
The proposed algorithm
(MATLAB implementation)

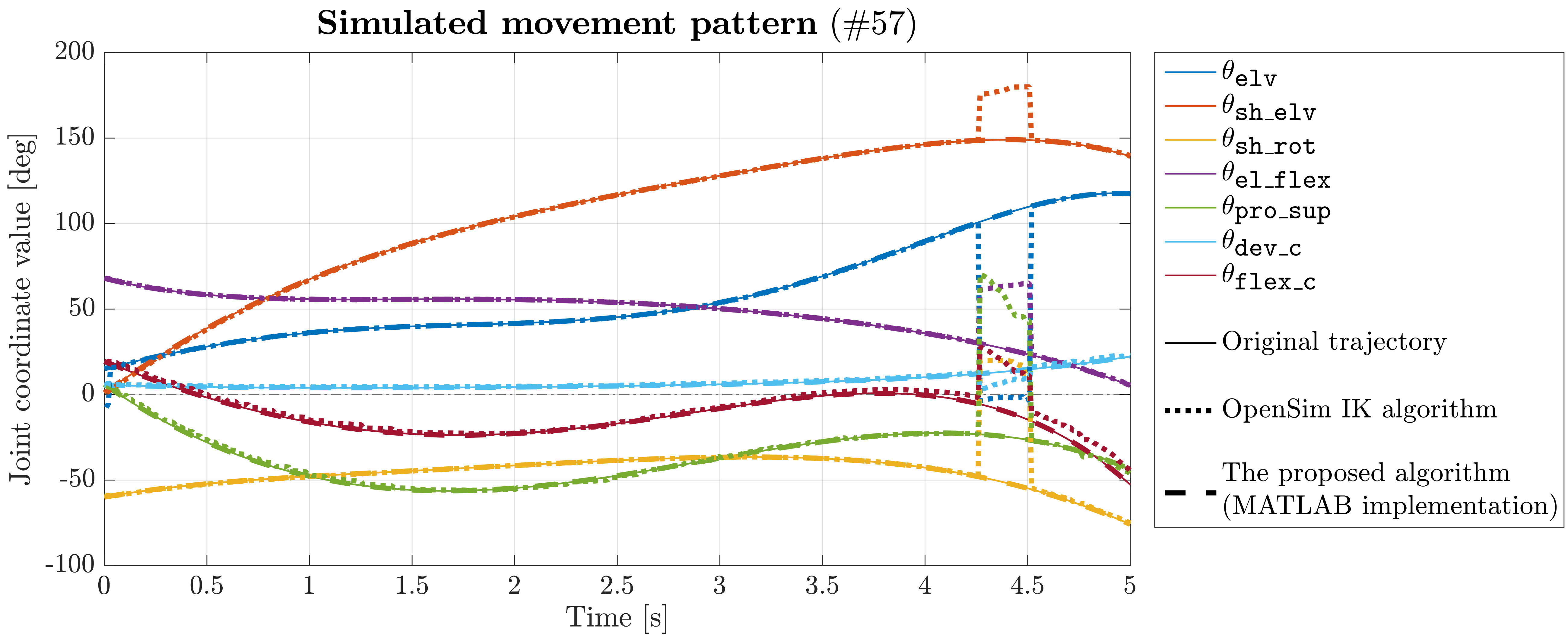
Simulated movement pattern (#57)
Joint coordinate value [deg]
Time [s]
200
150
100
50
0
-50
-100
0
0.5
1
1.5
2
2.5
3
3.5
4
4.5
5
θ_{elv}
θ_{sh_elv}
θ_{sh_rot}
θ_{el_flex}
θ_{pro_sup}
θ_{dev_c}
θ_{flex_c}
Original trajectory
OpenSim IK algorithm
The proposed algorithm (MATLAB implementation)

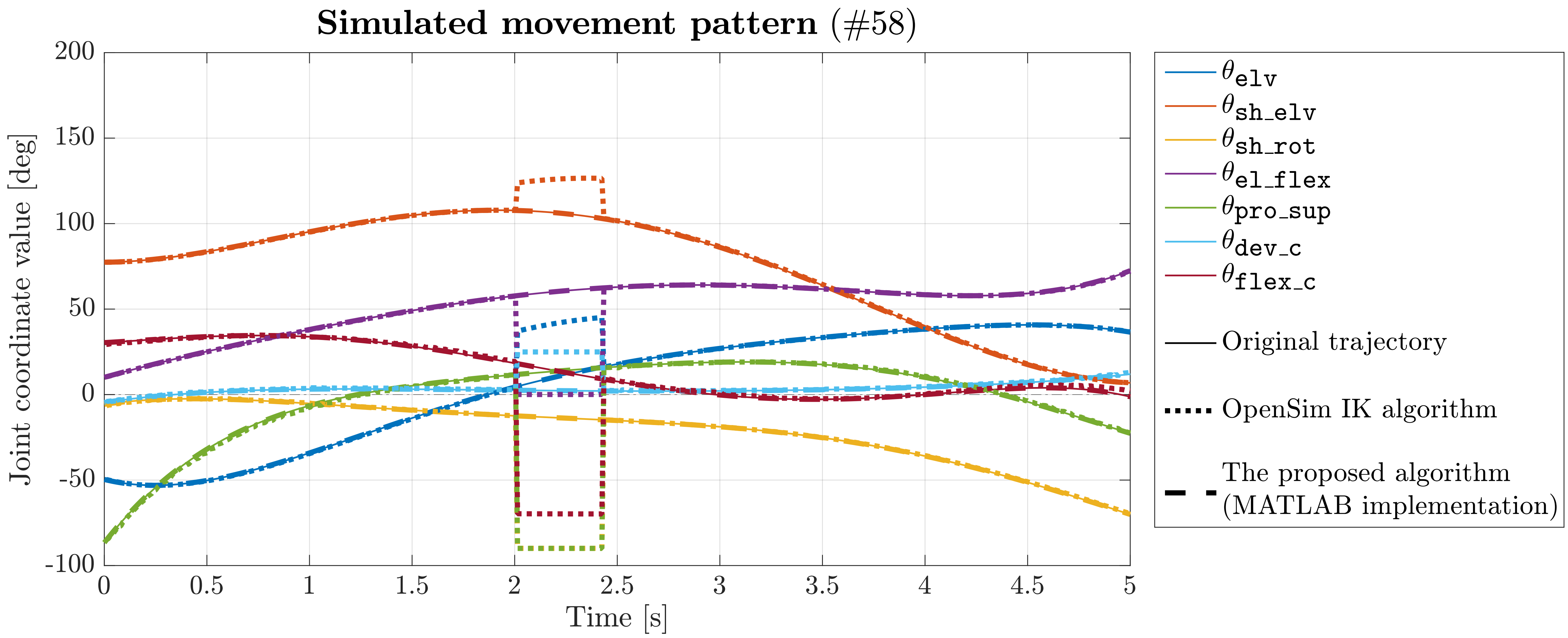

Simulated movement pattern (#58)
Joint coordinate value [deg]
200
150
100
50
0
-50
-100
0
0.5
1
1.5
2
2.5
3
3.5
4
4.5
5
Time [s]
θelv
θsh_elv
θsh_rot
θel_flex
θpro_sup
θdev_c
θflex_c
Original trajectory
OpenSim IK algorithm
The proposed algorithm
(MATLAB implementation)

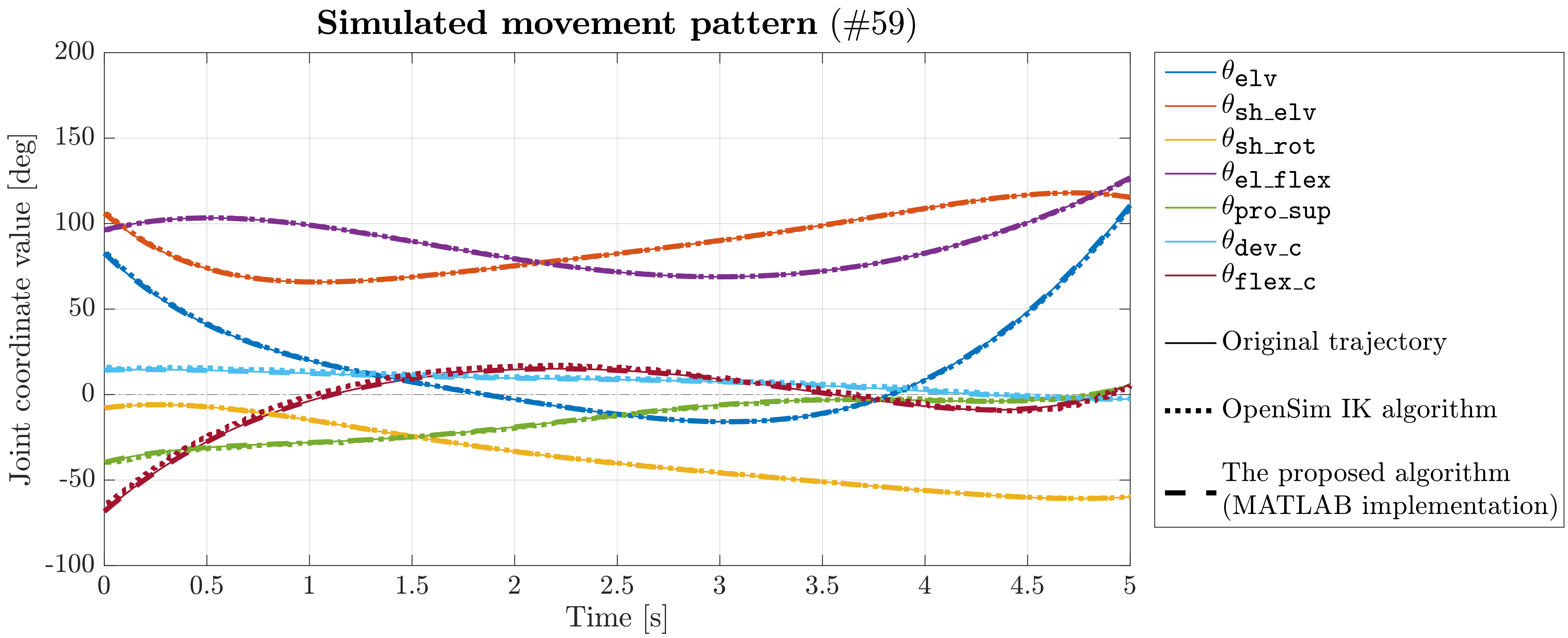

Simulated movement pattern (#59)
Joint coordinate value [deg]
Time [s]
200
150
100
50
0
-50
-100
0
0.5
1
1.5
2
2.5
3
3.5
4
4.5
5
θ_{elv}
θ_{sh_elv}
θ_{sh_rot}
θ_{el_flex}
θ_{pro_sup}
θ_{dev_c}
θ_{flex_c}
Original trajectory
OpenSim IK algorithm
The proposed algorithm (MATLAB implementation)

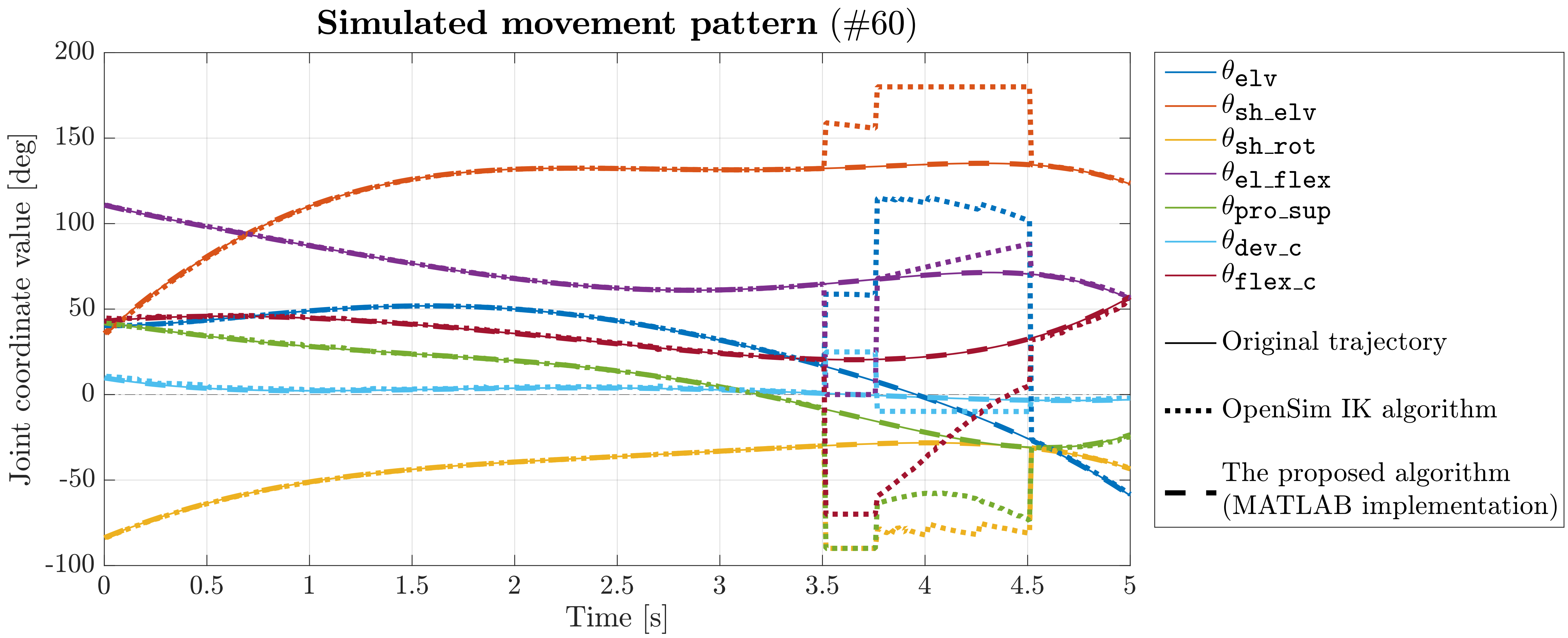

Simulated movement pattern (#60)
Joint coordinate value [deg]
Time [s]
200
150
100
50
0
-50
-100
0
0.5
1
1.5
2
2.5
3
3.5
4
4.5
5
θ_{elv}
θ_{sh_elv}
θ_{sh_rot}
θ_{el_flex}
θ_{pro_sup}
θ_{dev_c}
θ_{flex_c}
Original trajectory
OpenSim IK algorithm
The proposed algorithm (MATLAB implementation)

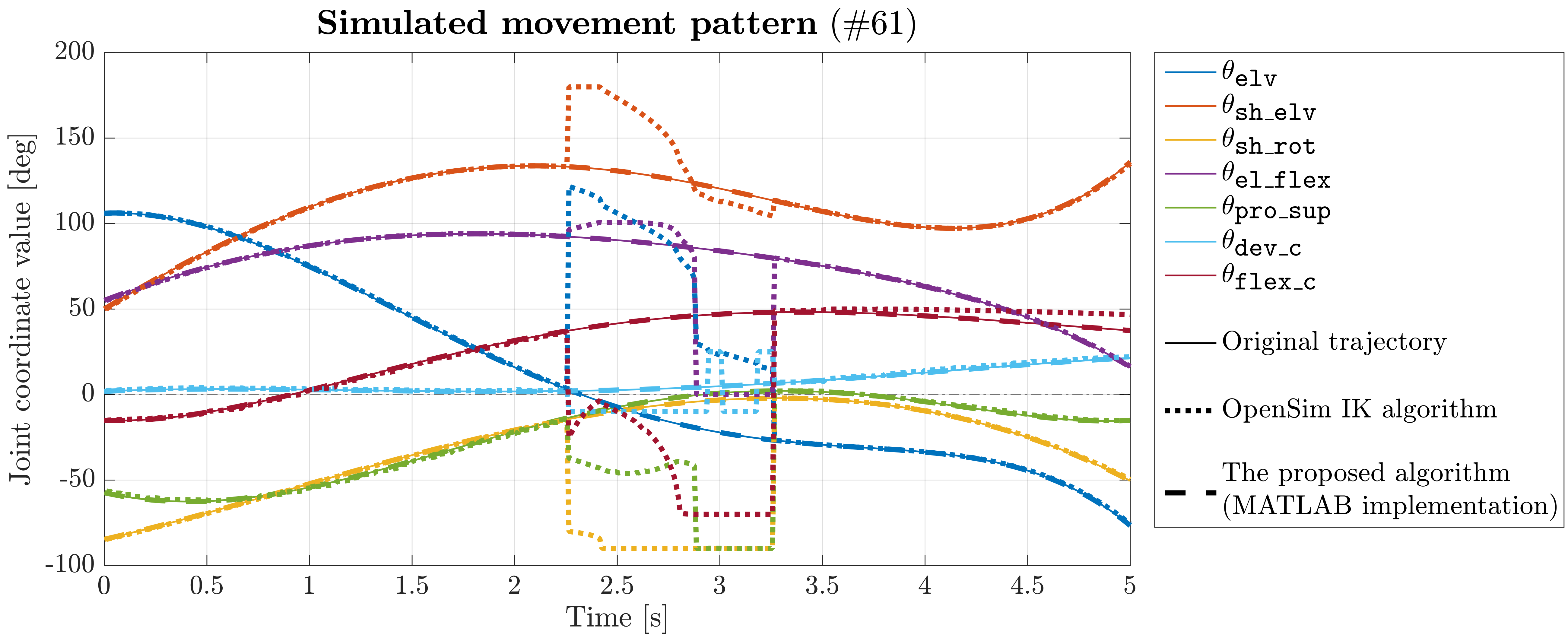

Simulated movement pattern (#61)
200
150
100
50
0
-50
-100
Joint coordinate value [deg]
0
0.5
1
1.5
2
2.5
3
3.5
4
4.5
5
Time [s]
$\theta_{\mathtt{elv}}$
$\theta_{\mathtt{sh\_elv}}$
$\theta_{\mathtt{sh\_rot}}$
$\theta_{\mathtt{el\_flex}}$
$\theta_{\mathtt{pro\_sup}}$
$\theta_{\mathtt{dev\_c}}$
$\theta_{\mathtt{flex\_c}}$
Original trajectory
OpenSim IK algorithm
The proposed algorithm (MATLAB implementation)

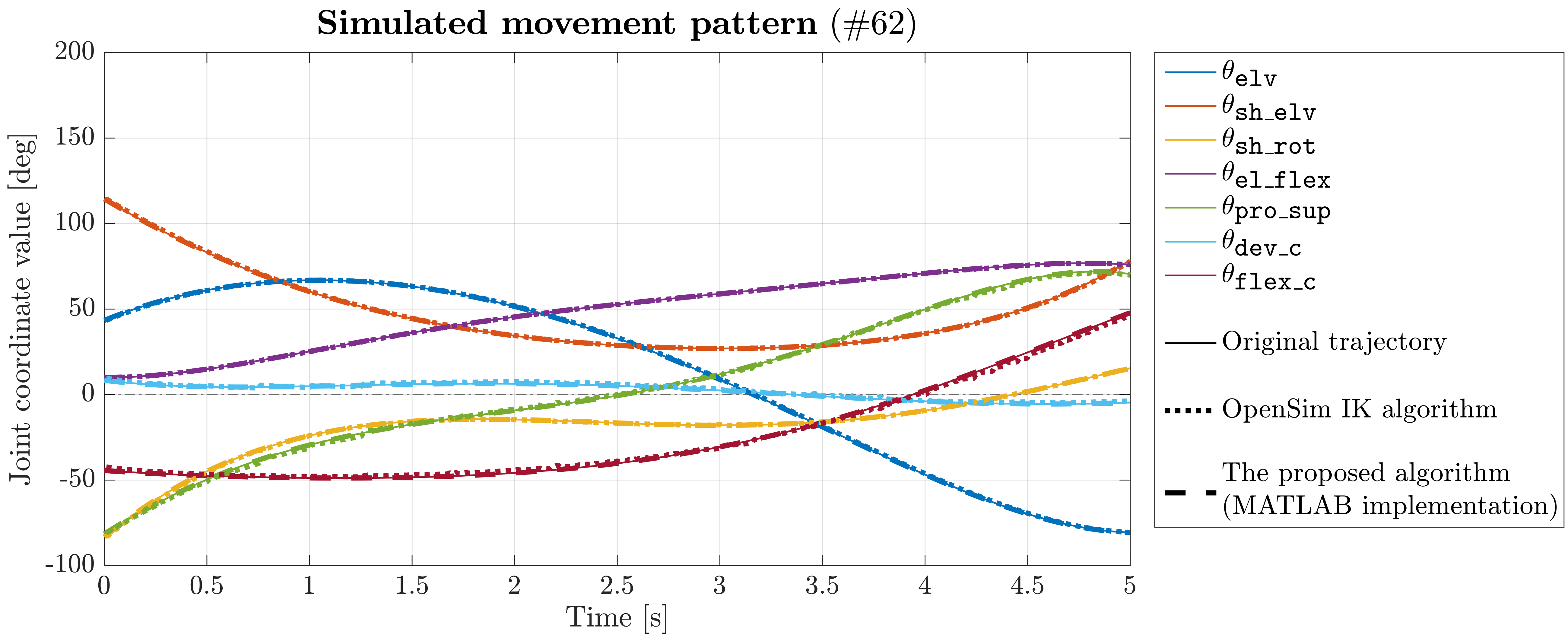
Simulated movement pattern (#62)
Joint coordinate value [deg]
Time [s]
200
150
100
50
0
-50
-100
0
0.5
1
1.5
2
2.5
3
3.5
4
4.5
5
$\theta_{\texttt{elv}}$
$\theta_{\texttt{sh\_elv}}$
$\theta_{\texttt{sh\_rot}}$
$\theta_{\texttt{el\_flex}}$
$\theta_{\texttt{pro\_sup}}$
$\theta_{\texttt{dev\_c}}$
$\theta_{\texttt{flex\_c}}$
Original trajectory
OpenSim IK algorithm
The proposed algorithm
(MATLAB implementation)

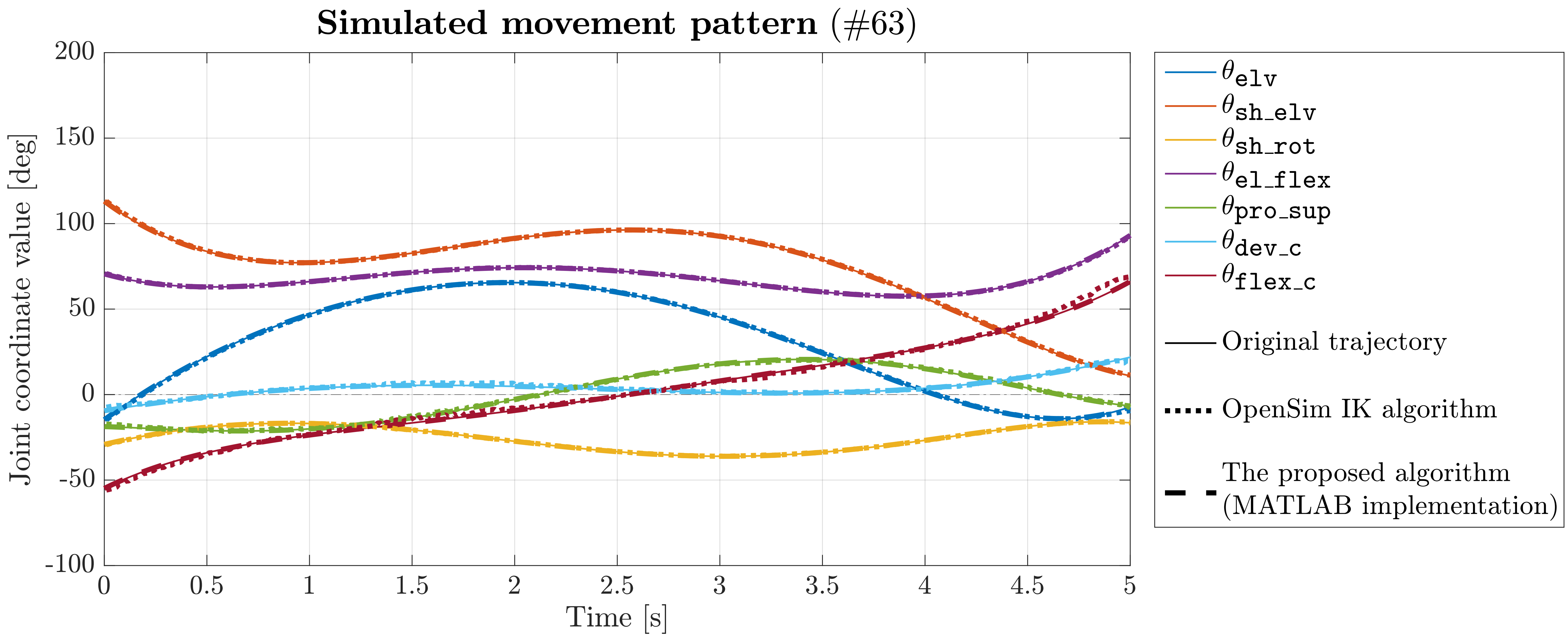

Simulated movement pattern (#63)
200
150
100
50
0
-50
-100
Joint coordinate value [deg]
0
0.5
1
1.5
2
2.5
3
3.5
4
4.5
5
Time [s]
θelv
θsh_elv
θsh_rot
θel_flex
θpro_sup
θdev_c
θflex_c
Original trajectory
OpenSim IK algorithm
The proposed algorithm (MATLAB implementation)

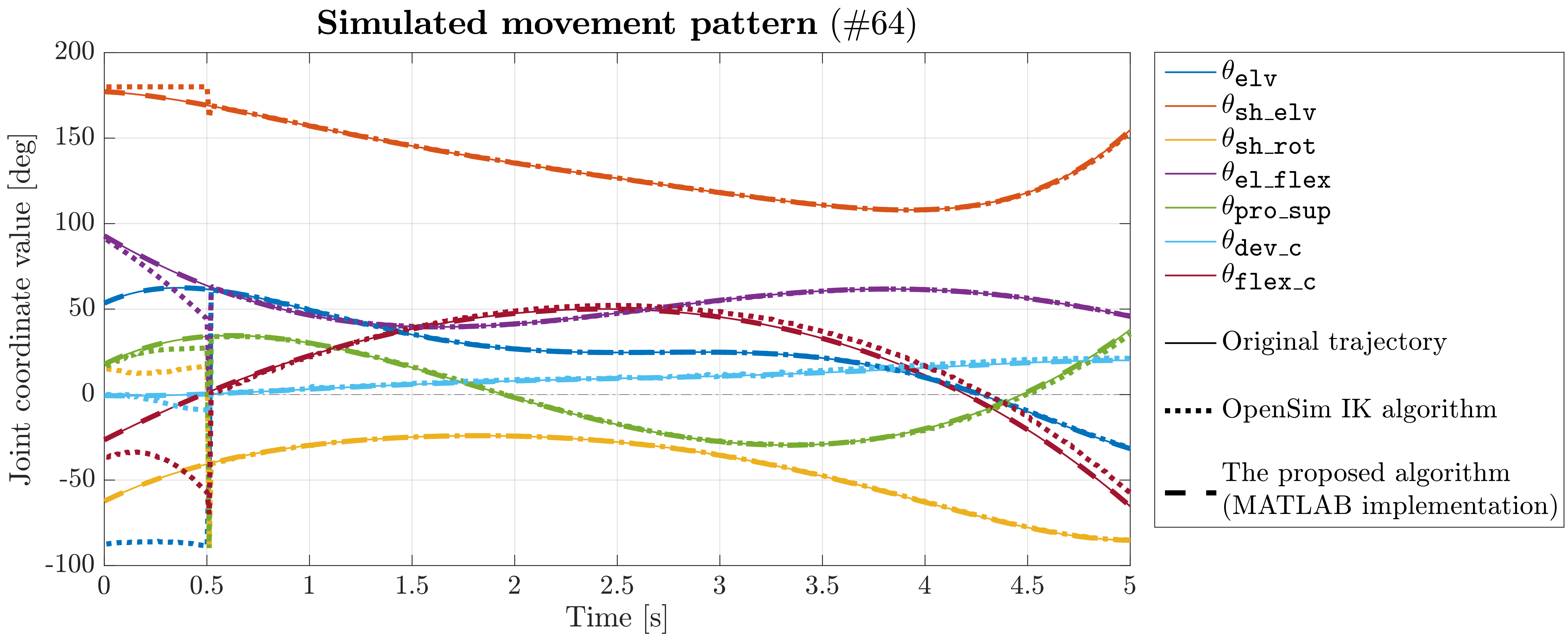

Simulated movement pattern (#64)
Joint coordinate value [deg]
Time [s]
200
150
100
50
0
-50
-100
0
0.5
1
1.5
2
2.5
3
3.5
4
4.5
5
θ_{elv}
θ_{sh_elv}
θ_{sh_rot}
θ_{el_flex}
θ_{pro_sup}
θ_{dev_c}
θ_{flex_c}
Original trajectory
OpenSim IK algorithm
The proposed algorithm (MATLAB implementation)

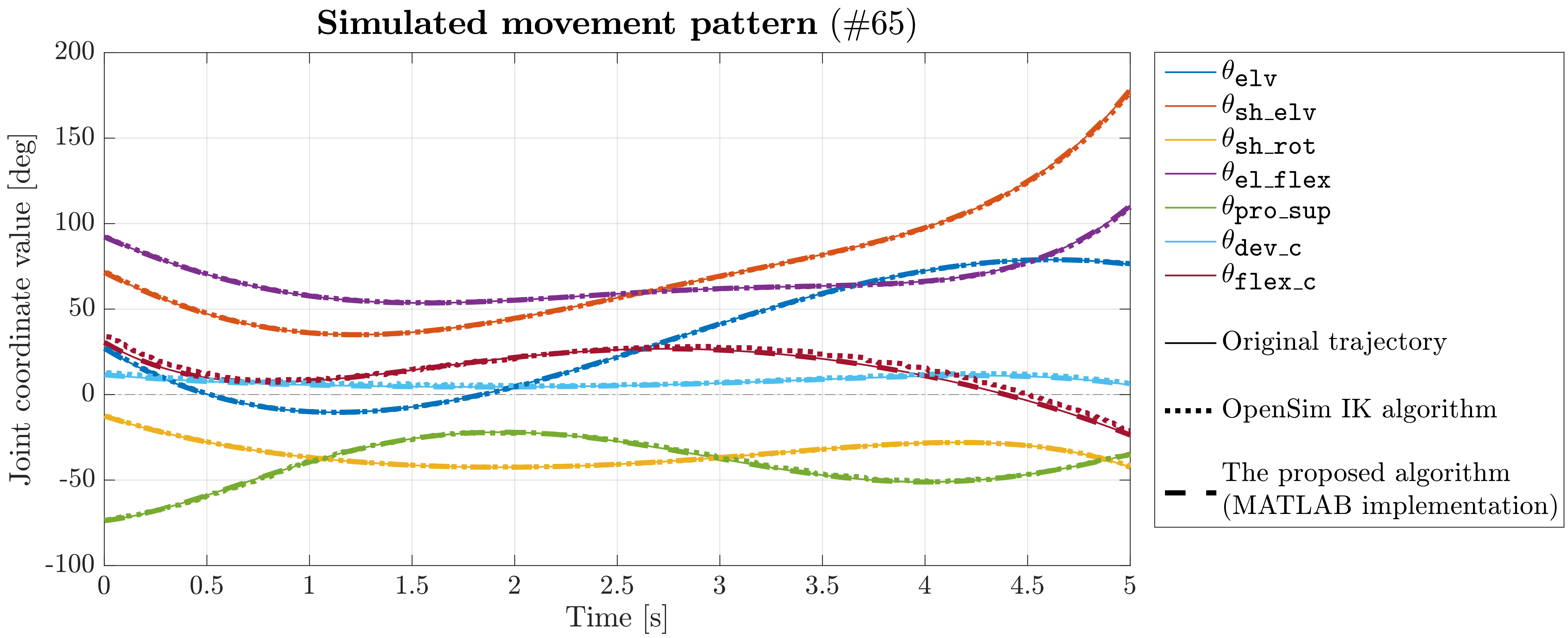

Simulated movement pattern (#65)
Joint coordinate value [deg]
200
150
100
50
0
-50
-100
0
0.5
1
1.5
2
2.5
3
3.5
4
4.5
5
Time [s]
$\theta_{\texttt{elv}}$
$\theta_{\texttt{sh\_elv}}$
$\theta_{\texttt{sh\_rot}}$
$\theta_{\texttt{el\_flex}}$
$\theta_{\texttt{pro\_sup}}$
$\theta_{\texttt{dev\_c}}$
$\theta_{\texttt{flex\_c}}$
Original trajectory
OpenSim IK algorithm
The proposed algorithm (MATLAB implementation)

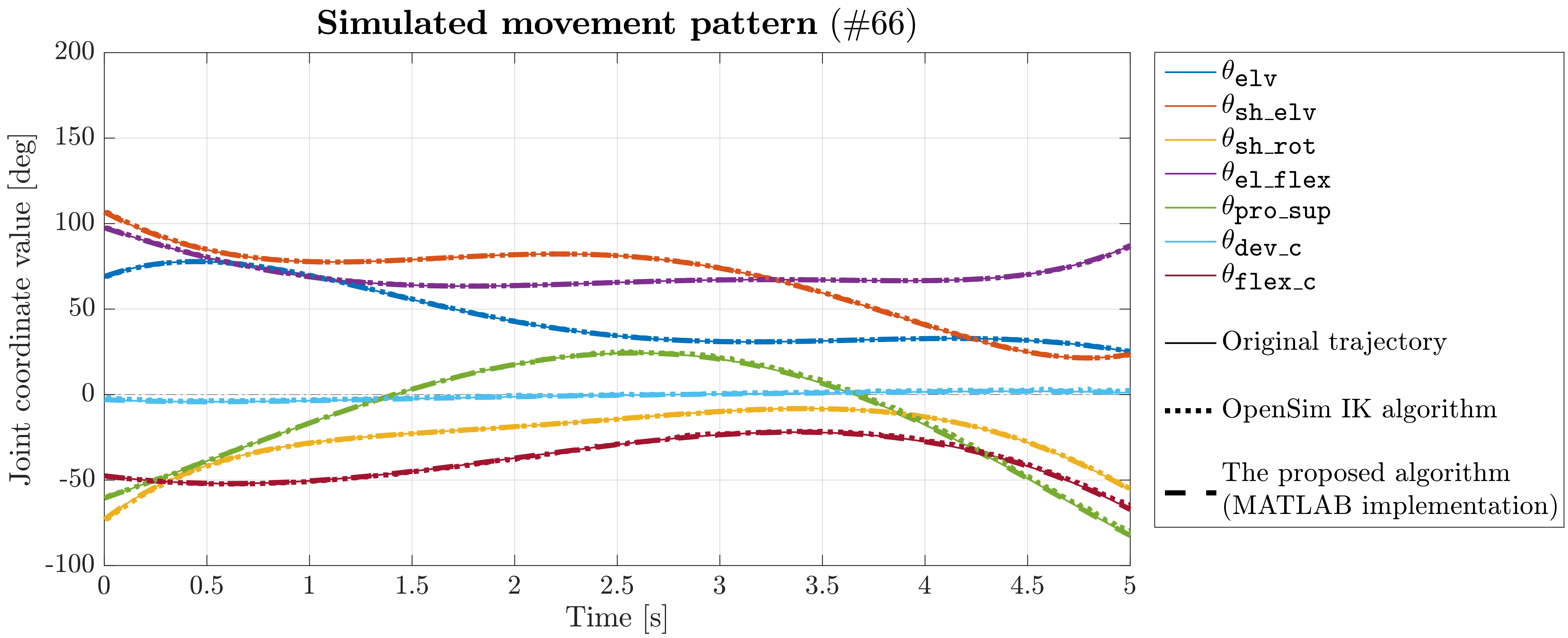

Simulated movement pattern (#66)
Joint coordinate value [deg]
Time [s]
200
150
100
50
0
-50
-100
0
0.5
1
1.5
2
2.5
3
3.5
4
4.5
5
θ_{elv}
θ_{sh_elv}
θ_{sh_rot}
θ_{el_flex}
θ_{pro_sup}
θ_{dev_c}
θ_{flex_c}
Original trajectory
OpenSim IK algorithm
The proposed algorithm (MATLAB implementation)

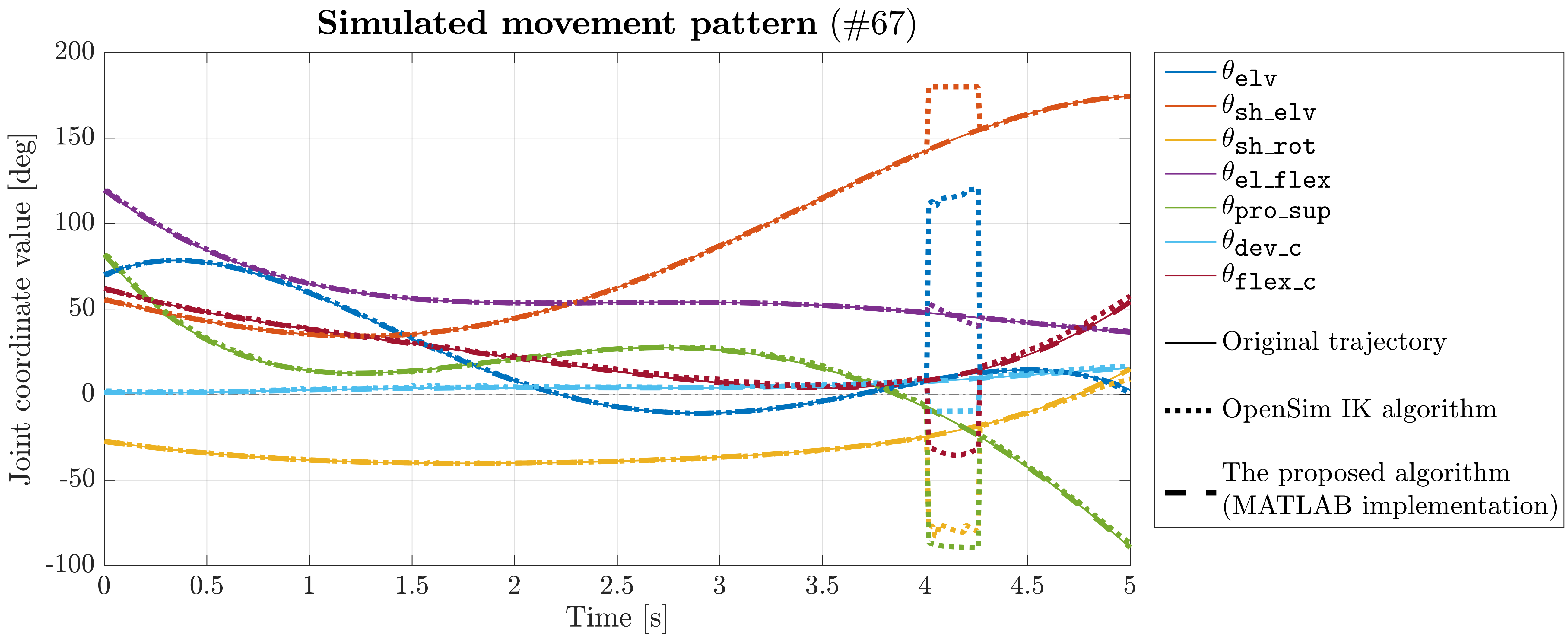

Simulated movement pattern (#67)
200
150
100
50
0
-50
-100
Joint coordinate value [deg]
0
0.5
1
1.5
2
2.5
3
3.5
4
4.5
5
Time [s]
$\theta_{\mathtt{elv}}$
$\theta_{\mathtt{sh\_elv}}$
$\theta_{\mathtt{sh\_rot}}$
$\theta_{\mathtt{el\_flex}}$
$\theta_{\mathtt{pro\_sup}}$
$\theta_{\mathtt{dev\_c}}$
$\theta_{\mathtt{flex\_c}}$
Original trajectory
OpenSim IK algorithm
The proposed algorithm
(MATLAB implementation)

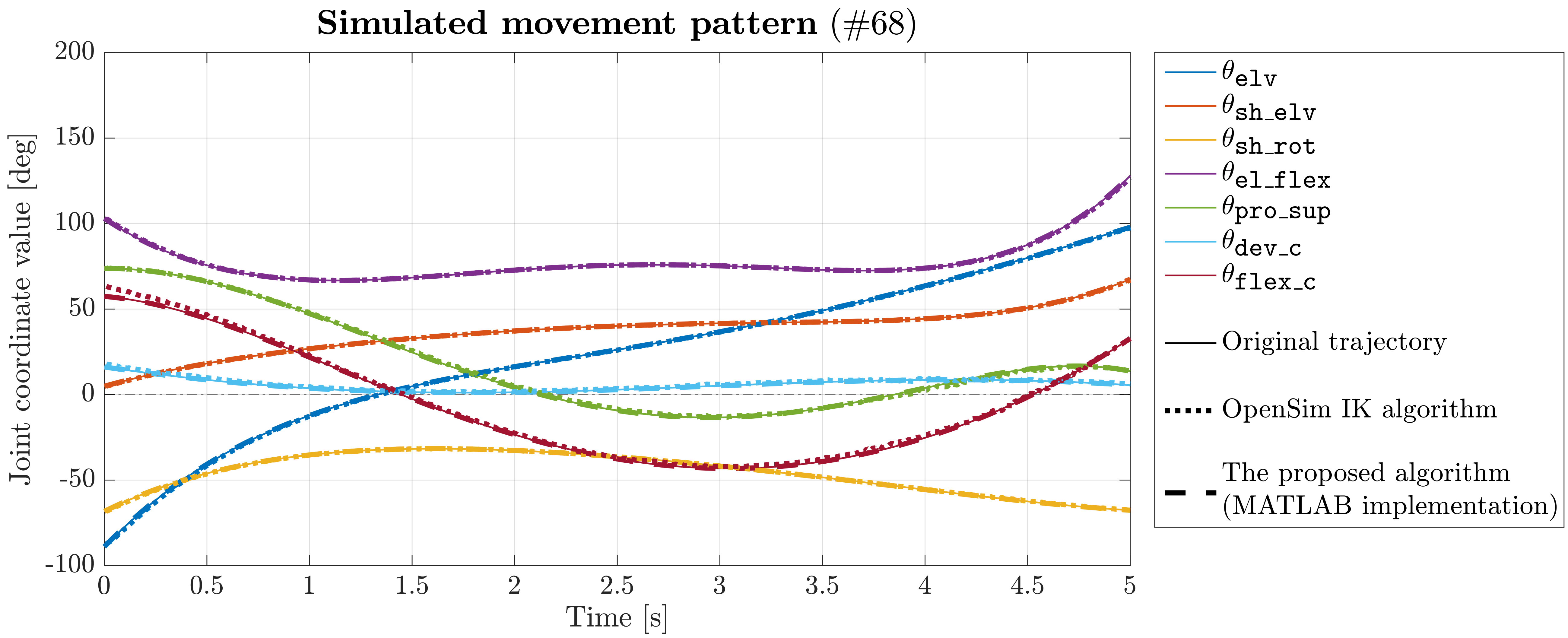

Simulated movement pattern (#68)
Joint coordinate value [deg]
200
150
100
50
0
-50
-100
0
0.5
1
1.5
2
2.5
3
3.5
4
4.5
5
Time [s]
θelv
θsh_elv
θsh_rot
θel_flex
θpro_sup
θdev_c
θflex_c
Original trajectory
OpenSim IK algorithm
The proposed algorithm
(MATLAB implementation)

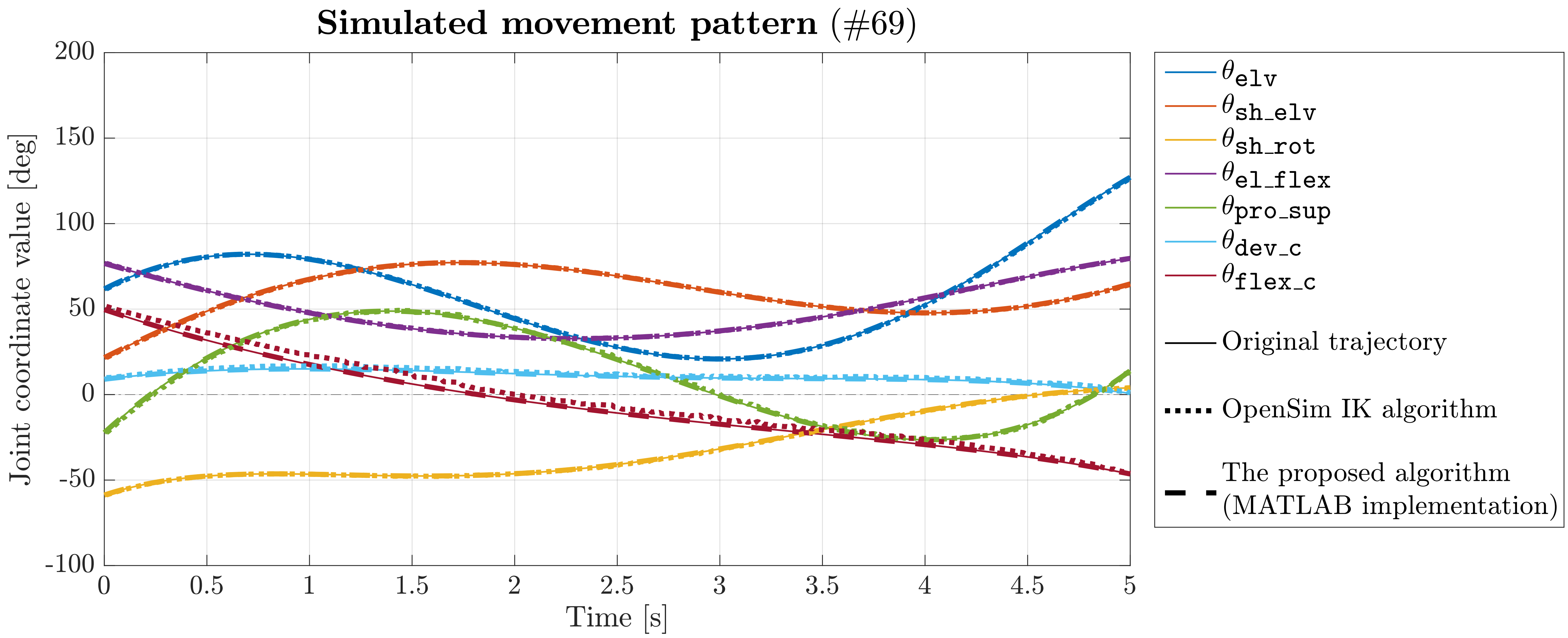

Simulated movement pattern (#69)
Joint coordinate value [deg]
200
150
100
50
0
-50
-100
0
0.5
1
1.5
2
2.5
3
3.5
4
4.5
5
Time [s]
$\theta_{\mathtt{elv}}$
$\theta_{\mathtt{sh\_elv}}$
$\theta_{\mathtt{sh\_rot}}$
$\theta_{\mathtt{el\_flex}}$
$\theta_{\mathtt{pro\_sup}}$
$\theta_{\mathtt{dev\_c}}$
$\theta_{\mathtt{flex\_c}}$
Original trajectory
OpenSim IK algorithm
The proposed algorithm (MATLAB implementation)

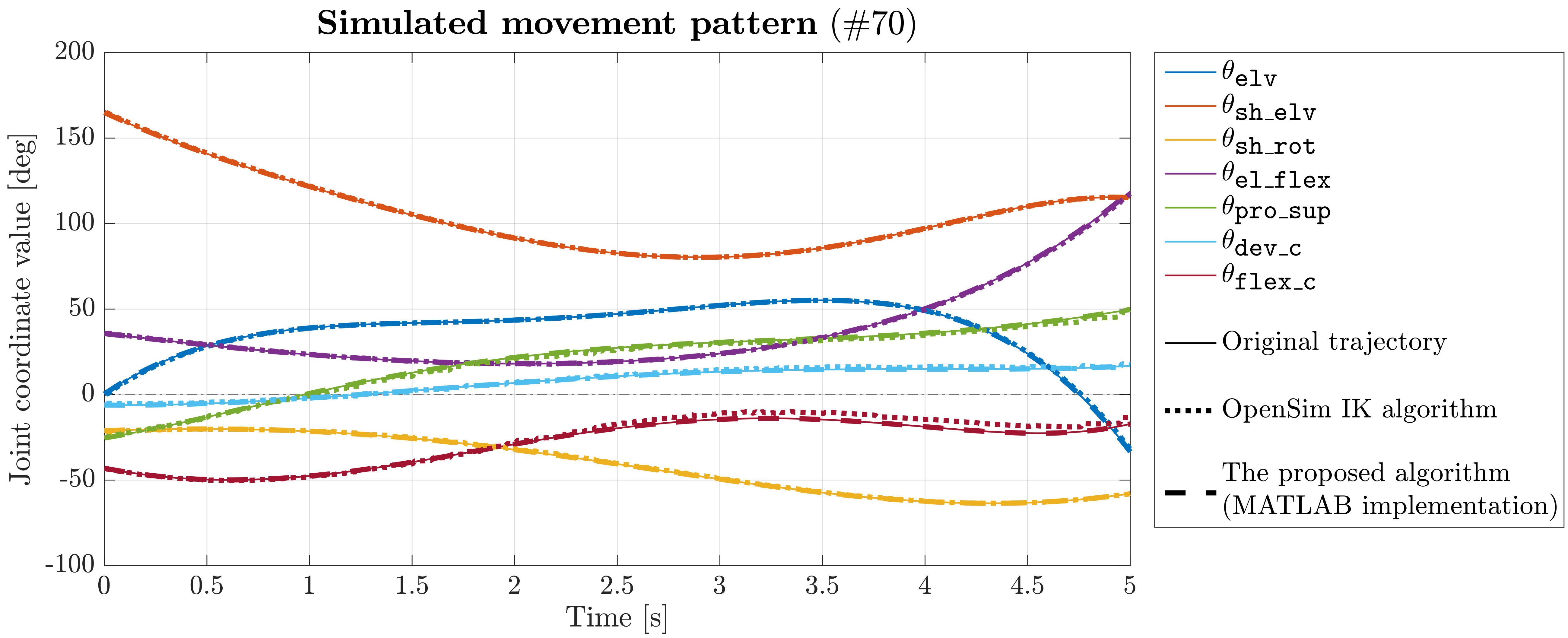

Simulated movement pattern (#70)
Joint coordinate value [deg]
Time [s]
200
150
100
50
0
-50
-100
0
0.5
1
1.5
2
2.5
3
3.5
4
4.5
5
$\theta_{\texttt{elv}}$
$\theta_{\texttt{sh\_elv}}$
$\theta_{\texttt{sh\_rot}}$
$\theta_{\texttt{el\_flex}}$
$\theta_{\texttt{pro\_sup}}$
$\theta_{\texttt{dev\_c}}$
$\theta_{\texttt{flex\_c}}$
Original trajectory
OpenSim IK algorithm
The proposed algorithm (MATLAB implementation)

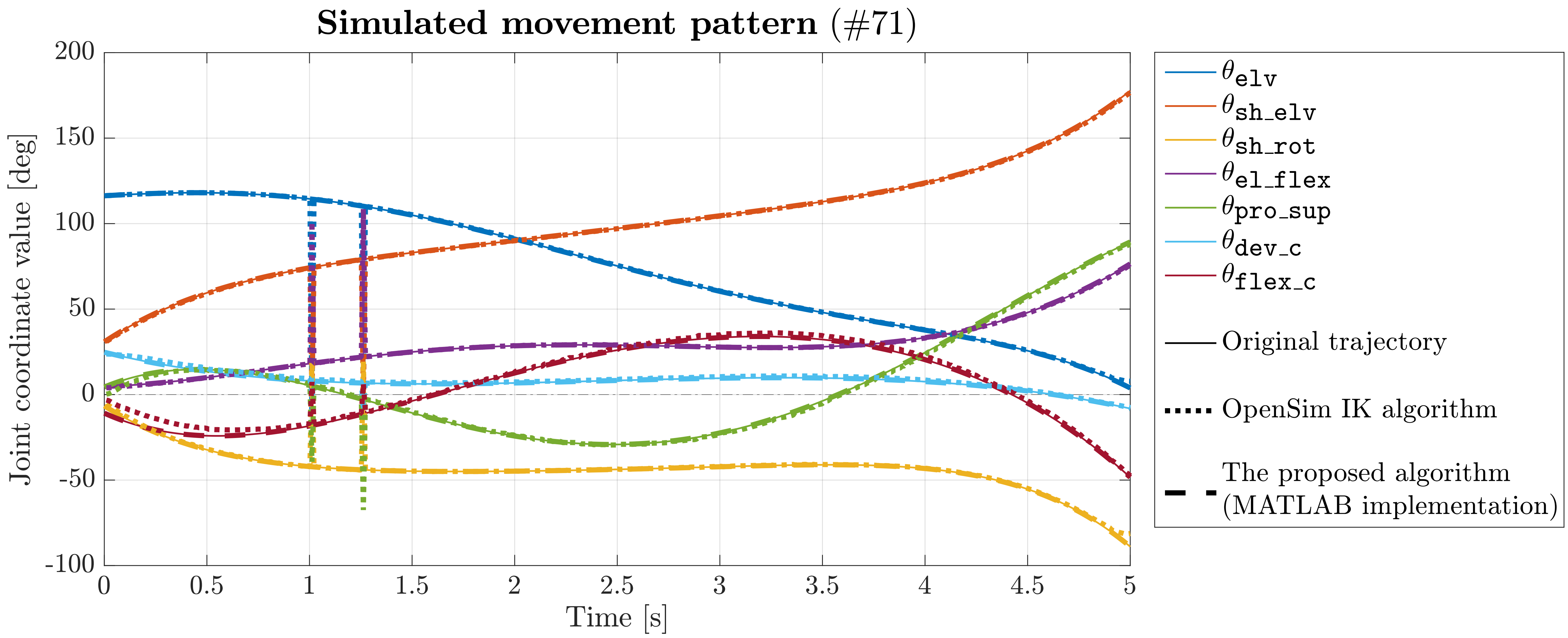

Simulated movement pattern (#71)
Joint coordinate value [deg]
Time [s]
200
150
100
50
0
-50
-100
0
0.5
1
1.5
2
2.5
3
3.5
4
4.5
5
θ_{elv}
θ_{sh_elv}
θ_{sh_rot}
θ_{el_flex}
θ_{pro_sup}
θ_{dev_c}
θ_{flex_c}
Original trajectory
OpenSim IK algorithm
The proposed algorithm (MATLAB implementation)

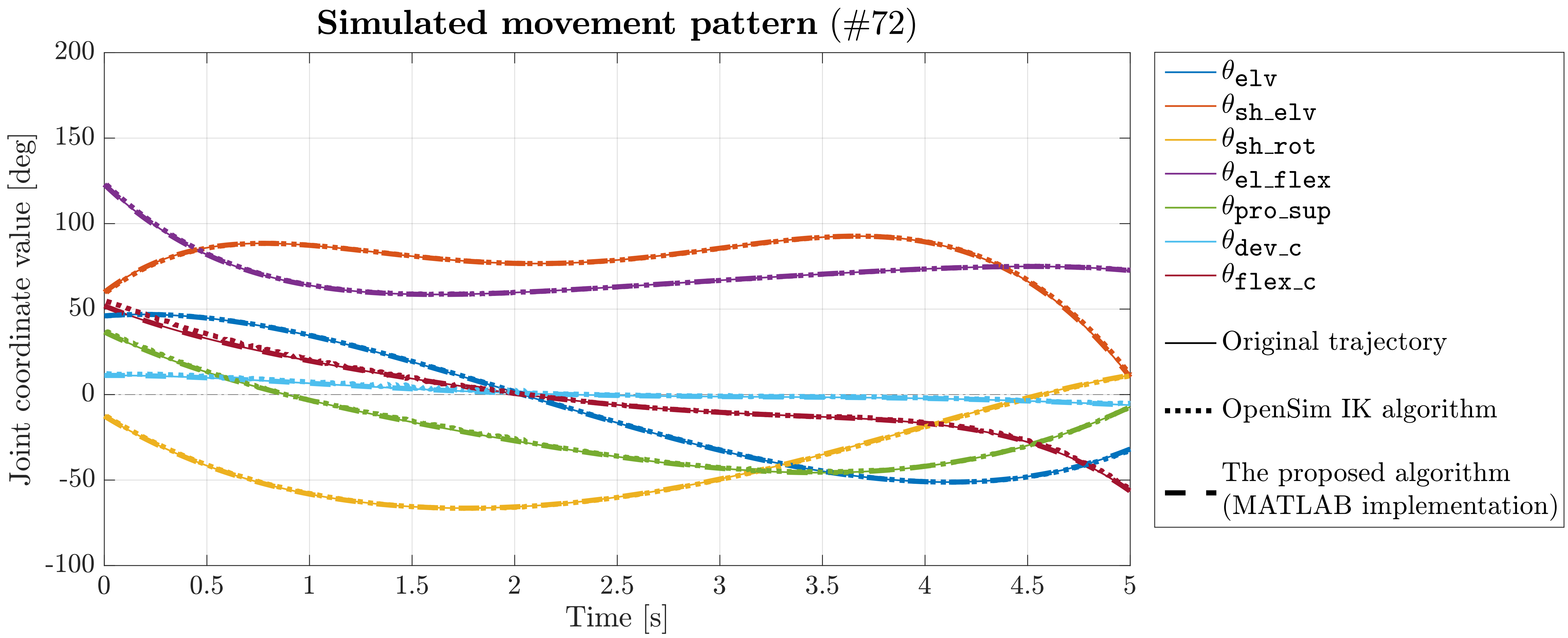

Simulated movement pattern (#72)
200
150
100
50
0
-50
-100
Joint coordinate value [deg]
0
0.5
1
1.5
2
2.5
3
3.5
4
4.5
5
Time [s]
θelv
θsh_elv
θsh_rot
θel_flex
θpro_sup
θdev_c
θflex_c
Original trajectory
OpenSim IK algorithm
The proposed algorithm
(MATLAB implementation)

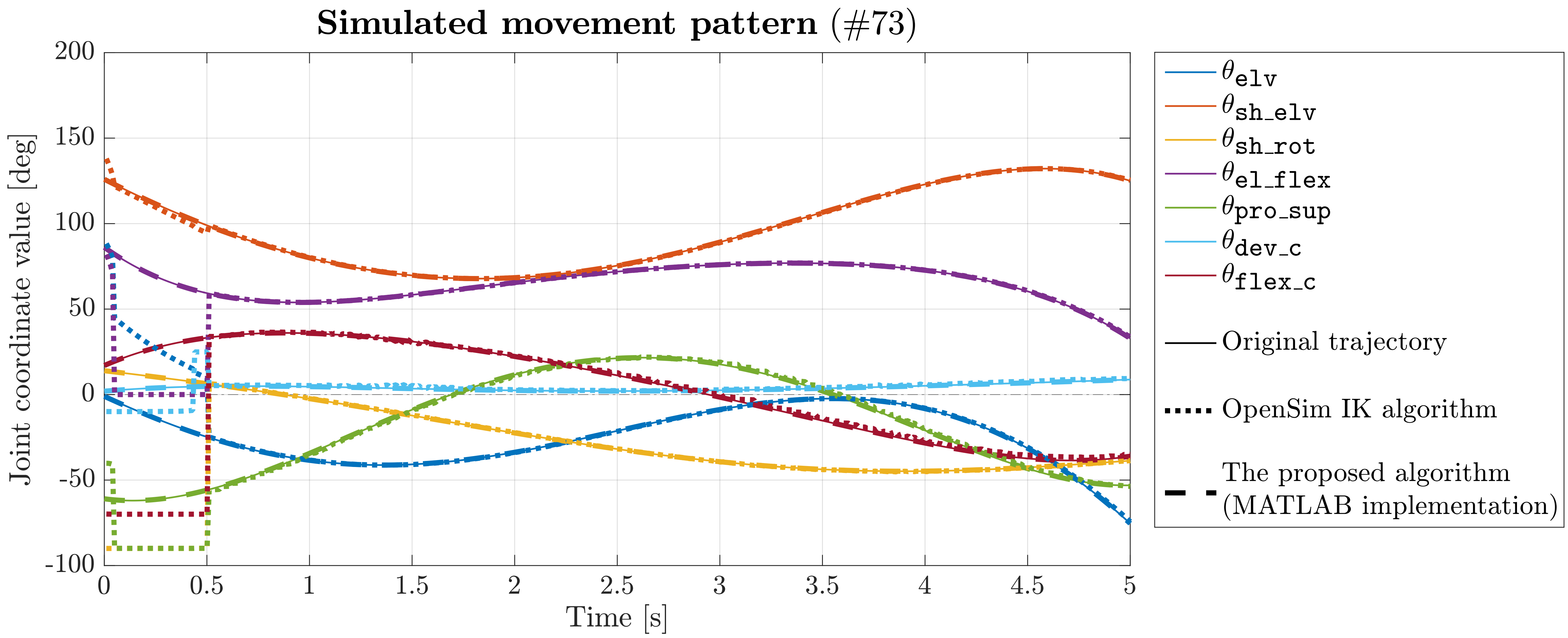
Simulated movement pattern (#73)
Joint coordinate value [deg]
Time [s]
200
150
100
50
0
-50
-100
0
0.5
1
1.5
2
2.5
3
3.5
4
4.5
5
$\theta_{\texttt{elv}}$
$\theta_{\texttt{sh\_elv}}$
$\theta_{\texttt{sh\_rot}}$
$\theta_{\texttt{el\_flex}}$
$\theta_{\texttt{pro\_sup}}$
$\theta_{\texttt{dev\_c}}$
$\theta_{\texttt{flex\_c}}$
Original trajectory
OpenSim IK algorithm
The proposed algorithm
(MATLAB implementation)

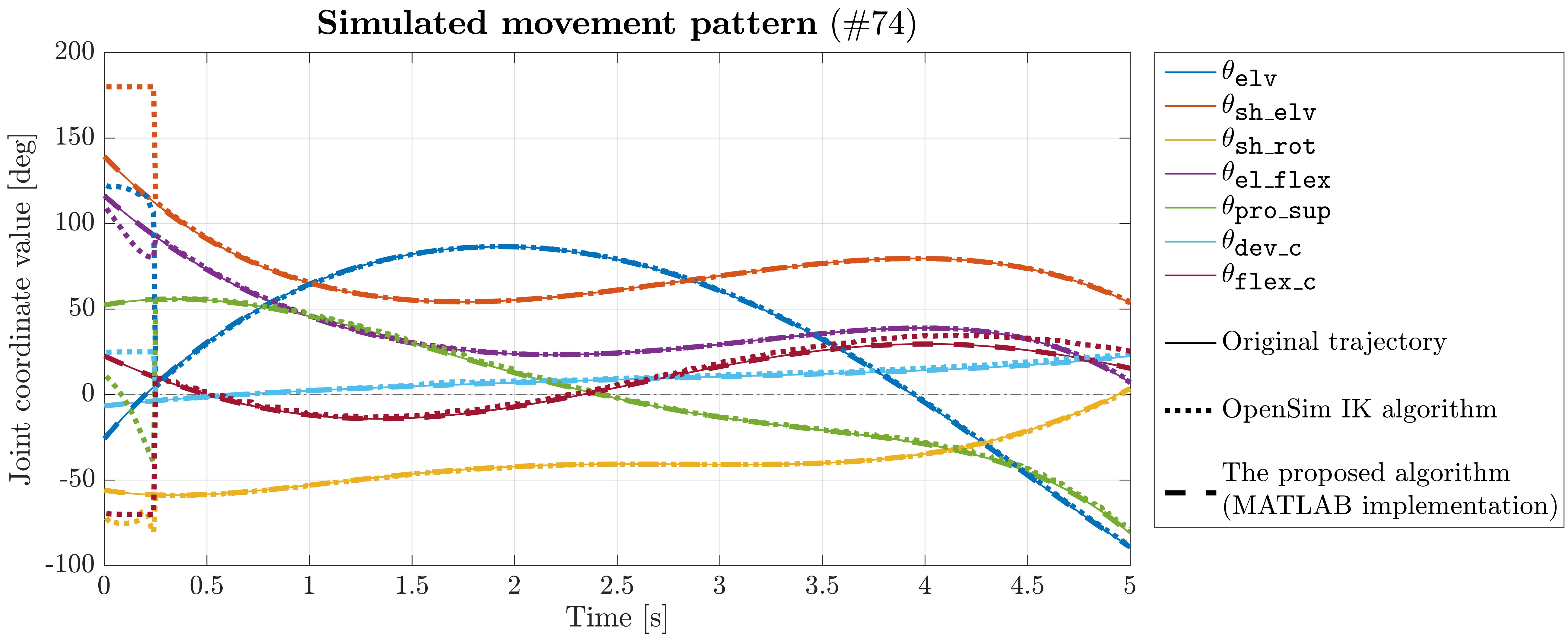

Simulated movement pattern (#74)
Joint coordinate value [deg]
Time [s]
200
150
100
50
0
-50
-100
0
0.5
1
1.5
2
2.5
3
3.5
4
4.5
5
$\theta_{\texttt{elv}}$
$\theta_{\texttt{sh\_elv}}$
$\theta_{\texttt{sh\_rot}}$
$\theta_{\texttt{el\_flex}}$
$\theta_{\texttt{pro\_sup}}$
$\theta_{\texttt{dev\_c}}$
$\theta_{\texttt{flex\_c}}$
Original trajectory
OpenSim IK algorithm
The proposed algorithm (MATLAB implementation)

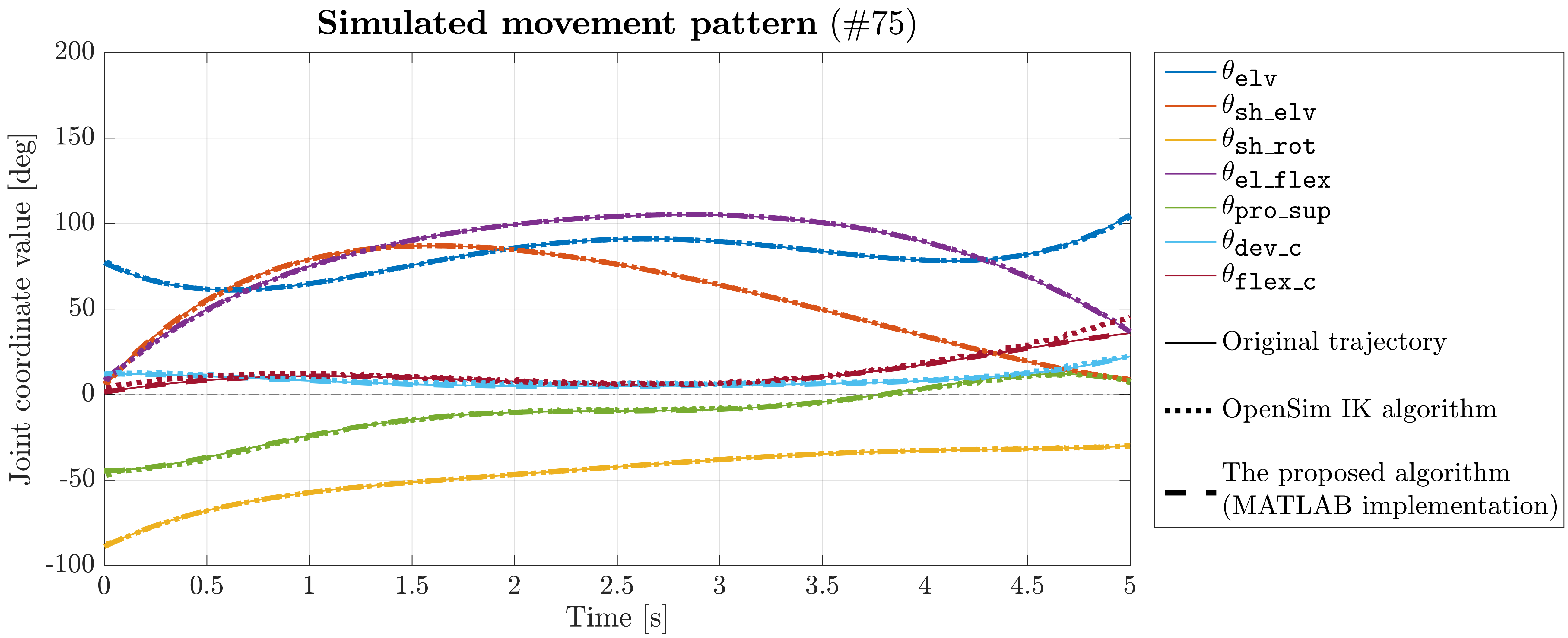

Simulated movement pattern (#75)
Joint coordinate value [deg]
200
150
100
50
0
-50
-100
0
0.5
1
1.5
2
2.5
3
3.5
4
4.5
5
Time [s]
θ_{elv}
θ_{sh_elv}
θ_{sh_rot}
θ_{el_flex}
θ_{pro_sup}
θ_{dev_c}
θ_{flex_c}
Original trajectory
OpenSim IK algorithm
The proposed algorithm (MATLAB implementation)

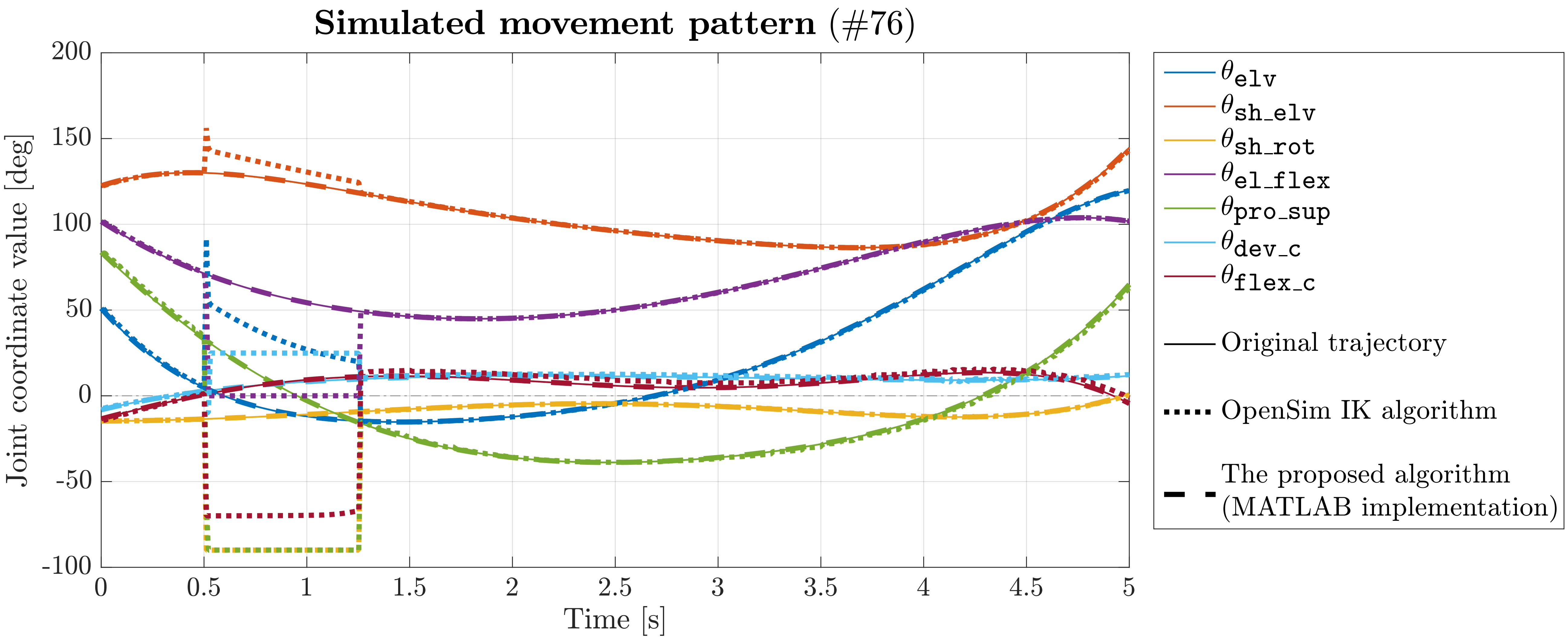

Simulated movement pattern (#76)
Joint coordinate value [deg]
Time [s]
200
150
100
50
0
-50
-100
0
0.5
1
1.5
2
2.5
3
3.5
4
4.5
5
$\theta_{\texttt{elv}}$
$\theta_{\texttt{sh\_elv}}$
$\theta_{\texttt{sh\_rot}}$
$\theta_{\texttt{el\_flex}}$
$\theta_{\texttt{pro\_sup}}$
$\theta_{\texttt{dev\_c}}$
$\theta_{\texttt{flex\_c}}$
Original trajectory
OpenSim IK algorithm
The proposed algorithm (MATLAB implementation)

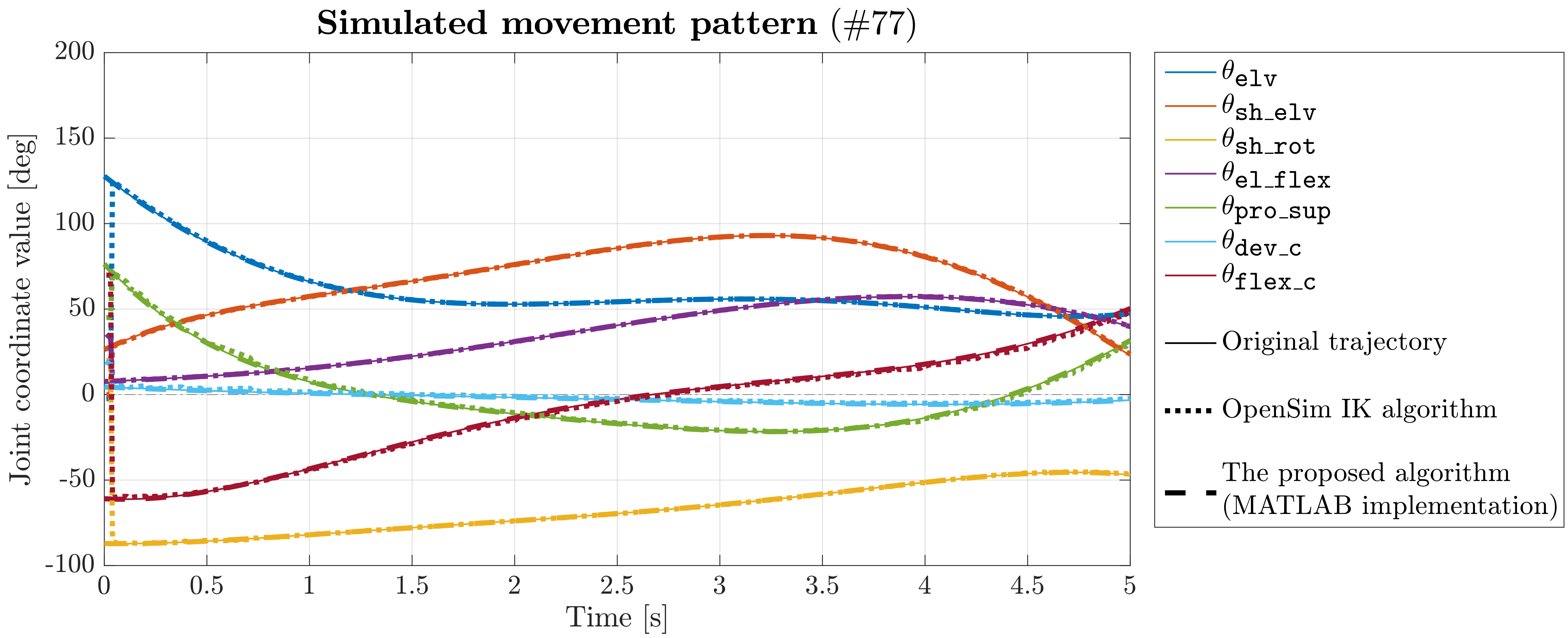

Simulated movement pattern (#77)
200
150
100
50
0
-50
-100
Joint coordinate value [deg]
0
0.5
1
1.5
2
2.5
3
3.5
4
4.5
5
Time [s]
θ_{elv}
θ_{sh_elv}
θ_{sh_rot}
θ_{el_flex}
θ_{pro_sup}
θ_{dev_c}
θ_{flex_c}
Original trajectory
OpenSim IK algorithm
The proposed algorithm
(MATLAB implementation)

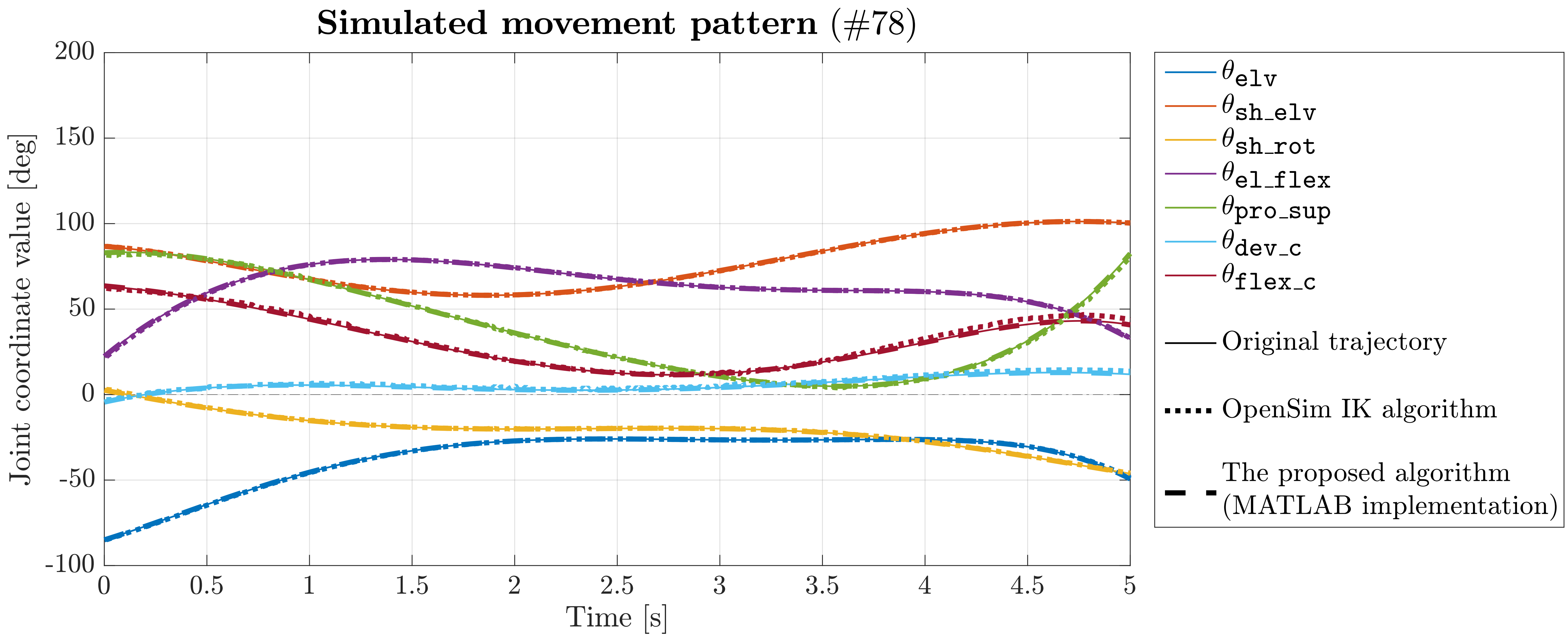

Simulated movement pattern (#78)
200
150
100
50
0
-50
-100
Joint coordinate value [deg]
0
0.5
1
1.5
2
2.5
3
3.5
4
4.5
5
Time [s]
θelv
θsh_elv
θsh_rot
θel_flex
θpro_sup
θdev_c
θflex_c
Original trajectory
OpenSim IK algorithm
The proposed algorithm (MATLAB implementation)

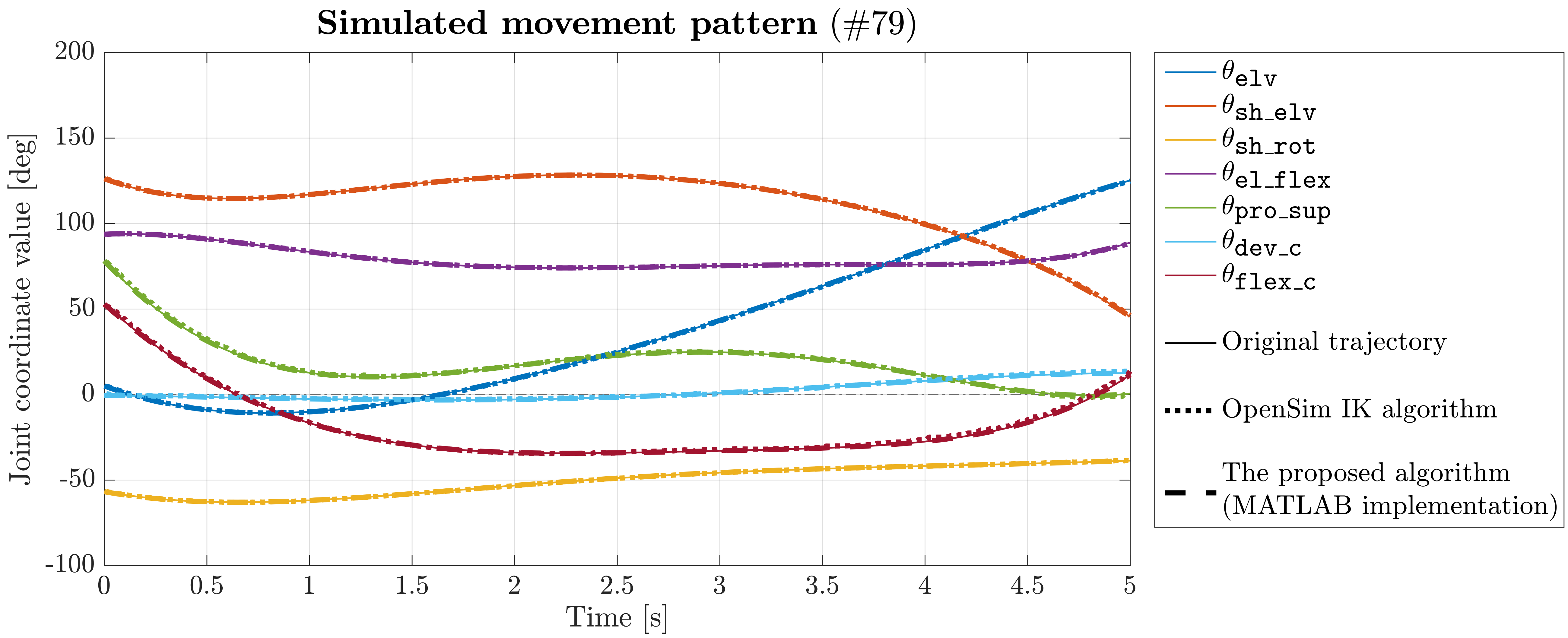

Simulated movement pattern (#79)
Joint coordinate value [deg]
200
150
100
50
0
-50
-100
0
0.5
1
1.5
2
2.5
3
3.5
4
4.5
5
Time [s]
θ_{elv}
θ_{sh_elv}
θ_{sh_rot}
θ_{el_flex}
θ_{pro_sup}
θ_{dev_c}
θ_{flex_c}
Original trajectory
OpenSim IK algorithm
The proposed algorithm (MATLAB implementation)

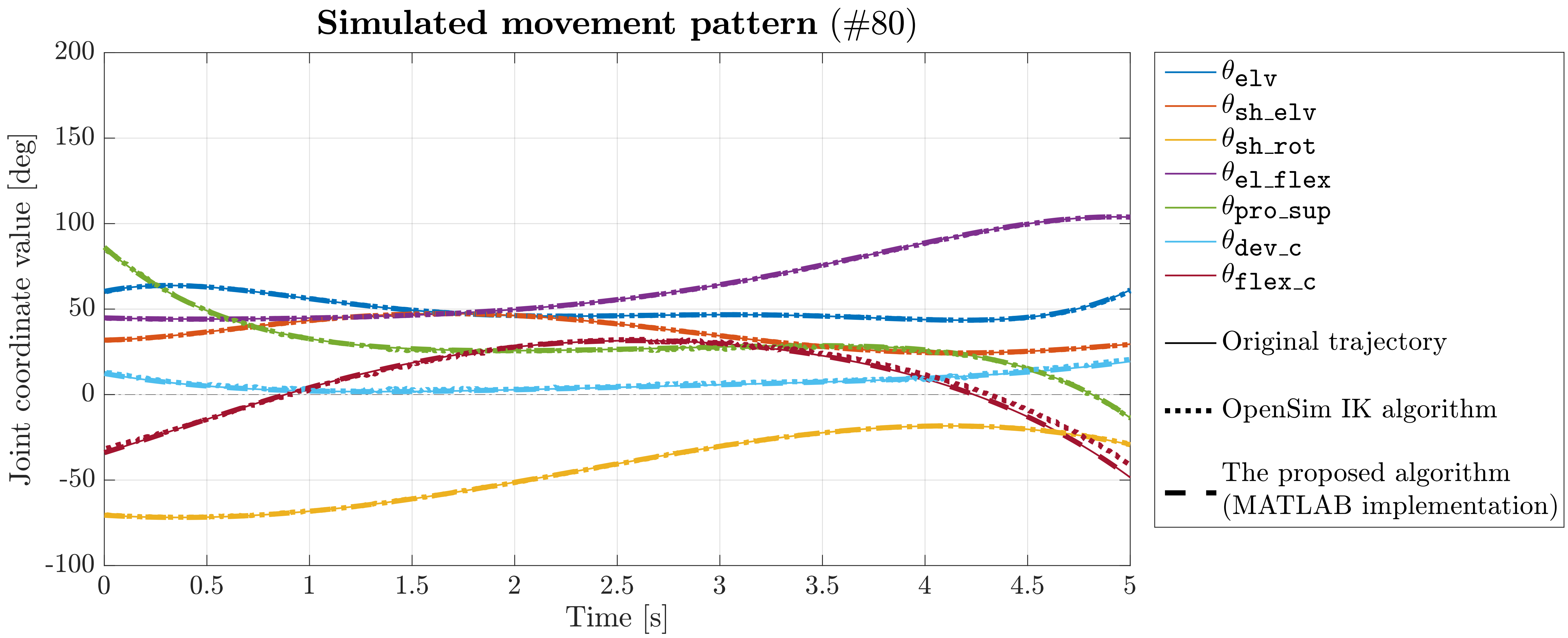

Simulated movement pattern (#80)
200
150
100
50
0
-50
-100
Joint coordinate value [deg]
0
0.5
1
1.5
2
2.5
3
3.5
4
4.5
5
Time [s]
θelv
θsh_elv
θsh_rot
θel_flex
θpro_sup
θdev_c
θflex_c
Original trajectory
OpenSim IK algorithm
The proposed algorithm (MATLAB implementation)

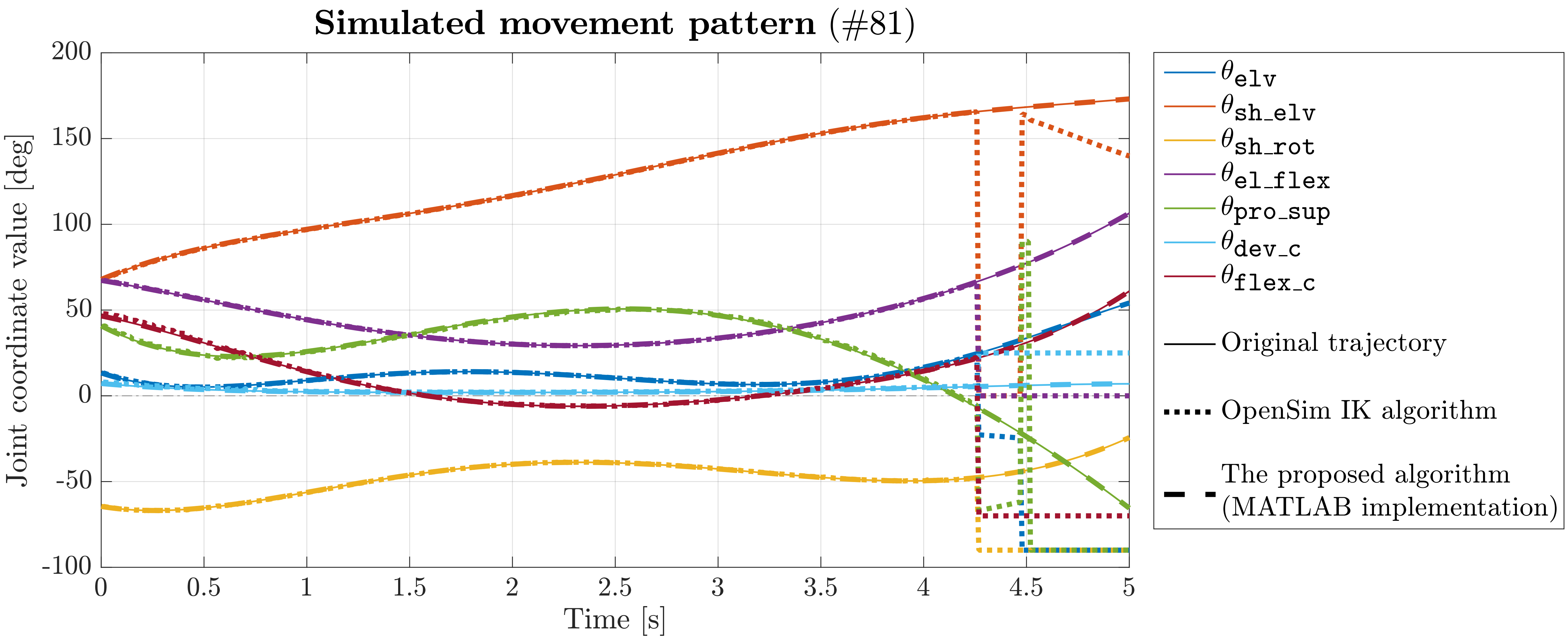

Simulated movement pattern (#81)
Joint coordinate value [deg]
200
150
100
50
0
-50
-100
0
0.5
1
1.5
2
2.5
3
3.5
4
4.5
5
Time [s]
θ_{elv}
θ_{sh_elv}
θ_{sh_rot}
θ_{el_flex}
θ_{pro_sup}
θ_{dev_c}
θ_{flex_c}
Original trajectory
OpenSim IK algorithm
The proposed algorithm (MATLAB implementation)

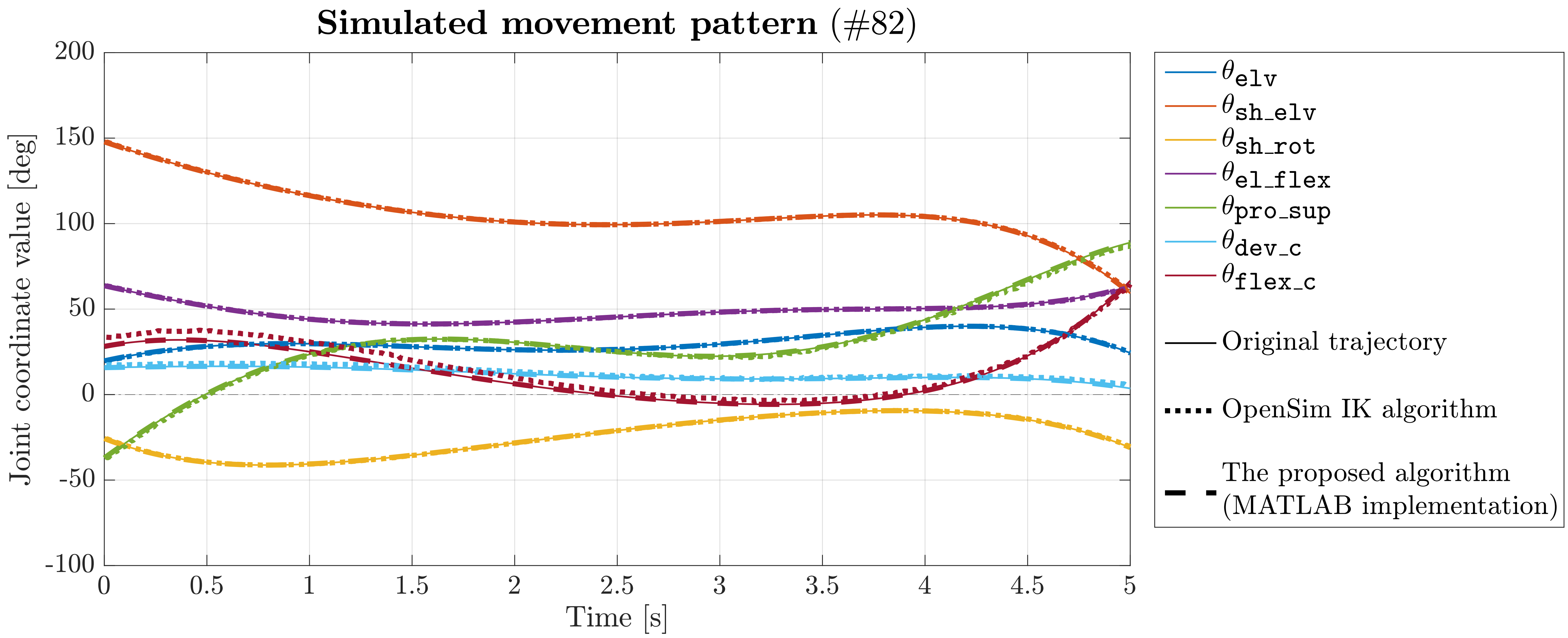

Simulated movement pattern (#82)
200
150
100
50
0
-50
-100
Joint coordinate value [deg]
0
0.5
1
1.5
2
2.5
3
3.5
4
4.5
5
Time [s]
$\theta_{\mathtt{elv}}$
$\theta_{\mathtt{sh\_elv}}$
$\theta_{\mathtt{sh\_rot}}$
$\theta_{\mathtt{el\_flex}}$
$\theta_{\mathtt{pro\_sup}}$
$\theta_{\mathtt{dev\_c}}$
$\theta_{\mathtt{flex\_c}}$
Original trajectory
OpenSim IK algorithm
The proposed algorithm
(MATLAB implementation)

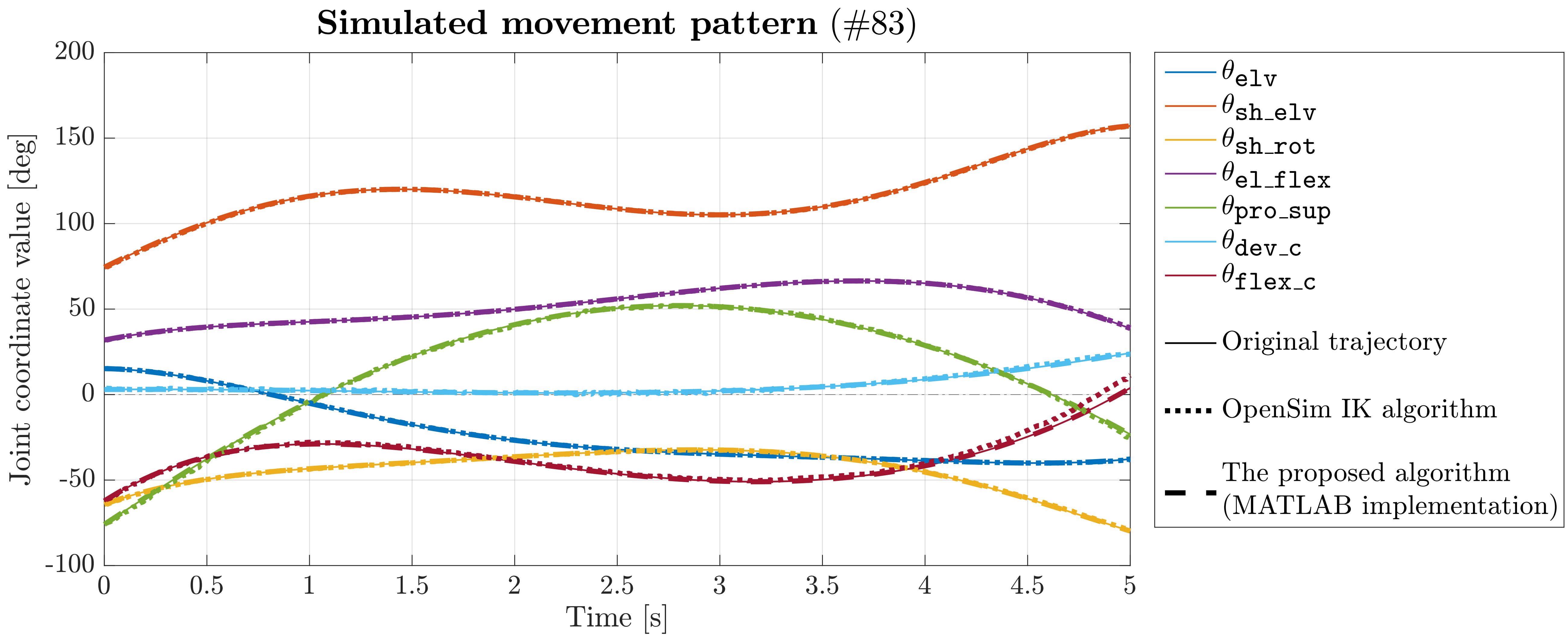

Simulated movement pattern (#83)
200
150
100
50
0
-50
-100
Joint coordinate value [deg]
0
0.5
1
1.5
2
2.5
3
3.5
4
4.5
5
Time [s]
$\theta_{\texttt{elv}}$
$\theta_{\texttt{sh\_elv}}$
$\theta_{\texttt{sh\_rot}}$
$\theta_{\texttt{el\_flex}}$
$\theta_{\texttt{pro\_sup}}$
$\theta_{\texttt{dev\_c}}$
$\theta_{\texttt{flex\_c}}$
Original trajectory
OpenSim IK algorithm
The proposed algorithm
(MATLAB implementation)

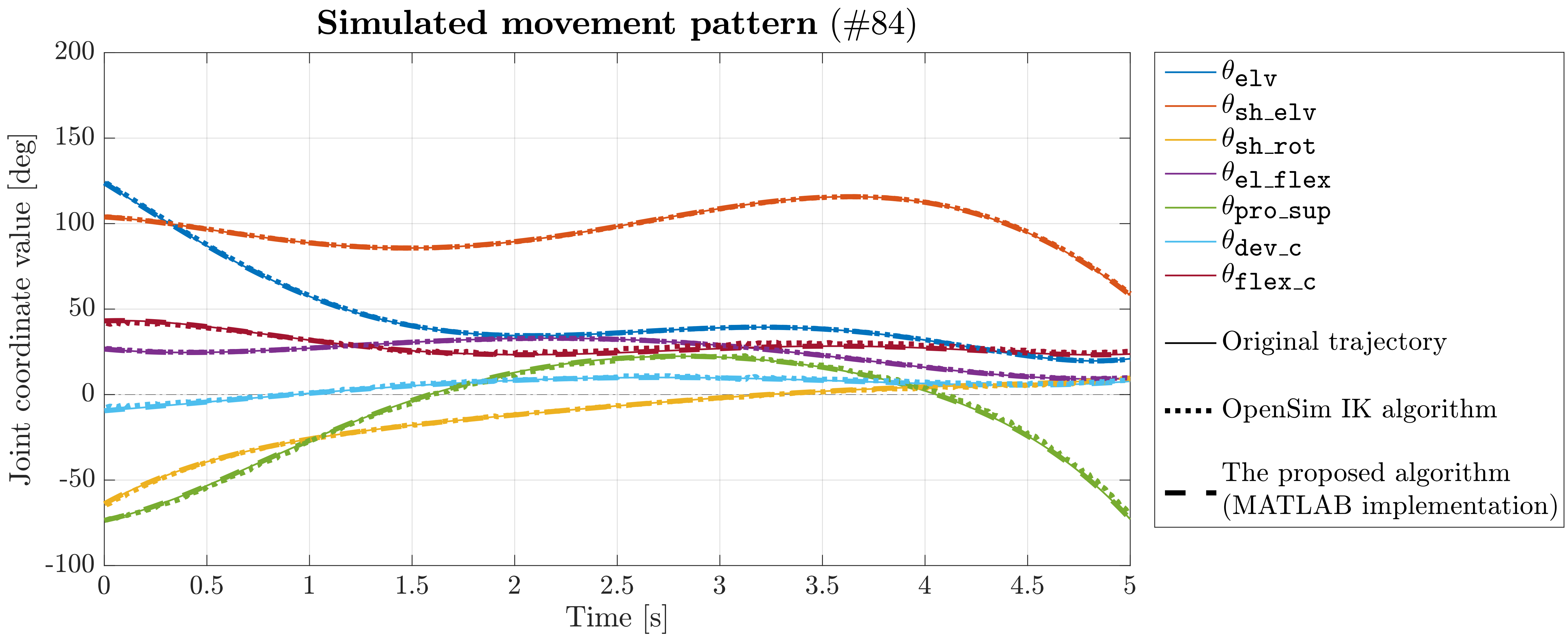

Simulated movement pattern (#84)
Joint coordinate value [deg]
200
150
100
50
0
-50
-100
0
0.5
1
1.5
2
2.5
3
3.5
4
4.5
5
Time [s]
θelv
θsh_elv
θsh_rot
θel_flex
θpro_sup
θdev_c
θflex_c
Original trajectory
OpenSim IK algorithm
The proposed algorithm
(MATLAB implementation)

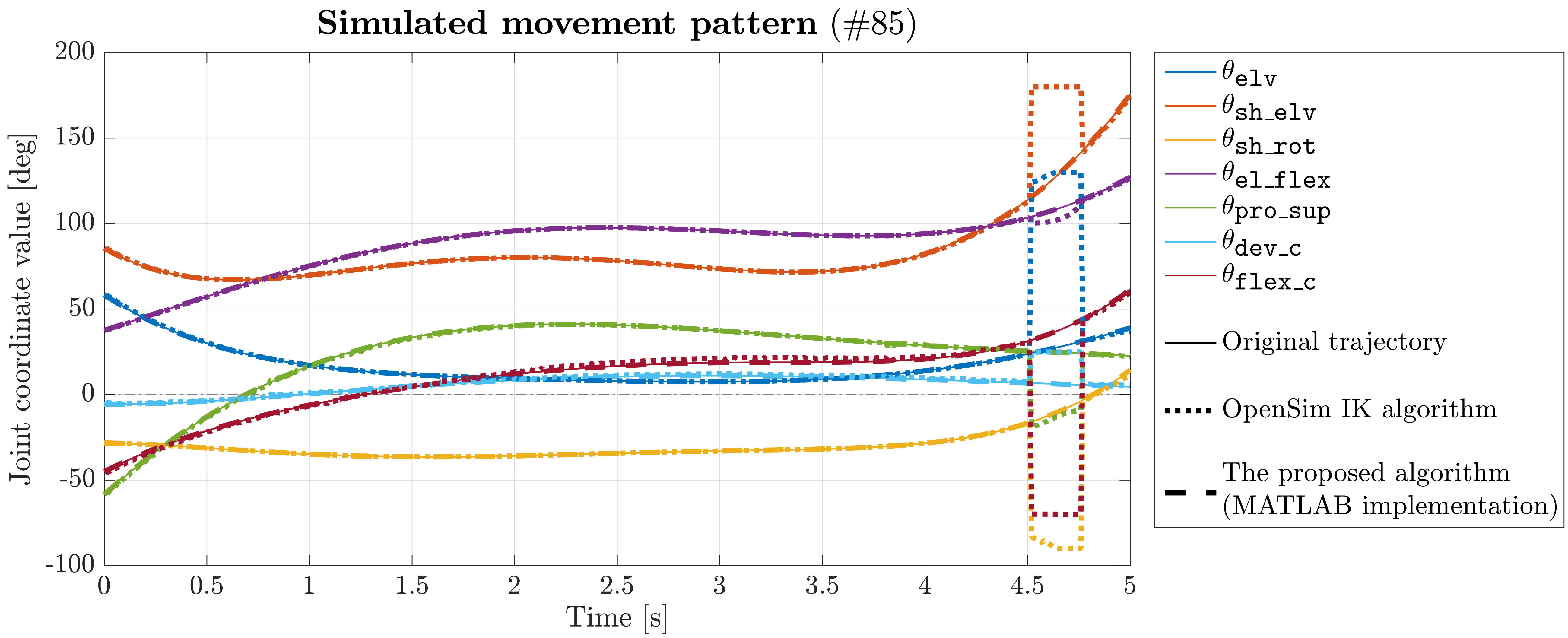

Simulated movement pattern (#85)
Joint coordinate value [deg]
200
150
100
50
0
-50
-100
0
0.5
1
1.5
2
2.5
3
3.5
4
4.5
5
Time [s]
$\theta_{\texttt{elv}}$
$\theta_{\texttt{sh\_elv}}$
$\theta_{\texttt{sh\_rot}}$
$\theta_{\texttt{el\_flex}}$
$\theta_{\texttt{pro\_sup}}$
$\theta_{\texttt{dev\_c}}$
$\theta_{\texttt{flex\_c}}$
Original trajectory
OpenSim IK algorithm
The proposed algorithm (MATLAB implementation)

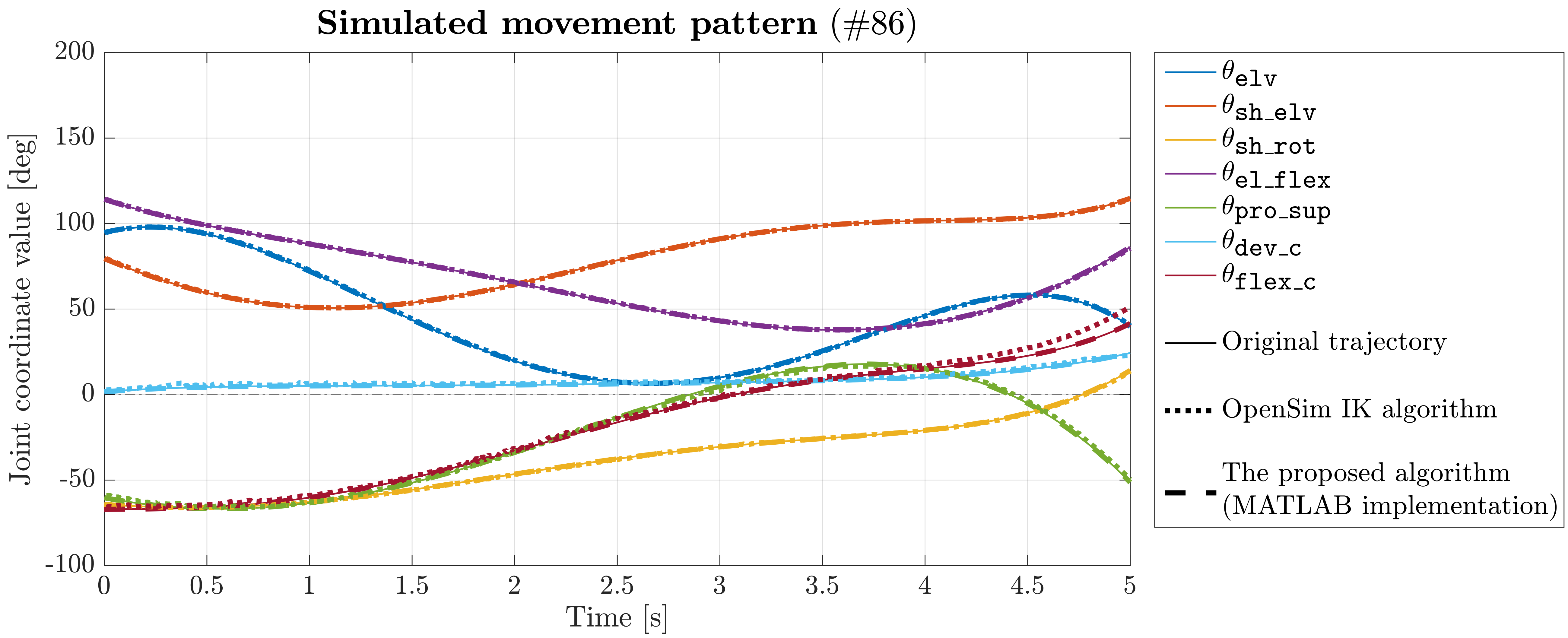

Simulated movement pattern (#86)
200
150
100
50
0
-50
-100
Joint coordinate value [deg]
0
0.5
1
1.5
2
2.5
3
3.5
4
4.5
5
Time [s]
θ_{elv}
θ_{sh_elv}
θ_{sh_rot}
θ_{el_flex}
θ_{pro_sup}
θ_{dev_c}
θ_{flex_c}
Original trajectory
OpenSim IK algorithm
The proposed algorithm (MATLAB implementation)

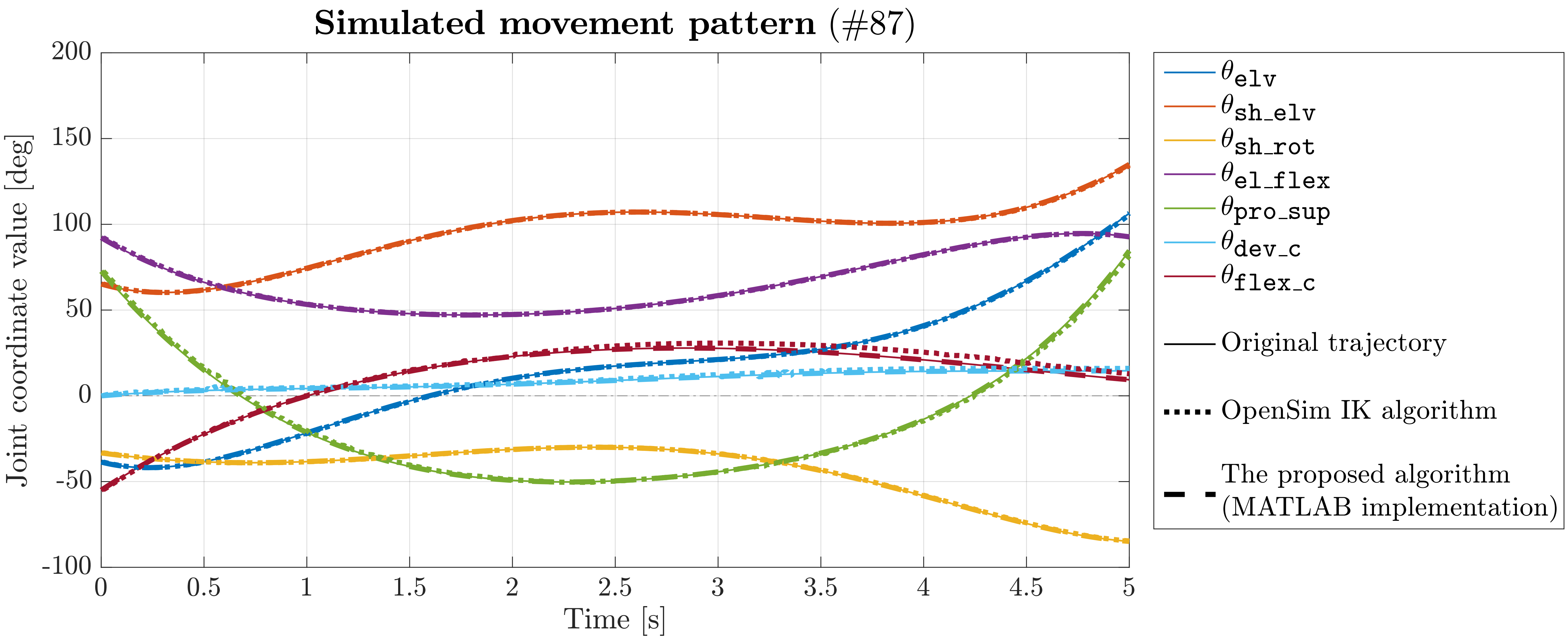

Simulated movement pattern (#87)
200
150
100
50
0
-50
-100
Joint coordinate value [deg]
0
0.5
1
1.5
2
2.5
3
3.5
4
4.5
5
Time [s]
θelv
θsh_elv
θsh_rot
θel_flex
θpro_sup
θdev_c
θflex_c
Original trajectory
OpenSim IK algorithm
The proposed algorithm
(MATLAB implementation)

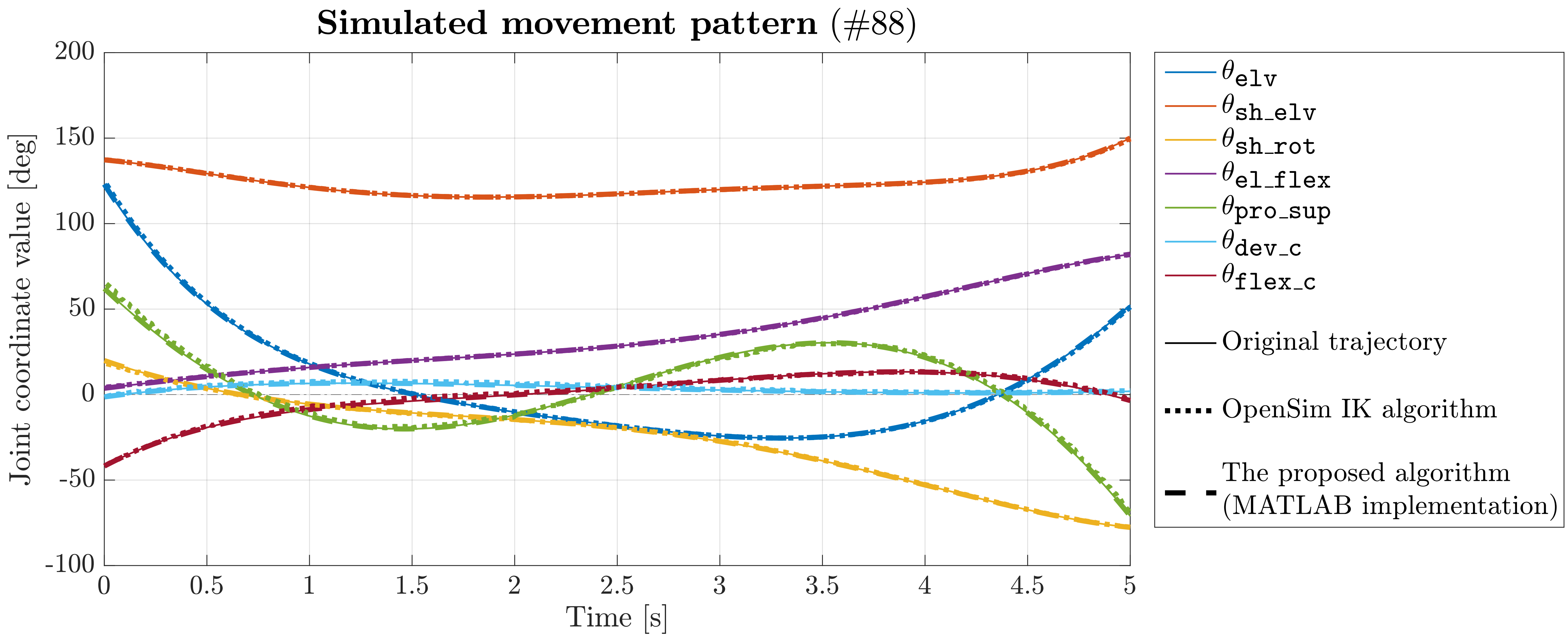

Simulated movement pattern (#88)
200
150
100
50
0
-50
-100
Joint coordinate value [deg]
0
0.5
1
1.5
2
2.5
3
3.5
4
4.5
5
Time [s]
θelv
θsh_elv
θsh_rot
θel_flex
θpro_sup
θdev_c
θflex_c
Original trajectory
OpenSim IK algorithm
The proposed algorithm
(MATLAB implementation)

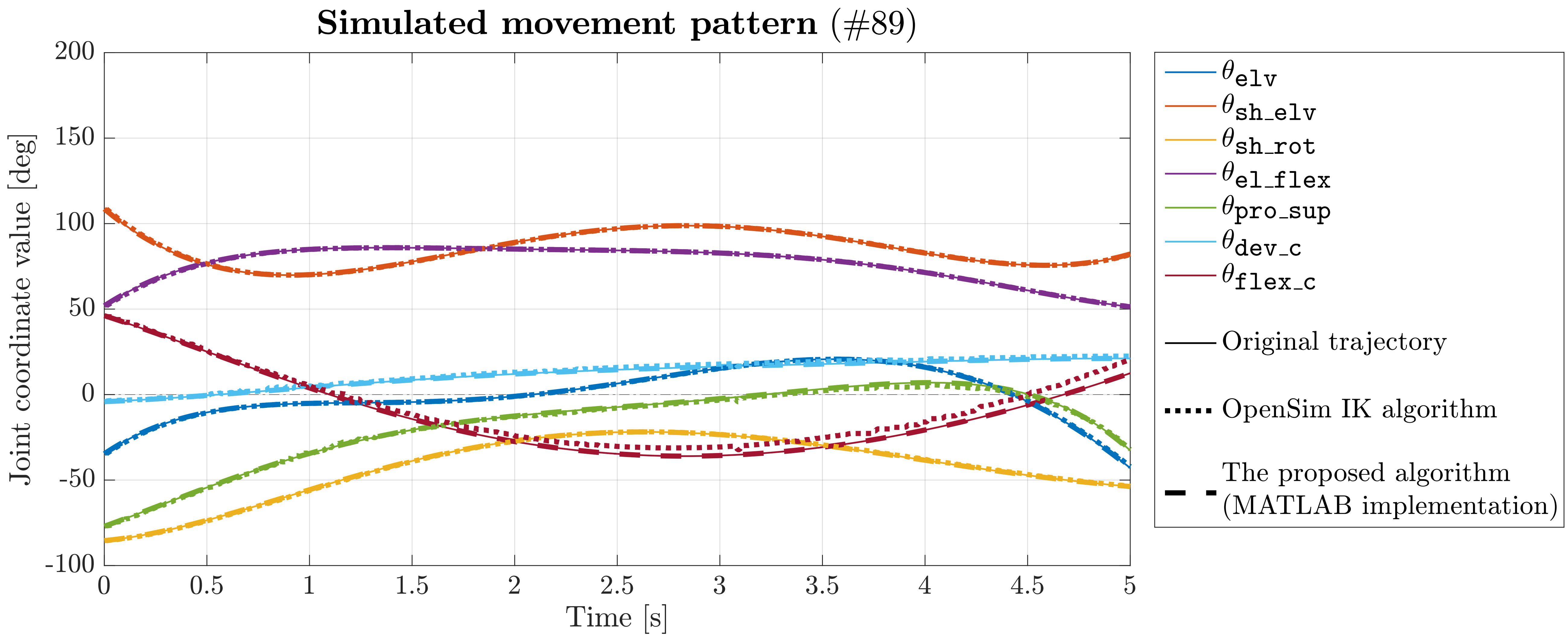

Simulated movement pattern (#89)
200
150
100
50
0
-50
-100
Joint coordinate value [deg]
0
0.5
1
1.5
2
2.5
3
3.5
4
4.5
5
Time [s]
θelv
θsh_elv
θsh_rot
θel_flex
θpro_sup
θdev_c
θflex_c
Original trajectory
OpenSim IK algorithm
The proposed algorithm
(MATLAB implementation)

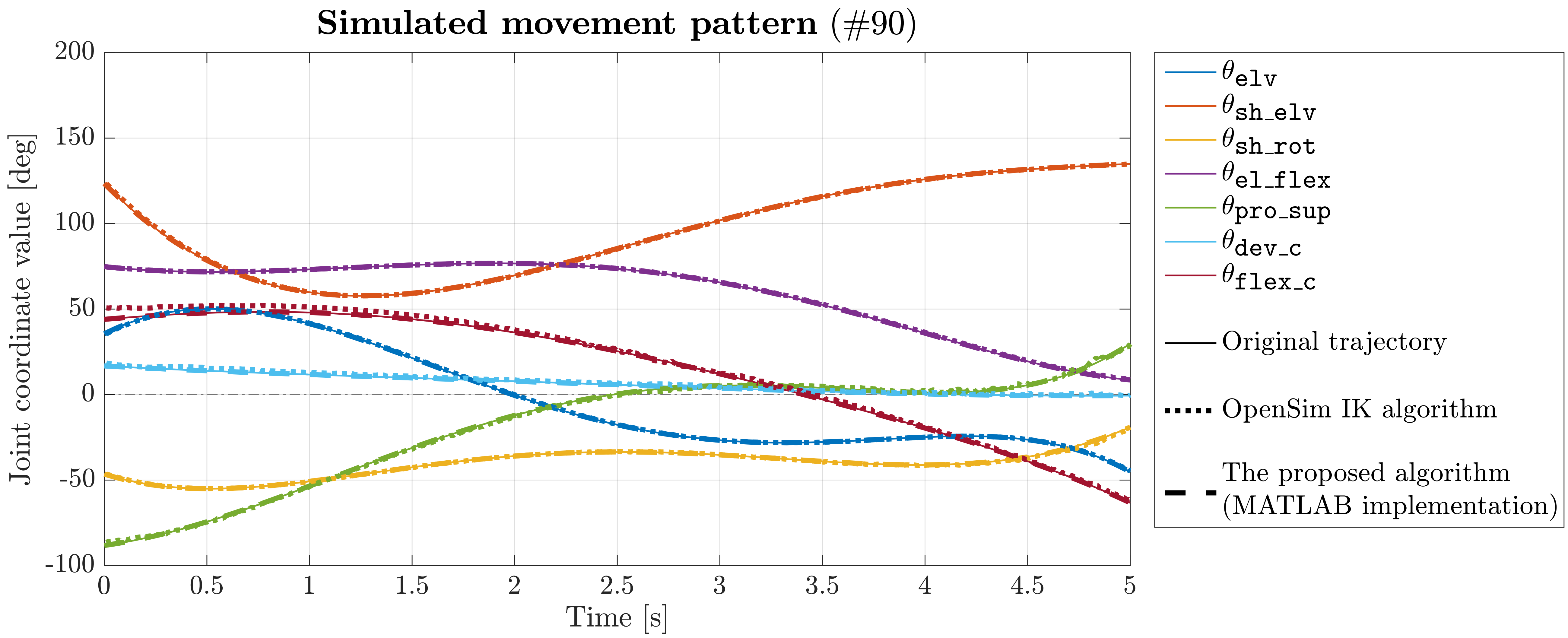
Simulated movement pattern (#90)
200
150
100
50
0
-50
-100
Joint coordinate value [deg]
0
0.5
1
1.5
2
2.5
3
3.5
4
4.5
5
Time [s]
θ_{elv}
θ_{sh_elv}
θ_{sh_rot}
θ_{el_flex}
θ_{pro_sup}
θ_{dev_c}
θ_{flex_c}
Original trajectory
OpenSim IK algorithm
The proposed algorithm (MATLAB implementation)

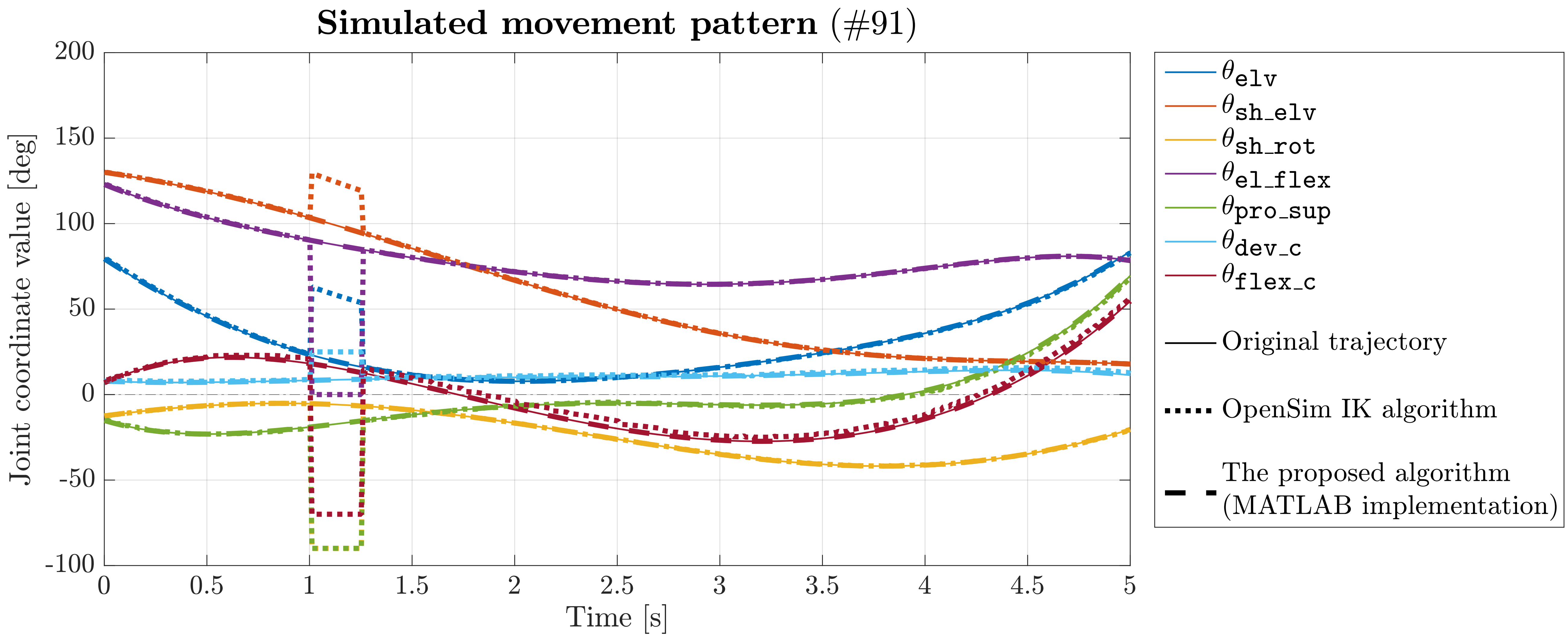
Simulated movement pattern (#91)
200
150
100
50
0
-50
-100
Joint coordinate value [deg]
0
0.5
1
1.5
2
2.5
3
3.5
4
4.5
5
Time [s]
θelv
θsh_elv
θsh_rot
θel_flex
θpro_sup
θdev_c
θflex_c
Original trajectory
OpenSim IK algorithm
The proposed algorithm
(MATLAB implementation)

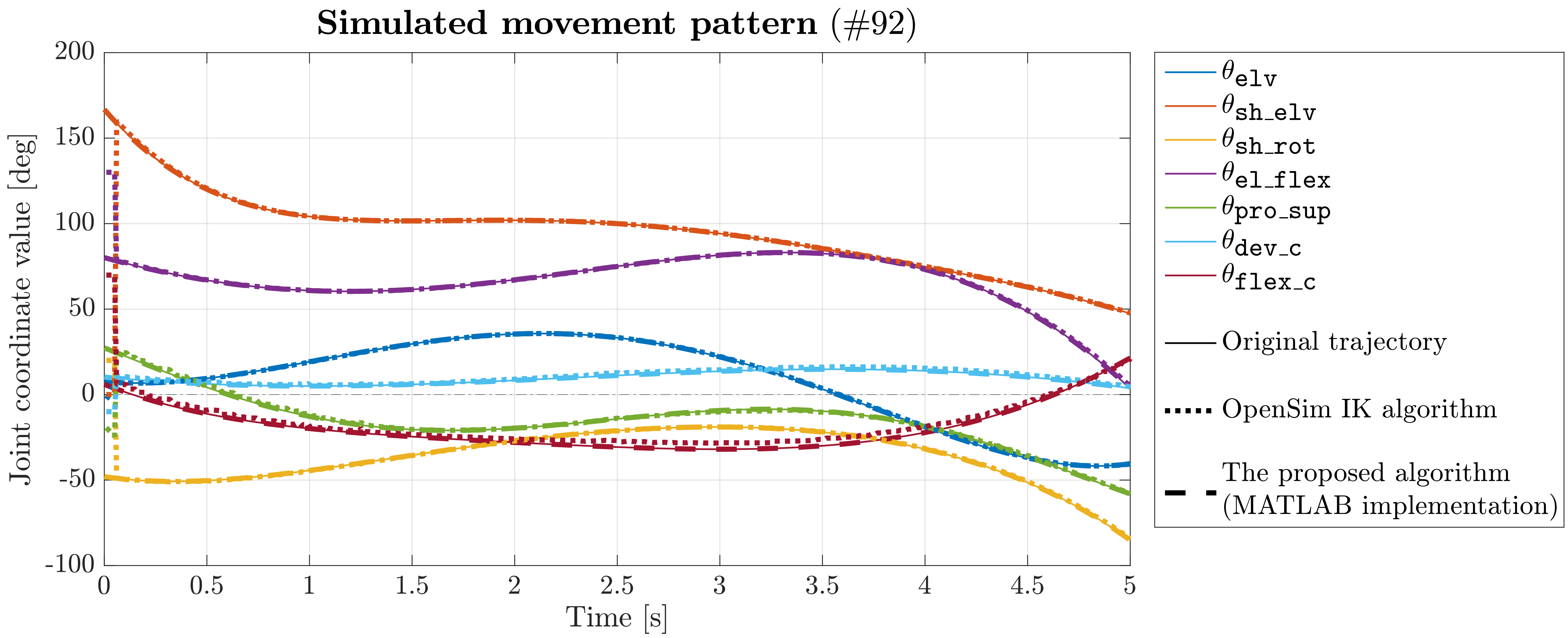

Simulated movement pattern (#92)
Joint coordinate value [deg]
200
150
100
50
0
-50
-100
0
0.5
1
1.5
2
2.5
3
3.5
4
4.5
5
Time [s]
$\theta_{\texttt{elv}}$
$\theta_{\texttt{sh\_elv}}$
$\theta_{\texttt{sh\_rot}}$
$\theta_{\texttt{el\_flex}}$
$\theta_{\texttt{pro\_sup}}$
$\theta_{\texttt{dev\_c}}$
$\theta_{\texttt{flex\_c}}$
Original trajectory
OpenSim IK algorithm
The proposed algorithm (MATLAB implementation)

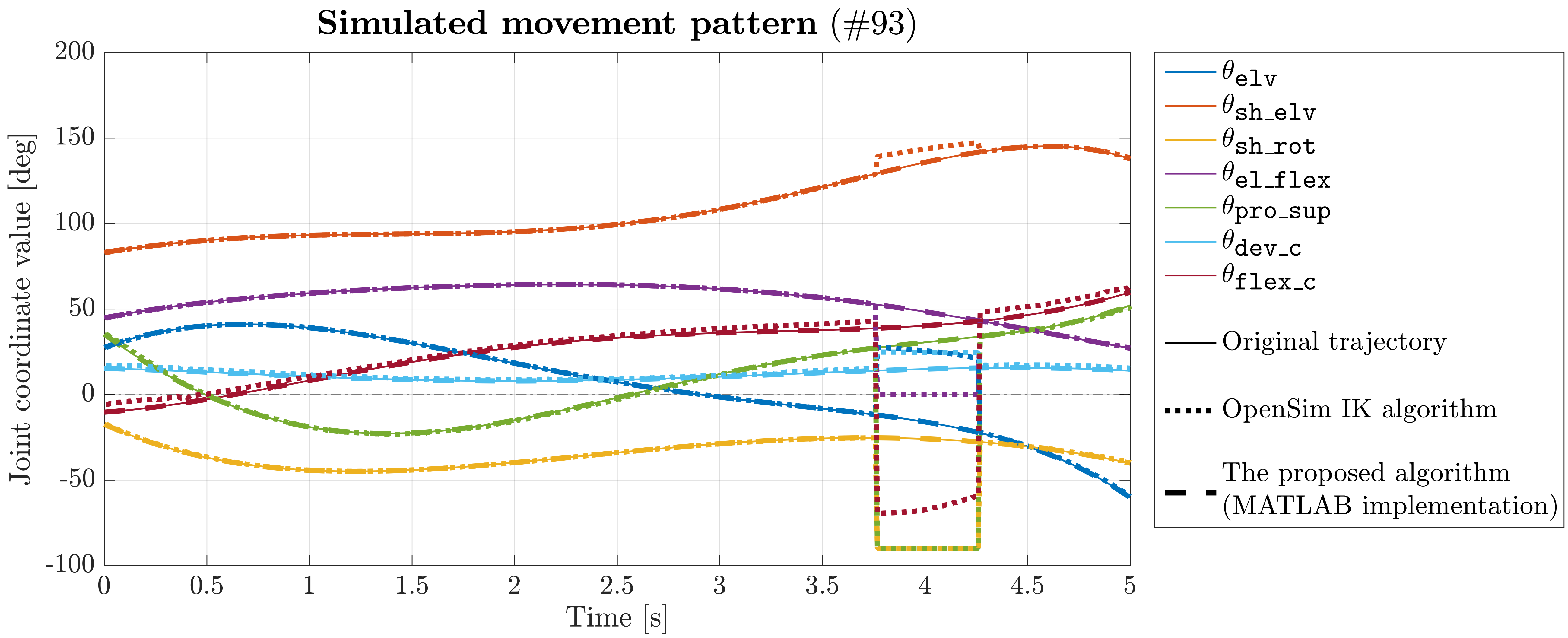

Simulated movement pattern (#93)
200
150
100
50
0
-50
-100
Joint coordinate value [deg]
0
0.5
1
1.5
2
2.5
3
3.5
4
4.5
5
Time [s]
θelv
θsh_elv
θsh_rot
θel_flex
θpro_sup
θdev_c
θflex_c
Original trajectory
OpenSim IK algorithm
The proposed algorithm
(MATLAB implementation)

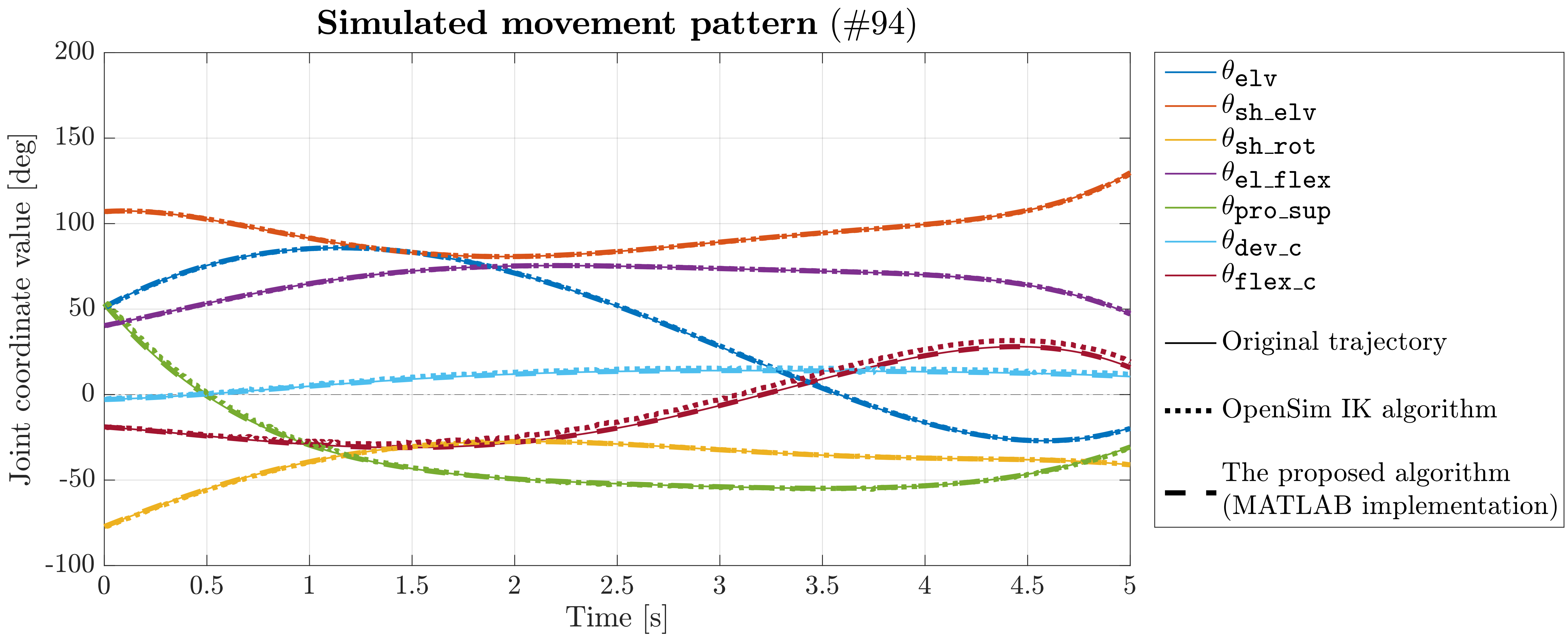

Simulated movement pattern (#94)
200
150
100
50
0
-50
-100
Joint coordinate value [deg]
0
0.5
1
1.5
2
2.5
3
3.5
4
4.5
5
Time [s]
θelv
θsh_elv
θsh_rot
θel_flex
θpro_sup
θdev_c
θflex_c
Original trajectory
OpenSim IK algorithm
The proposed algorithm
(MATLAB implementation)

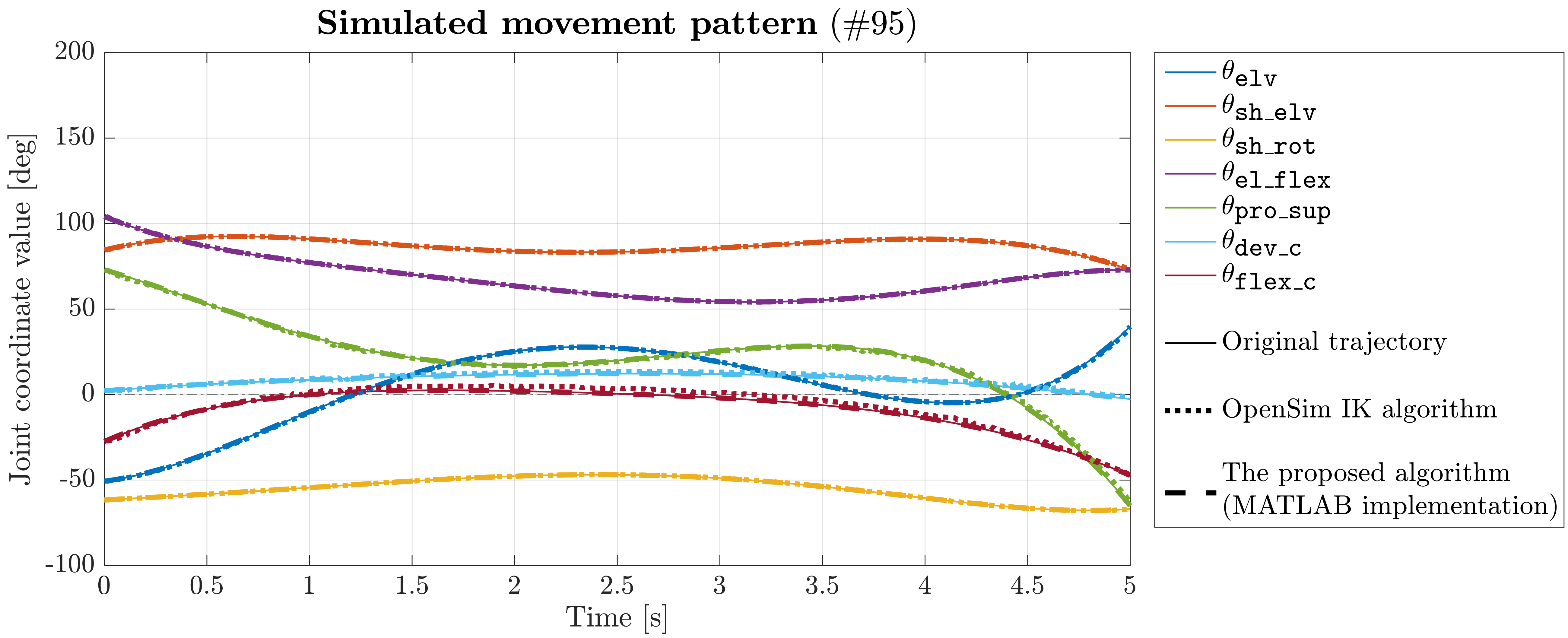

Simulated movement pattern (#95)
Joint coordinate value [deg]
Time [s]
200
150
100
50
0
-50
-100
0
0.5
1
1.5
2
2.5
3
3.5
4
4.5
5
$\theta_{\mathtt{elv}}$
$\theta_{\mathtt{sh\_elv}}$
$\theta_{\mathtt{sh\_rot}}$
$\theta_{\mathtt{el\_flex}}$
$\theta_{\mathtt{pro\_sup}}$
$\theta_{\mathtt{dev\_c}}$
$\theta_{\mathtt{flex\_c}}$
Original trajectory
OpenSim IK algorithm
The proposed algorithm
(MATLAB implementation)

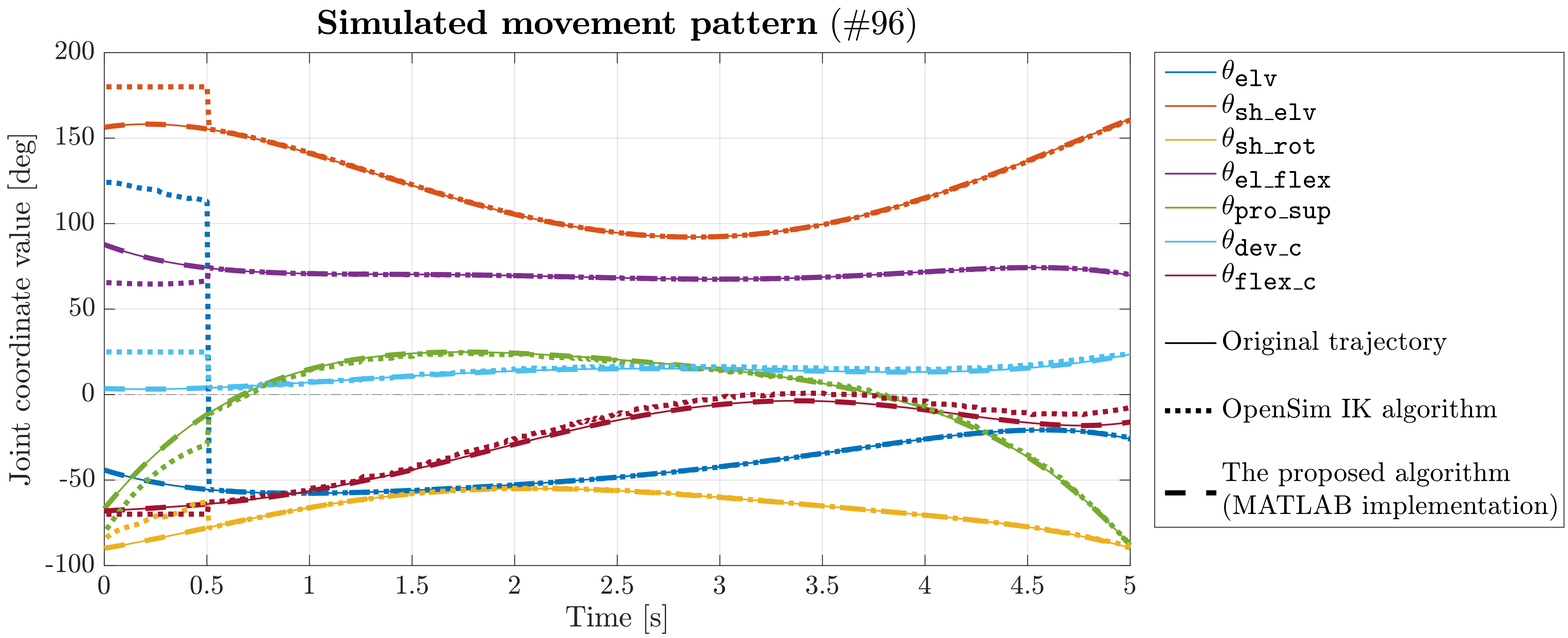

Simulated movement pattern (#96)
Joint coordinate value [deg]
200
150
100
50
0
-50
-100
0
0.5
1
1.5
2
2.5
3
3.5
4
4.5
5
Time [s]
θelv
θsh_elv
θsh_rot
θel_flex
θpro_sup
θdev_c
θflex_c
Original trajectory
OpenSim IK algorithm
The proposed algorithm (MATLAB implementation)

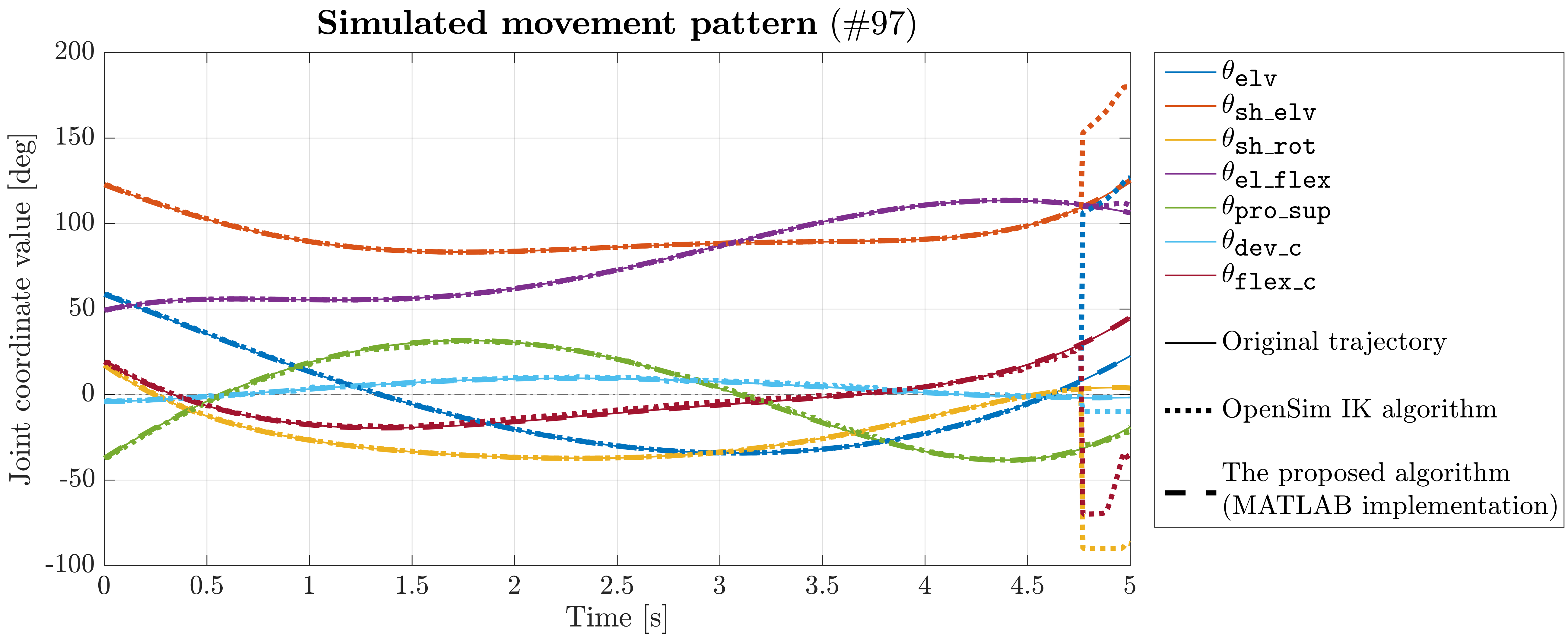
Simulated movement pattern (#97)
Joint coordinate value [deg]
Time [s]
200
150
100
50
0
-50
-100
0
0.5
1
1.5
2
2.5
3
3.5
4
4.5
5
$\theta_{\mathtt{elv}}$
$\theta_{\mathtt{sh\_elv}}$
$\theta_{\mathtt{sh\_rot}}$
$\theta_{\mathtt{el\_flex}}$
$\theta_{\mathtt{pro\_sup}}$
$\theta_{\mathtt{dev\_c}}$
$\theta_{\mathtt{flex\_c}}$
Original trajectory
OpenSim IK algorithm
The proposed algorithm
(MATLAB implementation)

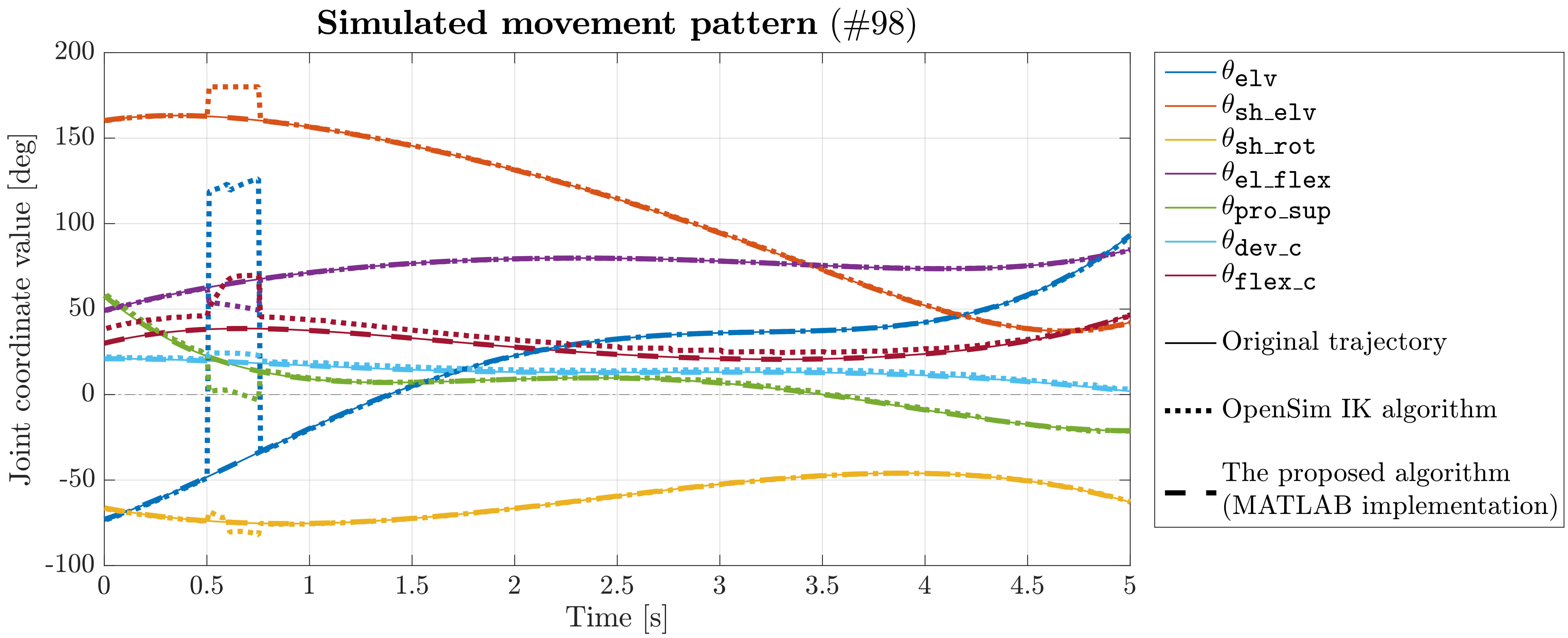

Simulated movement pattern (#98)
Joint coordinate value [deg]
Time [s]
200
150
100
50
0
-50
-100
0
0.5
1
1.5
2
2.5
3
3.5
4
4.5
5
$\theta_{\texttt{elv}}$
$\theta_{\texttt{sh\_elv}}$
$\theta_{\texttt{sh\_rot}}$
$\theta_{\texttt{el\_flex}}$
$\theta_{\texttt{pro\_sup}}$
$\theta_{\texttt{dev\_c}}$
$\theta_{\texttt{flex\_c}}$
Original trajectory
OpenSim IK algorithm
The proposed algorithm
(MATLAB implementation)

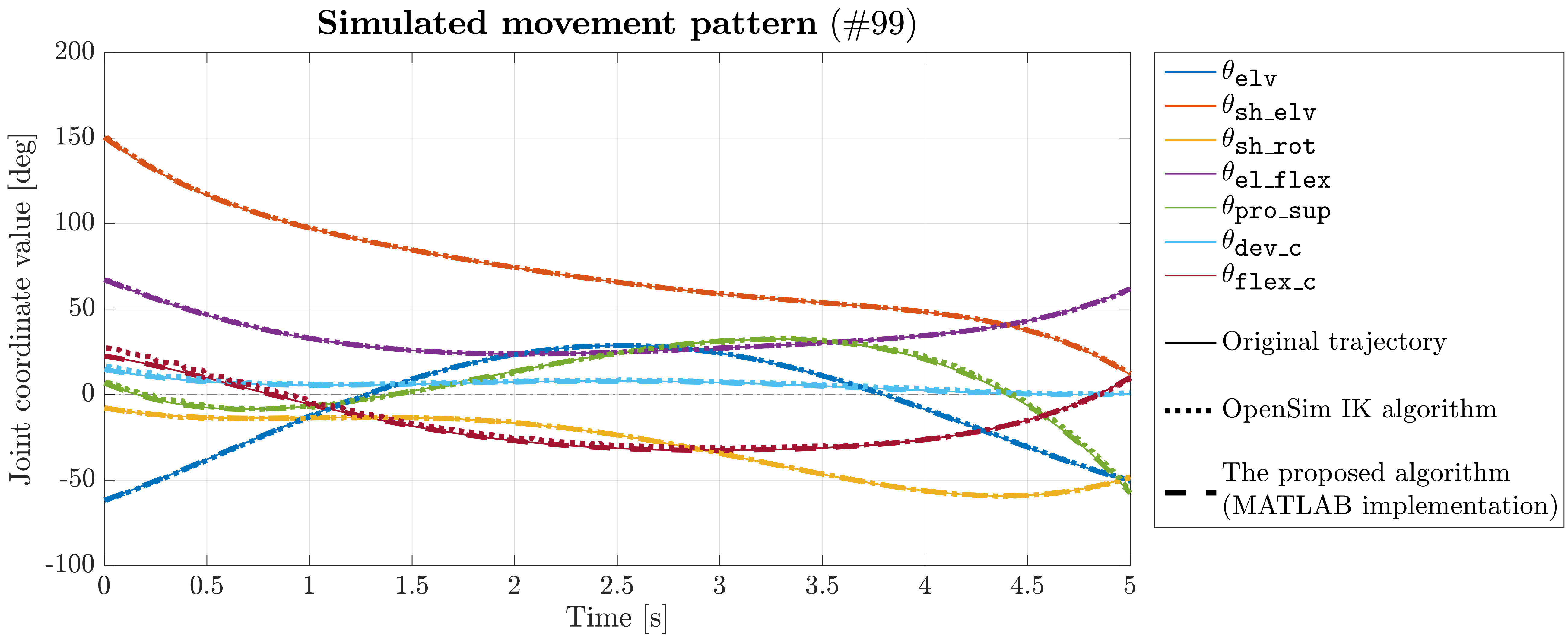

Simulated movement pattern (#99)
200
150
100
50
0
-50
-100
Joint coordinate value [deg]
0
0.5
1
1.5
2
2.5
3
3.5
4
4.5
5
Time [s]
θ_{elv}
θ_{sh_elv}
θ_{sh_rot}
θ_{el_flex}
θ_{pro_sup}
θ_{dev_c}
θ_{flex_c}
Original trajectory
OpenSim IK algorithm
The proposed algorithm (MATLAB implementation)

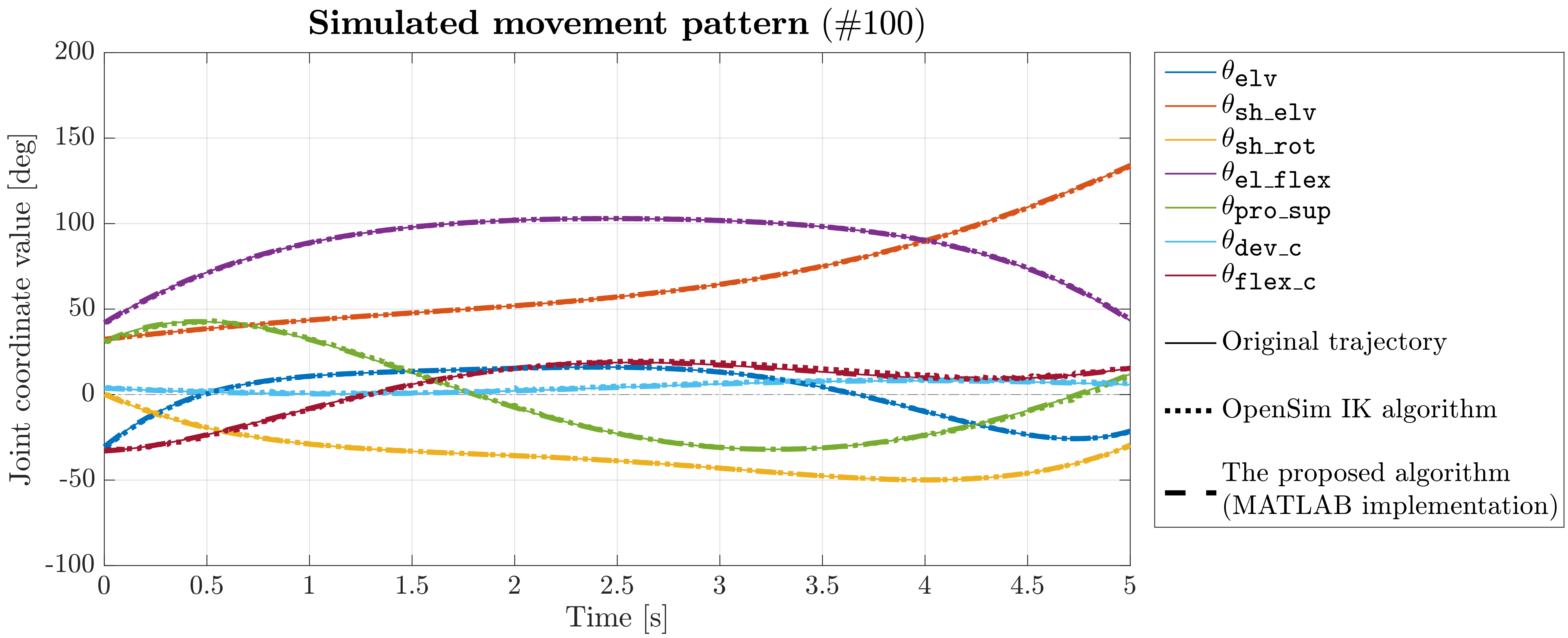

Simulated movement pattern (#100)
200
150
100
50
0
-50
-100
Joint coordinate value [deg]
0
0.5
1
1.5
2
2.5
3
3.5
4
4.5
5
Time [s]
θelv
θsh_elv
θsh_rot
θel_flex
θpro_sup
θdev_c
θflex_c
Original trajectory
OpenSim IK algorithm
The proposed algorithm
(MATLAB implementation)
